# Effects of Single Low Dose of Dexamethasone before Noncardiac and Nonneurologic Surgery and General Anesthesia on Postoperative Cognitive Dysfunction—A Phase III Double Blind, Randomized Clinical Trial

**DOI:** 10.1371/journal.pone.0152308

**Published:** 2016-05-06

**Authors:** Livia Stocco Sanches Valentin, Valeria Fontenelle Angelim Pereira, Ricardo S. Pietrobon, Andre P. Schmidt, Jean P. Oses, Luis V. Portela, Diogo O. Souza, João Ricardo Nickenig Vissoci, Vinicius Fernando da Luz, Leticia Maria de Araujo de Souza Trintoni, Karen C. Nielsen, Maria José Carvalho Carmona

**Affiliations:** 1 Department of Anesthesia, LIM 8 –Laboratory of Anesthesiology, Faculdade de Medicina da Universidade de São Paulo, São Paulo, São Paulo, Brazil; 2 Department of Surgery, Duke University Health System, Durham, North Carolina, United States of America; 3 Department of Biochemistry, Federal University of Rio Grande do Sul, Porto Alegre, Rio Grande do Sul, Brazil; 4 Department of Anesthesia and Perioperative Medicine, Hospital de Porto Alegre, Porto Alegre, Rio Grande do Sul, Brazil; 5 Center of Sciences of the Life and Health, Universidade Católica de Pelotas, Pelotas, Rio Grande do Sul, Brazil; 6 Department of Anesthesiology, Duke University Medical Center, Durham, North Carolina, United States of America; Hungarian Academy of Sciences, HUNGARY

## Abstract

Postoperative cognitive dysfunction (POCD) is a multifactorial adverse event most frequently in elderly patients. This study evaluated the effect of dexamethasone on POCD incidence after noncardiac and nonneurologic surgery. METHODS: One hundred and forty patients (ASA I-II; age 60–87 years) took part in a prospective phase III, double blind, randomized study involving the administration or not of 8 mg of IV dexamethasone before general anesthesia under bispectral index (BIS) between 35–45 or 46–55. Neuropsychological tests were applied preoperatively and on the 3rd, 7th, 21st, 90th and 180th days after surgery and compared with normative data. S100β was evaluated before and 12 hours after induction of anesthesia. The generalized estimating equations (GEE) method was applied, followed by the posthoc Bonferroni test considering P<0.05 as significant. RESULTS: On the 3rd postoperative day, POCD was diagnosed in 25.2% and 15.3% of patients receiving dexamethasone, BIS 35–45, and BIS 46–55 groups, respectively. Meanwhile, POCD was present in 68.2% and 27.2% of patients without dexamethasone, BIS 35–45 and BIS 46–55 groups (p<0.0001). Neuropsychological tests showed that dexamethasone associated to BIS 46–55 decreased the incidence of POCD, especially memory and executive function. The administration of dexamethasone might have prevented the postoperative increase in S100β serum levels. CONCLUSION: Dexamethasone can reduce the incidence of POCD in elderly patients undergoing surgery, especially when associated with BIS 46–55. The effect of dexamethasone on S100β might be related with some degree of neuroprotection.

***Trial Registration*:** www.clinicaltrials.gov NCT01332812

## Introduction

Postoperative cognitive dysfunction (POCD) is frequently reported following general anesthesia, especially in elderly patients and after cardiac surgery. POCD was defined as a cognitive decline in two or more neuropsychology functions, according to the International Study of Postoperative Cognitive Dysfunction (ISPOCD) study group. Global cognitive function was evaluated by a neuropsychology battery, and the Z-score method was applied as a measure of cognition[1].

The ISPOCD demonstrated that in geriatric patients submitted to non-cardiac surgery under general anesthesia, this adverse event affected up to 26% of them, one week after the procedure. After three months of operation, POCD remained in up to 10% of that group[2,3]. In elderly patients, POCD increased the risk of comorbidities in the first year after surgery[4]. However, there is no consensus about precipitating factors and preventive measures for cognitive dysfunction in surgical patients. Besides age, the postoperative cognitive function may be compromised by hypoxia, hypotension, anesthetics and depth of anesthesia, surgical procedures and external factors such as quality of life[5]. Patients might present different levels of mental status. It can vary from mental confusion to memory loss. Clinically, it is shown as a decrease in the consciousness level, also known as delirium.

Surgery can result in a complex neuronal system response, which includes inflammation and may directly affect patient outcome[6]. Data support the concept that inflammation and brain injury are a possible pathogenic mechanism for POCD. S100b is a known biomarker related to brain injury. It is released into blood circulation in early stages of brain suffering when glial cells are activated [7].

Corticosteroids such as dexamethasone are often used as additional drugs in anesthesia to mitigate its side effects, such as pain, vomiting and fatigue, all of which are physical symptoms that compromise cognitive functioning[8,9]. The use of dexamethasone was never investigated as a possible protective factor against cognitive impairment in surgery.

This study aimed to evaluate the effect of dexamethasone on the incidence of postoperative cognitive dysfunction in elderly patients undergoing surgery under general anesthesia. It is registered in www.clinicaltrials.gov Identifier: NCT01332812.

## Materials and Methods

### Study design and subject enrollment

The study was conceived as a randomized, double-blind and prospective clinical trial. After approval by the “Comissão de Etica para Analise de Projeto de Pesquisa do Hospital das Clinicas da Faculdade de Medicina da Universidade de São Paulo”(CAPPesq-HCFMUSP) S1 File, and “Comissão Nacional de Etica em Pesquisa”(CONEP) S2 File, and receiving written informed consent, 140 patients (aged 60 years or older) who were candidates for elective noncardiac and nonneurologic surgery in a university hospital between January 2011 and February 2012 were enrolled in the study, and the neuropsychological assessment was completed in March 2013. www.clinicaltrial.gov was not realized before the beginning of the study patient recruitment period, because at that moment, in Brazil, only CAPPesq-HCFMUSP and CONEP S2 File registrations were required for that. Later, the www.clinicaltrial.gov registration was performed. Identifier: NCT01332812.

Inclusion criteria were as follows: candidates for noncardiac and nonneurologic surgery under general anesthesia that was not expected to exceed 6 hours, no history of brain disease, dementia or other psychiatric disorders that affect cognition, literate, and no continuous use corticosteroids, antidepressants or opioids preoperatively.

The mental state was preliminarily assessed using the Mini-Mental State Examination (MMSE), which is validated for the Brazilian population as a brief screening instrument designed to measure and quantify the global cognitive state, assessing reasoning, spatial-temporal orientation, memory, and schooling. The test uses for the Brazilian people a cut-off of 18 points for subjects who have completed up to 4 years of formal academic study and 23 points for individuals with higher levels of education. Patients were excluded if they had an MMSE score below 18 or 23 by educational level[10].

#### Preoperative evaluation and neuropsychological assessment

Demographic data were analyzed, including education, marital status, occupation, and current medication. Signs and symptoms of depression were assessed preoperatively using the Beck Depression Inventory (BDI), which consists of 21 questions that explore depressive symptoms on a scale of 0 to 4, where zero represents no symptoms and four is the maximum symptomatology. A total of 14 points was considered indicative of the presence of moderate depressive symptoms[11]. The quality of life was investigated using the Short Form Health Survey (SF-36) to investigate health-related conditions, activities of daily living, productivity, emotional problems, relationships, and motivation, and one question about the perception of the patients itself[12].

The Telephone Interview for Cognitive Status (TICS) was used as a standardized test to assess neuropsychological functioning when assessing cognitive skills. This test was used when screening personally was impractical or when patients were unable to attend the clinic. This test consists of a structured interview with 11 items that assess the skills of spatial and temporal orientation, mental control, memory, general information, language, and calculations[13].

POCD diagnosis was performed by TICS and Neuropsychological Battery, near the seventh postoperative day.

The specific neuropsychological battery test, based on the International Studies of POCD 1 and 2 translated and adapted for the individually studied population, was performed one day before surgery (baseline) and on the 3^rd^, 7^th^, 21^st^, 90^th^ and 180^th^ postoperative day. The cognitive functions assessed were memory, attention and executive function in aspects such as strategy, elaboration of thought and inhibiting the control of actions. Tests used for the evaluation of these skills are redundant, especially for the assessment of attention.

Memory was evaluated using the Rey-Auditory Verbal Learning Test (RAVLT), which consists of a list of 15 words to be memorized and recalled on three successive attempts, with delayed recall after 15 to 25 minutes, evaluating the number of words recalled and the number of errors made for each presentation[14].

The Symbol Digit Modified Test (SDMT) was applied for the evaluation of short-term memory, visual search skill, and attention. SDMT is a graphical task where the individual has to fill in symbols exemplified in the spaces below the corresponding number within 180 seconds. The result is measured as the number of symbols drawn and the number of errors[14].

Attention was also evaluated using the Trail Making Test (TMT) for the assessment of selective and alternating attention. In part A of the test, the subject must consecutively draw lines connecting numbered circles. In part B, the subject must draw lines connecting circles alternately with letters and numbers in a sequence. The result is measured as time and trail errors[14].

#### Criteria for POCD diagnosis

TICS values were used to evaluate the evolution of general cognition postoperatively and were applied in every phase’s pre and postoperatively. For the diagnosis of POCD, a composite cognitive index was established defined by the occurrence of cognitive impairment in TICS and at least 1 of 8 possible deficits of the others neuropsychologist tests. Changes in neuropsychological tests have been compared with the results of tables of normative subjects matched for age, gender, and formal education[15].

#### Assessment of serum levels of S100β

Serum levels of S100β protein were obtained from venous blood samples collected before anesthesia induction and 12 hours after surgery. Samples were placed in dry tubes and centrifuged; serum was removed and stored to -80°C until the analysis; and S100β protein was measured by a commercially available S100β enzyme-linked immune absorbent assay, (ELISA) kit (Diasorin, Italy). A quantitative monoclonal 2-site ELISA microplate assay in which the last antibody added to the reaction system is labeled with peroxidase. After the addition of a peroxidase substrate, the reaction produces a final color product read in a spectrophotometer. S100β levels were expressed as micrograms per milliliter[16].

#### Randomization and anesthesia technique

Immediately before anesthesia induction patients were randomized to receive or not 8 mg of dexamethasone, stratified into subgroups according to the bispectral index (BIS) and allocated into BIS 46–55 or 35–45 subgroups inside the operating room. The randomization was performed using the website http://www.randomizer.org/form.htm, recorded on paper and stored in sealed opaque envelopes to be opened in the operating room. Anesthesiologists were aware of the BIS allocated arm when the patients entered in the operating room, but patients, neuropsychologists, laboratory technicians and outcome assessors involved in the study were kept blinded to the stratification group to which each patient belonged. The study groups were identified only after the evaluation of all patients and statistical analysis.

In the operating room, standard monitoring included continuous registration of electrocardiogram at DII, pulse oximetry, non-invasive blood pressure, end tidal CO_2_ and bispectral index (BIS). General anesthesia was induced with propofol (1.0–2.5 mg/kg), fentanyl (2.0–3.0 μg/kg) and cisatracurium (0.10–0.2 mg/kg). Maintenance of anesthesia was performed with propofol target-controlled infusion according to Schneider’s pharmacokinetic model for the intended BIS-value subgroup. The remifentanil (0.10 to 1.0 μg/kg/ min) or fentanyl (intermittent doses of 2.0–3.0 μg/kg) were added according to anesthesiologist judgment, plus cisatracurium in supplemental doses (0.03–0.05 mg/kg). Glycaemia was controlled perioperatively; after anesthesia induction. Insulin was given when blood glucose concentrations exceeded 180 mg/dL. At the end of surgery and after tracheal extubation, patients were transferred to the post-anesthesia care unit. The control of postoperative pain was conducted following institutional protocols.

The CONSORT criteria (www.consort-statement.org) was followed S3 File.

### Statistical analyses

**Fig 1:**
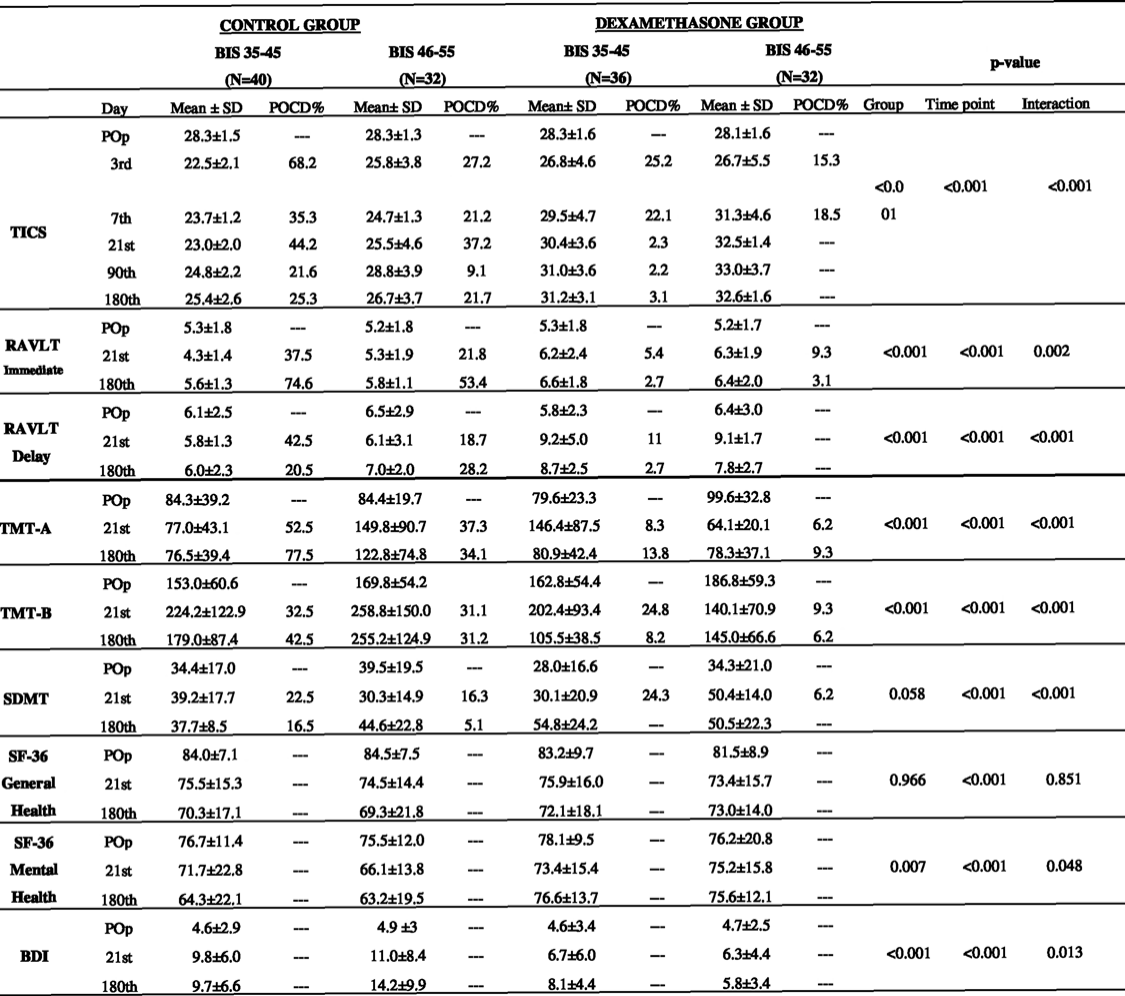
Pre and postoperative results of the specific test: for global cognitive, memory, attention, executive functions, and Quality of life and depressive symptoms, compared to normative data ^a^. BIS: bispectral index; POCD: postoperative cognitive disorder; TICS: Telephone interview for cognitive status; RAVLT immediate: Rey Auditory- verbal learning test (immediate); RAVLT delay: Rey Auditory- verbal learning test (delay); TMT-A: Trail making test- A; TMT-B: Trail making test-B; SDMT: symbol digit modalities—Test; SF-36 general health: Short form health survey (general health); SF-36 Mental Health: Short form health survey (mental health); BDI: Back depression inventory. ^a^ Normative values. Data are mean ± SD and percentage for patients with POCD. TICS: 27±3; VLT: immediate 6.3±2.1; delay 10.2±2.5; TMT-A ≥ 55 and TMT-B ≥137; SDMT 50.1±8.1; SF-36 scale >60±10; BDI <12; Pop = Preoperative; Postoperative days = 3^rd^; 7^th^; 21^st^; 90^th^ and 180^th^; ^b^ *p-value for the Generalized Estimating Equations (GEE).

It was estimated a sample size of 138 patients in the study. The sample size was based upon a 40% POCD incidence in elderly patients as previously described in the literature[17]. As was showed before, there is a possible improvement of 20% in POCD with dexamethasone use during surgery[18]. It was considered a maximum error of 5%, and a statistical power of 80%. The results of neuropsychological tests used to evaluate cognitive function were described for the two groups; each one stratified in two arms. The tests values were analyzed according to the 6 phases of evaluation (Fig 1). The interaction between time point and groups were tested. The results were shown as means and standard deviation. The comparison between the four groups, according to the six evaluation phases were analyzed by using Generalized Estimation Equation (GEE). Data was evaluated under the intention to treat analysis[15].

Changes in neuropsychological tests have been compared with the results of the table of normative subjects (Fig 1), matched for age, gender, and formal education, using Z-score analysis[19]. General linear mixed modeling was applied. Imputation treated the Missing data, which did not exceed 12%. Bonferroni multiple comparisons were used to complete the evaluation Statistical calculations. They were performed using SPSS12 and GraphPad Prism version 6.00 for Mac (GraphPad Software, La Jolla California USA www.graphpad.com).

## Results

**Fig 2:**
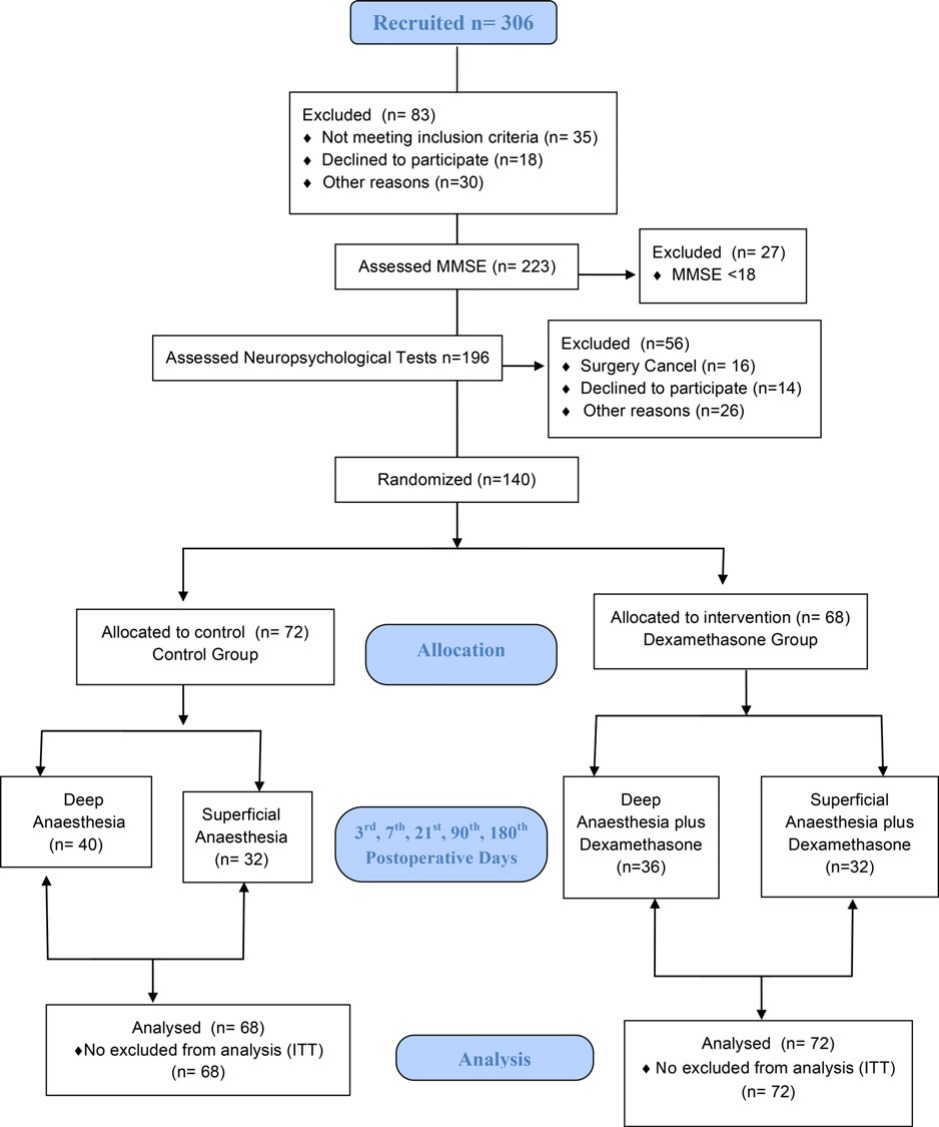
Study Fluxogram. MMSE: Mini-Mental State Examination; ITT: Intention to treat.

Three hundred and six patients were recruited for elective surgery under general anesthesia, but 83 patients were excluded because they are not in the inclusion criteria or declined to participate in the study or for other reasons not justified by the patient. Two hundred and twenty-three patients were assessed with the MMSE cognitive screening test, and 27 patients were excluded after failing to normative score (≤ 18 or ≤ 23 points) according to education levels. One hundred and ninety-six patients were assessed using neuropsychological battery tests adapted from ISPOCD and 140 patients were ultimately randomized and evaluated under the intention to treat[15] analysis up to an 180^th^ postoperative day (Fig 2).

**Fig 3:**
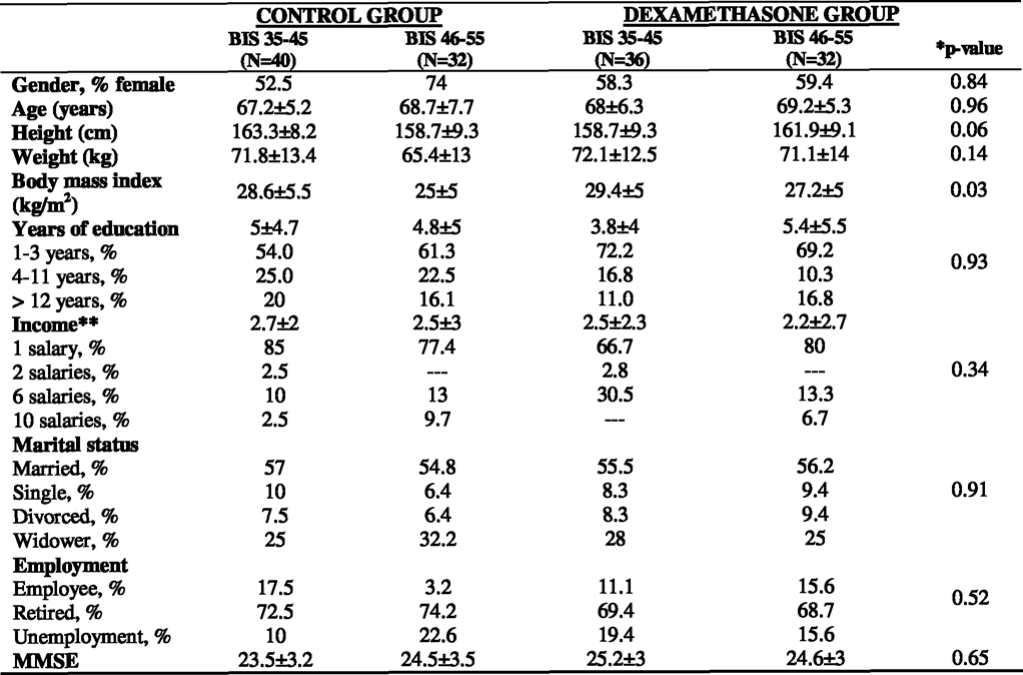
Baseline and demographic data. MMSE: Mini-Mental State Examination; *p-value >0.05; **Salary rates equivalent to current Brazilian minimum salary in 2012 ($331.129 USD); *** Data are mean ± SD or per cent.

**Fig 4:**
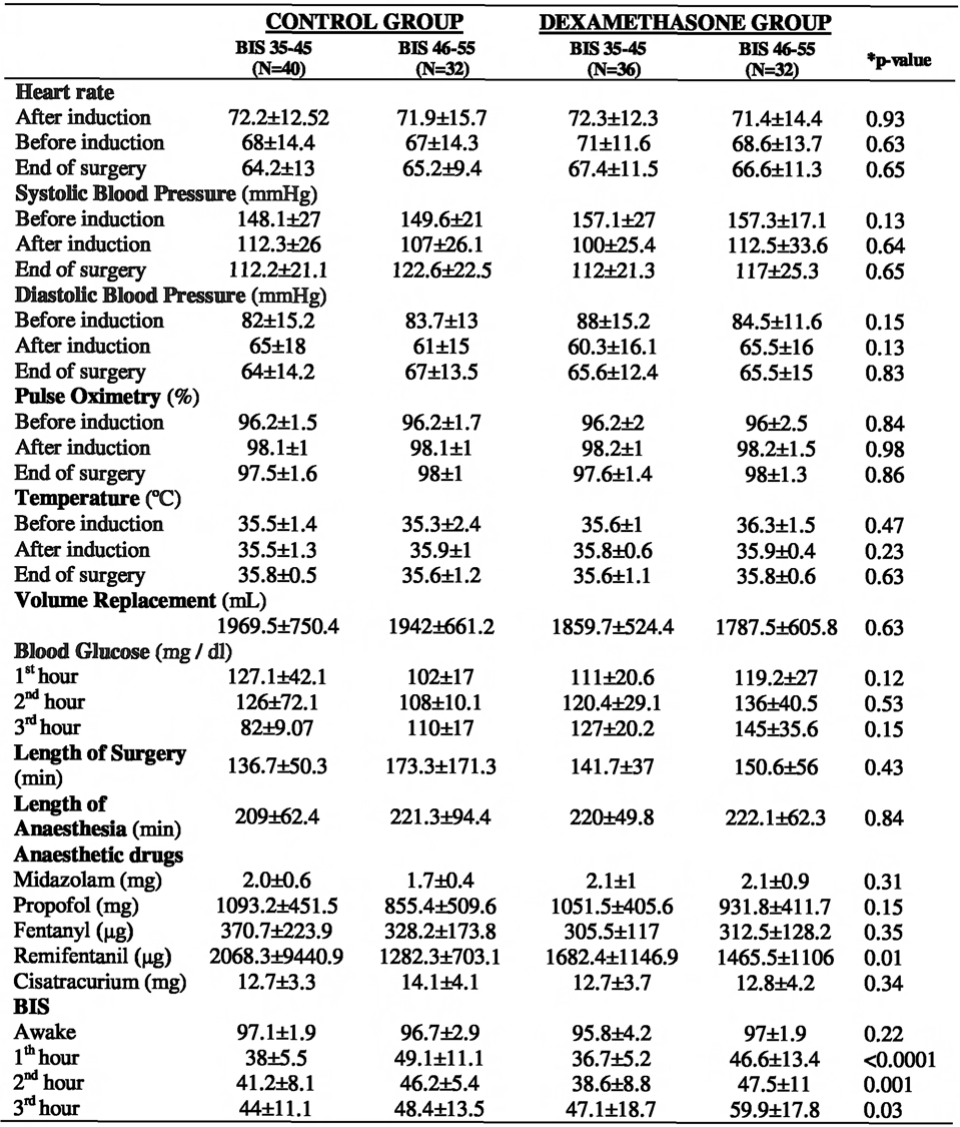
Intraoperative data. Data are mean ± SD; BIS: bispectral index; *p-value for the Generalized Estimating Equations (GEE).

There was no statistical difference between groups concerning baseline and demographic data (Fig 3), and the intraoperative data are shown in (Fig 4). No reports of intraoperative recall were related. The mean bispectral index values were comparable between groups at baseline but differed according to anesthesia procedure (Fig 4). The results for global cognitive function evaluated by TICS (Fig 1) showed that the highest incidence of POCD on the 3^rd^ postoperative day was observed in the BIS 35–45 group without preoperative dexamethasone shot (68.2%, p<0.0001). After six months, the same group showed the highest incidence of POCD, although the cognitive function improved compared to 3^rd^ postoperative day (25.3%, p<0.0001). The group receiving BIS 46–55 and dexamethasone at anesthesia induction showed the lowest incidence of POCD during the study. Their global cognitive function according to TICS was recovered on the 21^st^, 90^th^ and 180^th^ postoperative days. The cut-off point was (27 ± 3) and the three cited postoperative days presented the values (32.5±1.4; 33±3.7 and 32.5±1.6), respectively.

Regarding memory function (immediate and delayed) assessed via RAVLT, Cognitive dysfunction was most impaired in the groups without dexamethasone, independently of their BIS group. There was a significant difference during postoperative phases between groups for immediate memory (p = 0.0002) and delayed recall (p = 0.001). Regarding immediate memory, the highest incidence of dysfunction was observed on the 180^th^ day. (74.6% and 53.4%) For both groups: BIS 35–45 and BIS 46–55, respectively. For delayed recall the most impaired cognitive function was 42.5% in the BIS 35–45 group without dexamethasone on the 21^st^ day. The BIS 46–55 plus dexamethasone group showed no dysfunction for delayed recall. For the Selection Attention Function assessed by the TMT in part A, Group BIS 35–45 without dexamethasone presented cognitive dysfunction in 77.5% (p<0.0001) on the 180^th^ day. Meanwhile, its group ability to toggle attention, assessed by TMT part B showed an 42% of impairment on the 180^th^ day (p = 0.001). For executive function, visual and motor skills, evaluated by the SDMT, The groups receiving dexamethasone showed no executive dysfunction on the 180^th^ postoperative day (Fig 1).

Based on the SF-36 results, (Fig 1), all groups showed general good health at the end of the study (p = 0.09). All groups showed the absence of depressive symptoms in the preoperative assessment. At the end of the study, the group who received dexamethasone and BIS 46–55 showed a lower mean for depressive symptoms (5.8±3.4) assessed by the BDI (Fig 1).

**Fig 5:**
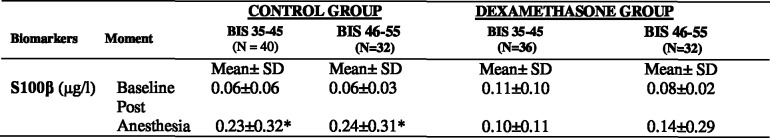
S100β serum level. BIS: bispectral index; Mean ± SD: Mean ± standard deviation. * = p<0.05 as compared to baseline biomarkers S100β level.

S100β serum levels were significantly increased 12 hours after surgery in patients without dexamethasone submitted to either BIS 35–45 or BIS 46–55 (p<0.05) (Fig 5).

## Discussion

This study found that patients receiving a single dose of dexamethasone at induction of anesthesia presented a lower incidence of postoperative cognitive dysfunction. When this corticosteroid was associated with BIS 46–55 rather than BIS 35–45, their cognitive functions established more rapidly. A better quality of life was also observed in the postoperative period in the dexamethasone groups, as a lower incidence of depressive symptoms.

In anesthesia practice, patients who have postoperative injuries, such as pain, nausea, and vomiting return to daily activities in more time than patients who presented fewer postoperative symptoms. Nowadays, dexamethasone is commonly used in the perioperative setting by anesthetists, both for its antiemetic properties and its ability to reduce airway swelling and fatigue. Our findings corroborate some of the observations made in other studies on corticosteroids such as the decrease in the postoperative recovery time. The inhibitory effect of dexamethasone on inflammation could also prevent some undesirable neuropsychological adverse events such as cognitive dysfunction, delirium, and even stroke[8,20]. Until now, no specific study have evaluated the possible effects of dexamethasone on the incidence of POCD in patients undergoing general surgery.

Although some controversy remains, several authors were able to demonstrate a relationship between the degree of cognitive dysfunction and S100β protein[6,21], which is consistent with our present findings. In the present study, S100β postoperative injuries serum levels were significantly elevated after surgery regardless BIS values. The glial cells are activated, In the early stages of brain injury, and S100β is released into blood circulation[7]. The dexamethasone group presented a smaller incidence of cognitive dysfunction. During surgical procedures without direct brain involvement; S100B increase might be due a variety of clinical states that might cause BBB disruption[20,22].

In our study, an enormous neuropsychological battery evaluated the cognitive function. Some cognitive functions are assessed by more than one test. So, we have chosen a specific test for each cognitive function evaluation. It is not a big limitation but a concern regarding data analysis. We have tried to avoid data duplication that might lead to an overestimation of cognitive function impairment. For example, the selective attention was evaluated only by TMT-A. The long-term memory was assessed only by VLT.

Our study found significant differences between groups concerning the BIS values. POCD incidence was increased in BIS 46–55 compared to BIS 35–45. The data also showed a more rapid cognitive function restoration in the last group, mainly in the group that received dexamethasone. We can suggest that anesthesia depth might influence the POCD grade and its recovery. Some other studies have shown that superficial anesthesia may benefit the patient and prevent postoperative cognitive dysfunction[23,24].

Although our study demonstrated the benefit of dexamethasone associated with BIS 46–55 regarding prevention of cognitive dysfunction, other factors should be investigated. Demographic data might have a significant influence on cognitive reserve. People with a higher educational level tend to be healthier than people with a lower educational level old age, and comorbidities may contribute as risk factors for POCD. Cognitive decline is inevitable during the aging process, but this impairment may also be associated with surgery and anesthesia.

One limitation of the current study was the loss of follow-up for some patients. Sometimes, they were not able to attend the assessments face to face in the hospital.

In conclusion, dexamethasone when administered with BIS 46–55 can help to preserve most cognitive functions in surgery. Additional studies could contribute to defining the neuroprotective properties of dexamethasone in the prevention of postoperative cognitive dysfunction in patients undergoing surgery.

## Supporting Information

S1 File CAPpesq appoval. (DOCX)

S2 File Cappesq_no99806_Disfuncao_Cognitiva-TODOS_OS_DOCUMENTOS 5. (ZIP)

S3 File CONSORT Checklist Completed 021216. (DOC)

## References

[1] Newman S, Stygall J, Hirani S, Shaefi S, Maze M (2007) Postoperative cognitive dysfunction after noncardiac surgery: a systematic review. Anesthesiology 106: 572–590. 17325517 10.1097/00000542-200703000-00023

[2] Abildstrom H, Rasmussen LS, Rentowl P, Hanning CD, Rasmussen H, et al (2000) Cognitive dysfunction 1–2 years after non-cardiac surgery in the elderly. ISPOCD group. International Study of Post-Operative Cognitive Dysfunction. Acta Anaesthesiol Scand 44: 1246–1251. 11065205 10.1034/j.1399-6576.2000.441010.x

[3] Rasmussen LS, investigators I (2005) Post-operative cognitive dysfunction in the elderly. Acta Anaesthesiol Scand 49: 1573. 10.1111/j.1399-6576.2005.00860.x 16223411

[4] Damuleviciene G, Lesauskaite V, Macijauskiene J (2010) [Postoperative cognitive dysfunction of older surgical patients]. Medicina (Kaunas) 46: 169–175. 20516755

[5] Jungwirth B, Zieglgansberger W, Kochs E, Rammes G (2009) Anesthesia and postoperative cognitive dysfunction (POCD). Mini Rev Med Chem 9: 1568–1579. 20088778 10.2174/138955709791012229

[6] Peng L, Xu L, Ouyang W (2013) Role of peripheral inflammatory markers in postoperative cognitive dysfunction (POCD): a meta-analysis. PLoS One 8: e79624 10.1371/journal.pone.0079624 24236147 PMC3827367

[7] Donato R, Cannon BR, Sorci G, Riuzzi F, Hsu K, et al (2013) Functions of S100 proteins. Curr Mol Med 13: 24–57. 22834835 PMC3707951

[8] Jakobsson J (2010) Preoperative single-dose intravenous dexamethasone during ambulatory surgery: update around the benefit versus risk. Curr Opin Anaesthesiol 23: 682–686. 10.1097/ACO.0b013e32833ff302 20847689

[9] Gomez-Hernandez J, Orozco-Alatorre AL, Dominguez-Contreras M, Oceguera-Villanueva A, Gomez-Romo S, et al (2010) Preoperative dexamethasone reduces postoperative pain, nausea and vomiting following mastectomy for breast cancer. BMC Cancer 10: 692 10.1186/1471-2407-10-692 21182781 PMC3017064

[10] Bertolucci PH, Brucki SM, Campacci SR, Juliano Y (1994) [The Mini-Mental State Examination in a general population: impact of educational status]. Arq Neuropsiquiatr 52: 1–7. 8002795

[11] Gorenstein C, Andrade L (1996) Validation of a Portuguese version of the Beck Depression Inventory and the State-Trait Anxiety Inventory in Brazilian subjects. Braz J Med Biol Res 29: 453–457. 8736107

[12] Cruz LN, Fleck MP, Oliveira MR, Camey SA, Hoffmann JF, et al (2013) Health-related quality of life in Brazil: normative data for the SF-36 in a general population sample in the south of the country. Cien Saude Colet 18: 1911–1921. 23827895 10.1590/s1413-81232013000700006

[13] Lopez OL, Kuller LH (2010) Telephone interview for cognitive status. Neuroepidemiology 34: 63–64. 10.1159/000264678 20016214 PMC2857624

[14] Strauss E SE SO (Third Edition) A Compendium of Neuropsychological Tests: Administration, Norms, and Commentary Oxford University Press 1: 1–1225.

[15] Moller JT, Cluitmans P, Rasmussen LS, Houx P, Rasmussen H, et al (1998) Long-term postoperative cognitive dysfunction in the elderly ISPOCD1 study. ISPOCD investigators. International Study of Post-Operative Cognitive Dysfunction. Lancet 351: 857–861. 9525362 10.1016/s0140-6736(97)07382-0

[16] Böhmer AE OJ, Schmidt AP, Perón CS, Krebs CL, Oppitz PP, D'Avila TT, Souza DO, Portela LV, Stefani MA. (2011) Neuron-specific enolase, S100B, and glial fibrillary acidic protein levels as outcome predictors in patients with severe traumatic brain injury. Neurosurgery 68: 1624–1630. 10.1227/NEU.0b013e318214a81f 21368691

[17] Johnson T, Monk T, Rasmussen LS, Abildstrom H, Houx P, et al (2002) Postoperative cognitive dysfunction in middle-aged patients. Anesthesiology 96: 1351–1357. 12170047 10.1097/00000542-200206000-00014

[18] Abdelmalak B, Maheshwari A, Mascha E, Srivastava S, Marks T, et al (2010) Design and Organization of the Dexamethasone, Light Anesthesia and Tight Glucose Control (DeLiT) Trial: a factorial trial evaluating the effects of corticosteroids, glucose control, and depth-of-anesthesia on perioperative inflammation and morbidity from major non-cardiac surgery. BMC Anesthesiol 10: 11 10.1186/1471-2253-10-11 20591163 PMC2910009

[19] Canet J, Raeder J, Rasmussen LS, Enlund M, Kuipers HM, et al (2003) Cognitive dysfunction after minor surgery in the elderly. Acta Anaesthesiol Scand 47: 1204–1210. 14616316 10.1046/j.1399-6576.2003.00238.x

[20] Vachon P, Moreau JP (2003) Low doses of dexamethasone decrease brain water content of collagenase-induced cerebral hematoma. Can J Vet Res 67: 157–159. 12760484 PMC227046

[21] Heyer EJ, Connolly ES (2003) Serum concentration of S-100 protein in assessment of cognitive dysfunction after general anesthesia in different types of surgery. Acta Anaesthesiol Scand 47: 911–912; author reply 912–913. 12859318 10.1034/j.1399-6576.2003.00176.x

[22] Marchi N, Cavaglia M, Fazio V, Bhudia S, Hallene K, et al (2004) Peripheral markers of blood-brain barrier damage. Clin Chim Acta 342: 1–12. 15026262 10.1016/j.cccn.2003.12.008

[23] Chen X, Zhao M, White PF, Li S, Tang J, et al (2001) The recovery of cognitive function after general anesthesia in elderly patients: a comparison of desflurane and sevoflurane. Anesth Analg 93: 1489–1494, table of contents. 11726429 10.1097/00000539-200112000-00029

[24] Gaba V (2007) Correlation of the depth of anesthesia with POCD (postoperative cognitive dysfunction). Anesth Analg 104: 1298; author reply 1298–1299. 17456698 10.1213/01.ane.0000260372.38206.b3

