# Supplementary figures and images for: Effects of Single Low Dose of Dexamethasone before Noncardiac and Nonneurologic Surgery and General Anesthesia on Postoperative Cognitive Dysfunction—A Phase III Double Blind, Randomized Clinical Trial
Source: PLoS One. 2016 May 6;11(5):e0152308. doi: 10.1371/journal.pone.0152308 (PMC4859565; doi:10.1371/journal.pone.0152308)

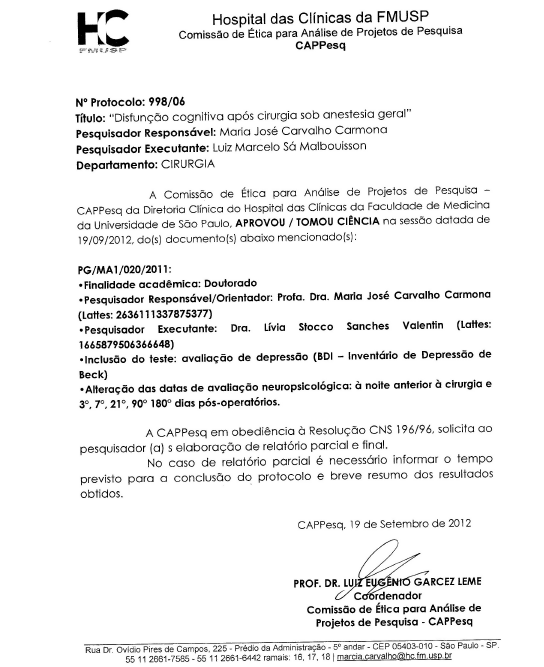

Supplement: S1 File — (DOCX) [file pone.0152308.s001.docx]
