# Supplementary material for: Effects of Single Low Dose of Dexamethasone before Noncardiac and Nonneurologic Surgery and General Anesthesia on Postoperative Cognitive Dysfunction—A Phase III Double Blind, Randomized Clinical Trial
Source: PLoS One. 2016 May 6;11(5):e0152308. doi: 10.1371/journal.pone.0152308 (PMC4859565; doi:10.1371/journal.pone.0152308)
Supplement: S2 File — (ZIP) [file pone.0152308.s002.zip › Cappesq_no99806_Disfuncao_Cognitiva-TODOS_OS_DOCUMENTOS 5.pdf]

Anexo II

HOSPITAL DAS CLÍNICAS

DA FACULDADE DE MEDICINA DA UNIVERSIDADE DE SÃO PAULO

CAIXA POSTAL, 8091 - SÃO PAULO - BRASIL

DIRETORIA CLÍNICA
COMISSÃO DE ÉTICA PARA ANÁLISE DE PROJETOS DE PESQUISA - CAPPesq

CADASTRO DE PROTOCOLO DE PESQUISA

Registro (uso reservado à Secretaria da CAPPesq)

Nº do Protocolo: ...998/06.......... Data de Entrada: ...29 SET. 2006...

1. Título do Protocolo de Pesquisa

DISFUNÇÃO COGNITIVA APÓS CIRURGIA SOB ANESTESIA GERAL

2. Palavras-chaves que caracterizam o assunto da Pesquisa

Anestesia Geral
Pós-Operatório
Disfunção Cognitiva

3. Resumo do Protocolo de Pesquisa:

Alguns dos determinantes de disfunção cognitiva pós-operatória são o uso de opióides no período pós-operatório, falta de atividade física, fadiga pós-operatória, a dor pós-operatória, a qualidade de vida, além de possível predisposição genética. O uso de corticóide pré-operatório diminui a dor pós-operatória, diminuindo também a necessidade de opióides, além de diminui a fadiga, a incidência de náusea e vômito, o período de recuperação e o tempo de retorno às atividades diárias no período pós-operatório, sendo freqüentemente utilizado como coadjuvante em anestesia. Os objetivos deste estudo são: 1) determinar a incidência de disfunção cognitiva no pós-operatório comparando dexametasona *versus* placebo em 150 pacientes submetidos à cirurgia sob anestesia geral; 2) Investigar a associação entre alterações cognitivas no pós-operatório e a presença do alelo ε4 do gene da apolipoproteína E e de marcadores bioquímicos séricos para lesão neuronal, como a proteína S100β e a enolase específica do neurônio (NSE). Serão aplicados testes neuropsicológicos para avaliar o estado mental, raciocínio e habilidade executiva, memória preliminar e secundária, língua, medida da memória e da concentração incluindo a velocidade e índice cognitivo da estabilidade (medida longitudinal do status cognitivo, que consiste em uma série de testes neurocognitivos repetidos que detectam estatísticamente mudanças significativas no funcionamento do sistema nervoso central). Esta avaliação será realizada pessoalmente antes da cirurgia e na noite após a cirurgia e por via telefônica nos dias pós-operatórios 3, 7, 21, e 90. A anestesia geral seguirá os padrões gerais incluindo Propofol (2-3 mg/kg), rocuronio (0.5 mg/kg), e fentanil (1-2 μg/kg) para indução. A anestesia subseqüente e mantida com sevoflurano (1.0-2.5%) e N_20 em 40% de oxigênio, rocuronio (doses suplementares serão administradas se necessário para manutenção de anestesia cirúrgica), e fentanil (doses suplementares 0.5-10 μg/kg com dose máxima de 250 μg). A administração de sevoflurano será ajustada com o objetivo de manter os níveis de hipnose avaliada pelo índice bispectral (BIS) de acordo com os grupos do estudo: os participantes serão randomizados em 2 grupos: anestesia superficial (índice bispectral entre 45 e 55) and anestesia profunda (índice bispectral entre 35 e 45). Ambos níveis são aceitáveis para anestesia geral e são atualmente usados por anestesiologistas. Após venóclise períférica, antes da indução anestésica, será colhida uma amostra de para determinação do perfil genotípico da Apoε e dosagem de S100β e NSE. Os resultados dos escores dos testes em cada grupo serão comparados através do teste de Friedmann, e entre os grupos será utilizado o teste de Kruskal Wallis. Porcentagens serão comparadas atráves do teste de Qui-Quadrado Os dados serão expressos como mediana e intervalo de confiança de 95%. Serão considerados significativos resultados cujos valor p sejam menores que 0,05 nos testes estatísticos.

RECEBIDO POR
DATA 29 SET. 2006
Comissão de Ética para Análise de Projetos de Pesquisa - CAPPesq - HCFMUSP

4. **Pesquisador Responsável**: Maria José Carvalho Carmona

5. **Pesquisador Executante**: Luiz Marcelo Sá Malbouisson

6. **Orientador**: Maria José Carvalho Carmona

7. **Especificação da finalidade acadêmica da pesquisa**

Graduação　　Pós-graduação { Doutorado / Mestrado

Outros - especificar: Investigação multicêntrica

8. **Unidades e Instituições envolvidas** (especificar)

- HOSPITAL DAS CLÍNICAS - FMUSP: Divisão de Anestesia do ICHC – Progamação de coleta de dados de 150 pacientes.
- ENTIDADES EXTERNAS:
  - Hospital Nossa Senhora do Carmo – Curitiba-PR - Progamação de coleta de dados de 75 pacientes.
  - Hospital Ministro Costa Cavalcanti da Fundacao Itaiguaipy – Londrina – PR - Progamação de coleta de dados de 75 pacientes.
  - Duke University – Não haverá coleta de dados neste centro, apenas a Coordenação do estudo referente à avaliação neuropsicológica e fornecimento de eletrodos para avaliação do Índice Bispectral.

9. **Pesquisa**:

X seres humanos　　animais (espécie): ....

10. **Investigação:**

Retrospectiva　　**X** Prospectiva

11. **Materiais e métodos:**

X Laboratorial　　Prontuários de pacientes

Peças anatômicas de cadáveres　　Tecidos, órgãos, fluídos orgânicos

X Entrevistas e questionários　　Outros: ....

12. **A Pesquisa envolve**: (preencher mais de um se necessário)

Isótopo Radioativo, Dispositivo Gerador de Radiação Ionizantes

Microorganismos Patogênicos

Ácidos Nucleares Recombinantes

Outros (especificar) : ....

13. Existe algum risco ambiental e/ou biológico com o descarte dos sub-produtos e/ou reagentes de sua pesquisa?

SIM X NÃO

14. Pesquisa em áreas temáticas especiais:

genética humana;
reprodução humana;
fármacos, medicamentos, vacinas e testes diagnósticos novos (fases I, II e III) ou não registrados no país (ainda que fase IV), ou quando a pesquisa for referente a seu uso com modalidades, indicações, doses ou vias de administração diferentes daquelas estabelecidas, incluindo seu emprego em combinações;
equipamentos, insumos e dispositivos para a saúde novos, ou não registrados no país;
novos procedimentos ainda não consagrados na literatura;
populações indígenas;
projetos que envolvam aspectos de biossegurança;
pesquisas coordenadas do exterior ou com participação estrangeira
pesquisas que envolvam remessa de material biológico para o exterior.

15. Gênero da pesquisa:

Clínica (Fisiopatológico, Terapêutico, Diagnóstico)

X Cirúrgica (Fisiopatológico, Terapêutico, Diagnóstico)

Experimental (Fisiopatológico, Terapêutico, Diagnóstico)

Anatômica

Epidemiológica

Teórica

16. Patrocínio:

Recursos Financeiros Solicitados (Serão solicitados após aprovação CAPPesq)

Instituições	Valores	Instituições	Valores
CNPq	R$20.000,00	Fundo Pesquisa FEJZ.	
FINEP		HC-FMUSP	
CAPES		Industrias:	
FAPESP		Laboratórios:	
F.F.M.		Outros: Duke University	materiais

O valor do recurso financeiro a ser solicitado ao CNPQ ou FAPESP corresponde à aquisição do material necessário para a avaliação da presença da APOε4 e S100β. Os eletrodos para monitorização do índice bispectral (BIS) e os formulários para aplicação dos testes neuropsicológicos serão fornecidos pela Duke University.

17. Existência de infraestrutura e recursos humanos para desenvolvimento da pesquisa (especificar).

A Divisão de Anestesia do Instituto Central do Hospital das Clínicas dispõe de Infra-estrutura e recursos humanos para desenvolvimento desta pesquisa.

18. Cronograma de execução da pesquisa

início: _01_/_09_/__2006__ término: _31_/__08_/_2008_

Prazo: 02 anos

19. Parecer da Comissão de Pesquisa e/ou de Ética do Departamento da FMUSP ou da entidade envolvida.

APROVADO

PROF. DR. ROLF GEMPERLI
Presidente da Comissão Assessora de Pes[illegible]
Departamento de Cirurgia da FM[illegible]

20. Conselho de Departamento da FMUSP

Aprovado " Ad referendum" do Conselho do Departamento de Cirurgia em 10/08/2006

PROF. DR. MARCUS CASTRO FERREIRA
Chefe do Departamento de Cirurgia
FMUSP

Assinatura
Carimbo

Aprovado em ____/______/_______

21. Parecer do Serviço de Verificação de Óbitos da Capital-SVOC, no caso de pesquisas realizadas em peças anatômicas de cadáveres necropsiados naquele Serviço.

Assinatura
Carimbo

Aprovado em ____/______/______

/tsc.

Anexo III

HOSPITAL DAS CLÍNICAS DA
FACULDADE DE MEDICINA DA UNIVERSIDADE DE SÃO PAULO

DIRETORIA CLÍNICA
COMISSÃO DE ÉTICA PARA ANÁLISE DE PROJETOS DE PESQUISA
CAPPesq

CADASTRO DO PESQUISADOR RESPONSÁVEL

---

1. Identificação e qualificação do pesquisador:

Nome: MARIA JOSÉ CARVALHO CARMONA ....RG:17 760 864.......................................

CPF: .059 174 158 / 03....................................Profissão: MÉDICA ....

Endereço: Rua Rodésia 161 – apto 82 - Vila Madalena...

CEP: .05435 -020.. Tefefone: 011-3816 3986 ....

---

2. Graduado em ...Medicina.. Data: 1985 ....

Instituição: UNICAMP..

3. Titulação acadêmica mais elevada: Livre Docente em Medicina ....

---

4. Vinculação Profissional Atual

X HC **X** FMUSP **X** Outras Instituições: Fundação E.J.Zerbini

Função: Professora Associada da Disciplina de Anestesiologia da FMUSP
Diretora da Divisão de Anestesia do Instituto Central do HCFMUSP

Lotação: Departamento de Cirurgia da FMUSP, Divisão de Anestesia do HCFMUSP

---

5. Vinculação Acadêmica Atual

Graduação Pós-Graduação { .Doutorado: Mestrado

X Outras: Orientadora do Programa de Pós-Graduação em Anestesiologia - FMUSP

---

6. Área do conhecimento : Anestesiologia - Terapia Intensiva ....

7. Relação de Co-autores

1- Luiz Marcelo Sá Malbouisson	
2. José Otavio Costa Auler Junior	

8. RESUMO DO CURRÍCULO

MARIA JOSÉ CARVALHO CARMONA, nascida em 19/03/61 em Santa Rita de Caldas (MG), RG 17 760 864 SSP – SP, CREMESP 54 142, CPF 059 174 158/03
Endereço: Rua Rodésia 161/82 – Vila Madalena – CEP 05435-020 – São Paulo - SP

GRADUAÇÃO

1985 Médica, Faculdade de Ciências Médicas da Universidade Estadual de Campinas (**UNICAMP**)

FORMAÇÃO ACADÊMICO-PROFISSIONAL

1986-1987 Residência Médica – Anestesiologia- Hospital das Clínicas - Faculdade de Medicina - USP
1988 Especialização em Anestesia para Cirurgia Cardiovascular e Torácica – Instituto do Coração - HCFMUSP
1995 Doutora em Medicina – Faculdade de Medicina - USP
1995 Título de Especialista em Terapia Intensiva – AMIB/AMB e CFM
2002 Professora Doutora da Faculdade de Medina da Universidade de São Paulo
2004 Livre-Docente – Faculdade de Medicina da USP
2006 Professora Associada da FMUSP – Disciplina de Anestesiologia, Departamento de Cirurgia.

FUNÇÕES ATUAIS

- Professora Associada da Disciplina de Anestesiologia, departamento de Cirurgia – FMUSP, desde 2006
- Diretora da Divisão de Anestesia do Hospital das Clínicas da Faculdade de Medicina da USP desde 2005
- Co-responsável pelo Centro de Ensino e Treinamento da Sociedade Brasileira de Anestesiologia (CET-SBA) do Hospital das Clínicas da Faculdade de Medicina da USP, desde 1996.
- Orientadora da Pós-Graduação da Disciplina de Anestesiologia da Faculdade de Medicina da USP desde 1997.
- Vice-Coordenadora do Programa de Pós-Graduação em Anestesiologia da FM-USP desde 2004.

LISTA DE PRINCIPAIS PUBLICAÇÕES:

1. Leite FD, Pereira VA, Omosako CE, **Carvalho Carmona MJ**, Costa Auler JO, Jr., Cavani Jorge Santos SR: A Micromethod for the Quantification of Atenolol in Plasma Using High-Performance Liquid Chromatography With Fluorescence Detection: Therapeutic Drug Monitoring of Two Patients With Severe Coronary Insufficiency Before Cardiac Surgery. Ther Drug Monit 2006; 28: 237-244
2. **Carmona MJ**, Malbouisson LM, Pereira VA, Bertoline MA, Omosako CE, Le Bihan KB, Auler Jr JO, Santos SR. - Cardiopulmonary bypass alters the pharmacokinetics of propranolol in patients undergoing cardiac surgery. *Braz J Med Biol Res.* 2005 May;38(5):713-21.
3. Nascimento JW, **Carmona MJ**, Strabelli TM, Auler JO Jr, Santos SR. - Systemic availability of prophylactic cefuroxime in patients submitted to coronary artery bypass grafting with cardiopulmonary bypass. *J Hosp Infect.* 2005 Apr;59(4):299-303.
4. Nozawa, E.; Kobayashi, Matsumoto, M.E.; Feltrin, M.I.Z.; **Carmona, M.J.C.**; Auler Jr., J.O.C. – Assessment of factors that influence weaning from long-term mechanical ventilation after cardiac surgery. *Arq Bras Cardiol* 2003; 80(3):306-10
5. Machado, L.B.; Chiaroni, S.; Vasconcelos F°, P.O.; Auler Jr., J.O.C.; **Carmona, M.J.C.**– Incidência de Cirurgia Cardíaca em Pacientes Octagenários. Estudo Retrospectivo. *Rev. Bras. Anestesiol* 2003, 53(5):646-653
6. Yoshinaga EM, Franceschi RC, Malbouisson LMS, Fraga AO, Auler Jr. JOC, **Carmona MJC**. Effects of xenon on hemodynamics in experimental model of hemorrhagic shock. *Eur J Anasth 2003*; 20, Suppl 30: 65.
7. **Carmona MJC**, Pereira VA, Bertolini MA, Malbouisson LMS, Le Bihan KB, Lopes MR, Auler Jr. JOC, Santos SRCJ. Atenolol and propranolol pharmacokinetics changes in patients undergoing coronary artery bypass surgery. Eur J Anasth 2003; 20, Suppl 30: 139.
8. Pereira, V.A.; **Carmona, M.J.C.;** Bertolini, M.A.; Auler Jr., J.O.C.; Santos, S.R.C.J. – Obesity influences Propranolol pharmacokinetics in patients undergoing coronary artery bypass grafting employing cardiopulmonary bypass. *Brazilian Journal of Pharmaceutical Sciences* 2003,39(4):409-414
9. Auler Jr., J.O.C.; **Carmona, M.J.C.;** Barbas, C.S.V.; Saldiva, P.H.N.; Malbouisson, L.M.S. - The Effects of Positive end-expiratory Pressure on Respiratory System Mechanics and Hemodynamics in Postoperative Cardiac Surgery Patients". *Braz. J. Med. Biol. Res* 2000, 33(1):31-42
10. Bocchi EA, Vilella de Moraes AV, Esteves-Filho A, Bacal F, Auler JO, **Carmona MJ**, Bellotti G, Ramires AF: L-arginine reduces heart rate and improves hemodynamics in severe congestive heart failure. Clin Cardiol 2000; 23: 205-10.
11. Pereira, V.A.; Auler Jr., J.O.C.; **Carmona, M.J.C.;** Mateus, F.H.; Lanchote, V.L.; Breimer, D.D.; Santos, S.R.C.J. - A Micromethod for Quantitation of Debrisoquine and 4-hydroxydebrisoquine in Urine by Liquid Chromatography. *Braz. J. Med. Biol. Res.* 2000, 33(5):509-514
12. **Carmona MJ**, Auler JO, Jr.: Effects of inhaled nitric oxide on respiratory system mechanics, hemodynamics, and gas exchange after cardiac surgery. J Cardiothorac Vasc Anesth 1998; 12: 157-61.
13. Auler Junior JO, **Carmona MJ**, Bocchi EA, Bacal F, Fiorelli AI, Stolf NA, Jatene AD: Low doses of inhaled nitric oxide in heart transplant recipients. J Heart Lung Transplant 1996; 15: 443-50.

NÚMEROS:

- **Livros publicados:** nada consta
- **Publicações em periódicos com seletiva política editorial:** 31
- **Capítulos de livro:** 18
- **Teses de mestrado orientadas e já defendidas:** 2 orientadas e já defendidas

Anexo III

HOSPITAL DAS CLÍNICAS
DA
FACULDADE DE MEDICINA DA UNIVERSIDADE DE SÃO PAULO

DIRETORIA CLÍNICA
COMISSÃO DE ÉTICA PARA ANÁLISE DE PROJETOS DE PESQUISA
CAPPesq

CADASTRO DO PESQUISADOR

---

1. Identificação e qualificação do pesquisador:

Nome: **JOSÉ OTÁVIO COSTA AULER JR.** RG: **3.952.803**

CPF: **531.866.998-00** Profissão: **Médico Anestesiologista**

Endereço: **Rua Guarará nº 538 - apartamento 51**

CEP: **01425-001** Telefone: **887-1002**

---

2. Graduado em **Medicina** Data: **1972**

Instituição **Faculdade Federal de Medicina Triângulo Mineiro - Uberaba - MG**

---

3. Titulação acadêmica mais elevada: **Professor Titular**

---

4. Vinculação Profissional Atual

X HC **X** FMUSP ☐ Outras Instituições: **InCor**

Função: **Professor Titular**

Lotação: **Disciplina de Anestesiologia da FMUSP**

---

5. Vinculação Acadêmica Atual

X Graduação **X** Pós-Graduação { ☐ Doutorado ☐ Mestrado }

X Outras - especificar: **Professor Titular**

---

6. Área do conhecimento: Medicina (Anestesiologia)

---

7. Relação de Co-autores

**Profa. Dra. Maria José Carvalho Carmona**	
**Dra. Kátia Osternack Pinto**	
**Dr.Luiz Marcelo de Sá Malbouisson**	

8. Resumo do Currículo

. Utilizar este campo abaixo para ressaltar, em síntese, os aspectos que o capacitam para a pesquisa.

. Não serão aceitos anexos.

Títulos Acadêmicos:

- **Doutor em Medicina** (1986) pela Faculdade de Medicina da Universidade de São Paulo, na área de Concentração do Departamento de Patologia, sobre o tema **Avaliação da Função Respiratória antes e depois da Cirurgia Cardíaca com Circulação Extracorpórea em Pacientes Anestesiados**, tendo sido aprovado com nota 10 (dez), com louvor.
- **Docente-Livre** (1989) da Faculdade de Medicina da Universidade de São Paulo - Departamento de Cirurgia, programa da Disciplina de Anestesiologia, com a tese **Efeitos do Volume e Fluxo Ventilatórios sobre as Propriedades Mecânicas do Sistema Respiratório**.
- **Professor Titular** da Disciplina de Anestesiologia do Departamento de Cirurgia da Faculdade de Medicina da Universidade de São Paulo, desde 1997.
- **Especialista em Anestesiologia** pela Sociedade Brasileira de Anestesiologia.Título obtido por concurso durante o Congresso Brasileiro de Anestesiologia, realizado em Belém do Pará, dezembro de 1976.
- **Especialista em Terapia Intensiva** pela Associação Médica Brasileira e pela Associação de Medicina Intensiva Brasileira, em São Paulo, outubro de 1983.

Participação em Comissões e Colegiados Institucionais:

Membro do Conselho Deliberativo do Hospital das Clínicas da FMUSP, no período de 1999 a 2001.
Chefe do Departamento de Cirurgia da FMUSP, no período de 2000 a 2001.
Membro do Conselho do Departamento de Cirurgião, desde 1991.
Membro da Congregação da FMUSP, desde 1997.
Membro da Comissão Científica do InCor, desde 2004.
Presidente da Comissão de Reestruturação Departamental e Criação de Novas Áreas de Conhecimento, criada em 09 de setembro de 2004.

Cargos Atuais:

- Responsável pelo Laboratório de Investigação Médica de Anestesiologia da FMUSP/LIM-08.
- Coordenador do Programa de Pós-Graduação Strictu Senso, na área de Anestesiologia da FMUSP.
- Responsável pelo Programa de Residência Médica em Anestesiologia do HCFMUSP.
- Diretor do Serviço de Anestesiologia e UTI Cirúrgica do InCor.

Assessoria *Ad hoc*: Assessor da FAPESP; Assessor do CNPq; Membro da Comissão de Avaliação da CAPES, área de Medicina III (triênio 2001-2003); Parecerista da Artificial Organs - EUA; Parecerista da Revista Brasileira de Anestesiologia – SBA; Parecerista da Revista Clinics; Parecerista da Revista Brasileira de Terapia Intensiva.

Linha de Pesquisa (CAPES):

Aspectos hemodinâmicos e metabólicos das técnicas e agentes anestésicos e do per-operatório de cirurgia cardiovascular e torácica.
Impactos da anestesia e da cirurgia cardiovascular e torácica sobre o sistema respiratório.
Aspectos fisiológicos da hemodinâmica e da hemodiluição aplicados a reposição volêmica.

Atividades de Formação:

Iniciação científica: 07 (sete) acadêmicos, todos com fomento, sendo 02 em andamento:
Processo FAPESP nº **04/11794-9**: "Estudo comparativo entre medidas contínuas e intermitentes de gasometria durante manobras de recrutamento em modelo experimental de lesão pulmonar aguda".
Processo FAPESP nº **04/10786-2**: "Estudo comparativo entre medidas contínuas e intermitentes do débito cardíaco durante manobras de recrutamento em modelo experimental de lesão pulmonar aguda".
Mestrado: 14 (quatorze) dissertações defendidas.
Doutorado: 16 (dezesseis) teses defendidas e 06 (seis) em andamento, sendo uma com bolsa CNPq.

Auxílios Pesquisa:

10 (dez) auxílios pesquisas outorgados pela FAPESP, totalizando aproximadamente R$ 943.000,00 (novecentos e quarenta e três mil reais)

Auxílios Pesquisa Vigentes :

Produção Científica (1999-2005): Trabalhos completos: 62 sendo: PubMed: 16/ ISI: 19 /Scielo: 27
Livros editados: 07 Capítulos de livros: 52, sendo: Nacionais: 30 / Internacional: 22
Comunicações em congressos: 104, sendo: Nacionais: 75 / Internacional: 29 / Aulas em eventos: 92 /Nacionais: 64 Internacionais: 28

Anexo III

HOSPITAL DAS CLÍNICAS
DA
FACULDADE DE MEDICINA DA UNIVERSIDADE DE SÃO PAULO
DIRETORIA CLÍNICA
COMISSÃO DE ÉTICA PARA ANÁLISE DE PROJETOS DE PESQUISA
CAPPesq

CADASTRO DO PESQUISADOR

---

1. Identificação e qualificação do pesquisador:

Nome: Kátia Osternack Pinto - RG: 13.035.630

CPF: 411.283.389-04 - Profissão: Psicóloga

Endereço: Av. Higienópolis, 147, ap. 1E.

CEP: 01238-001 - Tefefone: (11)3255-3032 e (11)9720-9789.

---

2. Graduado em Psicologia Data: 1985.

Instituição: Fac. de Filosofia, Ciências e Letras da USP, Campus de Ribeirão Preto

---

3. Titulação acadêmica mais elevada:.Doutor em Ciências pela FMUSP

---

4. Vinculação Profissional Atual

(X) HC ( ) FMUSP ( ) Outras Instituições: ....

Função: Diretor Técnico de Serviço de Saúde.
Lotação: Divisão de Psicologia do Instituto Central.

---

5. Vinculação Acadêmica Atual

( ) Graduação (X) Pós-Graduação { (X). Doutorado / ( ) Mestrado }

( ) Outras - especificar: ....

---

6. Área do conhecimento : Neuropsicologia.

---

7. Relação de Co-autores

8. Resumo do Currículo

. Utilizar este campo abaixo para ressaltar, em síntese, os aspectos que o capacitam para a pesquisa.

. Não serão aceitos anexos.

Titulação

- Doutor em Ciências: Neurologia da FMUSP. Ano: 2005. Tese: Análise comparativa das funções neuropsicológicas de portadores de doença de Parkinson em estágios inicial e avançado: uma determinação de padrões para diagnóstico em população brasileira.
- Mestre em Ciências. Fisiopatologia Experimental da FMUSP. Ano: 2001. Dissertação: Personalidade e qualidade de vida em portadores de doença inflamatória intestinal: subsídios para avaliação do paciente crônico em coloproctologia.
- Especialização em Neuropsicologia. Centro de Estudos em Psicologia da Saúde da Divisão de Psicologia do Instituto Central do HC-FMUSP. Ano: 2001. Monografia de conclusão: Avaliação neuropsicológica em portadores de Doença de Parkinson indicados para neurocirurgia: um estudo-piloto.
- Especialização em Psicologia Hospitalar em Hospital Geral. Divisão de Psicologia do Instituto Central do HC-FMUSP. Ano: 1997. Monografia de conclusão: O Método de Rorschach na avaliação neuropsicológica de quatro pacientes com acidente vascular cerebral.

Atuação profissional

- Psicóloga da Divisão de Psicologia do Instituto Central do HC-FMUSP, atuando na Clínica Neurológica como neuropsicóloga.
- Coordenadora e Docente do Curso de Especialização em Neuropsicologia do Centro de Estudos em Psicologia da Saúde da Divisão de Psicologia do Instituto Central do HC-FMUSP desde 2001, ministrando várias disciplinas.

Apresentação de trabalhos

- TERRONI, Luisa de Marillac Niro; SOBREIRO, M F M; YEH, M S L; TINONI, G; AMARO JR, Edson; CARVALHO, R C; SANTOS, M I; PINTO, Kátia Osternack; FRÁGUAS JR, Renério. Depressão pós AVC: aspectos cognitivos e tratamento. 2005. XXIII Congresso Brasileiro de Psiquiatria: diretrizes em psiquiatria. Pôster.
- PINTO, Kátia Osternack. Diagnóstico neuropsicológico no contexto interdisciplinar. 2005. II Congresso Brasileiro de Avaliação Psicológica: desafios para a formação, prática e pesquisa. Mesa Redonda.
- PINTO, Kátia Osternack. Neuropsicologia enquanto especialidade e campos de atuação. 2005. I Simpósio Interdisciplinar de Neurociências do Triângulo Mineiro. Conferência.
- PINTO, Kátia Osternack. Perdas cognitivas associadas à depressão: como investigar?. 2004. Simpósio; Tratando a depressão: mais do que antidepressivo e psicoterapia. Mesa Redonda.
- PINTO, Kátia Osternack. Neurociência e Neuropsicologia: perspectivas atuais e campos de atuação. 2004. Ciclo de Palestras IAP - Instituto de Avaliação Psicológica e Pesquisa. Palestra.

/tsc.

Anexo III

HOSPITAL DAS CLÍNICAS
DA
FACULDADE DE MEDICINA DA UNIVERSIDADE DE SÃO PAULO

DIRETORIA CLÍNICA
COMISSÃO DE ÉTICA PARA ANÁLISE DE PROJETOS DE PESQUISA
CAPPesq

CADASTRO DO PESQUISADOR

---

1. Identificação e qualificação do pesquisador:

Nome: Luiz Marcelo Sá Malbouisson.... RG: .04355961-10 (SSP/BA)...

CPF: 645.330.505-49 Profissão: médico...

Endereço: Rua Pascoal Vita, nº 366, ap 74, Vila Beatriz, SP, SP..

CEP: 05445 -000...................................... Telefone: 3031 6958

---

2. Graduado em Medicina Data: ..3/12/1994

Instituição Escola de Medicina e Saúde Pública –Salvador -Bahia

---

3. Titulação acadêmica mais elevada: .Résidencia médica em anestesiologia, Titulo superior em anestesiologia, Doutor em ciências, Título de Especialista em Terapia Intensiva

---

4. Vinculação Profissional Atual

X HC ☐ FMUSP **X** Outras Instituições: FFM

Função: Médico Supervisor...

Lotação: Serviço de Anestesiologia.e Terapia.Intensiva cirurgica, Incor, HCFMUSP

---

5. Vinculação Acadêmica Atual

☐ Graduação **X** Pós-Graduação { **X**.Doutorado: ☐ Mestrado }

☐ Outras - especificar: .

---

6. Área do conhecimento : Anestesiologia e Terapia intensiva ....

---

7. Relação de Co-autores

Silvia Daniela Scarpel de Oliveira	
Rodrigo Sanchez Veliz	
Ana Carla Giosa Fujita	

8. Resumo do Currículo

. Utilizar este campo abaixo para ressaltar, em síntese, os aspectos que o capacitam para a pesquisa.

. Não serão aceitos anexos.

Iniciação científica em neurofisiologia experimental na UFBA (1990 – 1994)
Bolsista de iniciação cientifica – CNPQ (1990-1994)
Residência médica em anestesiologia no HCFMUSP com especialização em anestesia para cirurgia cardiáca e terapia intensiva pós-operatória (1995 – 1998)
Estagio como médico pesquisador no Serviço de Anestesia do InCor (1998)
Estagio de pós-graduação em pesquisa clínica na área de terapia intensiva sob orientação do Pr. J-J. Rouby,H Pitié-Salpetrière, Paris, França com duração de 18 meses (1998-2000)

Artigos:
1) Acute effect of intracerebroventricular administration of zinc in the drinking behavior of rats induced by dehydration or central cholinergic and angiotensinergic stimulation. *Brazillian Journal of Mededical and Biological Research.* 27:2653-2633, 1994
2) Nutrição Parenteral em Cardiologia. *Revista da Sociedade de Cardiologia do Estado de São Paulo.* 7:529-536, 1997
3) Thermal filament continuous thermodilution cardiac output delayed response limits its value during acute hemodynamic instability. *J Trauma.* 47: 288-93, 1999
[illegible] The effects of positive end-expiratory pressure on respiratory system mechanics and hemodynamics in [illegible]stoperative cardiac surgery patients. *Braz J Med Biol Res.* 33: 31-42, 2000.
5) Role of heart in loss of aeration characterizing the lower lobes in acute respiratory distress syndrome. American Journal of Respiratory and Critical Care Medicine. In printing.
6)Validation of a software designed for computed tomographic (CT) measurement of lung water. *Intensive Care Med.* 27: 602-8, 2001.
7) Computed Tomography Assessment of Positive End-expiratory Pressure-induced Alveolar Recruitment in Patients with Acute Respiratory Distress Syndrome. Am. *J. Respir. Crit. Care Med*; 163: 1444-1450, 2001.
8) Assessment of PEEP-induced reopening of collapsed lung regions in acute lung injury: are one or three CT sections representative of the entire lung? *Intensive Care Med* 27(9):1504-10, 2001.

Resumos apresentados em congresso:
1) **Malbouisson LMS**, Mourgeon E, Lu Q, Puybasset L, Coriat P, Rouby JJ. "Role of heart in passive collapse of lower lobes in acute respiratory distress syndrome". 7th Congress of the European Society of Anesthesiologists, Amsterdam, Holanda, 05/1999
2) Puybasset L, Muller JC, Lu Q, **Malbouisson LMS**, Coriat P, Rouby JJ. "Distribution régionale de la réduction de CRF et de l'excès de tissu pulmonaire dans le SDRA". Congres de la Société Française d'Anesthésie et Réanimation, Paris, França, 09/1999
3) **Malbouisson LMS**, Figueiredo LP, Carmona MJC, Auler Jr. JOC. "Comparação entre métodos de avaliação do débito cardíaco durante choque hipovolêmico experimental." 45ºCongresso Brasileiro de Anestesiologia, Fortaleza, CE, 10/1998
4) **Malbouisson LMS**, Messias ERR, Puttini CM, Auler Jr. JOC. "Suporte Inotrópico após a circulação extracórporea em [illegible]evascularização do miocárdio" 45ºCongresso Brasileiro de Anestesiologia, Fortaleza, CE, 10/1998
[illegible]) **Malbouisson LMS**, Almeida DJNC, Gomes DL, Auler Jr JOC. "Anestesia Peridural torácica alta combinada com anestesia geral para revascularização miocárdica minimamente invasiva. Relato de caso. 45ºCongresso Brasileiro de Anestesiologia, Fortaleza, CE, 10/1998
6) Beck K, **Malbouisson LMS**, Hueb AC, Carmona MJC, Auler Jr. JOC. "Tempo para extubação no pós operatório de cirurgia cardíaca: Correlação entre tempo para extubação e duração de circulação extracorpórea, hidratação per-operatória e relação PaO_2/FiO_2." 5º Congresso Paulista de Terapia Intensiva, São Paulo, SP, 10/1998
7) Fregonese JB, Ferreira H, , Cunha M, Souza CB, Barros L, **Malbouisson LM,** De Oliveira IR, De Castro e Silva E. "Administração central de chumbo inibe a ingestão de água induzida pela angiotensina II." IX Reunião Anual da Federação de Sociedades de Biologia Experimental, Caxambu, MG, 08/1994
8) De Castro e Silva E, **Malbouisson LM,** Chaves A, Barros L, Fregonese JB, Antunes rodrigues J. "Efeito da administraçãocentral de um antagonista β_2 adrenérgico (IPS 339) sobre a resposta hiperglicemica ao estresse em ratos." VIII Reunião Anual da Federação de Sociedades de Biologia Experimental, Caxambu, MG, 10/1993
9) **Malbouisson LM**, De castro e Silva E, Chaves A, Saraiva A, Barros L, Antunes Rodrigues J. "Efeito da administração de fluoxetina sobre a hiperglicemia induzida por estresse em ratos." VIII Reunião Anual da Federação de Sociedades de Biologia Experimental, Caxambu, MG, 08/1993
10) **Malbouisson LM**, Fregonese JB, De Castro e Silva E, Chaves A, Barros L, Antunes Rodrigues J. "Efeito da fluoxetina sobre a glicemia basal em ratos." VIII Reunião Anual da Federação de Sociedades de Biologia Experimental, Caxambu, MG, 08/1993
Proficiência na Língua Francesa, Aliança Francesa, São Paulo, 1998
Proficiência na Língua Inglesa, Cultura Inglesa, Salvador, 1993-Associação Alumni, São Paulo, 1998

DISCIPLINA DE ANESTESIOLOGIA – FMUSP
INSTITUTO CENTRAL DO HCFMUSP

Protocolo de Pesquisa

DISFUNÇÃO COGNITIVA APÓS CIRURGIA SOB ANESTESIA GERAL

Pesquisadores:
Profa. Dra. Maria José Carvalho Carmona
Dra. Kátia Osternack Pinto
Dr. Luiz Marcelo Sá Malbouisson
Prof. Dr. José Otávio Costa Auler Jr.

2006

DISFUNÇÃO COGNITIVA APÓS CIRURGIA SOB ANESTESIA GERAL

RESUMO

Alguns dos determinantes de disfunção cognitiva pós-operatória são o uso de opióides no período pós-operatório, falta de atividade física, fadiga pós-operatória, a dor pós-operatória, a qualidade de vida, além de possível predisposição genética. O uso de corticóide pré-operatório diminui a dor pós-operatória, diminuindo também a necessidade de opióides, além de diminuir a fadiga, a incidência de náusea e vômito, o período de recuperação e o tempo de retorno às atividades diárias no período pós-operatório, sendo freqüentemente utilizado como coadjuvante em anestesia. Os objetivos deste estudo são: 1) determinar a incidência de disfunção cognitiva no pós-operatório comparando dexametasona *versus* placebo em 150 pacientes submetidos à cirurgia sob anestesia geral; 2) Investigar a associação entre alterações cognitivas no pós-operatório e a presença do alelo ε4 do gene da apolipoproteína E e de marcadores bioquímicos séricos para lesão neuronal, como a proteína S100β e a enolase específica do neurônio (NSE). Serão aplicados testes neuropsicológicos para avaliar o estado mental, raciocínio e conceituação, atenção e concentração, fluência verbal, percepção visuoespacial, memória imediata, operacional e de evocação e habilidades executivas, incluindo velocidade de processamento. Esta bateria define o índice cognitivo da estabilidade (medida longitudinal do status cognitivo em avaliações repetidas que detectam mudanças estatísticamente significativas no funcionamento do sistema nervoso central). Esta avaliação será realizada pessoalmente antes da cirurgia e na noite após a cirurgia e por via telefônica nos dias pós-operatórios 3, 7, 21, e 90. A anestesia geral seguirá os padrões gerais incluindo Propofol (2-3 mg/kg), rocuronio (0.5 mg/kg), e fentanil (1-2 µg/kg) para indução. A anestesia subseqüente e mantida com sevoflurano (1.0-2.5%) e N_2O em 40% de oxigênio, rocuronio (doses suplementares serão administradas se necessário para manutenção de anestesia cirúrgica), e fentanil (doses suplementares 0.5-10 µg/kg com dose máxima de 250 µg). A administração de sevoflurano será ajustada com o objetivo de manter os níveis de hipnose avaliada pelo índice bispectral (BIS) de acordo com os grupos do estudo: os participantes serão randomizados em 2 grupos: anestesia superficial (índice bispectral entre 45 e 55) ~~and~~ e anestesia profunda (índice bispectral entre 35 e 45). Ambos níveis são aceitáveis para anestesia geral e são atualmente usados por anestesiologistas. Após venóclise periférica, antes da indução anestésica, será colhida uma amostra de sangue para determinação do perfil genotípico da Apoε e dosagem de S100β e NSE. Os resultados dos escores dos testes em cada grupo serão comparados através do teste de Friedmann, e entre os grupos será utilizado o teste de Kruskal Wallis. Porcentagens serão comparadas pelo do teste de Qui-Quadrado Os dados serão expressos como mediana e intervalo de confiança de 95%. Serão considerados significativos resultados cujos valor p sejam menores que 0,05 nos testes estatísticos.

DISFUNÇÃO COGNITIVA APÓS CIRURGIA SOB ANESTESIA GERAL

1 – INTRODUÇÃO

Disfunção cognitiva no pós-operatório imediato de cirurgias sob anestesia geral é um evento relativamente freqüente, especialmente em pacientes geriátricos. Na maioria dos procedimentos é reversível, podendo ser permanente em menos de 1% dos casos. Procedimentos sob anestesia regional também podem causar, com menor freqüência, disfunção cognitiva transitória no pós-operatório imediato.

O uso de corticóide pré-operatório diminui a dor pós-operatória, diminuindo também a necessidade de opióides. Diminui fadiga, náusea e vômito, período de recuperação e tempo de retorno às atividades diárias no período pós-operatório. Todos estes fatores melhoram qualidade de vida. Estudos mostram que alguns dos determinantes de disfunção cognitiva pós-operatória são o uso de opióides no período pós-operatório, falta de atividade física, fadiga pós-operatória, dor pós-operatória e qualidade de vida.

Se o corticóide modifica positivamente fatores que estão ligados a disfunção cognitiva como dor, uso de opióides, fadiga, recuperação pós-operatória, convalescença, qualidade de vida e mais rápido retorno às atividades físicas, ele também modificaria positivamente a função cognitiva no período pós-operatório.

2 – OBJETIVOS

Os objetivos deste estudo são:

1) determinar a incidência de disfunção cognitiva no pós-operatório comparando dexametasona *versus* placebo em 150 pacientes submetidos à cirurgia sob anestesia geral;
2) Investigar a associação entre alterações cognitivas no pós-operatório e a presença do alelo ε4 do gene da apolipoproteína E e de marcadores bioquímicos séricos para lesão neuronal, como a proteína S100β e a enolase específica do neurônio (NSE).

3 – CASUÍSTICA E MÉTODOS

Após aprovação pela CAPPesq e obtenção de auxílio financeiro para o estudo, serão estudados 150 pacientes idosos de ambos os sexos submetidos a anestesia geral para prodedimentos cirúrgicos

Serão aplicados testes neuropsicológicos para avaliar o estado mental, raciocínio e conceituação, atenção e concentração, fluência verbal, percepção visuoespacial, memória imediata,

operacional e de evocação e habilidades executivas, incluindo velocidade de processamento. Esta bateria define o índice cognitivo da estabilidade (medida longitudinal do status cognitivo em avaliações repetidas que detectam mudanças estatísticamente significativas no funcionamento do sistema nervoso central).

Esta avaliação será realizada pessoalmente antes da cirurgia e na noite após a cirurgia e por via telefônica nos dias pós-operatórios 3, 7, 21, e 90.

Critérios de inclusão: paciente acima de 60 anos, submetido videocirurgia para herniorrafia inguinal, operação de Nissen ou colecistectomia sob anestesia geral com permanência hospitalar de até 24 horas.

Critérios de exclusão: idade inferior a 60 anos, história pregressa de doença cerebral ou demência, outras doenças psiquiátricas que afetem a cognição, falta de domínio da língua portuguesa, uso de corticóide ou de opióde.

Os pacientes que preencherem os critérios para participarem do estudo e aceitarem participar do estudo serão contatados por telefone e será aplicada a bateria de testes. Após os testes o paciente estará liberado ou não para fazer parte do estudo.

No dia da cirurgia, pacientes devem assinar um termo de consentimento em duas vias. Uma cópia ficará com o paciente e outra com o médico.

Após obter o consentimento, médico ira aplicar a droga designada para o paciente duas horas antes da indução da anestesia. Essa droga será determinada aleatoriamente sem o conhecimento do médico, podendo ser: dexametasona, 8 mg IV ou placebo (soro fisiologico), no mesmo volume.

Após venóclise periférica, antes da indução anestésica, serão colhidas duas amostras de sangue de para determinação do perfil genotípico da Apoε e dosagem de S100β e NSE. As amostras serão processadas no Laboratório de Biologia Molecular do InCor (APOε4) e Laboratório de Neurociências da UFRGS (S100β e NSE).

A anestesia geral será induzida com utilização dos seguintes fármacos: Propofol (2-3 mg/kg), rocuronio (0.5 mg/kg), e fentanil (1-2 μg/kg) para indução. A anestesia subseqüente será mantida com sevoflurano (1.0-2.5%) e N_20 em 40% de oxigênio, rocuronio (doses suplementares serão administradas se necessário para manutenção de anestesia cirúrgica), e fentanil (doses suplementares 0.5-10 μg/kg com dose máxima de 250 μg). Variação em requerimentos individuais preclude administração de doses idênticas para cada paciente. Ao invés, a administração de sevoflurano deverá ser ajustada pelo anestesiologista com o objetivo de manter of níveis do BIS (hipnose) de acordo com os grupos do estudo, e fentanil será administrado com o objetivo de manter a pressão arterial e freqüência cardíaca (analgesia) dentro de 20% dos valores pré-indução. A ventilação será controlada mecanicamente para manter a pressão de dióxido de carbono próximo a 35 mmHg. Fentanil (1-2 μg/kg) será dado ao final da

cirurgia para melhorar a analgesia durante a recuperação anestésica. Soro fisiológico 5-10 ml/kg/hora será dado durante a cirurgia.

O índice bispectral mede a atividade cerebral cortical (hipnose) numa escala de 0 a 100, onde 100 representa o estado "acordado" e 0 representa um eletroencefalograma isoelétrico. Medidas abaixo de 70 estão relacionadas a inconsciência e ausência de memória necessária para a anestesia geral. Neste estudo, os participantes serão randomizados em 2 grupos: anestesia superficial (índice bispectral entre 45 e 55) and anestesia profunda (índice bispectral entre 35 e 45). Ambos níveis são aceitáveis para anestesia geral e são atualmente usados por anestesiologistas.

A pele da região onde o monitor vai ser aplicado deve estar limpa e seca. Use álcool e seque esta área da pele.

Aplique o sensor na testa do paciente. Coloque o circulo numero 1 no centro aproximadamente 2 cm acima do ponte nasal. Coloque o círculo número 3 na area temporal.

Importante: Aplique pressão em torno do sensor (incluindo as áreas entre os círculos) para garantir a adesão adequada. Em seguida, pressione os círculos 1, 2 e 3 por 5 segundos para assegurar o contato dos eletrodos a pele.

Conecte o sensor aplicado na testa do participante no cabo do índice bispectral.

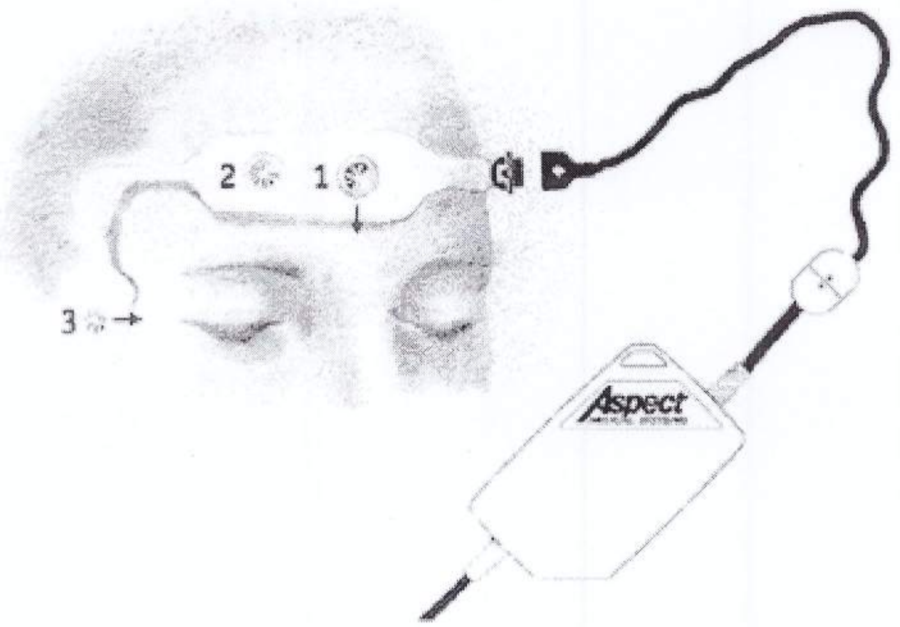

A avaliação da disfunção cognitiva nos dois grupos será realizada atráves de testes neuropsicológicos, padronizados especificamente para avaliar as funções cognitivas superiores, e questionários para avaliar as características sócio-demográficas, questionamentos sobre a percepção pessoal da saúde do paciente (nos anexos). Estes testes serão aplicados no pré-operatório, na noite

anterior à cirurgia, antes da administração de medicações pré-anestésicas ou sedativas, no 3º, 7º, 21º e 90º dia pós-operatório.

Os dados serão analisados utilizando-se o software de análise estatistica SPSS versão 10. Os dados demograficos como idade, peso, altura serão comparados nos quatro grupos através de análise de variância univariada seguida de teste de comparações múltiplas se aplicável. As variáveis colhidas através de testes neuropsicologicos aplicados antes e depois do procedimento cirurgico são de natureza qualitativa ou ordinal, sendo atribuídos resultados númericos e os resultados finais dos testes serão expressos em escores. Os resultados dos escores dos testes em cada grupo serão comparados através do teste de Friedmann, e entre os grupos será utilizado o teste de Kruskal Wallis. Porcentagens serão comparadas atráves do teste de Qui-Quadrado Os dados serão expressos como mediana e intervalo de confiança de 95%. Serão considerados significativos resultados cujos valor p sejam menores que 0,05 nos testes estatísticos.

INSTRUMENTOS

Informações Sócio-demográficas

Critérios para inclusão no estudo:

- Pacientes acima de 60 anos
- Submetido à cirurgia de pequeno porte
- Sob anestesia geral

Criterios para exclusão:

1. Idade inferior a 60 anos
2. História pregressa de doença cerebral ou demência
3. Outras doenças psiquiátricas que afetem a cognição
4. Falta de domínio da língua portuguesa
5. Estar fazendo uso de corticoíde

- Nome: ____
- Diagnóstico: ____
- Sexo: masculino feminino
- Idade (anos): __________
- Peso (quilos): __________
- Altura (centímetros): __________

Estado civil

( ) solteiro (a)
( ) casado(a)
( ) divorciado(a)
( ) viúvo(a)

Emprego

( ) empregado(a)
( ) desempregado(a)
( ) aposentado(a)

Salário mensal:

( ) 0
( ) 0-1 salário mínimo
( )1-5 salários mínimos
( )6-10 salários mínimos
( )10-15 salários mínimos
( )15-20 salários mínimos

( ) mais que 20 salários mínimos

Escolaridade

( ) Nenhuma
( ) Primário incompleto
( ) Primário completo
( ) Ginásio completo
( ) Colegial compleo
( ) Superior completo
(..) Pós-graduação

Versão Brasileira do Questionário de Qualidade de Vida SF-36

Instruções: Esta pesquisa questiona você sobre sua saúde. Estas informações nos manterão informados de como você se sente quão bem você é capaz de fazer suas atividades de vida diária. Responda cada questão marcando a resposta como indicado. Caso você esteja inseguro ou em dúvida em como responder, por favor tente responder o melhor que puder.

1. Em geral, você diria que a sua saúde é:

(circule uma)

Excelente	Muito boa	Boa	Ruim	Muito ruim
1	2	3	4	5

2. Comparada a um ano atrás, como você classificaria a sua saúde em geral, agora?

(circule uma)

Muito melhor	Um pouco melhor	Quase a mesma	Um pouco pior	Muito pior
1	2	3	4	5

3. Os seguintes items são sobre atividades que você poderia fazer atualmente durante um dia comum. Devido a sua saúde, você teria dificuldade para fazer essas atividades? Neste caso, quanto?

(circule um número em cada linha)

Atividades	Sim. Dificulta muito	Sim. Dificulta um pouco	Não. Não dificulta de modo algum
a. Atividades vigorosas, que exigem muito esforço, tais como correr, levantar objetos pesados, participar em esportes árduos	1	2	3
b. Atividades moderadas, tais como mover uma mesa, passar aspirador de pó, jogar bola, varrer a casa	1	2	3
c. Levantar ou carregar mantimentos	1	2	3
d. Subir vários lances de escada	1	2	3
e. Subir um lance de escadas	1	2	3
f. Curvar-se, ajoelhar-se ou dobrar-se	1	2	3
g. Andar mais de 1 quilômetro	1	2	3
h. Andar vários quarteirões	1	2	3
i. Andar um quarteirão	1	2	3
j. Tomar banho ou vestir-se	1	2	3

4. Durante as últimas 4 semanas, você teve algum dos seguintes problemas com o seu trabalho ou com alguma atividade diária regular, como conseqüência de sua saúde física?

(circule uma em cada linha)

	Sim	Não
a. Você diminuiu a quantidade de tempo que dedicava-se ao seu trabalho ou a outras atividades?	1	2
b. Realizou menos tarefas do que você gostaria?	1	2
c. Esteve limitado no seu tipo de trabalho ou em outras atividades?	1	2
d. Teve dificuldade de fazer seu trabalho ou outras atividades (p.ex.: necessitou de um esforço extra)?	1	2

5. Durante as últimas 4 semanas, você teve algums dos seguintes problemas com o seu trabalho ou outra atividade regular diária, como conseqüência de algum problema emocional (como sentir-se deprimido ou ansioso)?

(circule uma em cada linha)

	Sim	Não
a. Você diminuiu a quantidade de tempo que dedicava-se ao seu trabalho ou a outras atividades?	1	2
b. Realizou menos tarefas do que você gostaria?	1	2
c. Não trabalhou ou não fez qualquer das atividades com tanto cuidado como geralmente faz?	1	2

6. Durante as últimas 4 semanas, de que maneira sua saúde física ou problemas emocionais interferiram nas suas atividades sociais normais, em relação a família, vizinhos, amigos ou em grupo?

(circule uma)

De forma nenhuma	Ligeiramente	Moderadamente	Bastante	Extremamente
1	2	3	4	5

7. Quanta dor no corpo você teve durante as últimas 4 semanas?

(circule uma)

Nenhuma	Muito leve	Leve	Moderada	Grave	Muito grave
1	2	3	4	5	6

8. Durante as últimas 4 semanas, quanto a dor interferiu com o seu trabalho normal (incluindo tanto o trabalho fora de casa e dentro de casa)?

(circule uma)

De maneira alguma	Um pouco	Moderadamente	Bastante	Extremamente
1	2	3	4	5

9. Estas questões são sobre como você se sente e como tudo tem acontecido com você durante as últimas 4 semanas. Para cada questão, por favor dê uma resposta que mais se aproxime da maneira como você se sente. Em relação as últimas 4 semanas.

(circule um número para cada linha)

	Todo tempo	A maior parte do tempo	Uma boa parte do tempo	Alguma parte do tempo	Uma pequena parte do tempo	Nunca
a. Quanto tempo você tem se sentido cheio de vigor, cheio de vontade, cheio de força?	1	2	3	4	5	6
b. Quanto tempo você tem se sentido uma pessoa muito nervosa?	1	2	3	4	5	6
c. Quanto tempo você tem se sentido tão deprimido que nada pode animá-lo?	1	2	3	4	5	6
d. Quanto tempo você tem se sentido calmo e tranqüilo?	1	2	3	4	5	6
e. Quanto tempo você tem se sentido com muita energia?	1	2	3	4	5	6
f. Quanto tempo você tem se sentido desanimado e abatido?	1	2	3	4	5	6
g. Quanto tempo você tem se sentido esgotado?	1	2	3	4	5	6
h. Quanto tempo você tem se sentido uma pessoa feliz?	1	2	3	4	5	6
i. Quanto tempo você tem se sentido cansado?	1	2	3	4	5	6

10. Durante as últimas 4 semanas, quanto do seu tempo a sua saúde física ou problemas emocionais interferiram com as suas atividades sociais (como visitar amigos, parentes, etc.)?

(circule uma)

Todo o temp0	A maior parte do tempo	Alguma parte do tempo	Uma pequena parte do tempo	Nenhuma parte do tempo
1	2	3	4	5

11. O quanto verdadeiro ou falso é cada uma das afirmações para você?
(circule uma)

	Definitiva-mente verdadeiro	A maioria das vezes verdadeiro	Não sei	A maioria das vezes falsa	Definitiva-mente falsa
a. Eu costumo adoecer um pouco mais facilmente que as outras pessoas	1	2	3	4	5
b. Eu sou tão saudável quanto qualquer pessoa que eu conheço	1	2	3	4	5
c. Eu acho que a minha saúde vai piorar	1	2	3	4	5
d. Minha saúde é excelente	1	2	3	4	5

MINI EXAME DO ESTADO MENTAL - MEEM

Questões Pontos

1. Qual é: Ano? Estação (Metade do ano)? Data? Dia? Mês? 5
2. Onde estamos: Estado? País? Cidade? Bairro ou hospital? Andar? 5
3. Nomeie três objetos (carro, vaso, janela) levando 1 segundo para cada. Depois, peça ao paciente que os repita para você. Repita as respostas até o indivíduo aprender as 3 palavras (5 tentativas). 3
4. 7s seriados: Subtraia 7 de 100. Subtraia 7 desse número, etc. 5

Interrompa após 5 respostas. Alternativa: Soletre "MUNDO" de trás para frente. 5.

5. Peça ao paciente que nomeie os 3 objetos aprendidos em 3. 3
6. Mostre uma caneta e um relógio. Peça ao paciente que os nomeie conforme você os mostra. 2
7. Peça ao paciente que repita "nem aqui, nem ali, nem lá". 1

8 Peça ao paciente que obedeça sua instrução: "Pegue o papel com sua mão direita. Dobre-o ao meio com as duas mãos. Coloque o papel no chão". 3

9. Peça ao paciente para ler e obedecer o seguinte: "Feche os olhos". 1
10. Peça ao paciente que escreva uma frase de sua escolha. 1
11. Peça ao paciente que copie o seguinte desenho: 1

Escore total: (máximo de 30) ______

Fonte: Folstein et al., 1975; Bertolucci et al., 1994

Este teste permite realizar um screening do funcionamento cognitivo, excluindo possível processo demencial.

<u>TESTES NEUROPSICOLÓGICOS ESPECÍFICOS</u>

1- Capacidade de raciocínio e conceituação

Teste: subteste Semelhanças da Escala Wechsler de Inteligência para Adultos, terceira revisão - WAIS-III

Solicita o estabelecimento de associações categoriais entre estímulos concretos e abstratos em grau crescente de complexidade.

Padronizado para a população brasileira.

Considera-se como ponto de corte a nota ponderada inferior a 8.

2- Atenção Seletiva

Teste: Stroop Word Color Test

Consiste em três cartões.

O primeiro cartão é composto de retângulos coloridos (verde, rosa, azul, marrom) para simples nomeação, de modo a certificar-se da capacidade de discriminação e reconhecimento das cores e de estabelecer a velocidade base do indivíduo. O segundo cartão consiste em palavras coloridas, (cada, nunca, tudo, hoje) e o terceiro apresenta palavras coloridas com nomes de cores diferentes (marrom, azul, rosa, verde), impressas obedecendo as mesmas cores do primeiro cartão. Solicita-se ao sujeito que nomeie as cores das palavras e não faça a leitura das mesmas, de modo a determinar a capacidade de resistência à interferência do estímulo-palavra, de resposta automática, ao estímulo-cor, solicitado pela instrução.

Anota-se o tempo despedido e o número de erros em cada cartão através do uso de cronômetro.

3- Atenção Sustentada e Velocidade de Processamento

Teste: Symbol Digit Test

Apresentado nas versões escrita e oral, solicita que sejam pareados símbolos a números, através de cópia, de forma randômica, durante dois minutos.

Permite investigar a atenção sustentada, ou concentração, bem como a velocidade de processamento da informação.

Anota-se o número de símbolos completados desconsiderando-se os erros e omissões.

4- Fluência Verbal e Função Executiva

Testes: Controled Oral Word Association - FAS/COWA e Categorias Animais

Solicita-se ao sujeito que forneça palavras que iniciam com determinada letra, excetuando-se nomes próprios e sufixos e nomes de animais durante um minuto.
Avalia a capacidade de gerar palavras a partir da disponibilidade de informação fonológica (FAS) ou semântica (Animais) do indivíduo.
Permite investigar o funcionamento executivo através da presença de repetições ou inclusão de nomes que não pertençam à categoria solicitada, bem como a organização e flexibilidade mental através do modo como o sujeito agrupa as palavras e esgota as categorias escolhidas (clusters) antes de passar à outra.
A padronização é fornecida de acordo com o nível de instrução.

5- Percepção Visuoespacial

Teste: Hooper VOT - Visual Organization Test

Constitui-se em um bloco com 30 estímulos compostos de figuras separadas em partes, como peças de um quebra-cabeças, para os quais solicita-se o reconhecimento.
Investiga a capacidade de organização visuoespacial envolvendo a habilidade de análise e síntese visual.

6- Memória Imediata e Operacional

Teste: subteste Dígitos da Escala Wechsler de Inteligência para Adultos, terceira revisão - WAIS-III

É apresentado em duas partes. A primeira constitui-se na repetição de uma série de dígitos na mesma ordem apresentada e a segunda em ordem inversa. Obedecem a séries em ordem crescente permitindo investigar o span atencional e operacional do indivíduo.
Pontua-se de acordo com norma brasileira considerando-se como ponto de corte a nota ponderada inferior a 8.

7- Memória - codificação e evocação

Teste: RAVLT - Rey Auditory Verbal Learning Test

Consiste em quatro partes. Na primeira, é fornecida uma lista de 15 palavras solicitando-se a repetição imediata durante cinco apresentações. Na segunda solicita-se a evocação da mesma lista de palavras após leitura e repetição de uma segunda lista utilizada como elemento distrativo. A terceira etapa consiste na evocação da primeira lista após 20 minutos e, finalmente, a última etapa se dá através do reconhecimento da lista através da apresentação de palavras sob a forma de múltipla escolha.

Este teste permite investigar a relação temporal no processo de aquisição, retenção, recordação espontânea ou a partir do fornecimento de pistas, de modo a discriminar entre falhas nos processos de codificação, envolvendo formação hipocampal e/ou evocação, envolvendo estruturas pré-frontais executivas.

8- REFERÊNCIAS BIBLIOGRÁFICAS

1. Xie Z, Tanzi RE. Alzheimer's disease and post-operative cognitive dysfunction. Exp Gerontol 2006; 41:346-359.

3. Rohan D, Buggy DJ, Crowley S, et al. Increased incidence of postoperative cognitive dysfunction 24 hr after minor surgery in the elderly. Can J Anaesth 2005; 52:137-42.
4. Rodriguez RA, Tellier A, Grabowski J, et al. Cognitive dysfunction after total knee arthroplasty: effects of intraoperative cerebral embolization and postoperative complications. J Arthroplasty 2005; 20:763-71.
5. Rasmussen LS, O'Brien JT, Silverstein JH, et al. Is peri-operative cortisol secretion related to post-operative cognitive dysfunction? Acta Anaesthesiol Scand 2005; 49:1225-31.
6. Pratico C, Quattrone D, Lucanto T, et al. Drugs of anesthesia acting on central cholinergic system may cause post-operative cognitive dysfunction and delirium. Med Hypotheses 2005; 65:972-82.
7. Cohendy R, Brougere A, Cuvillon P. Anaesthesia in the older patient. Curr Opin Clin Nutr Metab Care 2005; 8:17-21.
8. Benoit AG, Campbell BI, Tanner JR, et al. Risk factors and prevalence of perioperative cognitive dysfunction in abdominal aneurysm patients. J Vasc Surg 2005; 42:884-90.
9. Arain SR, Barth CD, Shankar H, Ebert TJ. Choice of volatile anesthetic for the morbidly obese patient: sevoflurane or desflurane. J Clin Anesth 2005; 17:413-9.
10. Iohom G, Szarvas S, Larney V, et al. Perioperative plasma concentrations of stable nitric oxide products are predictive of cognitive dysfunction after laparoscopic cholecystectomy. Anesth Analg 2004; 99:1245-52, table of contents.
11. Rasmussen LS, Johnson T, Kuipers HM, et al. Does anaesthesia cause postoperative cognitive dysfunction? A randomised study of regional versus general anaesthesia in 438 elderly patients. Acta Anaesthesiol Scand 2003; 47:260-6.
12. Canet J, Raeder J, Rasmussen LS, et al. Cognitive dysfunction after minor surgery in the elderly. Acta Anaesthesiol Scand 2003; 47:1204-10.
13. Bekker AY, Weeks EJ. Cognitive function after anaesthesia in the elderly. Best Pract Res Clin Anaesthesiol 2003; 17:259-72.
14. Heyer EJ, Sharma R, Rampersad A, et al. A controlled prospective study of neuropsychological dysfunction following carotid endarterectomy. Arch Neurol 2002; 59:217-22.
15. Ancelin ML, de Roquefeuil G, Ledesert B, Bonnel F, Cheminal JC, Ritchie K. Exposure to anaesthetic agents, cognitive functioning and depressive symptomatology in the elderly. Br J Psychiatry 2001; 178:360-6.
16. Rasmussen LS, Moller JT. Central nervous system dysfunction after anesthesia in the geriatric patient. Anesthesiol Clin North America 2000; 18:59-70, vi.

17. Rasmussen LS, Steentoft A, Rasmussen H, Kristensen PA, Moller JT. Benzodiazepines and postoperative cognitive dysfunction in the elderly. ISPOCD Group. International Study of Postoperative Cognitive Dysfunction. Br J Anaesth 1999; 83:585-9.

18. Ritchie K, Polge C, de Roquefeuil G, Djakovic M, Ledesert B. Impact of anesthesia on the cognitive functioning of the elderly. Int Psychogeriatr 1997; 9:309-26.

19. Williams-Russo P, Sharrock NE, Mattis S, Szatrowski TP, Charlson ME. Cognitive effects after epidural vs general anesthesia in older adults. A randomized trial. Jama 1995; 274:44-50.

20. Moller JT, Svennild I, Johannessen NW, et al. Perioperative monitoring with pulse oximetry and late postoperative cognitive dysfunction. Br J Anaesth 1993; 71:340-7.

21. Campbell DN, Lim M, Muir MK, et al. A prospective randomised study of local versus general anaesthesia for cataract surgery. Anaesthesia 1993; 48:422-8.

HOSPITAL DAS CLÍNICAS
DA
FACULDADE DE MEDICINA DA UNIVERSIDADE DE SÃO PAULO

TERMO DE CONSENTIMENTO LIVRE E ESCLARECIDO

(Instruções para preenchimento no verso)

I - DADOS DE IDENTIFICAÇÃO DO SUJEITO DA PESQUISA OU RESPONSÁVEL LEGAL

1. NOME DO PACIENTE: ....
DOCUMENTO DE IDENTIDADE Nº: ....SEXO: M Ž F Ž
DATA NASCIMENTO: ....../....../......
ENDEREÇO:... Nº .... APTO: ....
BAIRRO: ....CIDADE ....
CEP:.. TELEFONE: DDD (............) ....

2.RESPONSÁVEL LEGAL ....
NATUREZA (grau de parentesco, tutor, curador etc.) ....
DOCUMENTO DE IDENTIDADE :..SEXO: M F
DATA NASCIMENTO: ....../......./......
ENDEREÇO: .... Nº .... APTO: ....
BAIRRO:.. CIDADE: ....
CEP: .... TELEFONE: DDD (...........)..

II - DADOS SOBRE A PESQUISA CIENTÍFICA

1. TÍTULO DO PROTOCOLO DE PESQUISA

DISFUNÇÃO COGNITIVA APÓS CIRURGIA SOB ANESTESIA GERAL

PESQUISADOR: Maria José Carvalho Carmona.
CARGO/FUNÇÃO: Professora Associada.... INSCRIÇÃO CONSELHO REGIONAL Nº ...54.142
UNIDADE DO HCFMUSP: .Divisão de Anestesia do Instituto Central

3. AVALIAÇÃO DO RISCO DA PESQUISA:

SEM RISCO   RISCO MÍNIMO X   RISCO MÉDIO

RISCO BAIXO   RISCO MAIOR

(probabilidade de que o indivíduo sofra algum dano como consequência imediata ou tardia do estudo)

4.DURAÇÃO DA PESQUISA : .2 anos.

III - REGISTRO DAS EXPLICAÇÕES DO PESQUISADOR AO PACIENTE OU SEU REPRESENTANTE LEGAL SOBRE A PESQUISA:

O senhor (a) está sendo convidado(a) a participar de uma pesquisa, coordenada por um profissional da saúde agora denominado pesquisador. Para poder participar é necessário que o senhor(a) leia este documento com atenção. Ele pode conter palavras que o senhor(a) não entenda. Por favor peça aos responsáveis pelo estudo para explicar qualquer palavra ou procedimentos que o senhor(a) não entenda claramente.

O propósito deste documento é dar o senhor(a) as informações sobre a pesquisa e, se assinado, dará a sua permissão para participar do estudo. O documento descreve o objetivo, procedimentos, benefícios e eventuais riscos ou desconfortos caso queira participar. O senhor(a) só deve participar do estudo se quiser e pode se recusar a participar ou se retirar deste estudo a qualquer momento.

O objetivo desse estudo é determinar a incidência de alteração da função mental no pós-operatório comparando o uso de dexametasona e placebo (soro fisiológico). A Dexametasona tem riscos de efeitos colaterais cardiovasculares, ósseos, retenção de líquidos, aumento de gorduras e glicose no sangue, mas como será usada em dose baixa e única não é de esperar a ocorrência de qualquer efeito colateral. Participarão do estudo pessoas acima dos 60 anos que serão submetidas a cirurgia sob anestesia geral e que não tenham alteração da função mental e que não estejam usando cortisona. Neste estudo o senhor(a) responderá verbalmente a um questionário antes e após a cirurgia e antes da cirurgia o senhor(a) irá receber dexametasona ou placebo (soro fisiológico), por via venosa. O segundo objetivo é a investigação da possível associação entre alterações mentais no pós-operatório e a presença de uma tendência hereditária para este fato, avaliada pela presença de proteínas específicas no organismo. Para tal haverá necessidade da coleta de uma amostra de sangue que será avaliada no laboratório de biologia molecular do InCor. O senhor(a) não terá qualquer risco cirúrgico adicional por isso. A coleta de duas amostras de 3mL de sangue será feita no momento da punção da veia para administração de soro durante a cirurgia e encaminhadas a laboratórios especializados para análise. Os questionários para avaliação neuropsicológica serão feitos por profissional especializado. É através das pesquisas clínicas que ocorrem os avanços na medicina, e sua participação é de fundamental importância. Esta pesquisa não ira trazer benefícios diretos para o senhor(a), mas ela irá nos ajudar a melhor entender a alteração da função mental após cirurgia.

IV - ESCLARECIMENTOS DADOS PELO PESQUISADOR SOBRE GARANTIAS DO SUJEITO DA PESQUISA :

A sua participação neste estudo é voluntária. O senhor(a) tem a liberdade de recusar participar do estudo, ou se aceitar participar, retirar seu consentimento a qualquer momento. Este fato não implicará na interrupção de seu atendimento e tratamento, os quais estão assegurados.

Pela sua participação no estudo, o senhor não receberá qualquer valor em dinheiro ou terá qualquer custo. Em caso de por eventuais danos à saúde decorrentes desta pesquisa, o senhor terá disponibilidade de assistência neste hospital.

As informações relacionadas ao estudo poderão ser inspecionadas pelos médicos que executam a pesquisa e pelas autoridades legais. No entanto, se qualquer informação for divulgada em relatório ou publicação, isto será feito sob forma codificada sem nomes. Esta medida assegura sua confidencialidade. Quando os resultados forem publicados, não aparecerá seu nome, e sim um código. O senhor(a) tem direito de acesso aos seus dados. O senhor(a) pode discutir esta questão mais adiante com seu médico do estudo. Se o senhor(a) ou seus parentes tiver(em) alguma dúvida com relação ao estudo, direitos do paciente, ou no caso de danos relacionados ao estudo, o senhor(a) deve contactar o pesquisador ou sua equipe .Se o senhor(a) tiver dúvidas sobre seus direitos como um paciente de pesquisa, você pode contactar o Comitê de Ética em Pesquisa em Seres Humanos do Hospital (CEP). A CEP trata-se de um grupo de indivíduos com conhecimento científicos e não científicos que realizam a revisão ética inicial e continuada do estudo de pesquisa para mantê-lo seguro e proteger seus direitos.

V. INFORMAÇÕES DE NOMES, ENDEREÇOS E TELEFONES DOS RESPONSÁVEIS PELO ACOMPANHAMENTO DA PESQUISA, PARA CONTATO EM CASO DE INTERCORRÊNCIAS CLÍNICAS E REAÇÕES ADVERSAS.

Dra Maria José Carvalho Carmona

Divisão de Anestesia do ICHC-FMUSP

Av Enéas Carvalho de Aguiar, n 255 – São Paulo

Fone: 11 3069-5012 ou 3069-6335

VI. OBSERVAÇÕES COMPLEMENTARES:

VII - CONSENTIMENTO PÓS-ESCLARECIDO

Declaro que, após convenientemente esclarecido pelo pesquisador e ter entendido o que me foi explicado, consinto em participar do presente Protocolo de Pesquisa.

São Paulo, de de .

____
assinatura do sujeito da pesquisa ou responsável legal

____
assinatura do pesquisador
(carimbo ou nome Legível)

UNIVERSIDADE DE SÃO PAULO
FACULDADE DE MEDICINA

São Paulo, 11 de julho de 2.006.

Ilmo. Sr.

Dr. Hazem Adel Ashmawi

Comissão Assessora de Pesquisa do

Departamento de Cirurgia da FMUSP

Protocolo de entrega de Projeto de Pesquisa

Título do Projeto de Pesquisa: Disfunção cognitiva após cirurgia sob anestesia geral.

Pesquisador Responsável: Profa. Dra. Maria José Carvalho Carmona.

Pesquisadora Executante: Dr. Luis Marcelo Sá Malbouisson.

CO-Autor: Prof. Dr. José Otávio Costa Auler Jr.

Registrado em 10 / 07 / 06 no Livro de Projeto
dos Projetos de Pesquisa da Disciplina de Anestesiologia da
Faculdade de Medicina da USP, sob a folha nº 74
Visto: Tatiana Rivas Rego

IDENTIFICAÇÃO – DISFUNÇÃO COGNITIVA APÓS CIRURGIA SOB ANESTESIA GERAL

Pesquisador Executante – Dr. Luís Marcelo Sá Malbouisson
Pesquisador Responsável – Profa. Dra. Maria José Carvalho Carmona
Co-autor – Prof. Dr. José Otávio Costa Auler jr

APRECIAÇÃO GERAL DO PROJETO

Trata-se de projeto de pesquisa clínica, prospectivo, duplo cego, controlado com placebo, que visa estudar o efeito da administração pré-operatória de dexametasona sobre a disfunção cognitiva pós-operatória (DCPO) em pacientes acima de sessenta anos, sob anestesia geral para cirurgias com tempo de permanência no hospital inferior a vinte e quatro horas. O estudo baseia-se na hipótese que os anti inflamatórios esteroidois apresentam efeito analgésico, de diminuição de fadiga e poupador de opióides, fatores citados como determinantes para a DCPO.

PROJETO

Os objetivos do projeto estão bem definidos? Comente.
O trabalho tem objetivo claro e o desenho do estudo está adequado.

O projeto envolve experimentação com seres humanos de forma a requerer análise de suas implicações éticas? [XX] Sim [ ] Não
Em caso negativo ignore os dois quesitos abaixo.
i) Há parecer da Comissão de Ética da Instituição? [ ] Sim [ ] Não
ii) O projeto contempla adequadamente os aspectos éticos envolvidos? Comente.

Parecer final –
APROVAÇÃO DO PROJETO.

Dr. Hazem Adel Ashmawi
São Paulo, 24 de julho de 2006.

UNIVERSIDADE DE SÃO PAULO
FACULDADE DE MEDICINA

São Paulo, 01 de agosto de 2.006.

Ilmo. Sr.

Prof. Dr. Rolf Gemperli

Presidente da Comissão Assessora de Pesquisa do

Departamento de Cirurgia da FMUSP

Protocolo de entrega de Projeto de Pesquisa

Título do Projeto de Pesquisa: Disfunção cognitiva após cirurgia sob anestesia geral.

Pesquisador Responsável: Profa. Dra. Maria José Carvalho Carmona.

Pesquisadora Executante: Dr. Luis Marcelo Sá Malbouisson.

Co-Autor: Prof. Dr. José Otávio Costa Auler Jr.

Registrado em 10/07/2006 no Livro de Projetos
dos Projetos de Pesquisa da Disciplina de Anestesiologia da
Faculdade de Medicina da USP, sob a folha nº 74
Visto: ____________________

Duke Surgery
DUKE UNIVERSITY MEDICAL CENTER

Dr. Ricardo Pietrobon, MD, PhD, MBA
Center for Excellence in Surgical Outcomes
Assistant Research Professor
Department of Orthopaedic Surgery
1-919-306-2535
rpietro@duke.edu

Caro Senhor ou Senhora
Instituição CAPPesq

Por meio dessa carta, declaramos a participação do CESO (Center for Excellence in Surgical Outcomes – Duke University Medical Center), no projeto "DISFUNÇÃO COGNITIVA APÓS CIRURGIA SOB ANESTESIA GERAL", através do fornecimento de:

1. Programa de coleta de dados via Internet - o programa se chama DADOS-prospective;
2. Assistência para design do estudo – através do PhD do Dr. Pietrobon sobre "validação psicométrica de escalas usando análises psicométricas clássicas" e "item response theory";
3. Tradutores bilíngues cuja primeira língua é português e inglês;
4. Análise estatística das análises psicométricas;
5. Acesso a biblioteca virtual da Duke para referências e pessoal local que pode conseguir referências que não estejam disponíveis on-line;
6. Auxílio para que o artigo seja escrito em português em conformidade com os padrões esperados por revistas internacionais -- esses padrões não são explícitos;
7. Tradutores do artigo para o inglês, isso faz parte de um programa de formação de uma rede internacional de pesquisa clínica que esta sendo montando;
8. Fornecimento dos eletrodos do BIS e da bateria de testes neuro-psicológicos.

Ricardo Pietrobon, MD, PhD, MBA
Director, Center for Excellence in Surgical Outcomes
Duke University Health System
Durham, NC 27710 USA

DIRETORIA CLÍNICA
COMISSÃO DE ÉTICA PARA ANÁLISE DE PROJETOS DE PESQUISA - CAPPesq

TRIAGEM DOCUMENTAL EFETUADA PELA SECRETARIA - 268-2

DATA: 12/09/06

TÍTULO DO PROJETO: Disfunção cognitiva após cirurgia sob anestesia geral

PESQUISADOR(A) RESPONSÁVEL: Dra. Maria José Carvalho Carmona

DEPARTAMENTO: Cirurgia

Devolvemos o Protocolo de Pesquisa para:

☐ - Anexar Cadastro do Protocolo de Pesquisa

☐ - Anexar Cadastro do Pesquisador Responsável

☐ - Anexar Cadastro do Pesquisador Executante

☐ - Anexar Termo de Consentimento Livre e Esclarecido - TCLE

X - Anexar Folha de Rosto SISNEP *(www.saude.gov.br/sisnep)* **A Folha de Rosto apresentada não consta no sistema**

☐- Anexar Aprovação do Departamento

☐- Anexar Identificação do Financiamento Externo e a Fundação de Apoio (Fundação de Faculdade de Medicina ou Fundação Zerbini).

☐ - Anexar Manifestação de Entidades Externas:

☐ - Anexar Manifestação de outros Serviços/Divisões envolvidos na Pesquisa:

☐- Outros:

Marcia Aparecida T. de Carvalho
Secretária da CAPPesq.
Diretoria Clínica do HCFMUSP

DIRETORIA CLÍNICA
COMISSÃO DE ÉTICA PARA ANÁLISE DE PROJETOS DE PESQUISA - CAPPesq

TRIAGEM DOCUMENTAL EFETUADA PELA SECRETARIA - 268

DATA: 11/08/06

TÍTULO DO PROJETO: Disfunção cognitiva após cirurgia sob anestesia geral

PESQUISADOR(A) RESPONSÁVEL: Dra. Maria José Carvalho Carmona

DEPARTAMENTO: Cirurgia

Devolvemos o Protocolo de Pesquisa para:

☐ - Anexar Cadastro do Protocolo de Pesquisa

☐ - Anexar Cadastro do Pesquisador Responsável

☐ - Anexar Cadastro do Pesquisador Executante

☐ - Anexar Termo de Consentimento Livre e Esclarecido - TCLE

☐ - - Anexar Folha de Rosto SISNEP *(www.saude.gov.br/sisnep)*

☐- Anexar Aprovação do Departamento

☐- Anexar Identificação do Financiamento Externo e a Fundação de Apoio (Fundação de Faculdade de Medicina ou Fundação Zerbini).

X - Anexar Manifestação de Entidades Externas: **Ítem 8 do Anexo II - Cadastro do Protocolo de Pesquisa**

☐ - Anexar Manifestação de outros Serviços/Divisões envolvidos na Pesquisa:

☐- Outros:

17/08/06 → encaminhado e-mail de solicitação p/ Dr. Ricardo Pietrobon.

Marcia Aparecida T. de Carvalho
Secretária da CAPPesq.
Diretoria Clínica do HCFMUSP

. 998/06 .

MINISTÉRIO DA SAÚDE
Conselho Nacional de Saúde
Comissão Nacional de Ética em Pesquisa - CONEP

FOLHA DE ROSTO PARA PESQUISA ENVOLVENDO SERES HUMANOS

FR - 109141

Projeto de Pesquisa<br>Disfunção cognitiva após cirurgia sob anestesia geral.		
Área de Conhecimento<br>4.00 - Ciências da Saúde - 4.01 - Medicina - Diag.	Grupo<br>Grupo IA	Nivel<br>Diagnóstico
Área(s) Temática(s) Especial(s)<br>Pesquisa com Cooperação Estrangeira,		Fase<br>Não se Aplica
Unitermos<br>Anestesia geral; Pós-operatório; Disfunção cognitiva.		

Sujeitos na Pesquisa

Nº de Sujeitos no Centro	Total Brasil	Nº de Sujeitos Total	Grupos Especiais	
150	300	300		
Placebo<br>SIM	Medicamentos HIV / AIDS<br>NÃO	Wash-out<br>NÃO	Sem Tratamento Especifico<br>NÃO	Banco de Materiais Biológicos<br>SIM

Pesquisador Responsável

Pesquisador Responsável<br>Maria José Carvalho Carmona	CPF<br>059.174.158-03	Identidade<br>17.760.864
Área de Especialização<br>Anestesiologia	Maior Titulação<br>LIVRE-DOCENTE	Nacionalidade<br>Brasileira
Endereço<br>Rua Rodésia 161, Apto. 82	Bairro<br>Vila Madalena	Cidade<br>São Paulo - SP
Código Postal 05435-020 / Telefone 11-3069-5012 / 11-3816-3986	Fax<br>11-3819-5533	Email<br>maria.carmona@incor.usp.br

Termo de Compromisso

Declaro que conheço e cumprirei os requisitos da Res. CNS 196/96 e suas complementares. Comprometo-me a utilizar os materiais e dados coletados exclusivamente para os fins previstos no protocolo e publicar os resultados sejam eles favoráveis ou não.

Aceito as responsabilidades pela condução científica do projeto acima.

Data: 27/09/2006

[signature] Assinatura

Instituição Onde Será Realizado

Nome<br>HCFMUSP - Hospital das Clínicas da Faculdade de Medicina da Universidade de São Paulo	CNPJ<br>60.448.040/0001-22	Nacional/Internacional<br>Nacional
Unidade/Órgão<br>Divisão de Anestesia	Participação Estrangeira<br>SIM	Projeto Multicêntrico<br>SIM
Endereço<br>Rua Dr. Ouvidio Pires de Campos N. 225	Bairro<br>Cerqueira Cesar	Cidade<br>São Paulo - SP
Código Postal 05403010 / Telefone (11) 3069-6442	Fax<br>(11) 3069-6442	Email<br>cappesq@hcnet.usp.br

Termo de Compromisso

Declaro que conheço e cumprirei os requisitos da Res. CNS 196/96 e suas complementares e como esta instituição tem condições para o desenvolvimento deste projeto, autorizo sua execução.

Nome: José Otávio Costa Auler Jr.

Data: 28/09/2006

[signature] Assinatura

Prof. Dr. José Otávio Costa Auler Junior
Professor Titular da Disciplina de Anestesiologia
Matrícula HC nº: 20.365/1

O Projeto deverá ser entregue no CEP em até 30 dias a partir de 26/09/2006. Não ocorrendo a entrega nesse prazo esta Folha de Rosto será INVALIDADA.

HOSPITAL DAS CLÍNICAS
DA FACULDADE DE MEDICINA
DA UNIVERSIDADE DE SÃO PAULO

Diretoria Clínica

Comissão de Ética para Análise de Projetos de Pesquisa - CAPPesq.

PARECER

PROTOCOLO DE PESQUISA Nº: 998/06	Data de entrada: 29/09/06<br>Data sessão: 08/11/06

TÍTULO DA PESQUISA: Disfunção cognitiva após cirurgia sob anestesia geral

PESQUISADOR(A) RESPONSÁVEL: Maria José Carvalho Carmona

DEPARTAMENTO: Cirurgia

CONSIDERAÇÕES DO RELATOR APROVADAS PELO PLENÁRIO:

Trata-se de um protocolo de pesquisa prospectivo que objetiva analisar o uso de dexametasona versus placebo em 150 pacientes submetidos a cirurgia sob anestesia geral.
Faz necessário atender as seguintes solicitações:

1. Definir o Centro coordenador no Brasil, pois o projeto precisa ser apreciado pela CONEP devido ao envio de informações para Duke University Medical Center (instituição em outro país).
2. Esclarecer se participarão pacientes do HC, pois consta que participarão do estudo 150 pacientes, mas no item 8 da folha de rosto está explicitado que serão 150 pacientes do HC, 75 pacientes do Hospital Nossa Senhora do Carmo e 75 pacientes Hospital Ministro Costa Cavalcanti.
3. O projeto será submetido aos outros CEPs?
4. Há necessidade de anexar carta de anuência das outras instituições participantes.
5. O TCLE deve ser aplicado antes da bateria de teste, pois ao respondê-la, o paciente torna-se sujeito de pesquisa, pois este é um procedimento incluso na pesquisa.
6. Esclarecer qual procedimento é utilizado no HC.
7. Justificar o uso do placebo.
8. Acrescentar na introdução o embasamento científico para a realização do trabalho.

APROVAR	☐	REPROVAR
X DEVOLVER PARA ATENDER AS CONSIDERAÇÕES	☐	CIENTE

TEMÁTICA ESPECIAL:	☐ SIM	☐ NÃO
CONEP:	☐ SIM	☐ NÃO
SVS (SECRETARIA DE VIGILÂNCIA SANITÁRIA):	☐ SIM	☐ NÃO

As respostas às pendências, deverão ser apresentadas no prazo de 30 dias.

Profº Euclides Ayres de Castilho
Presidente
Comissão de Ética P/ Análise de Projetos de Pesquisa

Comissão de Ética para Análise de Projetos de Pesquisa do HCFMUSP e da FMUSP
Diretoria Clínica do Hospital das Clínicas da Faculdade de Medicina da Universidade de São Paulo
Rua Ovídio Pires de Campos. 225, 5º andar - CEP 05430 010 - São Paulo - SP
Fone: 011 - 30696442 fax : 011 - 3069 6492 - e-mail : cappesq@hcnet.usp.br / secretariacappesq@hcnet.usp.br

RECEBIDO POR
DATA 1 8 FEV. 2007
Comissão de Ética para Análise de Projetos de Pesquisa - CAPPesq - HCFMUSP

São Paulo, 08 de Janeiro de 2007

Ilmo. Sr.
Prof. Dr. Euclydes Ayres de Castilho
D.D. Presidente de Ética para Análise de Projetos de Pesquisa
HCFMUSP

Ref: Projeto de Pesquisa nº 998/06: **Disfunção Cognitiva após cirurgia sob anestesia geral.**

Prezado Professor Euclides

Agradecemos a análise da CAPPesq ao projeto de pesquisa "**Disfunção Cognitiva após cirurgia sob anestesia geral**".

Após re-discussão do projeto com o centro coordenador (Duke University) e considerando as dificuldade de realização de testes neuropsicológicos padronizados e por profissionais qualificados nas diferentes instituições, decidiu-se pela execução do projeto apenas no HC-FMUSP. Desta forma, estamos re-submetendo o projeto e respondendo aos questionamentos do relator:

1- O estudo será unicêntrico no Brasil, no Intituto Central do HC-FMUSP, com coordenação da Duke University.
2- Serão incluídos 300 pacientes do Instituto Central do HC-FMUSP;
3- O projeto será submetido unicamente à aprovação da provação na CAPPesq e CONEP;
4- Considerando as dificuldades de realização do estudo multicêntrico neste caso, especialmente devido à necessidade de realização das avaliações neuropsicológicas de forma padronizada, o estudo não será mais submetido a outros comitês de ética e será realizado, após aprovação da CAPPEsq, apenas no ICHC;
5- O TCLE será aplicado durante a visita pré-anestésica, no dia anterior à cirurgia, antes da aplicação da primeira bateria de testes;
6- A técnica anestésica descrita no projeto é aquela comumente utilizada no HCFMUSP. A tecnologia de monitorização com índice bispectral (BIS), para avaliação do grau de hipnose durante a

Av. Dr. Enéas de Carvalho Aguiar, 44 - CEP: 05403-900 - São Paulo - SP - Brasil - Fone (011) 3069-5232

anestesia, encontra-se em fase de implantação nesta instituição e é de uso rotineiro em diversas instituições de referência. O uso profilático de corticosteróides não faz parte da rotina diária para o tipo de cirurgia estudado, mas é habitual em casos de anestesia para cirurgia cardíaca com circulação extracorpórea, dentre outros objetivos para minimizar os efeitos neurológicos e as alterações cognitivas relacionadas à CEC;

7- O uso de placebo se justifica neste estudo por não haver outro fármaco eficaz ou padronizado ao qual o corticosteróide possa ser comparado. Há necessidade de comparação entre a dexametasona e outra substância com aparência semelhante (soro fisiológico), onde o pesquisador não pode saber o que está sendo administrado;

8- As referências sobre o embasamento científico para a realização do estudo estão mencionadas na introdução.

Agradeço antecipadamente e coloco-me à disposição para esclarecimentos adicionais que se fizerem necessários.

Atenciosamente

Maria José Carvalho Carmona
Pesquisador Gerente

Av. Dr. Enéas de Carvalho Aguiar, 44 - CEP: 05403-900 - São Paulo - SP - Brasil - Fone (011) 3069-5232

HOSPITAL DAS CLÍNICAS
DA FACULDADE DE MEDICINA
DA UNIVERSIDADE DE SÃO PAULO

Diretoria Clínica

Comissão de Ética para Análise de Projetos de Pesquisa - CAPPesq.

PARECER

PROTOCOLO DE PESQUISA Nº: 998/06	Data de entrada: 29/09/06<br>Data sessão: 08/11/06

TÍTULO DA PESQUISA: Disfunção cognitiva após cirurgia sob anestesia geral

PESQUISADOR(A) RESPONSÁVEL: Maria José Carvalho Carmona

DEPARTAMENTO: Cirurgia

CONSIDERAÇÕES DO RELATOR APROVADAS PELO PLENÁRIO:

Trata-se de um protocolo de pesquisa prospectivo que objetiva analisar o uso de dexametasona versus placebo em 150 pacientes submetidos a cirurgia sob anestesia geral

Faz necessário atender as seguintes solicitações:

1. Definir o Centro coordenador no Brasil, pois o projeto precisa ser apreciado pela CONEP devido ao envio de informações para Duke University Medical Center (instituição em outro país).
2. Esclarecer se participarão pacientes do HC, pois consta que participarão do estudo 150 pacientes, mas no item 8 da folha de rosto está explicitado que serão 150 pacientes do HC, 75 pacientes do Hospital Nossa Senhora do Carmo e 75 pacientes Hospital Ministro Costa Cavalcanti.
3. O projeto será submetido aos outros CEPs?
4. Há necessidade de anexar carta de anuência das outras instituições participantes.
5. O TCLE deve ser aplicado antes da bateria de teste, pois ao respondê-la, o paciente torna-se sujeito de pesquisa, pois este é um procedimento incluso na pesquisa.
6. Esclarecer qual procedimento é utilizado no HC.
7. Justificar o uso do placebo.
8. Acrescentar na introdução o embasamento científico para a realização do trabalho.

APROVAR	☐ REPROVAR
X DEVOLVER PARA ATENDER AS CONSIDERAÇÕES	☐ CIENTE

TEMÁTICA ESPECIAL:	☐ SIM	☐ NÃO
CONEP:	☐ SIM	☐ NÃO
SVS (SECRETARIA DE VIGILÂNCIA SANITÁRIA):	☐ SIM	☐ NÃO

As respostas às pendências, deverão ser apresentadas no prazo de 30 dias.

Profº Euclides Ayres de Castilho
Presidente
Comissão de Ética P/ Análise de Projetos de Pesquisa

DISCIPLINA DE ANESTESIOLOGIA – FMUSP
INSTITUTO CENTRAL DO HCFMUSP

Protocolo de Pesquisa

DISFUNÇÃO COGNITIVA APÓS CIRURGIA SOB ANESTESIA GERAL

Pesquisadores:
Profa. Dra. Maria José Carvalho Carmona
Dra. Kátia Osternack Pinto
Dr. Rólisson G. B. Lellis
Dr. Luiz Marcelo Sá Malbouisson
Prof. Dr. José Otávio Costa Auler Jr.

2006

DISFUNÇÃO COGNITIVA APÓS CIRURGIA SOB ANESTESIA GERAL

RESUMO

Alguns dos determinantes de disfunção cognitiva pós-operatória são: o uso de opióides no período pós-operatório, falta de atividade física, fadiga pós-operatória, a dor pós-operatória, a qualidade de vida, além de possível predisposição genética. O uso de corticóide pré-operatório diminui a dor pós-operatória, a necessidade de opióides, além de diminuir a fadiga, a incidência de náusea e vômito, o período de recuperação e o tempo de retorno às atividades diárias no período pós-operatório, sendo freqüentemente utilizado como coadjuvante em anestesia. Os objetivos deste estudo são: 1) determinar a incidência de disfunção cognitiva no pós-operatório comparando dexametasona *versus* placebo em 300 pacientes submetidos à cirurgia sob anestesia geral; 2) Investigar a associação entre alterações cognitivas no pós-operatório e a presença do alelo ε4 do gene da apolipoproteína e de marcadores bioquímicos séricos para lesão neuronal, como a proteína S100β e a enolase específica do neurônio (NSE). Serão aplicados testes neuropsicológicos para avaliar o estado mental geral, aprendizagem, atenção, percepção visuoespacial, memória imediata, operacional e de evocação e habilidades executivas, incluindo velocidade de processamento. Esta bateria define o índice cognitivo da estabilidade (medida longitudinal do status cognitivo em avaliações repetidas que detectam mudanças estatisticamente significantes no funcionamento do sistema nervoso central). Esta avaliação será realiza antes da cirurgia, na noite após a cirurgia e nos dias pós-operatórios 3, 7, 21, e 90. A anestesia geral seguirá os padrões gerais incluindo Propofol (2-3 mg/kg), rocuronio (0.5 mg/kg), e fentanil (1-2 µg/kg) para indução. A anestesia subseqüente e mantida com sevoflurano (1.0-2.5%) e N_20 em 40% de oxigênio, rocuronio (doses suplementares serão administradas se necessário para manutenção de anestesia cirúrgica), e fentanil (doses suplementares 0.5-10 µg/kg com dose máxima de 250 µg). A administração de sevoflurano será ajustada com o objetivo de manter os níveis de hipnose avaliada pelo índice bispectral (BIS) de acordo com os grupos do estudo: os participantes serão randomizados em 2 grupos: anestesia superficial (índice bispectral entre 45 e 55) and anestesia profunda (índice bispectral entre 35 e 45). Ambos níveis são aceitáveis para anestesia geral e são atualmente usados por anestesiologistas. Após venóclise periférica, antes da indução anestésica, será colhida uma amostra de sangue para determinação do perfil genotípico da Apoε e dosagem de S100β e NSE. Os resultados dos escores dos testes em cada grupo serão comparados através do teste de Friedmann, e entre os grupos será utilizado o teste de Kruskal Wallis. Porcentagens serão comparadas pelo do teste de Qui-Quadrado. Os dados serão expressos como mediana e intervalo de confiança de 95%. Serão considerados significativos resultados cujos valores p sejam menores que 0,05 nos testes estatísticos.

DISFUNÇÃO COGNITIVA APÓS CIRURGIA SOB ANESTESIA GERAL

1 – INTRODUÇÃO

Disfunção cognitiva no pós-operatório imediato de cirurgias sob anestesia geral é um evento relativamente freqüente, especialmente em pacientes geriátricos (Rasmussen et al., 2000, Ancelin et al., 2001, Bekker et al., 2003, Wu et al., 2004, Cohendy et al., 2005 e Xie & Tanzi, 2006). Na maioria dos procedimentos é reversível, podendo ser permanente em menos de 1% dos casos. Procedimentos sob anestesia regional também podem causar, com menor freqüência, disfunção cognitiva transitória no pós-operatório imediato (Campbell et al., 1993, Rasmussen et al., 2003 e Canet et al., 2003).

O uso de corticóide pré-operatório diminui a dor pós-operatória e também a necessidade de opióides. Diminui fadiga, náusea e vômito, período de recuperação e tempo de retorno às atividades diárias no período pós-operatório. Todos estes fatores melhoram qualidade de vida. Estudos mostram que alguns dos determinantes de disfunção cognitiva pós-operatória são: o uso de opióides no período pós-operatório, falta de atividade física, fadiga pós-operatória, dor pós-operatória e qualidade de vida (Wu et al., 2004, Rasmussen et al., 2005 e Benoit et al., 2005).

Se o corticóide modifica positivamente fatores que estão ligados à disfunção cognitiva como dor, uso de opióides, fadiga, recuperação pós-operatória, convalescença, qualidade de vida e mais rápido retorno às atividades físicas, ele também modificaria positivamente a função cognitiva no período pós-operatório.

2 – OBJETIVOS

Os objetivos deste estudo são:

1) determinar a incidência de disfunção cognitiva no pós-operatório comparando dexametasona *versus* placebo em 300 pacientes submetidos à cirurgia sob anestesia geral;
2) Investigar a associação entre alterações cognitivas no pós-operatório e a presença do alelo ε4 do gene da apolipoproteína E de marcadores bioquímicos séricos para lesão neuronal, como a proteína S100β e a enolase específica do neurônio (NSE).

3 – CASUÍSTICA E MÉTODOS

Após aprovação pela CAPPesq e obtenção de auxílio financeiro para o estudo, serão estudados 300 pacientes idosos de ambos os sexos submetidos à anestesia geral para procedimentos cirúrgicos gerais sem utilização de circulação extracorpórea.

Serão aplicados testes neuropsicológicos para avaliar o estado mental geral, aprendizagem, atenção, percepção visuoespacial, memória imediata, operacional e de evocação e habilidades executivas, incluindo velocidade de processamento. Esta bateria define o índice cognitivo da estabilidade (medida longitudinal do status cognitivo em avaliações repetidas que detectam mudanças estatisticamente significantes no funcionamento do sistema nervoso central). A avaliação será realiza antes da cirurgia, na noite após a cirurgia e nos dias pós-operatórios 3, 7, 21, e 90.

Critérios de inclusão: paciente acima de 60 anos, submetido a videocirurgia para herniorrafia inguinal, operação de Nissen ou colecistectomia sob anestesia geral com permanência hospitalar de até 24 horas.

Critérios de exclusão: idade inferior a 60 anos, história pregressa de doença cerebral ou demência, outras doenças psiquiátricas que afetem a cognição, falta de domínio da língua portuguesa, uso de corticóide ou de opióde.

Os pacientes que preencherem os critérios para participarem e aceitarem participar do estudo serão contatados por telefone e será aplicada a bateria de testes. Após os testes o paciente estará liberado ou não para fazer parte do estudo.

No dia da cirurgia, pacientes devem assinar um termo de consentimento em duas vias. Uma cópia ficará com o paciente e outra com o médico.

Após obter o consentimento, médico irá aplicar a substância designada para o paciente duas horas antes da indução da anestesia. Essa droga será determinada aleatoriamente sem o conhecimento do médico, podendo ser: dexametasona, 8 mg IV ou placebo (soro fisiológico), no mesmo volume.

Após venóclise periférica, antes da indução anestésica, serão colhidas duas amostras de sangue de para determinação do perfil genotípico da Apoε e dosagem de S100β e NSE. As amostras serão processadas no Laboratório de Biologia Molecular do InCor (APOε4) e Laboratório de Neurociências da UFRGS (S100β e NSE).

A anestesia geral será induzida com utilização dos seguintes fármacos: Propofol (2-3 mg/kg), rocuronio (0.5 mg/kg), e fentanil (1-2 μg/kg) para indução. A anestesia subseqüente será mantida com sevoflurano (1.0-2.5%) e N_20 em 40% de oxigênio, rocuronio (doses suplementares serão administradas se necessário para manutenção de anestesia cirúrgica), e fentanil (doses suplementares 0.5-10 μg/kg com dose máxima de 250 μg). Variação em requerimentos individuais preclude administração de doses idênticas para cada paciente. Ao invés, a administração de sevoflurano deverá ser ajustada pelo anestesiologista com o objetivo de manter of níveis do BIS (hipnose) de acordo com os grupos do estudo, e fentanil será administrado com o objetivo de manter a pressão arterial e freqüência cardíaca (analgesia) dentro de 20% dos valores pré-indução. A ventilação será controlada mecanicamente para manter a pressão de dióxido de carbono próximo a 35 mmHg. Fentanil (1-2 μg/kg) será dado ao final da

cirurgia para melhorar a analgesia durante a recuperação anestésica. Soro fisiológico 5-10 ml/kg/hora será dado durante a cirurgia.

O índice bispectral mede a atividade cerebral cortical (hipnose) numa escala de 0 a 100, onde 100 representa o estado "acordado" e 0 representa um eletroencefalograma isoelétrico. Medidas abaixo de 70 estão relacionadas à inconsciência e ausência de memória necessária para a anestesia geral. Neste estudo, os participantes serão randomizados em 2 grupos: anestesia superficial (índice bispectral entre 45 e 55) and anestesia profunda (índice bispectral entre 35 e 45). Ambos os níveis são aceitáveis para anestesia geral e são atualmente usados por anestesiologistas.

A pele da região onde o monitor vai ser aplicado deve estar limpa e seca. Use álcool e seque esta área da pele.

Aplique o sensor na testa do paciente. Coloque o círculo número 1 no centro aproximadamente 2 cm acima da ponte nasal. Coloque o círculo número 3 na área temporal.

Importante: Aplique pressão em torno do sensor (incluindo as áreas entre os círculos) para garantir a adesão adequada. Em seguida, pressione os círculos 1, 2 e 3 por 5 segundos para assegurar o contato dos eletrodos à pele.

Conecte o sensor aplicado na testa do participante no cabo do índice bispectral.

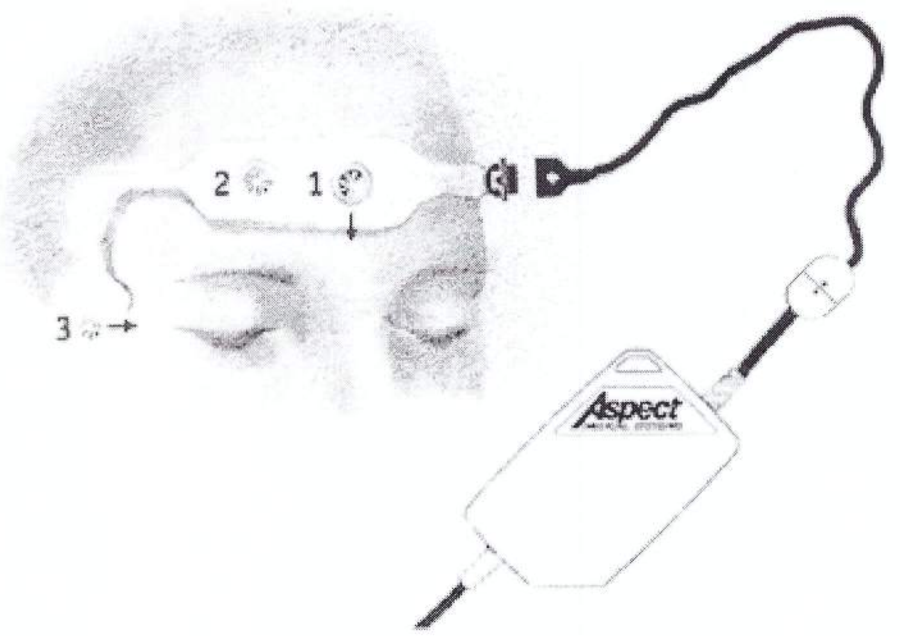

A avaliação da disfunção cognitiva nos dois grupos será realizada através de testes neuropsicológicos, padronizados especificamente para avaliar as funções cognitivas superiores, e questionários para avaliar as características sócio-demográficas, questionamentos sobre a percepção pessoal da saúde do paciente. Estes testes serão aplicados no pré-operatório, na noite anterior à cirurgia,

antes da administração de medicações pré-anestésicas ou sedativas, no 3°, 7°, 21° e 90° dia pós-operatório, conforme detalhado abaixo.

Fase 1:

Período: pré e pós-operatório imediatos
Procedimento: aplicar pessoalmente em enfermaria
Instrumento: ISPOCD

Fase 2:

Período: 3° e 7° dias pós-operatórios
Procedimento: aplicar via telefone
Instrumento: TICS

Fase 3:

Período: 21° dia pós-operatório
Procedimento: aplicar pessoalmente em retorno ambulatorial
Instrumento: ISPOCD

Fase 4:

Período: 90° dia pós-operatório
Procedimento: aplicar via telefone
Instrumento: TICS

Os dados serão analisados utilizando-se o software de análise estatística SPSS versão 10. Os dados demográficos como idade, peso, altura serão comparados nos quatro grupos através de análise de variância univariada seguida de teste de comparações múltiplas se aplicável. As variáveis colhidas através de testes neuropsicológicos aplicados antes e depois do procedimento cirúrgico são de natureza qualitativa ou ordinal, sendo atribuídos resultados numéricos e os resultados finais dos testes serão expressos em escores. Os resultados dos escores dos testes em cada grupo serão comparados através do teste de Friedmann, e entre os grupos será utilizado o teste de Kruskal Wallis. Mann Whitney para comparações da evolução do sujeito Porcentagens serão comparadas através do teste de Qui-Quadrado Os dados serão expressos como mediana e intervalo de confiança de 95%. Serão considerados significativos resultados cujos valores p sejam menores que 0,05 nos testes estatísticos.

INSTRUMENTOS

1- Informações Sócio-demográficas

Serão investigadas as informações sobre idade, sexo, escolaridade, estado civil, ocupação, e demais informações relevantes para compor o perfil sociodemográfico da amostra, bem como a compatibilidade com os critérios de exclusão e inclusão, conforme detalhado em anexo.

2- Versão Brasileira do Questionário de Qualidade de Vida SF-36

O SF-36 investiga condições relacionadas à saúde, atividades de vida diária, produtividade, problemas emocionais, relacionamentos, motivação e outras condições que traçam o perfil da qualidade de vida a partir da percepção do próprio sujeito, conforme apresentado em anexo.

3- International Study of Postoperative Cognitive Dysfunction - ISPOCD

A bateria neuropsicológica proposta neste estudo seguirá o protocolo sugerido pelo Estudo Internacional de Disfunção Cognitiva Pós-Cirúrgica (do inglês, International Study of Postoperative Cognitive Disfunction - ISPOCD) para avaliar as funções de memória, velocidade sensoriomotora, flexibilidade mental e produtividade motora, com a finalidade de detectar disfunções neuropsicológicas sutis que possam ocorrer em pacientes após cirurgia com anestesia geral ou regional (Rasmussen et al., 1999, Rasmussen et al., 2001 e Rasmussen et al., 2002). Para detalhes, ver anexos.

4- Entrevista Telefônica para o Status Cognitivo (Telephone Interview for Cognitive Status - TICS)

Instrumentos padronizados para realização de entrevista telefônica têm se mostrado válidos e sensíveis, sendo muito indicados para utilizar em situações onde a avaliação de rastreio cognitivo realizada pessoalmente é impraticável ou ineficiente, como, por exemplo, em pesquisas epidemiológicas de grandes populações ou com pacientes que estão incapacitados de comparecerem ao retorno clínico (Ferrucci et al., 1998, De Jager et al., 2003, Barber & Stott, 2004, Musselwhite et al., 2006 e Dal Forno et al., 2006).

O TICS - Entrevista Telefônica para o Status Cognitivo é um teste padronizado para avaliação do funcionamento neuropsicológico que foi desenvolvido para utilizar em situações onde a avaliação de rastreio cognitivo realizada pessoalmente é impraticável ou ineficiente, como, por exemplo, em pesquisas epidemiológicas de grandes populações ou com pacientes que estão incapacitados de comparecerem ao retorno clínico. É muito útil também para avaliar pessoas com incapacidade para ler ou escrever, pois pode ser aplicado pessoalmente através de entrevista, exigindo apenas capacidade de compreensão verbal.

O teste consiste em um roteiro de entrevista com onze itens abordando as habilidades de orientação espacial e temporal controle mental, memória, informação geral, repetição de sentenças, memória semântica, praxias e antônimos de palavras, conforme detalhado em anexo.

4- ANÁLISE DO RISCO

O risco da pesquisa é mínimo, pois não modificará a técnica anestésica ou cirúrgica rotineiramente utilizada. A única intervenção proposta é a utilização de dexametasona em metade da população estudada, fármaco este freqüentemente utilizado em anestesia para prevenção de vômitos pós-operatórios.

5- CRONOGRAMA

Estimamos um prazo máximo de 24 meses para o estudo, sendo 18 meses para a coleta dos dados, 2 meses para análise estatística completa dos resultados obtidos e 2 a 4 meses para formulação do artigo científico a ser publicado.

6- ORIGEM DOS RECURSOS FINANCEIROS

Após aprovação pela CAPPesq, auxílio à pesquisa em valor aproximado de R$20.000,00 será solicitado ao CNPQ ou FAPESP para compra dos eletrodos para monitorização do índice bispectral (BIS).

7- PROCEDIMENTOS APÓS A REALIZAÇÃO DA PESQUISA

Os dados e resultados obtidos serão armazenados em banco de dados seguro e sigiloso. Após a análise completa dos resultados, serão submetidos à publicação em periódico científico indexado apropriado.

8- REFERÊNCIAS BIBLIOGRÁFICAS

- Ancelin ML, de Roquefeuil G, Ledesert B, Bonnel F, Cheminal JC, Ritchie K. Exposure to anaesthetic agents, cognitive functioning and depressive symptomatology in the elderly. Br J Psychiatry 2001; 178:360-6.
- Barber M, Stott DJ. Validity of the Telephone Interview for Cognitive Status (TICS) in post-stroke subjects. Int J Geriatr Psychiatry. 2004; 19(1):75-9.
- Bekker AY, Weeks EJ. Cognitive function after anaesthesia in the elderly. Best Pract Res Clin Anaesthesiol 2003; 17:259-72.
- Benoit AG, Campbell BI, Tanner JR, et al. Risk factors and prevalence of perioperative cognitive dysfunction in abdominal aneurysm patients. J Vasc Surg 2005; 42:884-90.
- Bertolucci PHF, Brucki SMD, Campacci SR, Juliano Y. O Mini-Exame do Estado Mental em uma população geral. Arq. Neuro-Psiquiatr. 1994;52(1):1-7.
- Campbell DN, Lim M, Muir MK, et al. A prospective randomised study of local versus general anaesthesia for cataract surgery. Anaesthesia 1993; 48:422-8.
- Canet J, Raeder J, Rasmussen LS, et al. Cognitive dysfunction after minor surgery in the elderly. Acta Anaesthesiol Scand 2003; 47:1204-10.
- Cohendy R, Brougere A, Cuvillon P. Anaesthesia in the older patient. Curr Opin Clin Nutr Metab Care 2005; 8:17-21.

- Dal Forno G, Chiovenda P, Bressi F, Ferreri F, Grossi E, Brandt J, Rossini PM, Pasqualetti P. Use of an Italian version of the telephone interview for cognitive status in Alzheimer's disease. int J Geriatr Psychiatry. 2006; 21(2):126-33.
- De Jager CA, Budge MM, Clarke R. Utility of TICS-M for the assessment of cognitive function in older adults. In J Geriatr Psychiatry. 2003 ; 18(4) :318-24.
- Ferrucci L, Del Lungo I, Guralnik JM, Bandinellil S, Benvenuti E, Salani B, Lamponi M, et al. Is the telephone interview for cognitive status a valid alternative in persons who cannot be evaluated by the Mini Mental State Examination? Aging. 1998; 10(4):332-8.
- Folstein MF, Folstein SE, McHugh PR. Mini-Mental State: a practical method for grading the cognitive state for the clinician. J. Psychiatr Res. 1975;12:189-98.
- Musselwhite K, Cuff L, McGregor L, King KM. The telephone interview is an effective method of data collection in clinical nursing research: a discussion paper. Intern J Nursing Stu. 2006; [Epub ahead of print].
- Rasmussen LS, Christiansen M, Eliasen K, Sander-Jensen K, Moller JT. Acta Anaesthesiol Scand. 2002; 46:547-51.
- Rasmussen LS, Johnson T, Kuipers HM, et al. Does anaesthesia cause postoperative cognitive dysfunction? A randomised study of regional versus general anaesthesia in 438 elderly patients. Acta Anaesthesiol Scand 2003; 47:260-6.
- Rasmussen LS, Larsen K, Houx P, Skovgaard LT, Hanning CD, Moller JT et al. Acta Anaesthesiol Scand. 2001; 45:275-89.
- Rasmussen LS, Moller JT. Central nervous system dysfunction after anesthesia in the geriatric patient. Anesthesiol Clin North America 2000; 18:59-70, vi.
- Rasmussen LS, O'Brien JT, Silverstein JH, et al. Is peri-operative cortisol secretion related to post-operative cognitive dysfunction? Acta Anaesthesiol Scand 2005; 49:1225-31.
- Rasmussen LS, Steentoft A, Rasmussen H, Kristensen PA, Moller JT. Benzodiazepines and postoperative cognitive dysfunction in the elderly. ISPOCD Group. International Study of Postoperative Cognitive Dysfunction. Br J Anaesth 1999; 83:585-9.
- Wu CL, Hsu W, Richman JM, Raja SN. Postoperative cognitive function as an outcome of regional anesthesia and analgesia. Reg Anesthesia Pain Med. 2004; 29(3):257-268.
- Xie Z, Tanzi RE. Alzheimer's disease and post-operative cognitive dysfunction. Exp Gerontol 2006; 41:346-359

OUTRAS REFERÊNCIAS RELACIONADAS

Selnes OA, McKhann GM. Neurocognitive complications after coronary artery bypass surgery. Ann Neurol 2005; 57:615-21.

Rohan D, Buggy DJ, Crowley S, et al. Increased incidence of postoperative cognitive dysfunction 24 hr after minor surgery in the elderly. Can J Anaesth 2005; 52:137-42.

Rodriguez RA, Tellier A, Grabowski J, et al. Cognitive dysfunction after total knee arthroplasty: effects of intraoperative cerebral embolization and postoperative complications. J Arthroplasty 2005; 20:763-71.

Pratico C, Quattrone D, Lucanto T, et al. Drugs of anesthesia acting on central cholinergic system may cause post-operative cognitive dysfunction and delirium. Med Hypotheses 2005; 65:972-82.

Arain SR, Barth CD, Shankar H, Ebert TJ. Choice of volatile anesthetic for the morbidly obese patient: sevoflurane or desflurane. J Clin Anesth 2005; 17:413-9.

Iohom G, Szarvas S, Larney V, et al. Perioperative plasma concentrations of stable nitric oxide products are predictive of cognitive dysfunction after laparoscopic cholecystectomy. Anesth Analg 2004; 99:1245-52, table of contents.

Heyer EJ, Sharma R, Rampersad A, et al. A controlled prospective study of neuropsychological dysfunction following carotid endarterectomy. Arch Neurol 2002; 59:217-22.

Ritchie K, Polge C, de Roquefeuil G, Djakovic M, Ledesert B. Impact of anesthesia on the cognitive functioning of the elderly. Int Psychogeriatr 1997; 9:309-26.

Williams-Russo P, Sharrock NE, Mattis S, Szatrowski TP, Charlson ME. Cognitive effects after epidural vs general anesthesia in older adults. A randomized trial. Jama 1995; 274:44-50.

Moller JT, Svennild I, Johannessen NW, et al. Perioperative monitoring with pulse oximetry and late postoperative cognitive dysfunction. Br J Anaesth 1993; 71:340-7.

ANEXOS: Instrumentos utilizados no protocolo de pesquisa

Informações Sócio-demográficas

Critérios para inclusão no estudo:

- Pacientes acima de 60 anos
- Submetido à cirurgia de pequeno porte
- Sob anestesia geral

Critérios para exclusão:

1. Idade inferior a 60 anos
2. História pregressa de doença cerebral ou demência
3. Outras doenças psiquiátricas que afetem a cognição
4. Falta de domínio da língua portuguesa
5. Estar fazendo uso de corticóide

- Nome: ____
- Diagnóstico: ____
- Sexo: masculino feminino
- Idade (anos): __________
- Peso (quilos): __________
- Altura (centímetros): __________

Estado civil

( ) solteiro (a)
( ) casado(a)
( ) divorciado(a)
( ) viúvo(a)

Emprego

( ) empregado(a)
( ) desempregado(a)
( ) aposentado(a)

Salário mensal:

( ) 0
( ) 0-1 salário mínimo
( )1-5 salários mínimos
( )6-10 salários mínimos
( )10-15 salários mínimos
( )15-20 salários mínimos
( ) mais que 20 salários mínimos

Escolaridade

( ) Nenhuma
( ) Primário incompleto
( ) Primário completo
( ) Ginásio completo
( ) Colegial completo
( ) Superior completo
(..) Pós-graduação

Versão Brasileira do Questionário de Qualidade de Vida SF-36

Instruções: Esta pesquisa questiona você sobre sua saúde. Estas informações nos manterão informados de como você se sente quão bem você é capaz de fazer suas atividades de vida diária. Responda cada questão marcando a resposta como indicado. Caso você esteja inseguro ou em dúvida em como responder, por favor, tente responder o melhor que puder.

1. Em geral, você diria que a sua saúde é:

(circule uma)

Excelente	Muito boa	Boa	Ruim	Muito ruim
1	2	3	4	5

2. Comparada há um ano atrás, como você classificaria a sua saúde em geral, agora?

(circule uma)

Muito melhor	Um pouco melhor	Quase a mesma	Um pouco pior	Muito pior
1	2	3	4	5

3. Os seguintes itens são sobre atividades que você poderia fazer atualmente durante um dia comum. Devido a sua saúde, você teria dificuldade para fazer essas atividades? Neste caso, quanto?

(circule um número em cada linha)

Atividades	Sim. Dificulta muito	Sim. Dificulta um pouco	Não. Não dificulta de modo algum
A. Atividades vigorosas, que exigem muito esforço, tais como correr, levantar objetos pesados, participar em esportes árduos.	1	2	3
b. Atividades moderadas, tais como mover uma mesa, passar aspirador de pó, jogar bola, varrer a casa.	1	2	3
c. Levantar ou carregar mantimentos	1	2	3
d. Subir vários lances de escada	1	2	3
e. Subir um lance de escadas	1	2	3
f. Curvar-se, ajoelhar-se ou dobrar-se	1	2	3
g. Andar mais de 1 quilômetro	1	2	3
h. Andar vários quarteirões	1	2	3
i. Andar um quarteirão	1	2	3
j. Tomar banho ou vestir-se	1	2	3

4. Durante as últimas 4 semanas, você teve algum dos seguintes problemas com o seu trabalho ou com alguma atividade diária regular, como conseqüência de sua saúde física?

(circule uma em cada linha)

	Sim	Não
a. Você diminuiu a quantidade de tempo que se dedicava ao seu trabalho ou a outras atividades?	1	2
b. Realizou menos tarefas do que você gostaria?	1	2
c. Esteve limitado no seu tipo de trabalho ou em outras atividades?	1	2
d. Teve dificuldade de fazer seu trabalho ou outras atividades (p.ex.: necessitou de um esforço extra)?	1	2

5. Durante as últimas 4 semanas, você teve alguns dos seguintes problemas com o seu trabalho ou outra atividade regular diária, como conseqüência de algum problema emocional (como sentir-se deprimido ou ansioso)?

(circule uma em cada linha)

	Sim	Não
a. Você diminuiu a quantidade de tempo que se dedicava ao seu trabalho ou a outras atividades?	1	2
b. Realizou menos tarefas do que você gostaria?	1	2
c. Não trabalhou ou não fez qualquer das atividades com tanto cuidado como geralmente faz?	1	2

6. Durante as últimas 4 semanas, de que maneira sua saúde física ou problemas emocionais interferiram nas suas atividades sociais normais, em relação a família, vizinhos, amigos ou em grupo?

(circule uma)

De forma nenhuma	Ligeiramente	Moderadamente	Bastante	Extremamente
1	2	3	4	5

7. Quanta dor no corpo você teve durante as últimas 4 semanas?

(circule uma)

Nenhuma	Muito leve	Leve	Moderada	Grave	Muito grave
1	2	3	4	5	6

8. Durante as últimas 4 semanas, quanto a dor interferiu com o seu trabalho normal (incluindo tanto o trabalho fora de casa e dentro de casa)?

(circule uma)

De maneira alguma	Um pouco	Moderadamente	Bastante	Extremamente
1	2	3	4	5

9. Estas questões são sobre como você se sente e como tudo tem acontecido com você durante as últimas 4 semanas. Para cada questão, por favor dê uma resposta que mais se aproxime da maneira como você se sente. Em relação às últimas 4 semanas.

(circule um número para cada linha)

	Todo tempo	A maior parte do tempo	Uma boa parte do tempo	Alguma parte do tempo	Uma pequena parte do tempo	Nunca
a. Quanto tempo você tem se sentido cheio de vigor, cheio de vontade, cheio de força?	1	2	3	4	5	6
b. Quanto tempo você tem se sentido uma pessoa muito nervosa?	1	2	3	4	5	6
c. Quanto tempo você tem se sentido tão deprimido que nada pode animá-lo?	1	2	3	4	5	6
d. Quanto tempo você tem se sentido calmo e tranqüilo?	1	2	3	4	5	6
e. Quanto tempo você tem se sentido com muita energia?	1	2	3	4	5	6
f. Quanto tempo você tem se sentido desanimado e abatido?	1	2	3	4	5	6
g. Quanto tempo você tem se sentido esgotado?	1	2	3	4	5	6
h. Quanto tempo você tem se sentido uma pessoa feliz?	1	2	3	4	5	6
i. Quanto tempo você tem se sentido cansado?	1	2	3	4	5	6

10. Durante as últimas 4 semanas, quanto do seu tempo a sua saúde física ou problemas emocionais interferiram com as suas atividades sociais (como visitar amigos, parentes, etc.)?

(circule uma)

Todo o temp0	A maior parte do tempo	Alguma parte do tempo	Uma pequena parte do tempo	Nenhuma parte do tempo
1	2	3	4	5

11. O quanto verdadeiro ou falso é cada uma das afirmações para você?
(circule uma)

	Definitiva-mente verdadeiro	A maioria das vezes verdadeiro	Não sei	A maioria das vezes falsa	Definitiva-mente falsa
a. Eu costumo adoecer um pouco mais facilmente que as outras pessoas	1	2	3	4	5
b. Eu sou tão saudável quanto qualquer pessoa que eu conheço	1	2	3	4	5
c. Eu acho que a minha saúde vai piorar	1	2	3	4	5
d. Minha saúde é excelente	1	2	3	4	5

International Study of Postoperative Cognitive Dysfunction - ISPOCD

- ***Mini- Exame do Estado Mental (The Mini Mental State Examination* - MMSE).**

  Originalmente desenvolvido por (Folstein et al., 1975) para ser utilizado para realização de rasterio para demência. Inclui perguntas para avaliar as habilidades de orientação espacial e temporal, memória, seguimento de instrução visual, nomeação de objetos, redação de repetição de sentenças, controle mental e cópia de figura, conforme modelo em anexo. Serão utilizados os pontos de corte sugeridos na adaptação para a população brasileira por Bertolucci e colaboradores (1994).

- ***Aprendizagem Verbal Visual (Visual Verbal Learning*).**

  Baseado no teste de aprendizagem verbal de Rey, será utilizado para avaliar a aprendizagem verbal. Será apresentada uma lista de 15 palavras em uma tela de computador para ser memorizada e recordada em três tentativas sucessivas, com evocação tardia após 15 a 25 minutos. Serão avaliados o número de palavras recordadas e o número de erros cometidos (palavras não fornecidas).

- ***Tarefa de Alternância Conceitual (Concept Shifting Task*).**

  Baseada nos testes de trilhas, será fornecida uma folha de papel na qual o sujeito deverá traçar linhas ligando círculos em ordem seqüencial de números (parte A), letras (parte B) ou números e letras alternados (parte C) randomicamente distribuídos e, finalmente, deverá ligar círculos vazios no sentido horário. Serão avaliados o tempo despedido e o número de erros cometidos (seqüência incorreta).

- ***Teste de Palavras Coloridas de Stroop (Stroop Colour Word Test).***

  Serão apresentados cartões nos quais o sujeito ler 40 nomes de cores (parte 1), nomear 40 retângulos coloridos (parte 2) e 40 nomes de cores impressas em outra cor. Serão avaliados o tempo despedido e o número de erros (cores ou palavras incorretas) em cada cartão.

- ***Tarefa de Memória de Reconhecimento (Memory Scanning Task).***

  Será apresentada uma folha de papel com objetos-alvo que deverão ser memorizados e, em seguida, serão apresentados 120 estímulos (20 estímulos-alvo e 100 distratores), distribuídos em dez linhas, nos quais o indivíduo deverá identificar aqueles que estavam entre os estímulos inicialmente apresentados. Serão avaliados o tempo despendido e o número de erros (omissões ou estímulos não apresentados anteriormente).

- ***Codificação Letra-Número (Letter-Digit Coding*).**

  Baseado no subteste Códigos da Escala Wechsler de Inteligência para Adultos (WAIS), será fornecida uma folha de papel com nove letras que correspondem a nove letras, respectivamente. E, abaixo, serão apresentadas letras para as quais o sujeito deverá assinalar o número correspondente durante um minuto. Será avaliado o número de associações corretas entre letra e número realizadas.

- ***Teste das Quatro Caixas (Four Boxes Test).***

  Este teste avaliará o tempo de reação para escolha entre quatro estímulos. Serão apresentados quatro caixas em uma tela de computador nas quais 52 círculos pretos aparecerão randomicamente a cada 500ms. O sujeito deverá pressionar o botão correspondente à localização de cada círculo o mais rápido possível. Serão avaliados a média de respostas corretas e o número de erros cometidos.

Entrevista Telefônica para o Status Cognitivo (Telephone Interview for Cognitive Status - TICS)

O TICS é um teste padronizado para avaliação do funcionamento neuropsicológico que foi desenvolvido para utilizar em situações onde a avaliação de rastreio cognitivo realizada pessoalmente é impraticável ou ineficiente, como, por exemplo, em pesquisas epidemiológicas de grandes populações ou com pacientes que estão incapacitados de comparecerem ao retorno clínico. É muito útil também para avaliar pessoas com incapacidade para ler ou escrever, pois pode ser aplicado pessoalmente através de entrevista, exigindo apenas capacidade de compreensão verbal. Segundo os fabricantes, tem alta correlação com o MMSE, com excelente sensibilidade e especificidade para detectar comprometimento cognitivo, inclusive em idosos.

Antes de ser administrado via telefone, o entrevistador deverá conversar com algum familiar ou cuidador para se assegurar de que o ambiente estará adequado à entrevista e de que o sujeito estará em boas condições de ouvir e compreender a linguagem verbal.

O teste consiste em um roteiro de entrevista com onze itens abordando as habilidades de orientação espacial e temporal controle mental, memória, informação geral, repetição de sentenças, memória semântica, praxias e antônimos de palavras.

Serão avaliados os acertos em cada item com escores para a somatória total, utilizando-se ponto de corte como parâmetro de comparação entre as entrevistas realizadas nas fases 2 e 4 descritas no procedimento.

MINI EXAME DO ESTADO MENTAL - MEEM - Versão Brasileira

Questões Pontos

1. Qual é: Ano? Estação (Metade do ano)? Data? Dia? Mês? 5
2. Onde estamos: Estado? País? Cidade? Bairro ou hospital? Andar? 5
3. Nomeie três objetos (carro, vaso, janela) levando 1 segundo para cada. Depois, peça ao paciente que os repita para você. Repita as respostas até o indivíduo aprender as 3 palavras (5 tentativas). 3
4. 7s seriados: Subtraia 7 de 100. Subtraia 7 desse número, etc. 5
Interrompa após 5 respostas. Alternativa: Soletre "MUNDO" de trás para frente. 5.
5. Peça ao paciente que nomeie os 3 objetos aprendidos em 3. 3
6. Mostre uma caneta e um relógio. Peça ao paciente que os nomeie conforme você os mostra. 2
7. Peça ao paciente que repita "nem aqui, nem ali, nem lá". 1
8 Peça ao paciente que obedeça sua instrução: "Pegue o papel com sua mão direita. Dobre-o ao meio com as duas mãos. Coloque o papel no chão". 3
9. Peça ao paciente para ler e obedecer o seguinte: "Feche os olhos". 1
10. Peça ao paciente que escreva uma frase de sua escolha. 1
11. Peça ao paciente que copie o seguinte desenho: 1

Escore total: (máximo de 30) ______

HOSPITAL DAS CLÍNICAS
DA FACULDADE DE MEDICINA
DA UNIVERSIDADE DE SÃO PAULO

Diretoria Clínica

Comissão de Ética para Análise de Projetos de Pesquisa - CAPPesq.

PARECER

PROTOCOLO DE PESQUISA Nº: 998/06	Data de entrada: 29/09/06<br>Data sessão: 29 MAR. 2007

TÍTULO DA PESQUISA: Disfunção cognitiva após cirurgia sob anestesia geral

PESQUISADOR(A) RESPONSÁVEL: Maria José Carvalho Carmona

DEPARTAMENTO: Cirurgia

CONSIDERAÇÕES DO RELATOR APROVADAS PELO PLENÁRIO:

Trata-se de um protocolo de pesquisa prospectivo, unicêntrico que objetiva determinar a incidência de disfunção cognitiva no pós-operatório, comparando dexametasona versus placebo e investigar a associação entre alterações cognitivas no pós-operatório e a presença do alelo E4 do gene da polipoproteina E e de marcadores bioquímicos séricos para lesão neuronal, como a proteína S100B e a enolase específica do neurônio (NSE).

Serão estudados 150 pacientes acima de 60 anos, de ambos os sexos submetidos a anestesia geral para herniorrafia inguinal, operação de Nissen ou colecistectomia sob anestesia geral com permanência hospitalar de até 24 horas.

Apesar das solicitações realizadas, o projeto reenviado traz as mesmas pendências:

- explicitar que os pacientes primeiramente participarão de uma bateria de testes com a finalidade de descobrir se estão aptos ao estudo, para posteriormente formalizarem a anuência através da assinatura do TCLE.
- a introdução apresentada não fundamenta porque o estudo deve ser realizado apenas com idosos (considerada população vulnerável) e placebo.

Considerando que o estudo é unicêntrico solicito anexar ao protocolo a carta de anuência dos Laboratórios de Biologia Molecular do InCor e Neurociências da UFRGS onde as amostras serão processadas. Explicitar se haverá envio de amostras ao exterior.

O TCLE está bem redigido, entretanto há necessidade de:

- alterar o termo "via venosa" por "pela veia"
- explicitar que o paciente deverá comparecer ao Hospital para responder os questionários no 3º, 7º, 21º, e 90º dia pós-operatório.
- explicitar que além do questionário e da coleta de sangue, será utilizado um sensor aplicado na testa.
- corrigir os erros de digitação no ITEM IV – retirar a palavra "por" e acrescentar r em tiver

Favor esclarecer se a aquisição dos eletrodos para monitorização do BIS será via CNPQ ou FAPESP e/ou Duke University Medical Center.

Comissão de Ética para Análise de Projetos de Pesquisa do HCFMUSP e da FMUSP
Diretoria Clínica do Hospital das Clínicas da Faculdade de Medicina da Universidade de São Paulo
Rua Ovídio Pires de Campos. 225, 5º andar - CEP 05430 010 - São Paulo - SP
Fone: 011 - 30696442 fax : 011 - 3069 6492 - e-mail : cappesq@hcnet.usp.br / secretariacappesq@hcnet.usp.br

HOSPITAL DAS CLÍNICAS
DA FACULDADE DE MEDICINA
DA UNIVERSIDADE DE SÃO PAULO

APROVAR	☐ REPROVAR
X DEVOLVER PARA ATENDER AS CONSIDERAÇÕES	☐ CIENTE

TEMÁTICA ESPECIAL:	X SIM	☐ NÃO
CONEP: Participação estrangeira	X SIM	☐ NÃO
SVS (SECRETARIA DE VIGILÂNCIA SANITÁRIA):	☐ SIM	☐ NÃO

As respostas às pendências, deverão ser apresentadas no prazo de 30 dias.

PROF. DR. EDUARDO MASSAD
Presidente
CAPPesq-HCFMUSP

São Paulo, 18 de Maio de 2007

Ilmo. Sr.
Prof. Dr. Eduardo Mossad
D.D. Presidente de Ética para Análise de Projetos de Pesquisa
HCFMUSP

Ref: Projeto de Pesquisa nº 998/06: **Disfunção Cognitiva após cirurgia sob anestesia geral.**

Prezado Professor

Agradecemos a análise da CAPPesq ao projeto de pesquisa e estamos reencaminhando e mesmo, assim como o TCLE com as devidas correções

Enfatizamos as alterações ao projeto já apresentadas anteriormente de que o estudo será unicêntrico no Brasil, com inclusão de 300 pacientes do Instituto Central do HC-FMUSP, com coordenação da Duke University.

Estamos anexando as cartas de anuência dos Laboratórios de Biologia Molecular do InCor e de Neurociências da UFRGS, onde as amostras sangüíneas serão processadas. Não haverá envio de amostras sangüíneas para o exterior.

Agradeço antecipadamente e coloco-me à disposição para esclarecimentos adicionais que se fizerem necessários.

Atenciosamente

RECEBIDO POR [illegible]
DATA 25 MAIO 2007
Comissão de Ética para Análise de Projetos de Pesquisa - CAPPesq - HCFMUSP

Maria José Carvalho Carmona
Pesquisador Gerente

Av. Dr. Enéas de Carvalho Aguiar, 44 - CEP: 05403-900 - São Paulo - SP - Brasil - Fone (011) 3069-5232

UFRGS
Universidade Federal
do Rio Grande do Sul

DEPARTAMENTO ICBS BIOQUÍMICA

À COMISSÃO DE ÉTICA PARA ANÁLISE DE PROJETOS DE PESQUISA HOSPITAL DAS CLÍNICAS DA FACULDADE DE MEDICINA DA UNIVERSIDADE DE SÃO PAULO

Ratificamos a participação do Departamento de Bioquímica da Universidade Federal do Rio Grande do Sul no protocolo de pesquisa intitulado "DISFUNÇÃO COGNITIVA APÓS CIRURGIA SOB ANESTESIA GERAL" a ser realizado nas dependências do Hospital das Clínicas da Faculdade de Medicina da Universidade de São Paulo, sob responsabilidade da Professora Dra Maria José Carvalho Carmona da Disciplina de Anestesiologia da Faculdade de Medicina da Universidade de São Paulo.

Atenciosamente,

PROF. DIOGO SOUZA
DEPARTAMENTO DE BIOQUÍMICA
RAMIRO BARCELOS, 2600 - ANEXO
90035-003 - POA
ICBS - UFRGS

Prof Dr Diogo O. Souza, MD, PhD

Departamento de Bioquímica - Instituto de Ciências Básicas da Saúde (ICBS)

Universidade Federal do Rio Grande do Sul

Avenida Ramiro Barcelos, 2600-Anexo

CEP 90035-003

Porto Alegre - RS - Brasil

Fone: (51) 33085557 / 33085558

Fax: (51) 33085540 / 33085535

InCor-HC Zerbini
Ciência e Humanismo

HC Hospital das Clínicas

Medicina USP

À COMISSÃO DE ÉTICA PARA ANÁLISE DE PROJETOS DE PESQUISAS
HOSPITAL DAS CLÍNICAS DA FACULDADE DE MEDICINA DA UNIVERSIDADE DE SÃO PAULO

Ratificamos a participação do Laboratório de Biologia Molecular do Instituto do Coração do Hospital das Clínicas da Faculdade de Medicina da Universidade de São Paulo no protocolo de pesquisa intitulado “DISFUNÇÃO COGNITIVA APÓS CIRURGIA SOB ANESTESIA GERAL” a ser realizado nas dependências do Hospital das Clínicas da Faculdade de Medicina da Universidade de São Paulo, sob a responsabilidade da Professora Dra. Maria José Carvalho Carmona, da Disciplina de Anestesiologia da Faculdade de Medicina da Universidade de São Paulo.

Atenciosamente,

Dr. Alexandre da Costa Pereira

Médico-assistente do Laboratório de Biologia Molecular do Instituto do Coração (INCOR)

Instituto do Coração do Hospital das Clínicas da Faculdade de Medicina da Universidade de São Paulo

Av. Dr. Enéias de Carvalho Aguiar, 44 – 10º andar

CEP: 05403-000

São Paulo – SP

Fone: (11) 3069-5929

HOSPITAL DAS CLÍNICAS
DA
FACULDADE DE MEDICINA DA UNIVERSIDADE DE SÃO PAULO

TERMO DE CONSENTIMENTO LIVRE E ESCLARECIDO
(Instruções para preenchimento no verso)

I - DADOS DE IDENTIFICAÇÃO DO SUJEITO DA PESQUISA OU RESPONSÁVEL LEGAL

1. NOME DO PACIENTE: ....
DOCUMENTO DE IDENTIDADE Nº: ....SEXO: M Ž F Ž
DATA NASCIMENTO: ....../....../......
ENDEREÇO:.. Nº .... APTO: ....
BAIRRO: ....CIDADE ....
CEP:.. TELEFONE: DDD (............) ....

2.RESPONSÁVEL LEGAL ....
NATUREZA (grau de parentesco, tutor, curador etc.) ....
DOCUMENTO DE IDENTIDADE :...SEXO: M F
DATA NASCIMENTO: ....../......./......
ENDEREÇO: .... Nº .... APTO: ....
BAIRRO:... CIDADE: ....
CEP: .... TELEFONE: DDD (............)..

II - DADOS SOBRE A PESQUISA CIENTÍFICA

1. TÍTULO DO PROTOCOLO DE PESQUISA

DISFUNÇÃO COGNITIVA APÓS CIRURGIA SOB ANESTESIA GERAL

PESQUISADOR: Maria José Carvalho Carmona.
CARGO/FUNÇÃO: Professora Associada.... INSCRIÇÃO CONSELHO REGIONAL Nº ...54.142
UNIDADE DO HCFMUSP: .Divisão de Anestesia do Instituto Central

3. AVALIAÇÃO DO RISCO DA PESQUISA:

SEM RISCO ☐ RISCO MÍNIMO X RISCO MÉDIO ☐

RISCO BAIXO ☐ RISCO MAIOR ☐

(probabilidade de que o indivíduo sofra algum dano como consequência imediata ou tardia do estudo)

4.DURAÇÃO DA PESQUISA : .2 anos.

III - REGISTRO DAS EXPLICAÇÕES DO PESQUISADOR AO PACIENTE OU SEU REPRESENTANTE LEGAL SOBRE A PESQUISA:

O senhor (a) está sendo convidado(a) a participar de uma pesquisa, coordenada por um profissional da saúde agora denominado pesquisador. Para poder participar é necessário que o senhor(a) leia este documento com atenção. Ele pode conter palavras que o senhor(a) não entenda. Por favor peça aos responsáveis pelo estudo para explicar qualquer palavra ou procedimentos que o senhor(a) não entenda claramente.

O propósito deste documento é dar o senhor(a) as informações sobre a pesquisa e, se assinado, dará a sua permissão para participar do estudo. O documento descreve o objetivo, procedimentos, benefícios e eventuais riscos ou desconfortos caso queira participar. O senhor(a) só deve participar do estudo se quiser e pode se recusar a participar ou se retirar deste estudo a qualquer momento.

O objetivo desse estudo é determinar a incidência de alterações da função mental, especialmente da memória, após a cirurgia, comparando o uso de um corticóide, a dexametasona e placebo (soro fisiológico). O uso de corticóide antes da cirurgia pode diminuir a dor pós-operatória, além de diminuir a fadiga, a ocorrência de náuseas e vômitos, o período de recuperação e tempo de retorno às atividades diárias. Por outro lado, a dexametasona tem riscos de efeitos colaterais cardiovasculares, ósseos, retenção de líquidos, aumento de gorduras e glicose no sangue, mas como será usada em dose baixa e única não é de esperar a ocorrência de qualquer efeito colateral. Antes da cirurgia o senhor(a) irá receber dexametasona ou placebo (soro fisiológico), aplicados na veia.

Participarão do estudo pessoas acima dos 60 anos que serão submetidas a cirurgia sob anestesia geral e que não tenham alteração da função mental e que não estejam usando cortisona. O senhor já foi anteriormente contactado por via telefônica e respondeu algumas perguntas que indicaram que o senhor(a) pode participar desta pesquisa. Se o senhor concordar em participar deste estudo, um psicólogo lhe aplicará alguns testes que o senhor responderá verbalmente no dia anterior à cirurgia, na noite do dia da cirurgia e no 3°, 7°, 21° e 90° dia após a cirurgia. Nestas datas, o senhor será convidado a comparecer ao hospital para a realização dos testes ou os mesmos serão aplicados por via telefônica.

O segundo objetivo desta pesquisa é a investigação da possível associação entre alterações mentais no pós-operatório e a presença de uma tendência hereditária para este fato, avaliada pela presença de proteínas específicas no sangue. Para tal haverá necessidade da coleta de uma quantidade de 6 mL de sangue que será dividido em 2 amostras e cada tubo com sangue será encaminhado a um laboratório para realização de exames específicos.

O senhor(a) não terá qualquer risco cirúrgico adicional por participar desta pesquisa. A coleta das duas amostras de 3mL de sangue será feita no momento da punção da veia para administração da dexametasona ou placebo antes da cirurgia. Esta mesma veia será utilizada durante a cirurgia para que o senhor receba anestésicos e soro. Durante a cirurgia, os controles feitos pelo anestesista incluirão a utilização de um aparelho já bastante utilizado em anestesia chamado monitor de índice bispectral (BIS) e que utiliza 2 eletrodos conectados na testa para determinação da profundidade da anestesia.

É através das pesquisas clínicas que ocorrem os avanços na medicina, e sua participação é de fundamental importância. Esta pesquisa não irá trazer benefícios diretos para o senhor(a), mas ela irá nos ajudar a melhor entender a alteração da função mental após cirurgia.

IV - ESCLARECIMENTOS DADOS PELO PESQUISADOR SOBRE GARANTIAS DO SUJEITO DA PESQUISA :

A sua participação neste estudo é voluntária. O senhor(a) tem a liberdade de recusar participar do estudo, ou se aceitar participar, retirar seu consentimento a qualquer momento. Este fato não implicará na interrupção de seu atendimento e tratamento, os quais estão assegurados.

Pela sua participação no estudo, o senhor não receberá qualquer valor em dinheiro ou terá qualquer custo. Em caso de eventuais danos à saúde decorrentes desta pesquisa, o senhor terá disponibilidade de assistência neste hospital.

As informações relacionadas ao estudo poderão ser inspecionadas pelos médicos que executam a pesquisa e pelas autoridades legais. No entanto, se qualquer informação for divulgada em relatório ou publicação, isto será feito sob forma codificada sem nomes. Esta medida assegura sua confidencialidade. Quando os resultados forem publicados, não aparecerá seu nome, e sim um código. O senhor(a) tem direito de acesso aos seus dados. O senhor(a) pode discutir esta questão mais adiante com seu médico do estudo. Se o senhor(a) ou seus parentes tiver(em) alguma dúvida com relação ao estudo, direitos do paciente, ou no caso de danos relacionados ao estudo, o senhor(a) deve contactar o pesquisador ou sua equipe .Se o senhor(a) tiver dúvidas sobre seus direitos como um paciente de pesquisa, você pode contactar o Comitê de Ética em Pesquisa em Seres Humanos do Hospital (CEP). A CEP trata-se de um grupo de indivíduos com conhecimento científicos e não científicos que realizam a revisão ética inicial e continuada do estudo de pesquisa para mantê-lo seguro e proteger seus direitos.

V. INFORMAÇÕES DE NOMES, ENDEREÇOS E TELEFONES DOS RESPONSÁVEIS PELO ACOMPANHAMENTO DA PESQUISA, PARA CONTATO EM CASO DE INTERCORRÊNCIAS CLÍNICAS E REAÇÕES ADVERSAS.

Dra Maria José Carvalho Carmona

Divisão de Anestesia do ICHC-FMUSP

Av Enéas Carvalho de Aguiar, n 255 – São Paulo

Fone: 11 3069-5012 ou 3069-6335

VI. OBSERVAÇÕES COMPLEMENTARES:

VII - CONSENTIMENTO PÓS-ESCLARECIDO

Declaro que, após convenientemente esclarecido pelo pesquisador e ter entendido o que me foi explicado, consinto em participar do presente Protocolo de Pesquisa.

São Paulo, de de .

assinatura do sujeito da pesquisa ou responsável legal

assinatura do pesquisador
(carimbo ou nome Legível)

DISCIPLINA DE ANESTESIOLOGIA – FMUSP
INSTITUTO CENTRAL DO HCFMUSP

Protocolo de Pesquisa

DISFUNÇÃO COGNITIVA APÓS CIRURGIA SOB ANESTESIA GERAL

Pesquisadores:
Profa. Dra. Maria José Carvalho Carmona
Dra. Kátia Osternack Pinto
Dr. Rólisson G. B. Lellis
Dr. Luiz Marcelo Sá Malbouisson
Prof. Dr. José Otávio Costa Auler Jr.

2007

DISFUNÇÃO COGNITIVA APÓS CIRURGIA SOB ANESTESIA GERAL

RESUMO

Alguns dos determinantes de disfunção cognitiva pós-operatória são: o uso de opióides no período pós-operatório, falta de atividade física, fadiga pós-operatória, a dor pós-operatória, a qualidade de vida, além de possível predisposição genética. Os pacientes idosos são mais vulneráveis à ocorrência de alterações cognitivas pós-operatórias. O uso de corticóide pré-operatório diminui a dor pós-operatória, a necessidade de opióides, além de diminuir a fadiga, a incidência de náusea e vômito, o período de recuperação e o tempo de retorno às atividades diárias no período pós-operatório, sendo freqüentemente utilizado como coadjuvante em anestesia. Os objetivos deste estudo são: 1) determinar a incidência de disfunção cognitiva no pós-operatório comparando dexametasona *versus* placebo em 300 pacientes acima de 60 anos submetidos à cirurgia sob anestesia geral; 2) Investigar a associação entre alterações cognitivas no pós-operatório e a presença do alelo ε4 do gene da apolipoproteína e de marcadores bioquímicos séricos para lesão neuronal, como a proteína S100β e a enolase específica do neurônio (NSE). Serão aplicados testes neuropsicológicos para avaliar o estado mental geral, aprendizagem, atenção, percepção visuoespacial, memória imediata, operacional e de evocação e habilidades executivas, incluindo velocidade de processamento. Esta bateria define o índice cognitivo da estabilidade (medida longitudinal do status cognitivo em avaliações repetidas que detectam mudanças estatisticamente significantes no funcionamento do sistema nervoso central). Esta avaliação será realiza antes da cirurgia, na noite após a cirurgia e nos dias pós-operatórios 3, 7, 21, e 90. A anestesia geral seguirá os padrões gerais incluindo Propofol (2-3 mg/kg), rocuronio (0.5 mg/kg), e fentanil (1-2 μg/kg) para indução. A anestesia subseqüente e mantida com sevoflurano (1.0-2.5%) e N_2O em 40% de oxigênio, rocuronio (doses suplementares serão administradas se necessário para manutenção de anestesia cirúrgica), e fentanil (doses suplementares 0.5-10 μg/kg com dose máxima de 250 μg). A administração de sevoflurano será ajustada com o objetivo de manter os níveis de hipnose avaliada pelo índice bispectral (BIS) de acordo com os grupos do estudo: os participantes serão randomizados em 2 grupos: anestesia superficial (índice bispectral entre 45 e 55) e anestesia profunda (índice bispectral entre 35 e 45). Ambos níveis são aceitáveis para anestesia geral e são atualmente usados por anestesiologistas. Após venóclise periférica, antes da indução anestésica, será colhida uma amostra de sangue para determinação do perfil genotípico da Apoε e dosagem de S100β e NSE. Os resultados dos escores dos testes em cada grupo serão comparados através do teste de Friedmann, e entre os grupos será utilizado o teste de Kruskal Wallis. Porcentagens serão comparadas pelo do teste de Qui-Quadrado. Os dados serão expressos como mediana e intervalo de confiança de 95%. Serão considerados significativos resultados cujos valores p sejam menores que 0,05 nos testes estatísticos.

DISFUNÇÃO COGNITIVA APÓS CIRURGIA SOB ANESTESIA GERAL

1 – INTRODUÇÃO

Disfunção cognitiva no pós-operatório imediato de cirurgias sob anestesia geral é evento descrito com freqüência crescente, especialmente em pacientes geriátricos[1-5]. A idade é o principal fator de risco para disfunção cognitiva pós-operatória (COPD) e pacientes idosos submetidos a grandes cirurgias devem ser adequadamente avaliados quando referem alterações cognitivas, especialmente da memória, no período pós-operatório[4]. As alterações cerebrais próprias da idade, a presença de aterosclerose, fatores genéticos, alterações na metabolização de fármacos, a resposta inflamatória sistêmica relacionada à cirurgia, dentre outros fatores podem estar implicados na fisiopatologia da COPD[4]. Estudos mostram que alguns dos determinantes de disfunção cognitiva pós-operatória são: o uso de opióides no período pós-operatório, falta de atividade física, fadiga pós-operatória, dor pós-operatória e qualidade de vida[4, 6, 7]. A ocorrência de COPD pode comprometer o resultado cirúrgico, aumentar a morbimortalidade e piorar a qualidade de vida pós-operatória da população geriátrica, daí a importância do estudo de agentes e técnicas que possam minimizar esta complicação.

Na maioria dos casos a disfunção cognitiva pós-operatória é reversível, podendo ser permanente em menos de 1% dos pacientes. Procedimentos sob anestesia regional também podem causar, com menor freqüência, disfunção cognitiva transitória no pós-operatório imediato[4, 8, 9]. A predisposição genética tem sido aventada como possível fator de risco para COPD[10].

O uso de corticóide pré-operatório diminui a dor pós-operatória e também a necessidade de opióides. Diminui fadiga, náusea e vômito, período de recuperação e tempo de retorno às atividades diárias no período pós-operatório. Todos estes fatores melhoram qualidade de vida. Se o corticóide modifica positivamente fatores que estão ligados à disfunção cognitiva como dor, uso de opióides, fadiga, recuperação pós-operatória, convalescença, qualidade de vida e mais rápido retorno às atividades físicas, ele também modificaria positivamente a função cognitiva no período pós-operatório.

Considerando que os pacientes idosos constituem o grupo de maior risco para apresentação de COPD, os objetivos deste estudo são:

1) Determinar a incidência de disfunção cognitiva no pós-operatório, comparando dexametasona *versus* placebo em 300 pacientes acima de 60 anos, submetidos à cirurgia sob anestesia geral;
2) Investigar a associação entre alterações cognitivas no pós-operatório e a presença do alelo ε4 do gene da apolipoproteína E e de marcadores bioquímicos séricos para lesão neuronal, como a proteína S100β e a enolase específica do neurônio (NSE).

3 – CASUÍSTICA E MÉTODOS

Após aprovação pela CAPPesq e obtenção de auxílio financeiro para o estudo, serão estudados 300 pacientes acima de 60 anos, de ambos os sexos e com indicação de anestesia geral para procedimentos cirúrgicos gerais sem utilização de circulação extracorpórea.

Serão aplicados testes neuropsicológicos para avaliar o estado mental geral, aprendizagem, atenção, percepção visuoespacial, memória imediata, operacional e de evocação e habilidades executivas, incluindo velocidade de processamento. Esta bateria define o índice cognitivo da estabilidade (medida longitudinal do status cognitivo em avaliações repetidas que detectam mudanças estatisticamente significantes no funcionamento do sistema nervoso central). A avaliação será realiza antes da cirurgia, na noite após a cirurgia e nos dias pós-operatórios 3, 7, 21, e 90.

Critérios de inclusão: paciente acima de 60 anos, com programação cirúrgica para videocirurgia para herniorrafia inguinal, operação de Nissen ou colecistectomia sob anestesia geral com programação de permanência hospitalar de até 24 horas após a cirurgia.

Critérios de exclusão: idade inferior a 60 anos, história pregressa de doença cerebral ou demência, outras doenças psiquiátricas que afetem a cognição, falta de domínio da língua portuguesa, uso de corticóide ou de opióde.

Os pacientes agendados para cirurgia que preencherem os critérios para participação do estudo serão contactados por via telefônica e convidados a participar do mesmo. Aqueles que concordarem serão submetidos a testes neuropsicológicos específicos para serem utilizados por via telefônica, antes da internação hospitalar e da assinatura do TCLE. Após os testes os pacientes estarão liberados ou não para fazer parte do estudo. Após a internação hospitalar, os pacientes selecionados para participar do estudo serão novamente esclarecidos sobre o mesmo e será solicitada a assinatura do TCLE em duas vias, sendo que uma cópia ficará com o paciente e outra com o pesquisador.

Após obter o consentimento, médico irá aplicar a substância designada para o paciente duas horas antes da indução da anestesia. O grupo ao qual o paciente pertencerá será determinado aleatoriamente, sem o conhecimento do médico que realiza a anestesia ou do psicólogo que aplica os testes, podendo ser:

1. dexametasona, 8 mg IV ou
2. placebo (soro fisiológico), no mesmo volume da solução de dexametasona.

Após venóclise periférica, antes da indução anestésica, serão colhidas duas amostras de sangue de para determinação do perfil genotípico da Apoε e dosagem de S100β e NSE. As amostras serão processadas no Laboratório de Biologia Molecular do InCor (APOε4) e Laboratório de Neurociências da UFRGS (S100β e NSE), sendo que não haverá envio de amostras sangüíneas para o exterior.

A anestesia geral será induzida com utilização dos seguintes fármacos: Propofol (2-3 mg/kg), rocuronio (0.5 mg/kg), e fentanil (1-2 μg/kg) para indução. A anestesia subseqüente será mantida com sevoflurano (1.0-2.5%) e $N_2 0$ em 40% de oxigênio, rocuronio (doses suplementares serão administradas se necessário para manutenção de anestesia cirúrgica), e fentanil (doses suplementares 0.5-10 μg/kg com dose máxima de 250 μg). Variação em requerimentos individuais preclude administração de doses idênticas para cada paciente. Ao invés, a administração de sevoflurano deverá ser ajustada pelo anestesiologista com o objetivo de manter of níveis do BIS (hipnose) de acordo com os grupos do estudo, e fentanil será administrado com o objetivo de manter a pressão arterial e freqüência cardíaca (analgesia) dentro de 20% dos valores pré-indução. A ventilação será controlada mecanicamente para manter a pressão de dióxido de carbono próximo a 35 mmHg. Fentanil (1-2 μg/kg) será dado ao final da cirurgia para melhorar a analgesia durante a recuperação anestésica. Soro fisiológico 5-10 ml/kg/hora será dado durante a cirurgia.

O índice bispectral mede a atividade cerebral cortical (hipnose) numa escala de 0 a 100, onde 100 representa o estado "acordado" e 0 representa um eletroencefalograma isoelétrico. Medidas abaixo de 70 estão relacionadas à inconsciência e ausência de memória necessária para a anestesia geral. Neste estudo, os participantes serão randomizados em 2 grupos: anestesia superficial (índice bispectral entre 45 e 55) and anestesia profunda (índice bispectral entre 35 e 45). Ambos os níveis são aceitáveis para anestesia geral e são atualmente usados por anestesiologistas.

A pele da região onde o monitor vai ser aplicado deve estar limpa e seca. Use álcool e seque esta área da pele.

Aplique o sensor na testa do paciente. Coloque o círculo número 1 no centro aproximadamente 2 cm acima da ponte nasal. Coloque o círculo número 3 na área temporal.

Importante: Aplique pressão em torno do sensor (incluindo as áreas entre os círculos) para garantir a adesão adequada. Em seguida, pressione os círculos 1, 2 e 3 por 5 segundos para assegurar o contato dos eletrodos à pele.

Conecte o sensor aplicado na testa do participante no cabo do índice bispectral.

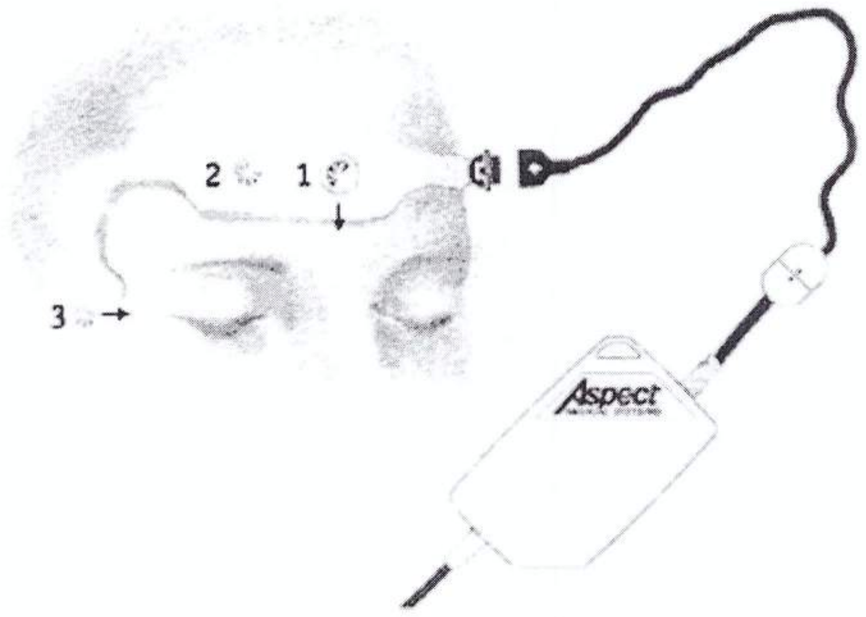

A avaliação da disfunção cognitiva nos dois grupos será realizada através de testes neuropsicológicos, padronizados especificamente para avaliar as funções cognitivas superiores, e questionários para avaliar as características sócio-demográficas, questionamentos sobre a percepção pessoal da saúde do paciente. Estes testes serão aplicados no pré-operatório, na noite anterior à cirurgia, antes da administração de medicações pré-anestésicas ou sedativas, no 3º, 7º, 21º e 90º dia pós-operatório, conforme detalhado abaixo.

Fase 1:

Período: pré e pós-operatório imediatos
Procedimento: aplicar pessoalmente em enfermaria
Instrumento: ISPOCD

Fase 2:

Período: 3º e 7º dias pós-operatórios
Procedimento: aplicar via telefone
Instrumento: TICS

Fase 3:

Período: 21º dia pós-operatório
Procedimento: aplicar pessoalmente em retorno ambulatorial
Instrumento: ISPOCD

Fase 4:

Período: 90º dia pós-operatório
Procedimento: aplicar via telefone
Instrumento: TICS

Os dados serão analisados utilizando-se o software de análise estatística SPSS versão 10. Os dados demográficos como idade, peso, altura serão comparados nos quatro grupos através de análise de

variância univariada seguida de teste de comparações múltiplas se aplicável. As variáveis colhidas através de testes neuropsicológicos aplicados antes e depois do procedimento cirúrgico são de natureza qualitativa ou ordinal, sendo atribuídos resultados numéricos e os resultados finais dos testes serão expressos em escores. Os resultados dos escores dos testes em cada grupo serão comparados através do teste de Friedmann, e entre os grupos será utilizado o teste de Kruskal Wallis. Mann Whitney para comparações da evolução do sujeito Porcentagens serão comparadas através do teste de Qui-Quadrado Os dados serão expressos como mediana e intervalo de confiança de 95%. Serão considerados significativos resultados cujos valores p sejam menores que 0,05 nos testes estatísticos.

INSTRUMENTOS

1- Informações Sócio-demográficas

Serão investigadas as informações sobre idade, sexo, escolaridade, estado civil, ocupação, e demais informações relevantes para compor o perfil sociodemográfico da amostra, bem como a compatibilidade com os critérios de exclusão e inclusão, conforme detalhado em anexo.

2- Versão Brasileira do Questionário de Qualidade de Vida SF-36

O SF-36 investiga condições relacionadas à saúde, atividades de vida diária, produtividade, problemas emocionais, relacionamentos, motivação e outras condições que traçam o perfil da qualidade de vida a partir da percepção do próprio sujeito, conforme apresentado em anexo.

3- International Study of Postoperative Cognitive Dysfunction - ISPOCD

A bateria neuropsicológica proposta neste estudo seguirá o protocolo sugerido pelo Estudo Internacional de Disfunção Cognitiva Pós-Cirúrgica (do inglês, International Study of Postoperative Cognitive Disfunction - ISPOCD) para avaliar as funções de memória, velocidade sensoriomotora, flexibilidade mental e produtividade motora, com a finalidade de detectar disfunções neuropsicológicas sutis que possam ocorrer em pacientes após cirurgia com anestesia geral ou regional (Rasmussen et al., 1999, Rasmussen et al., 2001 e Rasmussen et al., 2002). Para detalhes, ver anexos.

4- Entrevista Telefônica para o Status Cognitivo (Telephone Interview for Cognitive Status - TICS)

Instrumentos padronizados para realização de entrevista telefônica têm se mostrado válidos e sensíveis, sendo muito indicados para utilizar em situações onde a avaliação de rastreio cognitivo realizada pessoalmente é impraticável ou ineficiente, como, por exemplo, em pesquisas epidemiológicas de grandes populações ou com pacientes que estão incapacitados de comparecerem ao retorno clínico (Ferrucci et al., 1998, De Jager et al., 2003, Barber & Stott, 2004, Musselwhite et al., 2006 e Dal Forno et al., 2006).

O TICS - Entrevista Telefônica para o Status Cognitivo é um teste padronizado para avaliação do funcionamento neuropsicológico que foi desenvolvido para utilizar em situações onde a avaliação de rastreio cognitivo realizada pessoalmente é impraticável ou ineficiente, como, por exemplo, em pesquisas epidemiológicas de grandes populações ou com pacientes que estão incapacitados de comparecerem ao retorno clínico. É muito útil também para avaliar pessoas com incapacidade para

ler ou escrever, pois pode ser aplicado pessoalmente através de entrevista, exigindo apenas capacidade de compreensão verbal.
O teste consiste em um roteiro de entrevista com onze itens abordando as habilidades de orientação espacial e temporal controle mental, memória, informação geral, repetição de sentenças, memória semântica, praxias e antônimos de palavras, conforme detalhado em anexo.

4- ANÁLISE DO RISCO

O risco da pesquisa é mínimo, pois não modificará a técnica anestésica ou cirúrgica rotineiramente utilizada. A única intervenção proposta é a utilização de dexametasona em metade da população estudada, fármaco este freqüentemente utilizado em anestesia para prevenção de vômitos pós-operatórios. A monitorização utilizada e a amostra sangüínea colhida não acrescentam risco ao procedimento cirúrgico.

5- CRONOGRAMA

Estimamos um prazo máximo de 24 meses para o estudo, sendo 18 meses para a coleta dos dados, 2 meses para análise estatística completa dos resultados obtidos e 2 a 4 meses para formulação do artigo científico a ser publicado.

6- ORIGEM DOS RECURSOS FINANCEIROS

Após aprovação pela CAPPesq, auxílio à pesquisa em valor aproximado de R$20.000,00 será solicitado ao CNPQ ou FAPESP para compra dos eletrodos para monitorização do índice bispectral (BIS). O equipamento monitor de BIS (índice bispectral) será fornecido pela Duke University.

A bateria de testes neuropsicológicos mais indicados para avaliação da disfunção cognitiva pós-operatória serão adquiridos pela Duke University e fornecidos pela ISPOCD (International Study of Post-Operative Cognitive Dysfuntion).

Será solicitado também auxílio financeiro para realização dos testes neuropsicológicos e aquisição de *laptop* específico para o serviço de neuropsicologia, onde serão armazenados os testes e seus resultados.

7- PROCEDIMENTOS APÓS A REALIZAÇÃO DA PESQUISA

Os dados e resultados obtidos serão armazenados em banco de dados seguro e sigiloso. Após a análise completa dos resultados, serão submetidos à publicação em periódico científico indexado apropriado.

8- REFERÊNCIAS BIBLIOGRÁFICAS

1. Ancelin, M.L., et al., *Exposure to anaesthetic agents, cognitive functioning and depressive symptomatology in the elderly.* Br J Psychiatry, 2001. **178**: p. 360-6.
2. Bekker, A.Y. and E.J. Weeks, *Cognitive function after anaesthesia in the elderly.* Best Pract Res Clin Anaesthesiol, 2003. **17**(2): p. 259-72.
3. Cohendy, R., A. Brougere, and P. Cuvillon, *Anaesthesia in the older patient.* Curr Opin Clin Nutr Metab Care, 2005. **8**(1): p. 17-21.
4. Rasmussen, L.S. and J.T. Moller, *Central nervous system dysfunction after anesthesia in the geriatric patient.* Anesthesiol Clin North America, 2000. **18**(1): p. 59-70, vi.
5. Xie, Z. and R.E. Tanzi, *Alzheimer's disease and post-operative cognitive dysfunction.* Exp Gerontol, 2006. **41**(4): p. 346-359.
6. Benoit, A.G., et al., *Risk factors and prevalence of perioperative cognitive dysfunction in abdominal aneurysm patients.* J Vasc Surg, 2005. **42**(5): p. 884-90.
7. Wu, C.L., et al., *Postoperative cognitive function as an outcome of regional anesthesia and analgesia.* Reg Anesth Pain Med, 2004. **29**(3): p. 257-68.
8. Campbell, D.N., et al., *A prospective randomised study of local versus general anaesthesia for cataract surgery.* Anaesthesia, 1993. **48**(5): p. 422-8.
9. Canet, J., et al., *Cognitive dysfunction after minor surgery in the elderly.* Acta Anaesthesiol Scand, 2003. **47**(10): p. 1204-10.
10. Lelis, R.G., et al., *Apolipoprotein E4 genotype increases the risk of postoperative cognitive dysfunction in patients undergoing coronary artery bypass graft surgery.* J Cardiovasc Surg (Torino), 2006. **47**(4): p. 451-6.

ANEXOS: Instrumentos utilizados no protocolo de pesquisa

Informações Sócio-demográficas

Critérios para inclusão no estudo:

- Pacientes acima de 60 anos
- Submetido à cirurgia de pequeno porte
- Sob anestesia geral

Critérios para exclusão:

1. Idade inferior a 60 anos
2. História pregressa de doença cerebral ou demência
3. Outras doenças psiquiátricas que afetem a cognição
4. Falta de domínio da língua portuguesa
5. Estar fazendo uso de corticóide

- Nome: ____
- Diagnóstico: ____
- Sexo: masculino feminino
- Idade (anos): __________
- Peso (quilos): __________
- Altura (centímetros): __________

Estado civil

( ) solteiro (a)
( ) casado(a)
( ) divorciado(a)
( ) viúvo(a)

Emprego

( ) empregado(a)
( ) desempregado(a)
( ) aposentado(a)

Salário mensal:

( ) 0
( ) 0-1 salário mínimo
( )1-5 salários mínimos
( )6-10 salários mínimos
( )10-15 salários mínimos
( )15-20 salários mínimos
( ) mais que 20 salários mínimos

Escolaridade

( ) Nenhuma
( ) Primário incompleto
( ) Primário completo
( ) Ginásio completo
( ) Colegial completo
( ) Superior completo
(..) Pós-graduação

Versão Brasileira do Questionário de Qualidade de Vida SF-36

Instruções: Esta pesquisa questiona você sobre sua saúde. Estas informações nos manterão informados de como você se sente quão bem você é capaz de fazer suas atividades de vida diária. Responda cada questão marcando a resposta como indicado. Caso você esteja inseguro ou em dúvida em como responder, por favor, tente responder o melhor que puder.

1. Em geral, você diria que a sua saúde é:

(circule uma)

Excelente	Muito boa	Boa	Ruim	Muito ruim
1	2	3	4	5

2. Comparada há um ano atrás, como você classificaria a sua saúde em geral, agora?

(circule uma)

Muito melhor	Um pouco melhor	Quase a mesma	Um pouco pior	Muito pior
1	2	3	4	5

3. Os seguintes itens são sobre atividades que você poderia fazer atualmente durante um dia comum. Devido a sua saúde, você teria dificuldade para fazer essas atividades? Neste caso, quanto?

(circule um número em cada linha)

Atividades	Sim. Dificulta muito	Sim. Dificulta um pouco	Não. Não dificulta de modo algum
A. Atividades vigorosas, que exigem muito esforço, tais como correr, levantar objetos pesados, participar em esportes árduos.	1	2	3
b. Atividades moderadas, tais como mover uma mesa, passar aspirador de pó, jogar bola, varrer a casa.	1	2	3
c. Levantar ou carregar mantimentos	1	2	3
d. Subir vários lances de escada	1	2	3
e. Subir um lance de escadas	1	2	3
f. Curvar-se, ajoelhar-se ou dobrar-se	1	2	3
g. Andar mais de 1 quilômetro	1	2	3
h. Andar vários quarteirões	1	2	3
i. Andar um quarteirão	1	2	3
j. Tomar banho ou vestir-se	1	2	3

4. Durante as últimas 4 semanas, você teve algum dos seguintes problemas com o seu trabalho ou com alguma atividade diária regular, como conseqüência de sua saúde física?

(circule uma em cada linha)

	Sim	Não
a. Você diminuiu a quantidade de tempo que se dedicava ao seu trabalho ou a outras atividades?	1	2
b. Realizou menos tarefas do que você gostaria?	1	2
c. Esteve limitado no seu tipo de trabalho ou em outras atividades?	1	2
d. Teve dificuldade de fazer seu trabalho ou outras atividades (p.ex.: necessitou de um esforço extra)?	1	2

5. Durante as últimas 4 semanas, você teve alguns dos seguintes problemas com o seu trabalho ou outra atividade regular diária, como conseqüência de algum problema emocional (como sentir-se deprimido ou ansioso)?

(circule uma em cada linha)

	Sim	Não
a. Você diminuiu a quantidade de tempo que se dedicava ao seu trabalho ou a outras atividades?	1	2
b. Realizou menos tarefas do que você gostaria?	1	2
c. Não trabalhou ou não fez qualquer das atividades com tanto cuidado como geralmente faz?	1	2

6. Durante as últimas 4 semanas, de que maneira sua saúde física ou problemas emocionais interferiram nas suas atividades sociais normais, em relação a família, vizinhos, amigos ou em grupo?

(circule uma)

De forma nenhuma	Ligeiramente	Moderadamente	Bastante	Extremamente
1	2	3	4	5

7. Quanta dor no corpo você teve durante as últimas 4 semanas?

(circule uma)

Nenhuma	Muito leve	Leve	Moderada	Grave	Muito grave
1	2	3	4	5	6

8. Durante as últimas 4 semanas, quanto a dor interferiu com o seu trabalho normal (incluindo tanto o trabalho fora de casa e dentro de casa)?

(circule uma)

De maneira alguma	Um pouco	Moderadamente	Bastante	Extremamente
1	2	3	4	5

9. Estas questões são sobre como você se sente e como tudo tem acontecido com você durante as últimas 4 semanas. Para cada questão, por favor dê uma resposta que mais se aproxime da maneira como você se sente. Em relação às últimas 4 semanas.
(circule um número para cada linha)

	Todo tempo	A maior parte do tempo	Uma boa parte do tempo	Alguma parte do tempo	Uma pequena parte do tempo	Nunca
a. Quanto tempo você tem se sentido cheio de vigor, cheio de vontade, cheio de força?	1	2	3	4	5	6
b. Quanto tempo você tem se sentido uma pessoa muito nervosa?	1	2	3	4	5	6
c. Quanto tempo você tem se sentido tão deprimido que nada pode animá-lo?	1	2	3	4	5	6
d. Quanto tempo você tem se sentido calmo e tranqüilo?	1	2	3	4	5	6
e. Quanto tempo você tem se sentido com muita energia?	1	2	3	4	5	6
f. Quanto tempo você tem se sentido desanimado e abatido?	1	2	3	4	5	6
g. Quanto tempo você tem se sentido esgotado?	1	2	3	4	5	6
h. Quanto tempo você tem se sentido uma pessoa feliz?	1	2	3	4	5	6
i. Quanto tempo você tem se sentido cansado?	1	2	3	4	5	6

10. Durante as últimas 4 semanas, quanto do seu tempo a sua saúde física ou problemas emocionais interferiram com as suas atividades sociais (como visitar amigos, parentes, etc.)?
(circule uma)

Todo o temp0	A maior parte do tempo	Alguma parte do tempo	Uma pequena parte do tempo	Nenhuma parte do tempo
1	2	3	4	5

11. O quanto verdadeiro ou falso é cada uma das afirmações para você? (circule uma)

	Definitiva-mente verdadeiro	A maioria das vezes verdadeiro	Não sei	A maioria das vezes falsa	Definitiva-mente falsa
a. Eu costumo adoecer um pouco mais facilmente que as outras pessoas	1	2	3	4	5
b. Eu sou tão saudável quanto qualquer pessoa que eu conheço	1	2	3	4	5
c. Eu acho que a minha saúde vai piorar	1	2	3	4	5
d. Minha saúde é excelente	1	2	3	4	5

International Study of Postoperative Cognitive Dysfunction - ISPOCD

- ***Mini- Exame do Estado Mental (The Mini Mental State Examination* - MMSE).**

Originalmente desenvolvido por (Folstein et al., 1975) para ser utilizado para realização de rasterio para demência. Inclui perguntas para avaliar as habilidades de orientação espacial e temporal, memória, seguimento de instrução visual, nomeação de objetos, redação de repetição de sentenças, controle mental e cópia de figura, conforme modelo em anexo. Serão utilizados os pontos de corte sugeridos na adaptação para a população brasileira por Bertolucci e colaboradores (1994).

- ***Aprendizagem Verbal Visual (Visual Verbal Learnin*g).**

Baseado no teste de aprendizagem verbal de Rey, será utilizado para avaliar a aprendizagem verbal. Será apresentada uma lista de 15 palavras em uma tela de computador para ser memorizada e recordada em três tentativas sucessivas, com evocação tardia após 15 a 25 minutos. Serão avaliados o número de palavras recordadas e o número de erros cometidos (palavras não fornecidas).

- ***Tarefa de Alternância Conceitual (Concept Shifting Tas*k).**

Baseada nos testes de trilhas, será fornecida uma folha de papel na qual o sujeito deverá traçar linhas ligando círculos em ordem seqüencial de números (parte A), letras (parte B) ou números e letras alternados (parte C) randomicamente distribuídos e, finalmente, deverá ligar círculos vazios no sentido horário. Serão avaliados o tempo despedido e o número de erros cometidos (seqüência incorreta).

- ***Teste de Palavras Coloridas de Stroop (Stroop Colour Word Test).***

Serão apresentados cartões nos quais o sujeito ler 40 nomes de cores (parte 1), nomear 40 retângulos coloridos (parte 2) e 40 nomes de cores impressas em outra cor. Serão avaliados o tempo despedido e o número de erros (cores ou palavras incorretas) em cada cartão.

- ***Tarefa de Memória de Reconhecimento (Memory Scanning Task).***

Será apresentada uma folha de papel com objetos-alvo que deverão ser memorizados e, em seguida, serão apresentados 120 estímulos (20 estímulos-alvo e 100 distratores), distribuídos em dez linhas, nos quais o indivíduo deverá identificar aqueles que estavam entre os estímulos inicialmente apresentados. Serão avaliados o tempo despendido e o número de erros (omissões ou estímulos não apresentados anteriormente).

- ***Codificação Letra-Número (Letter-Digit Codin*g).**

Baseado no subteste Códigos da Escala Wechsler de Inteligência para Adultos (WAIS), será fornecida uma folha de papel com nove letras que correspondem a nove letras, respectivamente. E, abaixo, serão apresentadas letras para as quais o sujeito deverá assinalar o número correspondente durante um minuto. Será avaliado o número de associações corretas entre letra e número realizadas.

- ***Teste das Quatro Caixas (Four Boxes Test).***

Este teste avaliará o tempo de reação para escolha entre quatro estímulos. Serão apresentados quatro caixas em uma tela de computador nas quais 52 círculos pretos aparecerão randomicamente a cada 500ms. O sujeito deverá pressionar o botão correspondente à localização de cada círculo o mais rápido possível. Serão avaliados a média de respostas corretas e o número de erros cometidos.

Entrevista Telefônica para o Status Cognitivo (Telephone Interview for Cognitive Status - TICS)

O TICS é um teste padronizado para avaliação do funcionamento neuropsicológico que foi desenvolvido para utilizar em situações onde a avaliação de rastreio cognitivo realizada pessoalmente é impraticável ou ineficiente, como, por exemplo, em pesquisas epidemiológicas de grandes populações ou com pacientes que estão incapacitados de comparecerem ao retorno clínico. É muito útil também para avaliar pessoas com incapacidade para ler ou escrever, pois pode ser aplicado pessoalmente através de entrevista, exigindo apenas capacidade de compreensão verbal. Segundo os fabricantes, tem alta correlação com o MMSE, com excelente sensibilidade e especificidade para detectar comprometimento cognitivo, inclusive em idosos.

Antes de ser administrado via telefone, o entrevistador deverá conversar com algum familiar ou cuidador para se assegurar de que o ambiente estará adequado à entrevista e de que o sujeito estará em boas condições de ouvir e compreender a linguagem verbal.

O teste consiste em um roteiro de entrevista com onze itens abordando as habilidades de orientação espacial e temporal controle mental, memória, informação geral, repetição de sentenças, memória semântica, praxias e antônimos de palavras.

Serão avaliados os acertos em cada item com escores para a somatória total, utilizando-se ponto de corte como parâmetro de comparação entre as entrevistas realizadas nas fases 2 e 4 descritas no procedimento.

MINI EXAME DO ESTADO MENTAL - MEEM - Versão Brasileira

Questões Pontos

1. Qual é: Ano? Estação (Metade do ano)? Data? Dia? Mês? 5

2. Onde estamos: Estado? País? Cidade? Bairro ou hospital? Andar? 5

3. Nomeie três objetos (carro, vaso, janela) levando 1 segundo para cada. Depois, peça ao paciente que os repita para você. Repita as respostas até o indivíduo aprender as 3 palavras (5 tentativas). 3

4. 7s seriados: Subtraia 7 de 100. Subtraia 7 desse número, etc. 5

Interrompa após 5 respostas. Alternativa: Soletre "MUNDO" de trás para frente. 5.

5. Peça ao paciente que nomeie os 3 objetos aprendidos em 3. 3

6. Mostre uma caneta e um relógio. Peça ao paciente que os nomeie conforme você os mostra. 2

7. Peça ao paciente que repita "nem aqui, nem ali, nem lá". 1

8 Peça ao paciente que obedeça sua instrução: "Pegue o papel com sua mão direita. Dobre-o ao meio com as duas mãos. Coloque o papel no chão". 3

9. Peça ao paciente para ler e obedecer o seguinte: "Feche os olhos". 1

10. Peça ao paciente que escreva uma frase de sua escolha. 1

11. Peça ao paciente que copie o seguinte desenho: 1

Escore total: (máximo de 30) ______

FINANCIAMENTO DA PESQUISA

As fontes de financiamento da pesquisa serão:

1. Duke University:
   - Forneciemento do monitor e dos eletrodos para avaliação do índice bispectral.
   - compra dos testes neuropsicológicos específicos para o estudo
   - Auxílio para análise estatística e custos de publicação
2. FAPESP ou CNPQ: após aprovação do projeto pela CAPPesq será solicitado auxílio em valor estimado de R$20.000,00 (vinte mil reais) para:
   - aplicação dos testes neuropsicológicos pelo Serviço de Neuropsicologia
   - de *laptop* específico para aplicação dos testes neuropsicológicos.
   - Custos da pesquisa de APOε4 pelo Laboratório de Biologia Molecular do INCOR
   - Custos da dosagem de S100β e NSE pelo Laboratório de Neurociências da UFRGS.

HC HOSPITAL DAS CLÍNICAS DA FACULDADE DE MEDICINA DA UNIVERSIDADE DE SÃO PAULO

Diretoria Clínica

Comissão de Ética para Análise de Projetos de Pesquisa - CAPPesq.

PARECER

PROTOCOLO DE PESQUISA Nº: 968/06 (9 manuscrito sobre o 6)	Data de entrada: 29.09.06
	Data sessão: 20.06.07

TÍTULO DA PESQUISA: *Disfunção cognitiva após cirurgia sob anestesia geral*

PESQUISADOR(A) RESPONSÁVEL: Maria José Carvalho Carmona

DEPARTAMENTO: Cirurgia

CONSIDERAÇÕES DO RELATOR APROVADAS PELO PLENÁRIO:

Trata-se de um protocolo de pesquisa prospectivo, unicêntrico que objetiva determinar a incidência de disfunção cognitiva no pós-operatório (COPD), comparando dexametasona versus placebo e investigar a associação entre alterações cognitivas no pós-operatório e a presença do alelo E4 do gene da polipoproteina E e de marcadores bioquímicos séricos para lesão neuronal, como a proteína S100B e a enolase específica do neurônio (NSE).

Serão estudados 300 pacientes acima de 60 anos, de ambos os sexos submetidos a anestesia geral para herniorrafia inguinal, operação de Nissen ou colecistectomia sob anestesia geral com permanência hospitalar de até 24 horas. A opção pela população idosa ocorreu pelo fato da idade ser o principal fator de risco para a COPD.

Ainda falta

Justificativa do Placebo

A inscrição na ANVISA do monitor e eletrodos da avaliação do índice bispectral

Esclarecer se os eletrodos serão comprados pela verba FAPESP ou Duke University

Na carta, a Duke University informou apenas o fornecimento dos eletrodos do BIS e não do monitor

Os pacientes serão convidados a participar do estudo pelo telefone a somente se apresentarem os critérios de inclusão e não apresentarem o exclusão assinarão o TCLE.????

- REFAZER PROJETO: - ENTREVISTA - CÂMARA 5 - 20/06/07
  - GRUPO CONTROLE - PROCEDIMENTO PADRÃO HC
  - EQUIPAMENTO COMERCIALIZADO NO BRASIL
  - EXPLICAÇÃO DO PROTOCOLO ANTES DO TESTE - CONSENTIMENTO ORAL

☐ **APROVAR** ☐ **REPROVAR**

☐ **DEVOLVER PARA ATENDER AS CONSIDERAÇÕES** ☐ **CIENTE**

São Paulo, 21 de junho de 2007

Ilmo. Sr.
Prof. Dr. Eduardo Mossad
D.D. Presidente de Ética para Análise de Projetos de Pesquisa
HCFMUSP

Ref: Projeto de Pesquisa nº 998/06: **Disfunção Cognitiva após cirurgia sob anestesia geral.**

Prezado Professor

Agradecemos a análise da CAPPesq ao projeto de pesquisa e estamos reencaminhando e mesmo, assim como o TCLE com as devidas correções

Agradeço antecipadamente e coloco-me à disposição para esclarecimentos adicionais que se fizerem necessários.

Atenciosamente

Maria José Carvalho Carmona
Pesquisador Gerente

RECEBIDO POR
DATA 22/JUN. 2007
Comissão de Ética para Análise de Projetos de Pesquisa - CAPPesq - HCFMUSP

Anexo II

HOSPITAL DAS CLÍNICAS

DA FACULDADE DE MEDICINA DA UNIVERSIDADE DE SÃO PAULO

CAIXA POSTAL, 8091 - SÃO PAULO - BRASIL

DIRETORIA CLÍNICA
COMISSÃO DE ÉTICA PARA ANÁLISE DE PROJETOS DE PESQUISA - CAPPesq

CADASTRO DE PROTOCOLO DE PESQUISA

Registro (uso reservado à Secretaria da CAPPesq)

Nº do Protocolo: .... Data de Entrada: ....

1. Título do Protocolo de Pesquisa

DISFUNÇÃO COGNITIVA APÓS CIRURGIA SOB ANESTESIA GERAL

2. Palavras-chaves que caracterizam o assunto da Pesquisa

Anestesia Geral
Pós-Operatório
Disfunção Cognitiva

3. Resumo do Protocolo de Pesquisa:

Alguns dos determinantes de disfunção cognitiva pós-operatória são: o uso de opióides no período pós-operatório, falta de atividade física, fadiga pós-operatória, a dor pós-operatória, a qualidade de vida, além de possível predisposição genética. Os pacientes idosos são mais vulneráveis à ocorrência de alterações cognitivas pós-operatórias. O uso de corticóide pré-operatório diminui a dor pós-operatória, a necessidade de opióides, além de diminuir a fadiga, a incidência de náusea e vômito, o período de recuperação e o tempo de retorno às atividades diárias no período pós-operatório, sendo freqüentemente utilizado como coadjuvante em anestesia. Os objetivos deste estudo são: 1) avaliar os efeitos da dexametasona sobre a incidência de disfunção cognitiva em pacientes acima de 60 anos submetidos à cirurgia sob anestesia geral; 2) Investigar a associação entre alterações cognitivas no pós-operatório e a presença do alelo ε4 do gene da apolipoproteína e de marcadores bioquímicos séricos para lesão neuronal, como a proteína S100β e a enolase específica do neurônio (NSE). Prevê-se o estudo de 300 pacientes aos quais serão aplicados testes neuropsicológicos para avaliar o estado mental geral, aprendizagem, atenção, percepção visuoespacial, memória imediata, operacional e de evocação e habilidades executivas, incluindo velocidade de processamento. Esta bateria de testes define o índice cognitivo da estabilidade (medida longitudinal do status cognitivo em avaliações repetidas que detectam mudanças estatisticamente significantes no funcionamento do sistema nervoso central). Os pacientes selecionados para o estudo serão contatados por via telefônica no dia anterior ao estudo e, se derem o consentimento verbal, serão submetidos a teste neuropsicológico específico para ser aplicado por via telefônica. Após a internação hospitalar e assinatura do TCLE o paciente será submetido os testes neuropsicológicos específicos e randomizado para participação em um dos dois grupos de estudo, sendo que o psicólogo que aplicará os testes neuropsicológicos não terá conhecimento do grupo ao qual o paciente participa: **Grupo Dexametasona**: 8 mg de dexametasona por via venosa, aplicada duas horas antes do horário marcado para início do procedimento cirúrgico e **Grupo Controle**: realização da técnica anestésica habitual, sem utilização de dexametasona. A anestesia geral seguirá os padrões gerais incluindo Propofol (2-3 mg/kg), rocuronio (0.5 mg/kg), e fentanil (1-2 µg/kg) para indução. A anestesia subseqüente e mantida com sevoflurano (1.0-2.5%) e N_20 em 40% de oxigênio, rocuronio (doses suplementares serão administradas se necessário para manutenção de anestesia cirúrgica), e fentanil (doses suplementares 0.5-10 µg/kg com dose máxima de 250 µg). A administração de sevoflurano será ajustada com o objetivo de manter os níveis de hipnose avaliada pelo índice bispectral (BIS) de acordo com os grupos do estudo: os participantes serão randomizados em 2 grupos: anestesia superficial (índice bispectral entre 45 e 55) e anestesia profunda (índice bispectral entre 35 e 45). Ambos níveis são aceitáveis para anestesia geral e são atualmente usados por anestesiologistas. Após venóclise periférica, antes da indução anestésica, será colhida uma amostra de sangue para determinação do perfil genotípico da Apoε e dosagem de S100β e NSE. Os testes neuropsicológicos serão aplicados antes da cirurgia, na noite após a cirurgia e nos dias pós-operatórios 3, 7, 21, e 90.Os resultados dos escores dos testes em cada grupo serão comparados através do teste de Friedmann, e entre os grupos será utilizado o teste de Kruskal Wallis. Porcentagens serão comparadas pelo do teste de Qui-Quadrado. Os dados serão expressos como mediana e intervalo de confiança de 95%. Serão considerados significativos resultados cujos valores p sejam menores que 0,05 nos testes estatísticos.

4. **Pesquisador Responsável**: Maria José Carvalho Carmona

5. **Pesquisador Executante**: Luiz Marcelo Sá Malbouisson

6. **Orientador**: Maria José Carvalho Carmona

7. **Especificação da finalidade acadêmica da pesquisa**

☐ Graduação ☐ Pós-graduação { ☐Doutorado / ☐ Mestrado }

☐ Outros - especificar: Investigação multicêntrica

8. **Unidades e Instituições envolvidas** (especificar)

- HOSPITAL DAS CLÍNICAS - FMUSP: Divisão de Anestesia do ICHC – Progamação de coleta de dados de 150 pacientes.
- ENTIDADES EXTERNAS:
  - Hospital Nossa Senhora do Carmo – Curitiba-PR - Progamação de coleta de dados de 75 pacientes.
  - Hospital Ministro Costa Cavalcanti da Fundacao Itaiguaipy – Londrina – PR - Progamação de coleta de dados de 75 pacientes.
  - Duke University – Não haverá coleta de dados neste centro, apenas a Coordenação do estudo referente à avaliação neuropsicológica e fornecimento de eletrodos para avaliação do Índice Bispectral.

9. **Pesquisa**:

X seres humanos ☐ animais (espécie): ....

10. **Investigação:**

☐ Retrospectiva X Prospectiva

11. **Materiais e métodos:**

X Laboratorial ☐ Prontuários de pacientes

☐ Peças anatômicas de cadáveres ☐ Tecidos, órgãos, fluídos orgânicos

X Entrevistas e questionários ☐ Outros: ....

12. **A Pesquisa envolve**: (preencher mais de um se necessário)

☐ Isótopo Radioativo, Dispositivo Gerador de Radiação Ionizantes

☐ Microorganismos Patogênicos

☐ Ácidos Nucleares Recombinantes

☐ Outros (especificar) : ....

13. Existe algum risco ambiental e/ou biológico com o descarte dos sub-produtos e/ou reagentes de sua pesquisa?

☐ SIM X NÃO

14. Pesquisa em áreas temáticas especiais:

- ☐ genética humana;
- ☐ reprodução humana;
- ☐ fármacos, medicamentos, vacinas e testes diagnósticos novos (fases I, II e III) ou não registrados no país (ainda que fase IV), ou quando a pesquisa for referente a seu uso com modalidades, indicações, doses ou vias de administração diferentes daquelas estabelecidas, incluindo seu emprego em combinações;
- ☐ equipamentos, insumos e dispositivos para a saúde novos, ou não registrados no país;
- ☐ novos procedimentos ainda não consagrados na literatura;
- ☐ populações indígenas;
- ☐ projetos que envolvam aspectos de biossegurança;
- ☐ pesquisas coordenadas do exterior ou com participação estrangeira
- ☐ pesquisas que envolvam remessa de material biológico para o exterior.

15. Gênero da pesquisa:

☐ Clínica (Fisiopatológico, Terapêutico, Diagnóstico)

X Cirúrgica (Fisiopatológico, Terapêutico, Diagnóstico)

☐ Experimental (Fisiopatológico, Terapêutico, Diagnóstico)

☐ Anatômica

☐ Epidemiológica

☐ Teórica

16. Patrocínio:

Recursos Financeiros Solicitados (Serão solicitados após aprovação CAPPesq)

Instituições	Valores	Instituições	Valores
CNPq	R$20.000,00	Fundo Pesquisa FEJZ.	
FINEP		HC-FMUSP	
CAPES		Industrias:	
FAPESP		Laboratórios:	
F.F.M.		Outros: Duke University	materiais

O valor do recurso financeiro a ser solicitado ao CNPQ ou FAPESP corresponde à aquisição do material necessário para a avaliação da presença da APOε4 e S100β. Os eletrodos para monitorização do índice bispectral (BIS) e os formulários para aplicação dos testes neuropsicológicos serão fornecidos pela Duke University.

17. Existência de infraestrutura e recursos humanos para desenvolvimento da pesquisa (especificar).

A Divisão de Anestesia do Instituto Central do Hospital das Clínicas dispõe de Infra-estrutura e recursos humanos para desenvolvimento desta pesquisa.

18. Cronograma de execução da pesquisa

início: _01_/_09_/__2006__ término: _31_/__08_/_2008_

Prazo: 02 anos

19. Parecer da Comissão de Pesquisa e/ou de Ética do Departamento da FMUSP ou da entidade envolvida.

20. Conselho de Departamento da FMUSP

Assinatura
Carimbo

Aprovado em _____/______/_______

21. Parecer do Serviço de Verificação de Óbitos da Capital-SVOC, no caso de pesquisas realizadas em peças anatômicas de cadáveres necropsiados naquele Serviço.

Assinatura
Carimbo

Aprovado em _____/______/______

/tsc.

Anexo I

HOSPITAL DAS CLÍNICAS
DA
FACULDADE DE MEDICINA DA UNIVERSIDADE DE SÃO PAULO

TERMO DE CONSENTIMENTO LIVRE E ESCLARECIDO

(Instruções para preenchimento no verso)

I - DADOS DE IDENTIFICAÇÃO DO SUJEITO DA PESQUISA OU RESPONSÁVEL LEGAL

1. NOME DO PACIENTE: ....
DOCUMENTO DE IDENTIDADE Nº: ....SEXO: M Ž F Ž
DATA NASCIMENTO: ....../....../......
ENDEREÇO:.. Nº .... APTO: ....
BAIRRO: ....CIDADE ....
CEP:... TELEFONE: DDD (...........) ....

2.RESPONSÁVEL LEGAL ....
NATUREZA (grau de parentesco, tutor, curador etc.) ....
DOCUMENTO DE IDENTIDADE :..SEXO: M F
DATA NASCIMENTO: ....../......./......
ENDEREÇO: .... Nº .... APTO: ....
BAIRRO:.. CIDADE: ....
CEP: .... TELEFONE: DDD (............)..

II - DADOS SOBRE A PESQUISA CIENTÍFICA

1. TÍTULO DO PROTOCOLO DE PESQUISA

DISFUNÇÃO COGNITIVA APÓS CIRURGIA SOB ANESTESIA GERAL

PESQUISADOR: Maria José Carvalho Carmona.
CARGO/FUNÇÃO: Professora Associada.... INSCRIÇÃO CONSELHO REGIONAL Nº ...54.142
UNIDADE DO HCFMUSP: .Divisão de Anestesia do Instituto Central

3. AVALIAÇÃO DO RISCO DA PESQUISA:

SEM RISCO ☐ RISCO MÍNIMO X RISCO MÉDIO ☐

RISCO BAIXO ☐ RISCO MAIOR ☐

(probabilidade de que o indivíduo sofra algum dano como consequência imediata ou tardia do estudo)

4.DURAÇÃO DA PESQUISA : .2 anos.

III - REGISTRO DAS EXPLICAÇÕES DO PESQUISADOR AO PACIENTE OU SEU REPRESENTANTE LEGAL SOBRE A PESQUISA:O senhor (a) está sendo convidado(a) a participar de uma pesquisa, coordenada por um profissional da saúde agora denominado pesquisador. Para poder participar é necessário que o senhor(a) leia este documento com atenção. Ele pode conter palavras que o senhor(a) não entenda. Por favor peça aos responsáveis pelo estudo para explicar qualquer palavra ou procedimentos que o senhor(a) não entenda claramente.

O propósito deste documento é dar o senhor(a) as informações sobre a pesquisa e, se assinado, dará a sua permissão para participar do estudo. O documento descreve o objetivo, procedimentos, benefícios e eventuais riscos ou desconfortos caso queira participar. O senhor(a) só deve participar do estudo se quiser e pode se recusar a participar ou se retirar deste estudo a qualquer momento.

O objetivo desse estudo é determinar a incidência de alterações da função mental, especialmente da memória após anestesia geral e avaliar os efeitos da dexametasona sobre a incidência de disfunção cognitiva (alterações da memória) em pacientes submetidos a cirurgia sob anestesia geral. O uso de corticóide antes da cirurgia pode diminuir a dor pós-operatória, além de diminuir a fadiga, a ocorrência de náuseas e vômitos, o período de recuperação e tempo de retorno às atividades diárias. Por outro lado, a dexametasona tem riscos de efeitos colaterais cardiovasculares, ósseos, retenção de líquidos, aumento de gorduras e glicose no sangue, mas como será usada em dose baixa e única não é de esperar a ocorrência de qualquer efeito colateral.

Antes da cirurgia o senhor(a) será sorteado para receber ou não dexametasona e os pacientes que receberam esta medicação serão comparados com os que não receberam esta medicação.

Participarão do estudo pessoas acima dos 60 anos que serão submetidas a cirurgia sob anestesia geral e que não tenham alteração da função mental e que não estejam usando cortisona. O senhor já foi anteriormente contactado por via telefônica e, se o senhor deu seu consentimento verbal para a pesquisa, respondeu algumas perguntas que indicaram que o senhor(a) pode participar desta pesquisa. Se o senhor concordar em continuar participando deste estudo, um psicólogo lhe aplicará alguns testes que o senhor responderá verbalmente no dia anterior à cirurgia, na noite do dia da cirurgia e no 3º, 7º, 21º e 90º dia após a cirurgia. Nestas datas, o senhor será convidado a comparecer ao hospital para a realização dos testes ou os mesmos serão aplicados por via telefônica.

O segundo objetivo desta pesquisa é a investigação da possível associação entre alterações mentais no pós-operatório e a presença de uma tendência hereditária para este fato, avaliada pela presença de proteínas específicas no sangue. Para tal haverá necessidade da coleta de uma quantidade de 6 mL de sangue que será dividido em 2 amostras e cada tubo com sangue será encaminhado a um laboratório para realização de exames específicos.

O senhor(a) não terá aumento de seu risco cirúrgico por participar desta pesquisa. A coleta das duas amostras de 3mL de sangue será feita no momento da punção da veia para administração da dexametasona antes da cirurgia. Esta mesma veia será utilizada durante a cirurgia para que o senhor receba anestésicos e soro. Durante a cirurgia, os controles feitos pelo anestesista incluirão a utilização de um aparelho já bastante utilizado em anestesia chamado monitor de índice bispectral (BIS) e que utiliza 2 eletrodos conectados na testa para determinação da profundidade da anestesia.

É através das pesquisas clínicas que ocorrem os avanços na medicina, e sua participação é de fundamental importância. Esta pesquisa não ira trazer benefícios diretos para o senhor(a), mas ela irá nos ajudar a melhor entender a alteração da função mental após cirurgia.

IV - ESCLARECIMENTOS DADOS PELO PESQUISADOR SOBRE GARANTIAS DO SUJEITO DA PESQUISA :

A sua participação neste estudo é voluntária. O senhor(a) tem a liberdade de recusar participar do estudo, ou se aceitar participar, retirar seu consentimento a qualquer momento. Este fato não implicará na interrupção de seu atendimento e tratamento, os quais estão assegurados.

Pela sua participação no estudo, o senhor não receberá qualquer valor em dinheiro ou terá qualquer custo. Em caso de eventuais danos à saúde decorrentes desta pesquisa, o senhor terá disponibilidade de assistência neste hospital.

As informações relacionadas ao estudo poderão ser inspecionadas pelos médicos que executam a pesquisa e pelas autoridades legais. No entanto, se qualquer informação for divulgada em relatório ou publicação, isto será feito sob forma codificada sem nomes. Esta medida assegura sua confidencialidade. Quando os resultados forem publicados, não aparecerá seu nome, e sim um código. O senhor(a) tem direito de acesso aos seus dados. O senhor(a) pode discutir esta questão mais adiante com seu médico do estudo. Se o senhor(a) ou seus parentes tiver(em) alguma dúvida com relação ao estudo, direitos do paciente, ou no caso de danos relacionados ao estudo, o senhor(a) deve contactar o pesquisador ou sua equipe .Se o senhor(a) tiver dúvidas sobre seus direitos como um paciente de pesquisa, você pode contactar o Comitê de Ética em Pesquisa em Seres Humanos do Hospital (CEP). A CEP trata-se de um grupo de indivíduos com conhecimento científicos e não científicos que realizam a revisão ética inicial e continuada do estudo de pesquisa para mantê-lo seguro e proteger seus direitos.

V. INFORMAÇÕES DE NOMES, ENDEREÇOS E TELEFONES DOS RESPONSÁVEIS PELO ACOMPANHAMENTO DA PESQUISA, PARA CONTATO EM CASO DE INTERCORRÊNCIAS CLÍNICAS E REAÇÕES ADVERSAS.

Dra Maria José Carvalho Carmona

Divisão de Anestesia do ICHC-FMUSP

Av Enéas Carvalho de Aguiar, n 255 – São Paulo

Fone: 11 3069-5012 ou 3069-6335

VI. OBSERVAÇÕES COMPLEMENTARES:

VII - CONSENTIMENTO PÓS-ESCLARECIDO

Declaro que, após convenientemente esclarecido pelo pesquisador e ter entendido o que me foi explicado, consinto em participar do presente Protocolo de Pesquisa.

São Paulo, de de .

____ __________________________________

assinatura do sujeito da pesquisa ou responsável legal | assinatura do pesquisador (carimbo ou nome Legível)

DISCIPLINA DE ANESTESIOLOGIA – FMUSP
INSTITUTO CENTRAL DO HCFMUSP

Protocolo de Pesquisa

DISFUNÇÃO COGNITIVA APÓS CIRURGIA SOB ANESTESIA GERAL

Pesquisadores:
Profa. Dra. Maria José Carvalho Carmona
Dra. Kátia Osternack Pinto
Dr. Rólisson G. B. Lellis
Dr. Luiz Marcelo Sá Malbouisson
Prof. Dr. José Otávio Costa Auler Jr.

2007

DISFUNÇÃO COGNITIVA APÓS CIRURGIA SOB ANESTESIA GERAL

RESUMO

Alguns dos determinantes de disfunção cognitiva pós-operatória são: o uso de opióides no período pós-operatório, falta de atividade física, fadiga pós-operatória, a dor pós-operatória, a qualidade de vida, além de possível predisposição genética. Os pacientes idosos são mais vulneráveis à ocorrência de alterações cognitivas pós-operatórias. O uso de corticóide pré-operatório diminui a dor pós-operatória, a necessidade de opióides, além de diminuir a fadiga, a incidência de náusea e vômito, o período de recuperação e o tempo de retorno às atividades diárias no período pós-operatório, sendo freqüentemente utilizado como coadjuvante em anestesia. Os objetivos deste estudo são: 1) avaliar os efeitos da dexametasona sobre a incidência de disfunção cognitiva em pacientes acima de 60 anos submetidos à cirurgia sob anestesia geral; 2) Investigar a associação entre alterações cognitivas no pós-operatório e a presença do alelo ε4 do gene da apolipoproteína e de marcadores bioquímicos séricos para lesão neuronal, como a proteína S100β e a enolase específica do neurônio (NSE). Prevê-se o estudo de 300 pacientes aos quais serão aplicados testes neuropsicológicos para avaliar o estado mental geral, aprendizagem, atenção, percepção visuoespacial, memória imediata, operacional e de evocação e habilidades executivas, incluindo velocidade de processamento. Esta bateria de testes define o índice cognitivo da estabilidade (medida longitudinal do status cognitivo em avaliações repetidas que detectam mudanças estatisticamente significantes no funcionamento do sistema nervoso central). Os pacientes selecionados para o estudo serão contatados por via telefônica no dia anterior ao estudo e, se derem o consentimento verbal, serão submetidos a teste neuropsicológico específico para ser aplicado por via telefônica. Após a internação hospitalar e assinatura do TCLE o paciente será submetido os testes neuropsicológicos específicos e randomizado para participação em um dos dois grupos de estudo, sendo que o psicólogo que aplicará os testes neuropsicológicos não terá conhecimento do grupo ao qual o paciente participa: **Grupo Dexametasona**: 8 mg de dexametasona por via venosa, aplicada duas horas antes do horário marcado para início do procedimento cirúrgico e **Grupo Controle**: realização da técnica anestésica habitual, sem utilização de dexametasona. A anestesia geral seguirá os padrões gerais incluindo Propofol (2-3 mg/kg), rocuronio (0.5 mg/kg), e fentanil (1-2 µg/kg) para indução. A anestesia subseqüente e mantida com sevoflurano (1.0-2.5%) e N_20 em 40% de oxigênio, rocuronio (doses suplementares serão administradas se necessário para manutenção de anestesia cirúrgica), e fentanil (doses suplementares 0.5-10 µg/kg com dose máxima de 250 µg). A administração de sevoflurano será ajustada com o objetivo de manter os níveis de hipnose avaliada pelo índice bispectral (BIS) de acordo com os grupos do estudo: os participantes serão randomizados em 2 grupos: anestesia superficial (índice bispectral entre 45 e 55) e anestesia profunda (índice bispectral entre 35 e 45). Ambos níveis são aceitáveis para anestesia geral e são atualmente usados por anestesiologistas. Após venóclise periférica, antes da indução anestésica, será colhida uma amostra de sangue para determinação do perfil genotípico da Apoε e dosagem de S100β e NSE. Os testes neuropsicológicos serão aplicados antes da cirurgia, na noite após a cirurgia e nos dias pós-operatórios 3, 7, 21, e 90.Os resultados dos escores dos testes em cada grupo serão comparados através do teste de Friedmann, e entre os grupos será utilizado o teste de Kruskal Wallis. Porcentagens serão comparadas pelo do teste de Qui-Quadrado. Os dados serão expressos como mediana e intervalo de confiança de 95%. Serão considerados significativos resultados cujos valores p sejam menores que 0,05 nos testes estatísticos.

DISFUNÇÃO COGNITIVA APÓS CIRURGIA SOB ANESTESIA GERAL

1 – INTRODUÇÃO

Disfunção cognitiva no pós-operatório imediato de cirurgias sob anestesia geral é evento descrito com freqüência crescente, especialmente em pacientes geriátricos[1-5]. A idade é o principal fator de risco para disfunção cognitiva pós-operatória (COPD) e pacientes idosos submetidos a grandes cirurgias devem ser adequadamente avaliados quando referem alterações cognitivas, especialmente da memória, no período pós-operatório[4]. As alterações cerebrais próprias da idade, a presença de aterosclerose, fatores genéticos, alterações na metabolização de fármacos, a resposta inflamatória sistêmica relacionada à cirurgia, dentre outros fatores podem estar implicados na fisiopatologia da COPD[4]. Estudos mostram que alguns dos determinantes de disfunção cognitiva pós-operatória são: o uso de opióides no período pós-operatório, falta de atividade física, fadiga pós-operatória, dor pós-operatória e qualidade de vida[4, 6, 7]. A ocorrência de COPD pode comprometer o resultado cirúrgico, aumentar a morbimortalidade e piorar a qualidade de vida pós-operatória da população geriátrica, daí a importância do estudo de agentes e técnicas que possam minimizar esta complicação.

Na maioria dos casos a disfunção cognitiva pós-operatória é reversível, podendo ser permanente em menos de 1% dos pacientes. Procedimentos sob anestesia regional também podem causar, com menor freqüência, disfunção cognitiva transitória no pós-operatório imediato[4, 8, 9]. A predisposição genética tem sido aventada como possível fator de risco para COPD[10].

O uso de corticóide pré-operatório diminui a dor pós-operatória e também a necessidade de opióides. Diminui fadiga, náusea e vômito, período de recuperação e tempo de retorno às atividades diárias no período pós-operatório. Todos estes fatores melhoram qualidade de vida. Se o corticóide modifica positivamente fatores que estão ligados à disfunção cognitiva como dor, uso de opióides, fadiga, recuperação pós-operatória, convalescença, qualidade de vida e mais rápido retorno às atividades físicas, ele também modificaria positivamente a função cognitiva no período pós-operatório.

Considerando que os pacientes idosos constituem o grupo de maior risco para apresentação de COPD, os objetivos deste estudo são:

1) Avaliar os efeitos da dexametasona sobre a incidência de disfunção cognitiva no pós-operatório em pacientes acima de 60 anos, submetidos à cirurgia sob anestesia geral;
2) Investigar a associação entre alterações cognitivas no pós-operatório e a presença do alelo ε4 do gene da apolipoproteína E e de marcadores bioquímicos séricos para lesão neuronal, como a proteína S100β e a enolase específica do neurônio (NSE).

3 – CASUÍSTICA E MÉTODOS

Após aprovação pela CAPPesq e obtenção de auxílio financeiro para o estudo, serão estudados 300 pacientes acima de 60 anos, de ambos os sexos e com indicação de anestesia geral para procedimentos cirúrgicos gerais sem utilização de circulação extracorpórea.

Serão aplicados testes neuropsicológicos para avaliar o estado mental geral, aprendizagem, atenção, percepção visuoespacial, memória imediata, operacional e de evocação e habilidades executivas, incluindo velocidade de processamento. Esta bateria define o índice cognitivo da estabilidade (medida longitudinal do status cognitivo em avaliações repetidas que detectam mudanças estatisticamente significantes no funcionamento do sistema nervoso central). A avaliação será realiza antes da cirurgia, na noite após a cirurgia e nos dias pós-operatórios 3, 7, 21, e 90.

Critérios de inclusão: paciente acima de 60 anos, com programação cirúrgica para videocirurgia para herniorrafia inguinal, operação de Nissen ou colecistectomia sob anestesia geral com programação de permanência hospitalar de até 24 horas após a cirurgia.

Critérios de exclusão: idade inferior a 60 anos, história pregressa de doença cerebral ou demência, outras doenças psiquiátricas que afetem a cognição, falta de domínio da língua portuguesa, uso de corticóide ou de opióde.

Os pacientes agendados para cirurgia que preencherem os critérios para participação do estudo serão contactados por via telefônica e convidados a participar do mesmo. Aqueles que derem a concordância verbal para participarem do estudo serão submetidos a testes neuropsicológicos específicos para serem utilizados por via telefônica, antes da internação hospitalar e da assinatura do TCLE. Após os testes os pacientes estarão liberados ou não para fazer parte do estudo. Após a internação hospitalar, os pacientes selecionados para participar do estudo serão novamente esclarecidos sobre o mesmo e será solicitada a assinatura do TCLE em duas vias, sendo que uma cópia ficará com o paciente e outra com o pesquisador.

Após assinatura do TCLE o paciente será submetido os testes neuropsicológicos específicos e será randomizado para participação em um dos dois grupos de estudo, sendo que o psicólogo que aplicará os testes neuropsicológicos não terá conhecimento do grupo ao qual o paciente participa:

1. **Grupo Dexametasona**: 8 mg de dexametasona por via venosa, aplicada duas horas antes do horário marcado para início do procedimento cirúrgico;
2. **Grupo Controle**: realização da técnica anestésica habitual, sem utilização de dexametasona.

Após venóclise periférica, antes da indução anestésica, serão colhidas duas amostras de sangue de para determinação do perfil genotípico da Apoε e dosagem de S100β e NSE. As amostras serão processadas no Laboratório de Biologia Molecular do InCor (APOε4) e Laboratório de

Neurociências da UFRGS (S100β e NSE), sendo que não haverá envio de amostras sangüíneas para o exterior.

A anestesia geral será induzida com utilização dos seguintes fármacos: Propofol (2-3 mg/kg), rocuronio (0.5 mg/kg), e fentanil (1-2 μg/kg) para indução. A anestesia subseqüente será mantida com sevoflurano (1.0-2.5%) e N_20 em 40% de oxigênio, rocuronio (doses suplementares serão administradas se necessário para manutenção de anestesia cirúrgica), e fentanil (doses suplementares 0.5-10 μg/kg com dose máxima de 250 μg). Variação em requerimentos individuais preclude administração de doses idênticas para cada paciente. Ao invés, a administração de sevoflurano deverá ser ajustada pelo anestesiologista com o objetivo de manter of níveis do BIS (hipnose) de acordo com os grupos do estudo, e fentanil será administrado com o objetivo de manter a pressão arterial e freqüência cardíaca (analgesia) dentro de 20% dos valores pré-indução. A ventilação será controlada mecanicamente para manter a pressão de dióxido de carbono próximo a 35 mmHg. Fentanil (1-2 μg/kg) será dado ao final da cirurgia para melhorar a analgesia durante a recuperação anestésica. Soro fisiológico 5-10 ml/kg/hora será dado durante a cirurgia.

O índice bispectral mede a atividade cerebral cortical (hipnose) numa escala de 0 a 100, onde 100 representa o estado "acordado" e 0 representa um eletroencefalograma isoelétrico. Medidas abaixo de 70 estão relacionadas à inconsciência e ausência de memória necessária para a anestesia geral. Neste estudo, os participantes serão randomizados em 2 grupos: anestesia superficial (índice bispectral entre 45 e 55) and anestesia profunda (índice bispectral entre 35 e 45). Ambos os níveis são aceitáveis para anestesia geral e são atualmente usados por anestesiologistas.

A pele da região onde o monitor vai ser aplicado deve estar limpa e seca. Use álcool e seque esta área da pele.

Aplique o sensor na testa do paciente. Coloque o círculo número 1 no centro aproximadamente 2 cm acima da ponte nasal. Coloque o círculo número 3 na área temporal.

Importante: Aplique pressão em torno do sensor (incluindo as áreas entre os círculos) para garantir a adesão adequada. Em seguida, pressione os círculos 1, 2 e 3 por 5 segundos para assegurar o contato dos eletrodos à pele.

Conecte o sensor aplicado na testa do participante no cabo do índice bispectral.

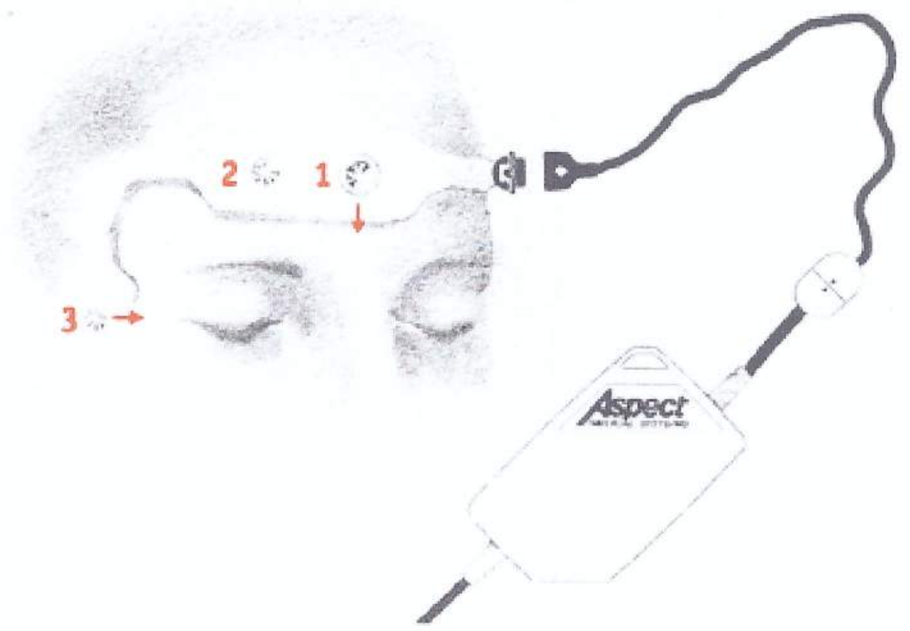

A avaliação da disfunção cognitiva nos dois grupos será realizada através de testes neuropsicológicos, padronizados especificamente para avaliar as funções cognitivas superiores, e questionários para avaliar as características sócio-demográficas, questionamentos sobre a percepção pessoal da saúde do paciente. Estes testes serão aplicados no pré-operatório, na noite anterior à cirurgia, antes da administração de medicações pré-anestésicas ou sedativas, no 3°, 7°, 21° e 90° dia pós-operatório, conforme detalhado abaixo.

Fase 1:

Período: pré e pós-operatório imediatos
Procedimento: aplicar pessoalmente em enfermaria
Instrumento: ISPOCD

Fase 2:

Período: 3° e 7° dias pós-operatórios
Procedimento: aplicar via telefone
Instrumento: TICS

Fase 3:

Período: 21° dia pós-operatório
Procedimento: aplicar pessoalmente em retorno ambulatorial
Instrumento: ISPOCD

Fase 4:

Período: 90° dia pós-operatório
Procedimento: aplicar via telefone
Instrumento: TICS

Os dados serão analisados utilizando-se o software de análise estatística SPSS versão 10. Os dados demográficos como idade, peso, altura serão comparados nos quatro grupos através de análise de

variância univariada seguida de teste de comparações múltiplas se aplicável. As variáveis colhidas através de testes neuropsicológicos aplicados antes e depois do procedimento cirúrgico são de natureza qualitativa ou ordinal, sendo atribuídos resultados numéricos e os resultados finais dos testes serão expressos em escores. Os resultados dos escores dos testes em cada grupo serão comparados através do teste de Friedmann, e entre os grupos será utilizado o teste de Kruskal Wallis. Mann Whitney para comparações da evolução do sujeito Porcentagens serão comparadas através do teste de Qui-Quadrado Os dados serão expressos como mediana e intervalo de confiança de 95%. Serão considerados significativos resultados cujos valores p sejam menores que 0,05 nos testes estatísticos.

INSTRUMENTOS

1- Informações Sócio-demográficas

Serão investigadas as informações sobre idade, sexo, escolaridade, estado civil, ocupação, e demais informações relevantes para compor o perfil sociodemográfico da amostra, bem como a compatibilidade com os critérios de exclusão e inclusão, conforme detalhado em anexo.

2- Versão Brasileira do Questionário de Qualidade de Vida SF-36

O SF-36 investiga condições relacionadas à saúde, atividades de vida diária, produtividade, problemas emocionais, relacionamentos, motivação e outras condições que traçam o perfil da qualidade de vida a partir da percepção do próprio sujeito, conforme apresentado em anexo.

3- International Study of Postoperative Cognitive Dysfunction - ISPOCD

A bateria neuropsicológica proposta neste estudo seguirá o protocolo sugerido pelo Estudo Internacional de Disfunção Cognitiva Pós-Cirúrgica (do inglês, International Study of Postoperative Cognitive Disfunction - ISPOCD) para avaliar as funções de memória, velocidade sensoriomotora, flexibilidade mental e produtividade motora, com a finalidade de detectar disfunções neuropsicológicas sutis que possam ocorrer em pacientes após cirurgia com anestesia geral ou regional (Rasmussen et al., 1999, Rasmussen et al., 2001 e Rasmussen et al., 2002). Para detalhes, ver anexos.

4- Entrevista Telefônica para o Status Cognitivo (Telephone Interview for Cognitive Status - TICS)

Instrumentos padronizados para realização de entrevista telefônica têm se mostrado válidos e sensíveis, sendo muito indicados para utilizar em situações onde a avaliação de rastreio cognitivo realizada pessoalmente é impraticável ou ineficiente, como, por exemplo, em pesquisas epidemiológicas de grandes populações ou com pacientes que estão incapacitados de comparecerem ao retorno clínico (Ferrucci et al., 1998, De Jager et al., 2003, Barber & Stott, 2004, Musselwhite et al., 2006 e Dal Forno et al., 2006).

O TICS - Entrevista Telefônica para o Status Cognitivo é um teste padronizado para avaliação do funcionamento neuropsicológico que foi desenvolvido para utilizar em situações onde a avaliação de rastreio cognitivo realizada pessoalmente é impraticável ou ineficiente, como, por exemplo, em pesquisas epidemiológicas de grandes populações ou com pacientes que estão incapacitados de comparecerem ao retorno clínico. É muito útil também para avaliar pessoas com incapacidade para

ler ou escrever, pois pode ser aplicado pessoalmente através de entrevista, exigindo apenas capacidade de compreensão verbal.
O teste consiste em um roteiro de entrevista com onze itens abordando as habilidades de orientação espacial e temporal controle mental, memória, informação geral, repetição de sentenças, memória semântica, praxias e antônimos de palavras, conforme detalhado em anexo.

4- ANÁLISE DO RISCO

O risco da pesquisa é mínimo, pois não modificará a técnica anestésica ou cirúrgica rotineiramente utilizada. A única intervenção proposta é a utilização de dexametasona em metade da população estudada, fármaco este freqüentemente utilizado em anestesia para prevenção de vômitos pós-operatórios. A monitorização utilizada e a amostra sangüínea colhida não acrescentam risco ao procedimento cirúrgico.

5- CRONOGRAMA

Estimamos um prazo máximo de 24 meses para o estudo, sendo 18 meses para a coleta dos dados, 2 meses para análise estatística completa dos resultados obtidos e 2 a 4 meses para formulação do artigo científico a ser publicado.

6- FINANCIAMENTO DA PESQUISA

As fontes de financiamento da pesquisa serão:

1. Duke University:
    - Forneciemento dos eletrodos para avaliação do índice bispectral (BIS) para ser utilizado em monitor já comercializado no Brasil e disponível no ICHC.
    - compra dos testes neuropsicológicos específicos para o estudo
    - Auxílio para análise estatística e custos de publicação
2. FAPESP ou CNPQ: após aprovação do projeto pela CAPPesq será solicitado auxílio em valor estimado de R$20.000,00 (vinte mil reais) para:
    - aplicação dos testes neuropsicológicos pelo Serviço de Neuropsicologia
    - de *laptop* específico para aplicação dos testes neuropsicológicos.
    - Custos da pesquisa de APOε4 pelo Laboratório de Biologia Molecular do INCOR
    - Custos da dosagem de S100β e NSE pelo Laboratório de Neurociências da UFRGS.

7- PROCEDIMENTOS APÓS A REALIZAÇÃO DA PESQUISA

Os dados e resultados obtidos serão armazenados em banco de dados seguro e sigiloso. Após a análise completa dos resultados, serão submetidos à publicação em periódico científico indexado apropriado.

8- REFERÊNCIAS BIBLIOGRÁFICAS

1. Ancelin, M.L., et al., *Exposure to anaesthetic agents, cognitive functioning and depressive symptomatology in the elderly.* Br J Psychiatry, 2001. **178**: p. 360-6.
2. Bekker, A.Y. and E.J. Weeks, *Cognitive function after anaesthesia in the elderly.* Best Pract Res Clin Anaesthesiol, 2003. **17**(2): p. 259-72.
3. Cohendy, R., A. Brougere, and P. Cuvillon, *Anaesthesia in the older patient.* Curr Opin Clin Nutr Metab Care, 2005. **8**(1): p. 17-21.
4. Rasmussen, L.S. and J.T. Moller, *Central nervous system dysfunction after anesthesia in the geriatric patient.* Anesthesiol Clin North America, 2000. **18**(1): p. 59-70, vi.
5. Xie, Z. and R.E. Tanzi, *Alzheimer's disease and post-operative cognitive dysfunction.* Exp Gerontol, 2006. **41**(4): p. 346-359.
6. Benoit, A.G., et al., *Risk factors and prevalence of perioperative cognitive dysfunction in abdominal aneurysm patients.* J Vasc Surg, 2005. **42**(5): p. 884-90.
7. Wu, C.L., et al., *Postoperative cognitive function as an outcome of regional anesthesia and analgesia.* Reg Anesth Pain Med, 2004. **29**(3): p. 257-68.
8. Campbell, D.N., et al., *A prospective randomised study of local versus general anaesthesia for cataract surgery.* Anaesthesia, 1993. **48**(5): p. 422-8.
9. Canet, J., et al., *Cognitive dysfunction after minor surgery in the elderly.* Acta Anaesthesiol Scand, 2003. **47**(10): p. 1204-10.
10. Lelis, R.G., et al., *Apolipoprotein E4 genotype increases the risk of postoperative cognitive dysfunction in patients undergoing coronary artery bypass graft surgery.* J Cardiovasc Surg (Torino), 2006. **47**(4): p. 451-6.

ANEXOS: Instrumentos utilizados no protocolo de pesquisa

Informações Sócio-demográficas

Critérios para inclusão no estudo:

- Pacientes acima de 60 anos
- Submetido à cirurgia de pequeno porte
- Sob anestesia geral

Critérios para exclusão:

1. Idade inferior a 60 anos
2. História pregressa de doença cerebral ou demência
3. Outras doenças psiquiátricas que afetem a cognição
4. Falta de domínio da língua portuguesa
5. Estar fazendo uso de corticóide

- Nome: ____
- Diagnóstico: ____
- Sexo: masculino feminino
- Idade (anos): __________
- Peso (quilos): __________
- Altura (centímetros): __________

Estado civil

( ) solteiro (a)
( ) casado(a)
( ) divorciado(a)
( ) viúvo(a)

Emprego

( ) empregado(a)
( ) desempregado(a)
( ) aposentado(a)

Salário mensal:

( ) 0
( ) 0-1 salário mínimo
( )1-5 salários mínimos
( )6-10 salários mínimos
( )10-15 salários mínimos
( )15-20 salários mínimos
( ) mais que 20 salários mínimos

Escolaridade

( ) Nenhuma
( ) Primário incompleto
( ) Primário completo
( ) Ginásio completo
( ) Colegial completo
( ) Superior completo
(..) Pós-graduação

Versão Brasileira do Questionário de Qualidade de Vida SF-36

Instruções: Esta pesquisa questiona você sobre sua saúde. Estas informações nos manterão informados de como você se sente quão bem você é capaz de fazer suas atividades de vida diária. Responda cada questão marcando a resposta como indicado. Caso você esteja inseguro ou em dúvida em como responder, por favor, tente responder o melhor que puder.

1. Em geral, você diria que a sua saúde é:

(circule uma)

Excelente	Muito boa	Boa	Ruim	Muito ruim
1	2	3	4	5

2. Comparada há um ano atrás, como você classificaria a sua saúde em geral, agora?

(circule uma)

Muito melhor	Um pouco melhor	Quase a mesma	Um pouco pior	Muito pior
1	2	3	4	5

3. Os seguintes itens são sobre atividades que você poderia fazer atualmente durante um dia comum. Devido a sua saúde, você teria dificuldade para fazer essas atividades? Neste caso, quanto?

(circule um número em cada linha)

Atividades	Sim. Dificulta muito	Sim. Dificulta um pouco	Não. Não dificulta de modo algum
A. Atividades vigorosas, que exigem muito esforço, tais como correr, levantar objetos pesados, participar em esportes árduos.	1	2	3
b. Atividades moderadas, tais como mover uma mesa, passar aspirador de pó, jogar bola, varrer a casa.	1	2	3
c. Levantar ou carregar mantimentos	1	2	3
d. Subir vários lances de escada	1	2	3
e. Subir um lance de escadas	1	2	3
f. Curvar-se, ajoelhar-se ou dobrar-se	1	2	3
g. Andar mais de 1 quilômetro	1	2	3
h. Andar vários quarteirões	1	2	3
i. Andar um quarteirão	1	2	3
j. Tomar banho ou vestir-se	1	2	3

4. Durante as últimas 4 semanas, você teve algum dos seguintes problemas com o seu trabalho ou com alguma atividade diária regular, como conseqüência de sua saúde física?

(circule uma em cada linha)

	Sim	Não
a. Você diminuiu a quantidade de tempo que se dedicava ao seu trabalho ou a outras atividades?	1	2
b. Realizou menos tarefas do que você gostaria?	1	2
c. Esteve limitado no seu tipo de trabalho ou em outras atividades?	1	2
d. Teve dificuldade de fazer seu trabalho ou outras atividades (p.ex.: necessitou de um esforço extra)?	1	2

5. Durante as últimas 4 semanas, você teve alguns dos seguintes problemas com o seu trabalho ou outra atividade regular diária, como conseqüência de algum problema emocional (como sentir-se deprimido ou ansioso)?

(circule uma em cada linha)

	Sim	Não
a. Você diminuiu a quantidade de tempo que se dedicava ao seu trabalho ou a outras atividades?	1	2
b. Realizou menos tarefas do que você gostaria?	1	2
c. Não trabalhou ou não fez qualquer das atividades com tanto cuidado como geralmente faz?	1	2

6. Durante as últimas 4 semanas, de que maneira sua saúde física ou problemas emocionais interferiram nas suas atividades sociais normais, em relação a família, vizinhos, amigos ou em grupo?

(circule uma)

De forma nenhuma	Ligeiramente	Moderadamente	Bastante	Extremamente
1	2	3	4	5

7. Quanta dor no corpo você teve durante as últimas 4 semanas?

(circule uma)

Nenhuma	Muito leve	Leve	Moderada	Grave	Muito grave
1	2	3	4	5	6

8. Durante as últimas 4 semanas, quanto a dor interferiu com o seu trabalho normal (incluindo tanto o trabalho fora de casa e dentro de casa)?

(circule uma)

De maneira alguma	Um pouco	Moderadamente	Bastante	Extremamente
1	2	3	4	5

9. Estas questões são sobre como você se sente e como tudo tem acontecido com você durante as últimas 4 semanas. Para cada questão, por favor dê uma resposta que mais se aproxime da maneira como você se sente. Em relação às últimas 4 semanas.
(circule um número para cada linha)

	Todo tempo	A maior parte do tempo	Uma boa parte do tempo	Alguma parte do tempo	Uma pequena parte do tempo	Nunca
a. Quanto tempo você tem se sentido cheio de vigor, cheio de vontade, cheio de força?	1	2	3	4	5	6
b. Quanto tempo você tem se sentido uma pessoa muito nervosa?	1	2	3	4	5	6
c. Quanto tempo você tem se sentido tão deprimido que nada pode animá-lo?	1	2	3	4	5	6
d. Quanto tempo você tem se sentido calmo e tranqüilo?	1	2	3	4	5	6
e. Quanto tempo você tem se sentido com muita energia?	1	2	3	4	5	6
f. Quanto tempo você tem se sentido desanimado e abatido?	1	2	3	4	5	6
g. Quanto tempo você tem se sentido esgotado?	1	2	3	4	5	6
h. Quanto tempo você tem se sentido uma pessoa feliz?	1	2	3	4	5	6
i. Quanto tempo você tem se sentido cansado?	1	2	3	4	5	6

10. Durante as últimas 4 semanas, quanto do seu tempo a sua saúde física ou problemas emocionais interferiram com as suas atividades sociais (como visitar amigos, parentes, etc.)?
(circule uma)

Todo o temp0	A maior parte do tempo	Alguma parte do tempo	Uma pequena parte do tempo	Nenhuma parte do tempo
1	2	3	4	5

11. O quanto verdadeiro ou falso é cada uma das afirmações para você?
(circule uma)

	Definitiva-mente verdadeiro	A maioria das vezes verdadeiro	Não sei	A maioria das vezes falsa	Definitiva-mente falsa
a. Eu costumo adoecer um pouco mais facilmente que as outras pessoas	1	2	3	4	5
b. Eu sou tão saudável quanto qualquer pessoa que eu conheço	1	2	3	4	5
c. Eu acho que a minha saúde vai piorar	1	2	3	4	5
d. Minha saúde é excelente	1	2	3	4	5

International Study of Postoperative Cognitive Dysfunction - ISPOCD

- *Mini- Exame do Estado Mental (The Mini Mental State Examination* - MMSE).

Originalmente desenvolvido por (Folstein et al., 1975) para ser utilizado para realização de rasterio para demência. Inclui perguntas para avaliar as habilidades de orientação espacial e temporal, memória, seguimento de instrução visual, nomeação de objetos, redação de repetição de sentenças, controle mental e cópia de figura, conforme modelo em anexo. Serão utilizados os pontos de corte sugeridos na adaptação para a população brasileira por Bertolucci e colaboradores (1994).

- *Aprendizagem Verbal Visual (Visual Verbal Learning*).

Baseado no teste de aprendizagem verbal de Rey, será utilizado para avaliar a aprendizagem verbal. Será apresentada uma lista de 15 palavras em uma tela de computador para ser memorizada e recordada em três tentativas sucessivas, com evocação tardia após 15 a 25 minutos. Serão avaliados o número de palavras recordadas e o número de erros cometidos (palavras não fornecidas).

- *Tarefa de Alternância Conceitual (Concept Shifting Task*).

Baseada nos testes de trilhas, será fornecida uma folha de papel na qual o sujeito deverá traçar linhas ligando círculos em ordem seqüencial de números (parte A), letras (parte B) ou números e letras alternados (parte C) randomicamente distribuídos e, finalmente, deverá ligar círculos vazios no sentido horário. Serão avaliados o tempo despedido e o número de erros cometidos (seqüência incorreta).

- *Teste de Palavras Coloridas de Stroop (Stroop Colour Word Test)*.

Serão apresentados cartões nos quais o sujeito ler 40 nomes de cores (parte 1), nomear 40 retângulos coloridos (parte 2) e 40 nomes de cores impressas em outra cor. Serão avaliados o tempo despedido e o número de erros (cores ou palavras incorretas) em cada cartão.

- *Tarefa de Memória de Reconhecimento (Memory Scanning Task)*.

Será apresentada uma folha de papel com objetos-alvo que deverão ser memorizados e, em seguida, serão apresentados 120 estímulos (20 estímulos-alvo e 100 distratores), distribuídos em dez linhas, nos quais o indivíduo deverá identificar aqueles que estavam entre os estímulos inicialmente apresentados. Serão avaliados o tempo despendido e o número de erros (omissões ou estímulos não apresentados anteriormente).

- *Codificação Letra-Número (Letter-Digit Coding*).

Baseado no subteste Códigos da Escala Wechsler de Inteligência para Adultos (WAIS), será fornecida uma folha de papel com nove letras que correspondem a nove letras, respectivamente. E, abaixo, serão apresentadas letras para as quais o sujeito deverá assinalar o número correspondente durante um minuto. Será avaliado o número de associações corretas entre letra e número realizadas.

- *Teste das Quatro Caixas (Four Boxes Test)*.

Este teste avaliará o tempo de reação para escolha entre quatro estímulos. Serão apresentados quatro caixas em uma tela de computador nas quais 52 círculos pretos aparecerão randomicamente a cada 500ms. O sujeito deverá pressionar o botão correspondente à localização de cada círculo o mais rápido possível. Serão avaliados a média de respostas corretas e o número de erros cometidos.

Entrevista Telefônica para o Status Cognitivo (Telephone Interview for Cognitive Status - TICS)

O TICS é um teste padronizado para avaliação do funcionamento neuropsicológico que foi desenvolvido para utilizar em situações onde a avaliação de rastreio cognitivo realizada pessoalmente é impraticável ou ineficiente, como, por exemplo, em pesquisas epidemiológicas de grandes populações ou com pacientes que estão incapacitados de comparecerem ao retorno clínico. É muito útil também para avaliar pessoas com incapacidade para ler ou escrever, pois pode ser aplicado pessoalmente através de entrevista, exigindo apenas capacidade de compreensão verbal. Segundo os fabricantes, tem alta correlação com o MMSE, com excelente sensibilidade e especificidade para detectar comprometimento cognitivo, inclusive em idosos.

Antes de ser administrado via telefone, o entrevistador deverá conversar com algum familiar ou cuidador para se assegurar de que o ambiente estará adequado à entrevista e de que o sujeito estará em boas condições de ouvir e compreender a linguagem verbal.

O teste consiste em um roteiro de entrevista com onze itens abordando as habilidades de orientação espacial e temporal controle mental, memória, informação geral, repetição de sentenças, memória semântica, praxias e antônimos de palavras.

Serão avaliados os acertos em cada item com escores para a somatória total, utilizando-se ponto de corte como parâmetro de comparação entre as entrevistas realizadas nas fases 2 e 4 descritas no procedimento.

MINI EXAME DO ESTADO MENTAL - MEEM - Versão Brasileira

Questões Pontos

1. Qual é: Ano? Estação (Metade do ano)? Data? Dia? Mês? 5

2. Onde estamos: Estado? País? Cidade? Bairro ou hospital? Andar? 5

3. Nomeie três objetos (carro, vaso, janela) levando 1 segundo para cada. Depois, peça ao paciente que os repita para você. Repita as respostas até o indivíduo aprender as 3 palavras (5 tentativas). 3

4. 7s seriados: Subtraia 7 de 100. Subtraia 7 desse número, etc. 5

Interrompa após 5 respostas. Alternativa: Soletre "MUNDO" de trás para frente. 5.

5. Peça ao paciente que nomeie os 3 objetos aprendidos em 3. 3

6. Mostre uma caneta e um relógio. Peça ao paciente que os nomeie conforme você os mostra. 2

7. Peça ao paciente que repita "nem aqui, nem ali, nem lá". 1

8 Peça ao paciente que obedeça sua instrução: "Pegue o papel com sua mão direita. Dobre-o ao meio com as duas mãos. Coloque o papel no chão". 3

9. Peça ao paciente para ler e obedecer o seguinte: "Feche os olhos". 1

10. Peça ao paciente que escreva uma frase de sua escolha. 1

11. Peça ao paciente que copie o seguinte desenho: 1

Escore total: (máximo de 30) ______

HOSPITAL DAS CLÍNICAS
DA FACULDADE DE MEDICINA
DA UNIVERSIDADE DE SÃO PAULO

PARECER

PROTOCOLO DE PESQUISA Nº: 998/06	Data de entrada: 29/09/07<br>Data sessão: 01.08.07

TÍTULO DA PESQUISA: *Disfunção cognitiva após cirurgia sob anestesia geral*

PESQUISADOR(A) RESPONSÁVEL: Maria José Carvalho Carmona

DEPARTAMENTO: Cirurgia

CONSIDERAÇÕES DO RELATOR APROVADAS PELO PLENÁRIO:

Atendidas as solicitações o projeto está aprovado.

X APROVAR	☐ REPROVAR
☐ DEVOLVER PARA ATENDER AS CONSIDERAÇÕES	☐ CIENTE

TEMÁTICA ESPECIAL:	☐ SIM	☒ NÃO
CONEP:	☐ SIM	☒ NÃO
SVS (SECRETARIA DE VIGILÂNCIA SANITÁRIA):	☐ SIM	☒ NÃO

Diretoria Clínica

Comissão de Ética para Análise de Projetos de Pesquisa - CAPPesq.

MEDICINA
USP

RECEBIDO POR: CA.O
DATA: 10 / 05 / 11
Comissão de Ética para Análise de Projeto de Pesquisa-CAPPesq - HCFMUSP

PG/MA1/020/2011

São Paulo, 09 de maio de 2011.

Ilmo. Sr.
Prof. Dr. Euclides Ayres de Castilho
Presidente da Comissão de Ética para Análise de Projetos de Pesquisa
CAPPESq

Ref.: **Protocolo de Pesquisa 998/06**

Prezado Professor,

Solicitamos a V.Sa. a gentileza de avaliar e efetuar as alterações aqui apresentadas no protocolo de pesquisa em referência, a saber: "*Disfunção cognitiva após cirurgia sob anestesia geral*", para tanto encaminhamos o relatório científico do mesmo.

- ✓ Finalidade acadêmica: Doutorado
- ✓ Pesquisador Responsável/Orientador: Profa. Dra. Maria José Carvalho Carmona (Lattes: 2636111337875377)
- ✓ Pesquisador executante: Dra. Lívia Stocco Sanches Valentin (Lattes: 1665879506366648)
- ✓ Inclusão do teste: avaliação de depressão (BDI – Inventário de Depressão de Beck)
- ✓ Alteração das datas de avaliação neuropsicológica: à noite anterior à cirurgia e 3°, 7°, 21°, 90°, 180° dias pós-operatórios.

Agradecemos antecipadamente a atenção e colocamo-nos à disposição para qualquer esclarecimento que se faça necessário.

Cordialmente,

Prof. Dr. José Otávio Costa Auler Jr.
Professor Titular da Disciplina de Anestesiologia da FMUSP
Coordenador do Programa de Pós-Graduação em Anestesiologia

São Paulo, 19 de Abril de 2011.

Ilmo. Sr.

Prof. Dr. Euclides Ayres de Castilho

D.D. Presidente da Comissão de Ética para Análise de Projetos de Pesquisa –

CAPPesq - HCFMUSP

Ref.: Projeto de Pesquisa nº 998/06 – **"DISFUNÇÃO COGNITIVA APÓS CIRURGIA SOB ANESTESIA GERAL".**

Prezado Professor Massad,

Solicito alteração do projeto de pesquisa acima referido, para inclusão do teste de avaliação de depressão (BDI – Inventário de Depressão de Beck). Tal inclusão se justifica pelas evidências de que a presença de sintomas depressivos podem ser desencadeadores de disfunção cognitiva pós operatória que podem interferir no pronto restabelecimento do paciente após a cirurgia.

Solicito também a alteração das datas de avaliação neuropsicológica que deverão ocorrer nos seguintes dias: noite anterior à cirurgia e 3º, 7º, 21º, 90º, 180º dias pós operatórios.

Adicionalmente, solicito a inclusão da neuropsicóloga e aluna de pós-graduação **Lívia Stocco Sanches Valentin** no projeto de pesquisa (em anexo o cadastro de pesquisador da aluna).

Informo que o projeto de pesquisa obteve auxílio pesquisa FAPESP (processo número 09/54233-0) e encontra-se em fase de coleta de dados. Em anexo, encaminho o relatório parcial FAPESP que inclui o andamento atual do projeto e as alterações aqui solicitadas.

Agradeço antecipadamente a atenção dispensada e coloco-me à disposição para eventuais esclarecimentos que se fizerem necessários.

Atenciosamente,

Profa. Dra. Maria José Carvalho Carmona
Pesquisador Gerente

Processo FAPESP NÚMERO 09/54233-0

1º RELATÓRIO CIENTÍFICO PARCIAL

DISFUNÇÃO COGNITIVA APÓS CIRURGIA SOB ANESTESIA GERAL

MARIA JOSÉ CARVALHO CARMONA
OUTORGADA

Pesquisadores:

Profa. Dra. Maria José Carvalho Carmona

Dra. Kátia Osternack Pinto

Doutoranda Lívia Stocco Sanches Valentin

São Paulo

2011

SUMÁRIO

RESUMO

APÊNDICES 1

1 INTRODUÇÃO 2

2 OBJETIVOS 7

3 REVISÃO DA LITERATURA 9

4 CASUÍSTICA E MÉTODOS 19

4.1 PROJETO DE PESQUISA 19

1.1Critérios de seleção dos pacientes *19*

1.2 Termo de consentimento livre e esclarecido *20*

4.2 CASUÍSTICA 20

4.3 MÉTODOS 20

3.1 Fases do estudo *20*

3.2 Análise dos resultados dos testes neuropsicológicos e psicológicos *27*

3.3Análise estatística *27*

5 RESULTADOS 30

5.1 População estudada *30*

6 DISCUSSÃO PRELIMINAR 33

7 ATIVIDADES DESENVOLVIDAS E PERSPECTIVAS PARA O PRÓXIMO PERÍODO 36

8 REFERÊNCIAS BIBLIOGRÁFICAS 42

APÊNDICES II

TERMO DE CONSENTIMENTO IV

APROVAÇÃO PELA COMISSÃO DE ÉTICA IX

Apêndices

LISTA DE ABREVIATURAS

- ANOVA — análise de variância para medidas repetidas
- CAPPesq — Comissão de Ética para Análise de Projetos de Pesquisa
- FAPESP — Fundação de Amparo à Pesquisa do Estado de São Paulo
- Pr. O. — Pré Operatório
- P.O — Pós Operatório
- USP — Universidade de São Paulo
- BTN — Bateria de testes neuropsicológicos
- POCD — Disfunção Cognitiva Pós Operatória
- TICS — Telephone Interview for Cognitive Status
- MMEE — Mini Exame do Estado Mental
- NSE — Enolase Específica do Neurônio
- APOE — Apolipoproteína
- BDI — Inventário de Depressão de Beck
- BDI — Inventário de Depressão de Beck
- VLT — Aprendizagem Verbal Visual (Visual Verbal Learning)
- TMT — Trail Making Test
- STROOP — Teste de Palavras coloridas

RESUMO

Carmona, M.J.C. - DISFUNÇÃO COGNITIVA APÓS CIRURGIA SOB ANESTESIA GERAL – Relatório Parcial - Processo FAPESP 09/54233-0. São Paulo, 2011.Faculdade de Medicina da Universidade de São Paulo.

INTRODUÇÃO: Um número significativo de pacientes submetidos a cirurgias com anestesia geral pode apresentar disfunção cognitiva por um período pós-operatório. Esta disfunção pode ser transitória e reversível, mas pode se tornar crônica em alguns casos, especialmente em idosos. A função cognitiva tende a melhorar e a se estabilizar após alguns meses do evento cirúrgico. A disfunção cognitiva pós-cirúrgica (POCD) envolve alterações de atenção, memória, velocidade do processamento de informações e alguns aspectos da função executiva. A POCD compreende desde transtornos sutis em quaisquer das áreas da cognição, até alterações incapacitantes como delirium e a demência. A etiologia da POCD, possivelmente é multifatorial e os fatores não estão bem definidos.
OBJETIVOS: Este estudo faz parte de um projeto de pesquisa aprovado pela Comissão de Ética Institucional (CAPPesq- processo n° 998/06) e que tem como objetivos primário avaliar o efeito da dexametasona, como fator de risco, sobre a incidência de disfunção cognitiva no pós-operatório de pacientes idosos submetidos a cirurgias sob anestesia geral. Como objetivos secundários objetiva-se investigar a associação entre alterações cognitivas e a presença do alelo ε4 do gene da apolipoproteína e de marcadores bioquímicos séricos para lesão neuronal, como a proteína S100β e a enolase específica do neurônio (NSE).
CASUÍSTICA E MÉTODOS: Estão sendo avaliados pacientes que preencham os critérios para inclusão do projeto de pesquisa (CAPPesq- processo n° 998/06): que tenham idade superior a 60 anos e submetidos a cirurgias de médio porte não cardíacas sob anestesia geral. Os pacientes são avaliados por testes psicológicos e neuropsicológicos para aferir a qualidade de vida, sintomas depressivos, o estado mental geral, a capacidade de aprendizagem, atenção e percepção, memória imediata, operacional e de evocação e habilidades executivas. A avaliação é realizada antes da cirurgia, e nos 3°, 7°, 21°, 90°, 180° dias pós-operatórios. Os resultados dos escores dos testes em cada grupo serão comparados através do teste de Friedmann, e entre os grupos será utilizado o teste de Kruskal Wallis. Porcentagens serão comparadas pelo do teste de Qui-Quadrado. Os dados serão expressos como mediana e intervalo de confiança de 95%. Serão considerados significativos resultados cujos valores p sejam menores que 0,05 nos testes estatísticos.
RESULTADOS: Até o momento, 155 pacientes preencheram os critérios do estudo mas a maioria não foi inclusa no protocolo por diversos motivos, principalmente relacionados à logística do estudo (suspensão da cirurgia, impossibilidade de avaliação neuropsicológica, mudança de conduta anestésica, dentre outros). Quarenta (40) pacientes foram efetivamente incluídos no protocolo. Destes, 10 completaram o estudo e os demais estão em seguimento. Medidas efetivas já foram tomadas para melhoria do recrutamento e da aderência dos pacientes.

INTRODUÇÃO

1 INTRODUÇÃO

Os progressivos avanços técnicos em cirurgia e anestesia têm possibilitado a intervenção em indivíduos cada vez mais idosos e com doenças mais graves. Com o aumento da expectativa de vida e de doenças relacionadas à longevidade, um número crescente de pacientes idosos são candidatos a procedimentos cirúrgicos. A melhoria dos índices de morbidade e mortalidade cirúrgicas, especialmente aqueles relacionados aos sistemas cardiovascular e respiratório, tem direcionado a investigação para a redução de complicações relacionadas a outros órgãos, dentre eles as complicações neurológicas e, particularmente, para a redução da incidência de disfunção cognitiva pós-operatória (POCD).

Os déficits cognitivos pós-operatórios podem prejudicar a recuperação cirúrgica, adiar o regresso às atividades normais e piorar a qualidade de vida pós-operatória. Newman et al (2010) realizaram estudo em que foi possível evidenciar a relação entre o funcionamento cognitivo e a qualidade de vida cinco anos após cirurgia cardíaca. Sendo mais freqüente no PO de cirurgia cardíaca, pacientes submetidos a cirurgias ortopédicas apresentam também alta incidência de POCD, seguido de pacientes submetidos a outros tipos de procedimentos cirúrgicos.

Para as cirurgias cardíacas, os déficits cognitivos são evidentes na diminuição da memória, da concentração, da compreenão verbal e do funcionamento executivo. As investigações conduzidas nesta área sugerem que as performances nos testes neuropsicológicos se encontrem afetadas de um modo geral, não ocorrendo deterioração de um domínio em particular. Contudo têm sido usados diversos métodos de avaliação e diferentes definições para disfunção cognitiva. Não existe portanto, uma definição universalmente aceita, o que pode explicar alguma variabilidade nos resultados relatados pelos diferentes estudos. (Kadoi Y, et al; 2006) De maneira semelhante, a duração das disfunções cognitivas associadas a cirurgias de grande e médio porte não se encontra

claramente delimitada, podendo se observar a sua ocorrência restrita a alguns dias ou semanas após a intervenção ou seu prolongamento permanente.

A disfunção cognitiva em cirurgia cardíaca é uma complicação frequente, apresentado, de acordo com os resultados da literatura, uma incidência variável entre 30 a 60% no pós operatório precoce e de cerca de um terço a 40% vários meses depois da intervenção. Nas últimas décadas, este tema tem sido objeto de numerosos estudos que têm incidido quase exclusivamente em pacientes submetidos a cirurgia cardíaca. Os dados relativos à incidência após cirurgia geral são muito limitados, sendo escasso o número de investigações específicas nestas cirurgias. A etiologia do déficit cognitivo após cirurgia geral é provavelmente multifatorial. Dentre os fatores de risco já descritos como relacionados à POCD citam-se idade, escolaridade, nível intelectual, funcionamento cognitivo prévio e a presença de co-morbidade com doença arteroscleróica, diabetes e insuficiência renal. Alguns estudos citados por Gaos et al (2005) referem-se ainda a um possível papel de fatores genéticos implicados na patogênese das alterações cognitivas após cirurgias cardíacas e, nomeadamente, à presença do alelo μ4 da apolipoproteína E (APO-μ4) e os que se relacionam com o procedimento cirúrgico. A presença de alterações neurocognitivas em doentes submetidos a cirurgias não cardíacas aponta como relevantes na gênese destas alterações fatores associados à cirurgia e/ou à anestesia. Após anestesia geral é comum ocorrer uma deterioração das funções cognitiva e psicomotora de curta duração. Esta é geralmente atribuída à metabolização incompleta de fármacos, que é tipicamente mais lenta nos idosos. Existem, até o momento, poucos estudos que permitam comparar os efeitos dos diversos anestésicos, e diferenciá-los de outros fatores potencialmente implicados na disfunção cognitiva pós operatória, como também os efeitos da doença ou da própria intervenção cirúrgica. A avaliação neurocognitiva por meio de estudos longitudinais favorece a comparação de resultados dos intra-individuais e é, provavelmente, mais fidedigna quanto à avaliação neuropsicológica. Segundo Selnes e Zenger (2007), ignorar os resultados da performance cognitiva pré cirúrgica corresponde, potencialmente, ao efeito de deprezar metada da informação disponível.

Além da elucidação dos fatores de risco relacionados à POCD, têm-se buscados meios para minimização de sua incidência. Dentre as técnicas anestésicas, embora a POCD seja descrita principalmente após anestesia geral, sabe-se que procedimentos sob anestesia regional também podem estar relacionados a este evento adverso. Tem-se procurado também avaliar o efeito de fármacos coadjuvantes para minimização desta complicação. O uso de corticóide pré-operatório diminui a dor pós-operatória, diminuindo também a necessidade de opióides, além de diminui a fadiga, a incidência de náusea e vômito, o período de recuperação e o tempo de retorno às atividades diárias no período pós-operatório, sendo freqüentemente utilizado como coadjuvante em anestesia. Sabendo-se que alguns determinantes de disfunção cognitiva pós-operatória são o uso de opióides no período pós-operatório, falta de atividade física, fadiga pós-operatória, a dor pós-operatória, a qualidade de vida, além de possível predisposição genética, questiona-se se o uso de corticóides poderia interferir na incidência de POCD de pacientes idosos submetidos a anestesia geral.

OBJETIVOS

2 OBJETIVOS

O objetivo primário deste estudo é a avaliação do efeito da dexametasona sobre a incidência de disfunção cognitiva no pós-operatório de pacientes idosos submetidos a cirurgias sob anestesia geral.

O objetivo secundário é a investigação da associação entre POCD e a presença do alelo ε4 do gene da apolipoproteína e de marcadores bioquímicos séricos para lesão neuronal, como a proteína S100β e a enolase específica do neurônio (NSE).

REVISÃO DA LITERATURA

3 REVISÃO DA LITERATURA

A memória, concentração, linguagem, compreensão e integração social são funções neuropsicológicas que podem apresentar comprometimento após intervenções cirúrgicas, caracterizando a chamada disfunção cognitiva pós-operatória (POCD), fato que pode ocorrer desde dias a semanas após a cirurgia e permanecer pelo resto da vida (Gao L et al, 2005; Kadoi Y et al, 2006). Muitas destas mudanças cognitivas são transitórias, com resolução entre seis semanas a seis meses após o procedimento, o que acaba minimizando a atenção médica sobre a importância dos déficits cognitivos transitórios para a qualidade de vida do paciente (Bryson GL et al, 2006).

Disfunção cognitiva pós-operatória (POCD) refere-se a uma deterioração da cognição ocorrida após um evento cirúrgico e anestésico (Hanning CD, 2005; Inzitari M et al, 2008) Estudos recentes têm mostrado uma série de correlações e fatores de risco para esta disfunção, embora ainda haja muito a ser elucidado sobre incidência, etiologia, prevenção e tratamento (Kadoi Y et al, 2006; Monk TG et al. 2008; Steinmetz J et al. 2009) .

Segundo Terri e pesquisadores (2008) um número significativo de pacientes idosos submetidos a cirurgias com anestesia geral pode apresentar disfunção cognitiva por um período pós-operatório. Esta disfunção pode ser transitória e reversível, mas pode se tornar crônica em alguns casos, especialmente em idosos. A função cognitiva tende a melhorar e a se estabilizar após alguns meses. A disfunção cognitiva pós-cirúrgica (POCD) envolve alterações de atenção, memória e alguns aspectos da função executiva (Dijkstra JB and Jolles J, 2002; Hanning CD, 2005).

A POCD compreende desde transtornos sutis em quaisquer das áreas da cognição, até alterações incapacitantes como delirium e a demência. A etiologia da

POCD, possivelmente é multifatorial e os fatores não estão bem definidos (Stern Y, 2002; Rasmussen LS et al. 2006). A POCD representa uma complicação no pós-operatório, e, embora os avanços tecnológicos na última década em cirurgias e anestesias conseguiram levar a uma diminuição da mortalidade e da taxa de morbidade associada a esses procedimentos, a incidência de POCD permanece relativamente alta. Pacientes com POCD apresentam um aumento de mortalidade no primeiro ano após a cirurgia, principalmente para cirurgias cardíacas e de grande porte. Os fatores de risco para a DPCO podem ser divididos em idade, comorbidades, fatores relacionados propriamente com a cirurgia e anestesia. Associados a POCD estão os abusos de substâncias tóxicas, álcool, doenças pré-existentes, distúrbios psiquiátricos e neurológicos. Estudos demonstram que o risco de desenvolvimento da POCD aumenta com a idade (Sear JW, 2003; Selwood A et al., 2004; Rohan D et al, 2005)

3.1 FATORES DE RISCO PARA POCD

A principal etiologia das disfunções cognitivas pós-operatória permanece em aberto, reforçando a hipótese de um problema multifatorial (Lelis RGB & Auler Jr JOC, 2004; Gao L et al, 2005; Tagarakis GI et al, 2007)

Entre os fatores de risco envolvidos estão os pré-operatórios (idade, escolaridade, doenças prévias), intra operatórios (liberação de êmbolos, duração do procedimento, pressão arterial, temperatura, etc) e pós-operatórios (temperatura, recuperação do procedimento) (Gao L et al, 2005). Além destes possíveis fatores de risco, alguns pesquisadores relataram influência genética sobre esse desfecho cirúrgico, merecendo especial destaque a presença do alelo e4 da apolipoproteína E (Ishida K et al, 2003; Kleindienst A et al, 2006)

3.3 FATORES DE RISCO PRÉ OPERATÓRIOS

Entre os fatores pré-operatórios a idade representa o fator de risco demográfico menos contestado para o declínio cognitivo, embora ainda com causa

injustificada. Aterosclerose progressiva associada a doença cerebrovascular oculta e fatores intrinsicamente relacionados ao risco de embolização parecem ser a explicação mais aceitável para a POCD associada ao aumento da idade (Johnson T et al, 2002; Inzitari M et al, 2008) Idosos são predispostos a alteração vascular e da auto regulação do fluxo cerebral (Di Carlo et al, 2001), apresentam também uma resposta anormal a fármacos e uma redução natural do nível cognitivo, o que associado a uma pequena diminuição cognitiva no pós-operatório pode acarretar num significante impacto sobre a qualidade de vida (Di Carlo et al, 2001; Kadoi Y et al, 2006)

Um estudo avaliou o fator idade após a alta hospitalar nos Estados Unidos, e em seus pacientes jovens (117-36%), pacientes de meia idade (112-30,4%) e idosos (138-41,4%), quanto a disfunção cognitiva após uma cirurgia, houve diferença significativa entre todos os grupos de idade. Em três meses após a cirurgia, a POCD esteve presente em 16 (5,7%) dos jovens, 19 (5,6%) de meia idade, e 39 (12,7%) pacientes idosos. A prevalência de disfunção cognitiva foi significativamente maior em pacientes idosos em comparação aos outros grupos de pacientes (Terri GM et al., 2008).

Um estudo internacional sobre POCD em pacientes idosos (média de idade de 68 anos, com intervalo de 60-81 anos) que sofreram cirurgias não cardíacas demonstrou uma incidência de 26% de POCD na primeira semana após a cirurgia, com 10% apresentando uma persistência da POCD durante os três meses pós-cirúrgicos (Johnson T et al, 2002)

Outro fator pré-operatório que merece importante destaque na patogênese da POCD é o grau de escolaridade. Não se conhece plenamente o modo como um maior grau de escolaridade implicaria em maior reserva cognitiva, mas uma hipótese que explicaria essa associação está baseada no fato de que a escolaridade aumenta a densidade sináptica no neocórtex, aumentando a comunicação neuronal e minimizando os sinais de comprometimento cognitivo e funcional (Ille R et al, 2007). História de comorbidades como diabete Mellito, hipertensão arterial sistêmica e insuficiência renal crônica são fatores pré-operatórios que igualmente a idade e

escolaridade estão relacionados à desfechos neurológicos pós-operatórios prejudicados (Nötzold A et al, 2006).

3.4 FATORES DE RISCO INTRA OPERATÓRIOS

Os fatores de risco intra-operatórios como a formação de êmbolos, cuja gênese estaria em ateromas da parede da aorta, agregados plaquetários, bolhas de ar oriundas do oxigenador e/ou das câmaras cardíacas podem ser a causa primária da lesão encefálica ou do agravamento de lesões preexistentes. Estes se subdividem em micro e macroêmbolos, sendo os primeiros mais relevantes para surgimento da POCD (Lelis RGB & Auler Jr JOC, 2004) A duração do procedimento cirúrgico também está relacionada à maior obstrução microvascular por êmbolos o que sugere uma relação entre estes fatores e o desenvolvimento de disfunção cognitiva (Hogue CW Jr et al, 2006) O valor da pressão arterial média durante o procedimento cirúrgico também é hipótese de disfunção cognitiva, sendo a hipotensão intra-operatória e consequentemente hipoperfusão cerebral uma causa potencial para comprometimento neurológico (Grocott HP et al, 2005). Outro fator que pode contribuir para a lesão neurológica é a resposta inflamatória, a hiperglicemia, principalmente nas cirurgias cardíacas e o efeito da temperatura durante o procedimento cirúrgico (Grocott HP & Yoshitani K, 2007)

A compreensão dos fatores relacionados ao tratamento cirúrgico dos idosos representa um desafio a uma equipe médica. Estes pacientes apresentam uma diminuição das reservas funcionais de diversos órgãos e sistemas e, como consequência, toleram muito pouco as exigências representadas pelo estresse anestésico- cirúrgico. Alguns fatores relacionados são a diminuição da capacidade homeostática autonômica, funções imunológicas prejudicadas e redução da capacidade aeróbica (Linstedt U et al. 2002; Bekker AY et al. 2003)

Terri GM e colaboradores (2008) evidenciaram em pesquisa a alta incidência de POCD em pacientes idosos submetidos a cirurgia não cardíaca grave, 54,3% em 6 semanas e 46,1% em 1 ano. Outro estudo internacional relatou POCD em 25,8% a prevalência de disfunção em uma semana e 9,9% após três meses. Pravat

K e seus colegas (2009) encontraram uma prevalência de 41,4% e 12,7% de POCD após uma semana e três meses respectivamente.

3.5 FATORES DE RISCO GENÉTICOS PARA POCD

Embora um grande número de fatores seja capaz de predizer o risco de POCD, os fatores genéticos contribuem em cerca de 10% a 40% para que estes episódios ocorram, porém pesquisas indicam que outros fatores podem influenciar esse declínio (Kadoi Y et al, 2006) Entre os possíveis polimorfismos genéticos candidatos a fatores de risco para a POCD, merece destaque especial a presença do alelo e4 da apolipoproteína E (Apo-e4). Esse polimorfismo é reconhecido e bem estabelecido como fator de risco para doença de Alzheimer e por desordens neurodegenerativas relacionadas. Na análise sobre a associação da presença de apo-e4 com POCD, os resultados são controversos e justificam a necessidade de estudo dos demais fatores etiológicos relacionados ao desenvolvimento da POCD (Rasmussen LS et al, 2000)

Alguns pesquisadores relatam influência genética sobre o desfecho cirúrgico, merecendo especial destaque a presença do alelo e4 da apolipoproteína E. A identificação de um suscetível genótipo pode permitir a identificação pré-operatória em pacientes propensos a desenvolver POCD, da mesma maneira um biomarcador de plasma poderá identificar os pacientes propensos a desenvolver POCD, permitindo uma intervenção precoce pré-cirúrgica (Ballard CG et al, 2004; Abilstrom H et al, 2004; Tagarakis GI et al, 2007)

Estudos indicam o aumento da concentração sanguínea da enolase específica do neurônio (NSE) e proteína S-100B após cirurgias acarretando, mesmo que temporariamente em prejuízos cognitivos identificados em testes neuropsicológicos, assim como em marcadores bioquímicos. NSE parece ser o marcador bioquímico mais útil na predição de disfunção cognitiva após intervenções cirúrgicas. Estudos levantam a hipótese de que APOE4 pode ser responsável pela disfunção cognitiva pós-operatória como consequência de um aumento significativo da resposta inflamatória (Rasmussen LS et al. 2000; Silbert BS et al, 2008)

A APOE é uma constituinte das lipoproteínas VLDL, HDL e desempenha um papel chave no transporte e metabolismo de colesterol e triglicérides. Esta proteína, adicionalmente, está relacionada com a proteção neuronal. Três principais variantes da APOE são encontradas na população humana sendo resultantes da mudança de um único aminoácido. Os alelos que codificam essas variantes são denominados APOE e2, APOE e3 e APOE e4.

A participação de 394 pacientes em um estudo para POCD evidenciou 24% destes com o alelo da APOE 4. Os polimorfismos da APOE estavam em equilíbrio de Hardy-Weinberg, e com exatidão genotípica de 100% para APOE 4. Entre os 350 (89%) pacientes que retornaram para o teste em seis semanas, a POCD foi percebida em 56% com o alelo APOE4 comparado com 52,6% naqueles sem a APOE4. Neste mesmo estudo não houve associação significativa entre o nível de biomarcadores e APOE4. Concluem que o estudo é uma primeira análise combinada da POCD após cirurgias não cardíacas, o genótipo APOE4 e biomarcadores plasmáticos de lesão cerebral e que o estudo, apesar de uma sólida amostra em tamanho, não foi possível encontrar uma associação entre declínio cognitivo e genótipo APOE4, acreditando que da mesma forma não poderiam encontrar associação entre o pós-operatório e NSE, e os níveis de S100B e o declínio cognitivo (Rasmussen LS et al, 2000).

Abildstrom e colaboradores (2004) estudaram a relação entre APOE4 e disfunção cognitiva não cardíaca em 972 pacientes e não encontraram diferença na incidência de POCD (10,3% vs 9,9%) entre os pacientes com e sem alelo APOE4. Da mesma forma, não houve associação do APOE4 com declínio cognitivo pós-operatório no estudo de 350 pacientes submetidos a cirurgias não cardíacas, bem como num estudo de 513 pacientes submetidos a cirurgias de revascularização (Monk TG et al, 2008). Porém estudos em pacientes de cirurgias cardíacas notou-se uma sutil alteração na cognição dos pacientes com o alelo APOE4 (Kadoi Y et al, 2006). Em um estudo sobre disfunção cognitiva após cirurgia cardíaca, houve uma incidência de 20,6% e a associação alélica (Ishida K et al, 2003). Na população para cirurgias não cardíacas, os dados sobre a resposta inflamatória e os biomarcadores de

plasma são raros. A avaliação dos biomarcadores plasmáticos de lesão cerebral, NSE e S100B, também produziram resultados pouco significativos. NSE é geralmente considerado um marcador de lesão cerebral, enquanto S100B é um marcador de astrocísticos gliais. Rasmussen et al (2000) estudaram 65 pacientes idosos submetidos a cirurgia abdominal e não encontraram associação entre os níveis de NSE e S100B e declínio cognitivo. Linstedt el al. Estudaram 120 pacientes submetidos a cirurgias não cardíacas e encontrou uma associação de S100B, mas não de NSE com disfunção cognitiva após uma semana das cirurgias. Em estudos sobre biomarcadores e POCD, os índices de S100B e NSE aumentaram ao longo do tempo pós-cirúrgico sugerindo uma resposta inflamatória própria do quadro pós-cirúrgico, mas não sugerem a presença de lesão neuronal, os níveis dos biomarcadores voltam a se estabilizar, na maioria dos casos após 18h (Kleindienst A & Bullock MR, 2006).

Alguns estudos revelam que o tempo mais adequado para a coleta de sangue para NSE é 36 horas após a cirurgia, confirmando que as concentrações máximas da proteína S-100B e NSE ocorrem logo após RM. Apontando assim evidências de que existe uma correlação negativa entre o aumento precoce em NSE e disfunção cognitiva (Rasmussen LS et al. 2003; Zhou W et al. 2008)

Alguns trabalhos, citados por Rasmussen et al. (2005), referem-se a um possível papel dos fatores genéticos implicados na patogênese das alterações cognitivas pós cirúrgicas e a presença do alelo 4 da apolipoproteína E. Este alelo, reconhecidamente associado com a demência de Alzheimer, dará ao seu portador uma propensão genética, tornando o paciente mais vulnerável a qualquer tipo de lesão neuronal.

3.6 FÁRMACOS E ANESTÉSICOS

Observa-se uma lacuna na literatura, principalmente no que se refere a avaliação cognitiva de longo prazo, com estudos padronizados, norteando-se por parâmetros rigorosos para se fazer otimizar o uso adequado de anestésicos e procedimentos cirúrgicos para a minimização de qualquer déficit seja ele mental ou físico.

Existe a necessidade de mais estudos específicos sobre técnicas anestésicas, e fármacos usados no transoperatório que possam comprovar realmente a influência destes nas alterações cognitivas de curto, médio e longo prazo. Porém, com o conhecimento dos fatores de risco, é possível predizer aqueles indivíduos que terão uma maior chance de desenvolvimento de POCD e, dessa forma, proporcionar mecanismos protetores, reduzindo sequelas e evitando a instalação de lesões cerebrais irreversíveis. A terapia neuroprotetora procura minimizar a ativação de vias tóxicas e incrementar os mecanismos endógenos de proteção (Gao L et al, 2005)

Alguns medicamentos que aumentam o risco para a disfunção cognitiva são os anticolinérgicos, benzodiazepínicos, sedativos, antidepressivos e antiparkissonianos (Sear JW, 2003; Bekker AY & Weeks EJ, 2003; Rohan D et al. 2005; Rasmussen LS 2006).

Estudos sobre os possíveis danos causados após intervenções cirúrgicas em anestesia geral se faz necessário, principalmente quando se planeja uma cirurgia e o tipo de anestesia. As alterações de cognição ao se instalarem podem trazer perdas de ordem psicoemocionais, sociais e econômicas. Importante se faz ressaltar que a anestesia não poderá ser responsabilizada como fator primordial para a POCD antes de muitos estudos de todos os fatores de risco envolvidos em um procedimento cirúrgico (Linstedt U et al. 2002; Praticò C et al. 2005) .

Rasmussen et al (2006) pesquisaram a incidência da disfunção cognitiva em idosos após cirurgias não cardíacas sob anestesia geral e regional. Em 188 pacientes com idade média de 60 anos. Pesquisaram a hipótese de que a incidência de POCD seria menor com as anestesias regionais ao invés das com anestesia geral. A função cognitiva foi avaliada por meio de quatro testes neuropsicológicos realizados no pré e pós-operatórios. Após sete dias da cirurgia a POCD foi encontrada em 37/188 pacientes (19,7% [14,3-26,1%]) após anestesia geral e em 22/176 (12,5% [8,0-18,3%]) após anestesia regional. Depois de três meses, a POCD esteve presente em 25/175 pacientes (14,3%, % [9,5-20,4]) após anestesia geral versus 23/165 (13,9%, [9,0-20,2%]) após anestesia regional. A incidência de POCD após uma semana foi significativamente maior após anestesia geral.

Os corticosteróides de modo geral, e em particular, os glicocorticóides, constituem um grupo de fármacos muito utilizados na atualidade. Em virtude de seu amplo espectro de ação e por serem os mais potentes antiinflamatórios existentes, encontram uso praticamente em todas as especialidades (Rasmussen et al, 2002; Fietta et al, 2009; Chrousos, & Kino, 2009; Marques et al, 2009).

A administração de antiinflamatórios antes de procedimentos cirúrgicos atenuam dores pós-operatórias, fadiga, náuseas e vômitos acelerando a recuperação do paciente oferecendo oportunidade do retorno às atividades diárias em tempo reduzido. A dexametasona é um medicamento pertencente à classe dos corticosteróides, atuando no controle da velocidade de sínteses de proteínas. Por sua ação antiinflamatória e imunossupressora, pode prevenir ou suprimir processos inflamatórios de várias naturezas e se administrada no período pré-operatório fará com que o paciente tenha benefícios em seu período de recuperação amenizando os sintomas desagradáveis durante o período pós-cirúrgico. (Iohom et al, 2004; Vaurio et al, 2006; Koster et al, 2008).

A preocupação com a POCD e quais implicadores para o seu desencadeamento já existe, seu diagnóstico requer uma avaliação neuropsicológica pré e pós-cirúrgica, razão pela qual se defende a avaliação cognitiva em todos os pacientes que estarão sujeitos a cirurgias eletivas. Existe também a necessidade de investigar quais fatores de risco desencadeiam a POCD para evitar o comprometimento do paciente em suas funções corticais superiores comprometendo consequentemente sua qualidade de vida.

CASUÍSTICA E MÉTODOS

4 CASUÍSTICA E MÉTODOS

4.1 Projeto de pesquisa

O protocolo do estudo foi aprovado pela Comissão de Ética Médica do Hospital das Clínicas da Faculdade de Medicina da Universidade de São Paulo (CAPPesq - projeto de pesquisa nº: 998/06) e recebeu auxílio à pesquisa do Fundo de Amparo à Pesquisa do Estado de São Paulo (Processo FAPESP nº: 09/54233-0).

Serão estudados 300 pacientes idosos de ambos os sexos submetidos à anestesia geral para procedimentos cirúrgicos.

São aplicados testes de qualidade de vida, depressão e bateria neuropsicológica para avaliar o estado mental geral, aprendizagem, atenção, percepção visuoespacial, memória imediata, operacional e de evocação e habilidades executivas, incluindo velocidade de processamento. Esta bateria define o índice cognitivo da estabilidade (medida longitudinal do status cognitivo em avaliações repetidas que detectam mudanças estatisticamente significantes no funcionamento do sistema nervoso central). A avaliação neuropsicológica será realiza na noite anterior à cirurgia e nos dias pós-operatórios 3, 7, 21, 90 e 180, sendo que os profissionais responsáveis pelos testes neuropsicológicos não terão conhecimentos dos grupos aos quais pertencem os pacientes, quanto a presença do alelo do gene da apolipoproteína e de marcadores bioquímicos séricos para lesão neuronal, como a proteína S100β e a enolase específica do neurônio (NSE).

4.1.1 Critérios de seleção dos pacientes

Critérios de inclusão: pacientes acima de 60 anos, submetido a cirurgia para herniorrafia inguinal, operação de Nissen, colecistectomia ou outras cirurgias de médio porte sob anestesia geral com permanência hospitalar prevista de até 72 horas.

Critérios de exclusão: idade inferior a 60 anos, história pregressa de doença cerebral ou demência, outras doenças psiquiátricas que afetem a cognição,

falta de domínio da língua portuguesa, uso de corticóide ou de opióde no pré-operatório.

4.1.1.1 Termo de consentimento livre e esclarecido

Os pacientes que preenchem os critérios de inclusão e aceitam participar do estudo são submetidos aos testes neuropsicológicos. Após definição da indicação cirúrgica e avaliação dos critérios de inclusão e exclusão, os pacientes são convidados a participar da pesquisa. Após esclarecimentos em relação aos objetivos gerais do estudo, assinam o termo de consentimento livre e esclarecido (apêndice 1).

O paciente assina o termo de consentimento em duas vias, sendo que uma cópia fica com o paciente e outra com o pesquisador.

4.2 Casuística

O estudo prevê a inclusão de 300 pacientes e até o momento foram recrutados 155 pacientes, sendo destes apenas 40 foram randomizados e inclusos no protocolo.

As fases pós cirúrgicas deixaram de ser realizadas quando o sujeito da amostra, mesmo convidado a comparecer para a avaliação e justificou o motivo para o não comparecimento, deixando desta forma algumas fases incompletas mas dando continuidade a avaliação e permanecendo no protocolo de pesquisa.

4.3 Métodos

4.3.1 Fases do estudo

O estudo realizou-se nos períodos pré, intra e pós-operatórios. As avaliações neuropsicológicas são realizadas nos períodos pré e pós operatórios.

Período pré-operatório

No dia anterior à cirurgia os pacientes incluídos no estudo foram avaliados com uma BTN. A avaliação pré cirúrgica foi realizada pela maioria dos pacientes, deixando de ser realizada apenas pelos sujeitos que não quiseram participar da pesquisa após o convite ou aceitaram participar mas apresentavam algum quesito dos critérios de exclusão.

Primeiro dia pré-operatório (1º Pr.O.)

No dia anterior a cirurgia os pacientes inclusos ao projeto de pesquisa são avaliados em suas funções cognitivas: memória, atenção, raciocínio, linguagem, executiva e nos aspectos emocionais: depressão e qualidade de vida.

O funcionamento mental é a função cognitiva como um todo e primordial para o funcionamento do raciocínio do indivíduo. O Estado mental é avaliado com um teste padrão MEEM (mini exame do estado mental) neste teste são avaliados raciocínio, orientação espaço temporal, memória e escolaridade. Para a inclusão do paciente ao estudo o ponto de corte deste instrumento é considerado para dois tipos de escolaridade – até 4 anos de estudo formal acadêmico e com mais de 4 anos de escolaridade. Com ponto de corte de 18 para os menos escolarizados e com mais de 23 pontos para indivíduos com maior nível de escolaridade. Em relação a função de atenção os testes são administrados verbalmente e graficamente. São cronometrados e avaliados individualmente e comparados com resultados em escalas de acordo com idade, gênero e educação de uma amostra padronizada em população americana. É relação ao tempo para a execução da tarefa é esperado que o indivíduo não exceda um tempo maior que > 3' (três minutos) . A memória é avaliada imediatamente após a evocação da linguagem e depois de vinte minutos para a memória tardia, juntamente com a avaliação da função executiva para a busca de estratégias, planejamento e organização do conteúdo armazenado. A linguagem é avaliada constantemente a partir do discurso do paciente, eloquência, fluência e curso do pensamento, além de uma avaliação padrão através de teste padronizado para idade,

gênero e escolaridade. O tônus atencional é avaliado durante a aplicação da BTN e por instrumentos padrão.

1-Informações Sócio-demográficas

Serão investigadas as informações sobre idade, sexo, escolaridade, estado civil, ocupação, e demais informações relevantes para compor o perfil sociodemográfico da amostra, bem como a compatibilidade com os critérios de exclusão e inclusão.

2-Versão Brasileira do Questionário de Qualidade de Vida SF-36

O SF-36 investiga condições relacionadas à saúde, atividades de vida diária, produtividade, problemas emocionais, relacionamentos, motivação e outras condições que traçam o perfil da qualidade de vida a partir da percepção do próprio sujeito.

3- Bateria de testes neuropsicológicos

A bateria neuropsicológica proposta neste estudo seguirá o protocolo sugerido pelo Estudo Internacional de Disfunção Cognitiva Pós-Cirúrgica (do inglês, International Study of Postoperative Cognitive Disfunction - ISPOCD) para avaliar as funções cognitivas superiores: memória, executiva, atenção e linguagem com a finalidade de detectar possíveis sutis disfunções neuropsicológicas em pacientes idosos após cirurgia com anestesia geral ou regional (Rasmussen et al., 1999, Rasmussen et al., 2001 e Rasmussen et al., 2002).

4-Entrevista Telefônica para o Status Cognitivo (Telephone Interview for Cognitive Status - TICS)

É um teste padronizado para avaliação do funcionamento neuropsicológico, é utilizado quando a avaliação de rastreio cognitivo realizada pessoalmente é impraticável ou ineficiente, como, por exemplo, em pesquisas epidemiológicas de grandes populações ou com pacientes que estão incapacitados de comparecerem ao retorno clínico. É muito útil também para avaliar pessoas com incapacidade para ler ou escrever, pois pode ser aplicado pessoalmente através de

entrevista, exigindo apenas capacidade de compreensão verbal. (Ferrucci et al., 1998, De Jager et al., 2003, Barber & Stott, 2004, Musselwhite et al., 2006 e Dal Forno et al., 2006).

O teste consiste em um roteiro de entrevista com onze itens que avaliam as habilidades de orientação espacial e temporal, controle mental, memória, informação geral, linguagem e cálculos conforme detalhado em anexo.

5- Inventário de Depressão de Beck – BDI

Inventário de Depressão de Beck (BDI) consta de 21 questões que exploram sintomas de depressão numa escala de 0 a 4, na qual zero corresponde a ausência de sintomas e quatro corresponde ao nível máximo da sintomatologia. Um total de 18 pontos tem sido considerado indicativo da presença de sintomatologia depressiva. Foi desenvolvido originariamente por Beck, Ward, Mendelson, Mock e Erbaugh (1961) estimativa de fidedignidade que variou entre 0,79 e 0,90 a partir de seis amostras psiquiátricas (Beck & Steer, 1993). É um instrumento adaptado à população brasileira e particularmente adequado para uso com pacientes psiquiátricos e tem sido também amplamente usado na clínica e em pesquisa com pacientes não psiquiátricos e na população geral (Cunha, 2001).

6- Testes Neuropsicológicos

6.1- Mini Exame do Estado Mental- MMEE é um instrumento para breve *screening* que permite avaliar e quantificar o cognitivo do paciente. A pontuação será de acordo com a padronização brasileira com pontos de corte para ensino fundamental maior ou igual a 18. Ensino médio ou superior será maior ou igual a 26.

6.2-Aprendizagem Verbal Visual (Visual Verbal Learning – VLT

Será apresentada uma lista de 15 palavras para ser memorizada e recordada em três tentativas sucessivas (VLT-A), com evocação tardia após 15 a 25 minutos (VLT-D). Serão avaliados os números de palavras recordadas e o número de erros cometidos para cada apresentação.

6.3-Subteste Digit Symbol do Wechsler de Inteligência- Scale-Revised- Este teste afere a memória de curto prazo, habilidade visuoespacial e atenção. É uma tarefa gráfica realizada em 180 segundos. O indivíduo tem que reproduzir os

símbolos exemplificados em uma tarjeta nos espaços abaixo do número correspondente.

6.4-Trail Making Test (TMT)- Este teste é composto por duas partes. Na parte A, o sujeito deve traçar linhas conectando consecutivamente círculos numerados. Na parte B, o sujeito deve traçar linhas conectando alternadamente círculos com letras e números em uma sequência ordenada. O teste envolve (Parte A) rastreio visual complexo e velocidade motora e, na parte B, processos executivos. Entre os processos executivos, a capacidade inibitória e a alternância cognitiva parecem ser aqueles mais envolvidos na realização da parte B. Serão avaliados o tempo dispendido e o número de erros cometidos em cada parte.

6.5-Teste de Palavras coloridas de Stroop- (Stroop colours Word Test) Serão avaliados, o tempo dispendido e o número de erros em cada cartão. Consiste na apresentação de três lâminas ao sujeito. Na primeira lâmina o sujeito deve verbalizar os nomes das cores impressas com tinta preta. Na segunda, a verbalização é feita da cor que estão preenchidos retângulos, na mesma disposição das palavras da lâmina anterior. A terceira consiste em verbalizar as cores impressas em detrimento da palavra escrita. Avalia atenção seletiva, capacidade inibitória e flexibilidade mental.

Randomização: Utilizou-se o método de randomização em bloco de 100 sujeitos. Considerando a possibilidade de perda de pacientes após a randomização optou-se pela randomização de 4 blocos de 100 pacientes. Em cada bloco, os pacientes foram distribuídos em quatro grupos:

- Controle + anestesia profunda
- Controle + anestesia superficial
- Dexametasona + anestesia profunda
- Dexametasona + anestesia superficial

O grupo randomizado foi colocado em envelope opaco e selado, sequencialmente numerados, sendo a abertura do envelope realizada após admissão do paciente à sala de operações. Apenas o anestesiologista tem conhecimento do grupo ao qual o

paciente pertence e esta informação não é compartilhada com a equipe de neuropsicólogos e com técnicos envolvidos nos exames laboratoriais.

O **GRUPO CONTROLE** não recebe nenhum fármaco adicional à técnica anestésica preconizada e o **GRUPO DEXAMETASONA** deve receber 8 mg de dexametasona por via venosa antes do início da indução anestésica.

O **ANESTESIA SUPERFICIAL** deve ser mantido no intraoperatório com índice bispectral entre 45 e 55 e o grupo **ANESTESIA PROFUNDA**) com BIS entre ou anestesia profunda 35 e 45). Ambos níveis são aceitáveis para anestesia geral e são atualmente usados por anestesiologistas.

Período Intra-operatório

Após admissão na sala de operações, os pacientes são monitorados com oximetria de pulso, eletrocardiografia contínua, pressão arterial não invasiva e índice bispectral, seguido de punção venosa periférica com cateter de calibre 18G.

Antes da indução anestésica, serão colhidas duas amostras de sangue de para determinação do perfil genotípico da Apoε e dosagem de S100β e NSE. A dosagem de S100β e NSE será repetida após 12 horas do final da cirurgia. As amostras serão processadas no Laboratório de Biologia Molecular do InCor (APOε4) e Laboratório de Neurociências da UFRGS (S100β e NSE).

A anestesia geral será realizada com Propofol (2-3 mg/kg), cisatracúrio (0,10 mg/kg), e fentanil (2-3 μg/kg) para indução anestésica. A manutenção anestésica subseqüente será com propofol em infusão alvo-controlada conforme valor do índice bispectral, remifentanil (0,10 a 1,0 μg/kg/min) e cisatracúrio (doses suplementares serão administradas se necessário para manutenção de anestesia cirúrgica). A ventilação será controlada mecanicamente para manter a pressão de dióxido de carbono próximo a 35 mmHg, utilizando-se FiO_2 de 60%. Solução de ringer simples será administrada durante a cirurgia para reposição volêmica. A analgesia pós-operatória será realizada com Dipirona na dose de 30mg/kg ao final do

procedimento cirúrgico e Tramadol na dose de 50-100mg no período pós-operatório, se necessário.

O índice bispectral (BIS) mede a atividade cerebral cortical (hipnose) numa escala de 0 a 100, onde 100 representa o estado "acordado" e 0 representa um eletroencefalograma isoelétrico. Medidas abaixo de 70 estão relacionadas à inconsciência e ausência de memória necessária para a anestesia geral. Neste estudo, os participantes serão randomizados em 2 grupos: anestesia superficial (índice bispectral entre 45 e 55) and anestesia profunda (índice bispectral entre 35 e 45). Ambos os níveis são aceitáveis para anestesia geral e são atualmente usados por anestesiologistas. Para monitoração do BIS devem ser seguidas as seguintes normas: 1) A pele da região onde o monitor vai ser aplicado deve estar limpa com álcool e seca. 2) Aplicar o sensor na região frontal do paciente. Colocar o círculo número 1 no centro aproximadamente 2 cm acima da ponte nasal. Colocar o círculo número 3 na área temporal. Aplicar pressão em torno do sensor (incluindo as áreas entre os círculos) para garantir a adesão adequada. Em seguida, pressionar os círculos 1, 2 e 3 por 5 segundos para assegurar o contato dos eletrodos à pele. 3) Conectar o sensor ao cabo do aparelho de índice bispectral.

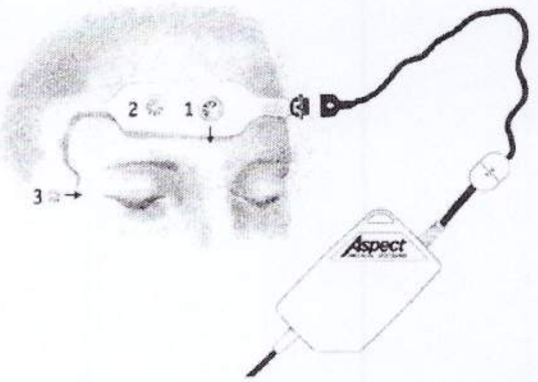

Período pós-operatório imediato

Este período pós operatório consiste em 4 fases. Após a cirurgia o paciente é avaliado com TICS instrumento para avaliação via telefone. Realizado em duas sub fases, uma no terceiro dia pós cirurgico e outrs no sétimo dia. A avaliação da disfunção cognitiva será realizada por meio de testes neuropsicológicos, padronizados especificamente para avaliar as funções cognitivas superiores, e questionários para avaliar as características sócio-demográficas, questionamentos sobre a percepção pessoal da saúde do paciente. Os profissionais responsáveis pela avaliação neuropsicológica aplicarão os testes no pré-operatório, na noite anterior à cirurgia, antes da administração de medicações pré-anestésicas ou sedativas e no 3°, 7°, 21°, 90° e 180° dias pós-operatórios.

4. 3.2 Análise dos resultados dos testes neuropsicológicos e psicológicos

As medidas mais utilizadas para aferir alterações cognitivas têm sido as baterias de testes neuropsicológicos. São técnicas que consistem em questionários de avaliação psicométrica ou testes de execução prática. A seleção dos instrumentos é importante devendo ser considerada a sensibilidade dos instrumentos para as alterações associadas ao pós-operatório. Para a pesquisa em questão serão utilizados os seguintes instrumentos.

A análise dos resultados será comparativa, de acordo com a idade, nível de educação e gênero por tabelas padronizadas de cada instrumento, adaptadas e validades para a população brasileira. Será considerada uma incidência esperada de déficit cognitivo pós-operatório de 10%, com confiança de 95%.

4.3.3 Análise estatística

Os dados serão analisados usando os programas estatísticos SAS (SAS Institute inc., Cary, NC, EUA) e SPSS (SPSS inc., Chicago, IL, EUA), considerando-se significativo $p<0,05$.

Os dados serão analisados utilizando-se o software de análise estatística SPSS versão 10. Os dados demográficos como idade, peso, altura serão comparados nos quatro grupos através de análise de variância univariada seguida de teste de comparações múltiplas se aplicável. As variáveis colhidas através de testes neuropsicológicos aplicados antes e depois do procedimento cirúrgico são de natureza qualitativa ou ordinal, sendo atribuídos resultados numéricos e os resultados finais dos testes serão expressos em escores. Os resultados dos escores dos testes em cada grupo serão comparados através do teste de Friedmann, e entre os grupos será utilizado o teste de Kruskal Wallis. Mann Whitney para comparações da evolução do sujeito Porcentagens serão comparadas através do teste de Qui-Quadrado Os dados serão expressos como mediana e intervalo de confiança de 95%. Serão considerados significativos resultados cujos valores p sejam menores que 0,05 nos testes estatísticos.

RESULTADOS

5 RESULTADOS

5.1 População estudada

Até o momento, 155 pacientes preencheram os critérios de inclusão mas a maioria não foi inclusa no protocolo por diversos motivos, principalmente relacionados à logística do estudo (suspensão da cirurgia, impossibilidade de avaliação neuropsicológica, mudança de conduta anestésica, dentre outros). Quarenta (40) pacientes foram efetivamente incluídos no protocolo. Destes, 10 completaram o estudo e os demais estão em seguimento.

Todos os pacientes efetivamente inclusos no estudo completaram a fase de avaliação pré-operatória (anestésica e neuropsicológica) e foram submetidos à cirurgia sob anestesia geral. A randomização para os grupos de estudo foi realizada na sala cirúrgica, imediatamente antes da indução da anestesia geral e os neuropsicólogos e técnicos de laboratório envolvidos no estudo não tem conhecimento do grupo ao qual os pacientes pertencem.

Dentre os indivíduos randomizados, 15% são do sexo masculino e 85% do sexo feminino, sendo a idade média de 68,8 anos e a média de idade dos pacientes randomizados foi de 68,08 anos (desvio padrão de 5,06 anos), com mediana de 67 anos. Em relação à escolaridade, o tempo médio de anos de estudo foi de 6,77 (desvio padrão de 6,85 anos), com mediana de 5 anos. Dentre os pacientes randomizados, 32 eram do sexo feminino e 8 do sexo masculino.

A maior prevalência foi de cirurgias de colecistectomia (66,67%). Com menor proporção estão as cirurgias: Esofagectomia Distal, Fechamento de Enterostomia, Hernioplastia, Hernioplastia Epigástrica, Hernioplastia Inguinal, Mastoidectomia, Radical, Paratireidectomia, Paratireoidectomia Radical, Reconstrução T. Intestinal conforme exposto na tabela 1.

Tabela 1. Tipos de cirurgias realizadas

Tipo de Cirurgia	Número de indivíduos	%
Colecistectomia	26	66,67
Hernioplastia Epigástriga	1	2,56
Hernioplastia Inguinal	1	2,56
Hernioplastia	1	2,56
Tireoidectomia Parcial	3	6,13
Tratamento Cirurgico Megaesofago	2	5,13
Tireoidectomia Total	1	2,56
Tratamento Refl. Gástrico	3	7,69
Esofágico	1	2,56
Reconstrução T. Intestinal	1	2,56
Paratireoidectomia Parcial		

As fases pós cirúrgicas deixaram de ser realizadas quando o sujeito da amostra, mesmo quando convidado a comparecer para a avaliação justificou o motivo para o não comparecimento, deixando desta forma algumas fases incompletas mas dando continuidade a avaliação e permanecendo no protocolo de pesquisa.

A fase 2, realizada por meio de ligação telefônica (TICS) nos 3° e 7° dias após a cirurgia, foi realizada por 97,4% dos pacientes. A fase 3 que ocorre 21 dias após o evento cirúrgico foi realizada por 28,21% dos pacientes.

Todos os pacientes realizaram os testes neuropsicológicos pré-operatórios. Em relação aos testes pós-operatórios, os resultados do número de pacientes em cada momento da avaliação encontram-se na tabela 2.

Tabela 1 – Número de pacientes em cada momento da avaliação

Momento da Avaliação	Número de Indivíduos	%
3° e 7° dias	38	97,44
21 dias	11	28,21
90 dias	11	28,21
180 dias	8	20,51

DISCUSSÃO PRELIMINAR

6 DISCUSSÃO PRELIMINAR

McDonagh DL et al (2010) mostram a alta incidência de POCD em idosos submetidos a cirurgias não cardíacas graves, 54,3% em 6 semanas e 46,1% em 1 ano. Outro estudo mostra uma incidência de 25,8% e 9,9% de prevalência de POCD em uma semana e três meses respectivamente, Steinmetz J, 2009. A prevalência de 41,4 % e 12,7% de POCD em pacientes que receberam alta hospitalar e após três meses, respectivamente foi encontrada na pesquisa de Monk et al, 2008. A maior incidência de POCD no estudo de McDonagh DL e colegas (2010) podem ser por causa das cirurgias eletivas ortopédicas (74% dos pacientes), ou podem ser atribuída a diferenças na metodologia, incluindo a definição de POCD e o uso de um grupo controle. Estudos sugerem que uma boa reserva cognitiva diminui a possibilidade de comprometimento neuropsicológico após eventos cirúrgicos, como nível de escolaridade, estrutura genética, sendo um fator significativo no pós-operatório.

Pesquisas longitudinais evidenciam que fatores genéticos respondem por mais de 50% da variação na capacidade cognitiva de adultos, McClean et al usaram uma grande quantidade de testes em gêmeos idosos e foi encontrada uma herdabilidade de 0,62% para habilidade cognitiva geral, 0,55% para a habilidade verbal, de 0,32% para capacidade espacial, 0,62% para a velocidade de processamento e de 0,52% para a memória, assim como as medidas de controle executivo 0,34%. Estes estudos convergem para a conclusão de que fatores genéticos aumentam a probabilidade da POCD em cirurgias com anestesia geral, enquanto diminuem esta probabilidade para os fatores ambientais. Aproximadamente, 50% da variância fenotípica na capacidade cognitiva humana é estável. No entanto, a influência da genética na POCD não é tão clara, especialmente em pacientes cirúrgicos não cardíacos. Abildstrom et al, estudaram a relação entre APOE4 e disfunção cognitiva após cirurgia não cardíaca em 972 pacientes e não encontraram nenhuma diferença na incidência de POCD (10,3% vs 9,9%) entre os pacientes com e sem alelo APOE4.

Rasmussen et al (2009), identificaram uma possível predisposição genética para POCD após cirurgias não cardíacas. Embora não tenham encontrado nenhuma associação estatisticamente significativa entre genótipo APOE e POCD em uma semana ou 3 meses após as cirurgias, a análise de regressão logística deste estudo identificou a idade, duração de anestesia e prevenção de álcool como fatores de risco após uma semana aliados à predisposição genética. Três meses depois, esses fatores de risco não foram mais identificados. Em relação a idade, a incidência de POCD uma semana após a cirurgia foi de 10,4%, que é menor do que no estudo ISPOCD, onde uma incidência de 25,8% foi detectada em pacientes idosos submetidos a cirurgia com anestesia geral. Outros mecanismos podem ser responsáveis, portanto o genótipo APOE pode ser conectado com POCD após cirurgia cardíaca, mas não depois de cirurgias não cardíacas.

Outro estudo prospectivo longitudinal para investigar simultaneamente o cognitivo no pós-operatório e mudanças após cirurgias cardíacas e não cardíacas em pacientes adultos de todas as idades apresentam resultados de 30-41% para POCD na alta hospitalar num total de todos os sujeitos do estudo. Os pacientes de todas as idades têm melhoras no funcionamento cognitivo por três meses após a cirurgia, mas a prevalência de POCD foi maior nos idosos do que nos jovens ou adultos de meia idade. O avanço da idade e baixa escolaridade são fatores de risco para o desenvolvimento de declínio cognitivo após cirurgia não cardíaca. A avaliação dos biomarcadores plasmáticos de lesão cerebral, NSE e S-100, também produziram resultados conflitantes em vários estudos com amostras de vários tamanhos. NSE é geralmente considerado um marcador de lesão neuronal, enquanto S100B é um marcador de astrocíticos (Glia). Rasmussen et al, estudaram 65 pacientes idosos submetidos a cirurgia abdominal e não encontraram associação entre os níveis NSE e S100B e declínio cognitivo. Da mesma forma, Linstedt et al estudaram S100 e NSE em 120 pacientes mas o alto nível de NSE não foi relacionado com a POCD. Embora estes biomarcadores tenham aumentado ao longo do tempo e apresentem uma resposta inflamatória, o S100B e NSE sugerem ausência de lesão neuronal.

Os estudos sobre POCD são condicionados pelas limitações metodológicas relacionadas com o tipo de investigação em causa e por fatores relacionados com a natureza do objeto de estudo. Das dificuldades inerentes encontradas no estudo da cognição destacam-se a seleção das medidas de avaliação neuropsicológica e o ajuste estatístico a potenciais variáveis de confusão e delirium, como por exemplo, os efeitos das comorbidades com doenças ateroscleróticas e a presença de estresse psicossocial. O estudo das funções cognitivas pode ser realizado por vários métodos de natureza distinta. As duas categorias principais são os testes neuropsicológicos ou os métodos fisiológicos, estes últimos incluindo testes neurofisiológicos e técnicas funcionais de imagem. Quanto as técnicas neuropsicológicas, nosso foco neste estudo, utilizam-se medidas para aferir as alterações cognitivas, as chamadas baterias de testes neuropicológicos, que consistem em questionários de avaliação psicométrica ou testes de execução prática. Na sua seleção é importante considerar a sensibilidade dos mesmos para as alterações associadas ao pós-operatório de cirurgia cardíaca, a abrangência dos vários domínios cognitivos envolvidos, a seleção de medidas resistentes aos efeitos da aprendizagem ou com formas alternativas que evitem a melhoria do desempenho pela administração seriada dos testes durante o seguimento clínico após a cirurgia.

A enorme variabilidade na composição das baterias entre os diferentes estudos e o uso de diferentes critérios estatísticos para a definição de disfunção cognitiva a comparação de resultados entre si dificulta a homogeneidade nos resultados apresentados pelos estudos.

Uma das limitações da maioria dos estudos que investigam a POCD é que existem grandes diferenças nas metodologias de pesquisa, incluindo as baterias de testes neuropsicológicos, o intervalo entre as sessões de teste, a definição de POCD e os métodos estatísticos utilizados para analisar os escores. Nesta condição este estudo também encontra limitações para o desenvolvimento do projeto, com as mesmas dificuldades já expostas em outras pesquisas, como o compromisso dos sujeitos em comparecer à todas as fases da avaliação, a escolha dos testes neuropsicológicos e a definição de disfunção cognitiva.

ATIVIDADES DESENVOLVIDAS E PERSPERCTIVAS PARA O PRÓXIMO PERÍODO

7 ATIVIDADES DESENVOLVIDAS E PERSPECTIVAS

7.1 ANDAMENTO DO ESTUDO

O andamento do estudo encontra-se atrasado principalmente em decorrência da indisponibilidade de profissionais da área da psicologia para a aplicação da avaliação da bateria neuropsicológica em meses anteriores. No início do estudo apenas uma neuropsicóloga aplicava os testes, e a disponibilidade de tempo da mesma mostrou-se incompatível com a necessidade do estudo. Foram incorporados mais três profissionais ao grupo, permitindo o aumento significativo do recrutamento. A partir do início de 2011 o recrutamento de pacientes cresceu e segue progredindo, no entanto, a previsão de finalização da inclusão de pacientes será março de 2012, baseado no número de pacientes inclusos até fevereiro de 2011.

Portanto, considerando que o tempo de seguimento dos pacientes após o recrutamento é de seis meses, vê-se a impossibilidade de finalização do estudo dentro do prazo inicialmente previsto (fevereiro de 2012). Sendo assim, solicita-se à FAPESP tempo adicional de 12 meses (até fevereiro de 2013) para finalização da pesquisa, incluindo a análise dos dados.

Cronograma para finalização do estudo:

**Etapas**	**Prazos**
Finalização da inclusão de pacientes	Março de 2012
Finalização da avaliação neuropsicológica dos pacientes	Setembro de 2012
Análise estatística	Dezembro de 2012
Avaliação dos resultados e elaboração do artigo	Fevereiro de 2013

7.2 PARTICIPAÇÃO DE ALUNOS NO PROJETO:

As residentes de Anestesiologia da Faculdade de Medicina da Universidade de São Paulo, Dra. Ruth Camarão e Dra. Mariane Galzerano Stahlberg participaram da fase piloto do estudo (antes do início da randomização), que visava a avaliação dos testes neuropsicológicos traduzidos para o português. Os resultados foram encaminhados como tema livre para o Congresso Brasileiro de Anestesiologia e serão encaminhados para publicação na Revista Brasileira de Anestesiologia.

A neuropsicóloga Lívia S.S. Valentin participa ativamente do estudo e está matriculada no programa de pós-graduação em anestesiologia da FMUSP, sendo que os dados deste projeto resultarão em sua tese de doutorado.

7.3 DISCUSSÃO DO PROJETO COM OUTROS GRUPOS DE PESQUISA

Durante viagem como "visiting professor" à Duke University, a evolução do estudo e os problemas apresentados, especialmente a dificuldade de recrutamento e de aderência foram discutidos com os pesquisadores Prof. Dr. Mark Newman e Profa. Dra. Terry Monk, anestesiologistas com diversas publicações em POCD. Os pesquisadores relataram as mesmas dificuldades durante a realização de seus estudos e citaram as estratégias utilizadas para solução dos mesmos, as quais já estão sendo atualmente aplicadas. Uma das medidas resultantes da discussão foi o aumento do número de neuropsicólogas participantes do estudo.

Adicionalmente, discutiu-se a necessidade de validação e adaptação transcultural dos testes neuropsicológicos atualmente em uso no projeto, o que está sendo discutido com o Prof. Dr. Lars S. Rasmussen, da Universidade de Kopenhagen, Dinamarca.

7.4 UTILIZAÇÃO DOS RECURSOS DE RESERVA TÉCNICA E BENEFÍCIOS COMPLEMENTARES

1. **A prestação de contas dos recursos utilizados encontra-se em anexo (encaminhados diretamente à FAPESP)**

2. **Utilização dos recursos da Reserva técnica:** foram utilizados para aquisição de estabilizador de rede voltagem e back up específicos para o freezer -70°C obtidos com este auxílio FAPESP, pois quando da solicitação do auxílio, desconhecíamos a necessidade de aquisição destes itens para prevenção de danos ao equipamento por flutuações na rede elétrica e manutenção de temperatura nos momentos de queda de fornecimento de eletricidade, motivo da utilização da reseva técnica com esta finalidade.
   A reserva técnica foi utilizada também para aquisição de material de consumo (Material de escritório) para administração do projeto de pesquisa.

3. **Utilização de recursos de benefícios complementares:** Os recursos foram utilizados para patrocinar, parcialmente, viagem como "visiting professor" à Duke University. Além do auxílio à pesquisa FAPESP, parte dos recursos deste projeto (aquisição dos testes neuropsicológicos) são provenientes da Duke University.

4. **Previsão de utilização futura dos recursos:** Considerando a solicitação de prazo adicional para finalizar a inclusão de pacientes, solicita-se também postergação da prestação final de contas para fevereiro/2013 devido à necessidade de aquisição de kits para exames laboratoriais apenas após o final da coleta dos dados.

5. **Solicitação de recursos adicionais:** Durante a solicitação do auxílio, os recursos para serviços de terceiros relacionados à aplicação dos testes neuropsicológicos foram subdimensionados (o orçamento inicial foi feito para 150 pacientes, enquanto que o número total de pacientes do projeto é 300), resultando que prevemos dificuldades de pagamento do serviço de avaliação

neuropsicológica do projeto e, portanto, solicita-se à FAPESP verba adicional para o para pagamento de serviços de terceiros.

REFERÊNCIAS BIBLIOGRÁFICAS

8 REFERÊNCIAS BIBLIOGRÁFICAS

1. Abildstrom H, Christiansen M, Siersma VD, Rasmussen LS: Apolipoprotein E genotype and cognitive dysfunction after noncardiac surgery. Anesthesiology, 2004; 101:855-61

2. Andrew MJ, Baker RA, Kneebone AC, Knight JL. Mood state as a predictor of neuropsychological deficits following cardiac surgery. Journal of Psychosomatic Research 48 (2000) 537 - 546

3. Aquino RB, Souza ACA, Argimon IL, Santos PFR. Effects of General Anesthesia in Elderly Patients Memory and Cognition. Rev Bras Anestesiol, 2004; 54: 5: 687- 692

4. Asioglu K, Celik SS. The effect of preoperative education on anxiety of open cardiac surgery patients. Patient Education and Counseling 53 (2004) 65-70

5. Astudillo, Linden JV, Radegran K, Hansson LO, Aberg B. Elevated serum levels of S-100 after deep hypothermic arrest correlate with duration of circulatory arrest. Eur J Cardio-thorac Surg (1996) 10:1107-1113

6. Arrowsmith JE, Grocott HP, Reves JG, Newman MF. Central nervous system complications of cardiac surgery. British Journal of Anaesthesia 84 (3): 378-93 (2000)

7. Bass DS, Attix DK, Phillips-Bute B, Monk TG. An Efficient Screening Tool for Preoperative Depression: The Geriatric Depression Scale-Short Form. Ambulatory Anesthesiology vol. 106, nº 3, March 2008

8. Barbosa FT, Cunha RM, Pinto ALCLT. Delirium Pós-Operatório em Idosos. Ver Bras Anestesiol; 2008:58: 6:665-670

9. Ballard CG, Morris M, Rao H, O'Brien JT, Barber R, Stephens S. APOE epsilon4 and cognitive decline in older stroke patients with early cognitive impairment. Neurology 2004; 63: 1399-1402

10. Bekker AY, Weeks EJ- Cognitive function after anaesthesia in elderly. Best Pract Res Clin Anaesthesiol, 2003; 17:259-272

11. Benoit AG, Campbell BI, Tanner JR, Staley JD, Wallbridge HR, Bihel DR, Bradley BD, Louridas G, Guzman RP and Fromm RA. Risk factors and prevalence of perioperative cognitive dysfunction in abdominal aneurysm patients. J Vasc Surg 2005;42: 884-90

12. Boss GL, Soares LF, Oliveira Filho GR. Postoperative Cognitive Dysfunction: Prevalence and Associated Factors Ver Bras Anestesiol, 2005; 55:5 517-524

13. Brown JP, Sollers III JJ,Thayer JF, Zonderman AB, Waldstein. Blood Pressure Reactivity and Cognitive Function in the Baltimore Longitudinal Study of Aging. Health Psychol. 2009 September; 28(5): 641-646.

14. Bryson GL, Wyand A- Evidence-based clinical update: general anestesia and the risk of delirium and postoperative cognitive dysfunction. Can J Anesth, 2006; 53: 669-677.

15. Canet J, Raeder J, Rasmussen LS- Cognitive dysfunction after minor surgery in the elderly. Acta Anaesthesiol Scand, 2003; 47: 1204-1210

16. Cottrell JE. We Care, Therefore We Are: Anesthesia-related Morbidity and Mortality. Anesthesiology, V 109. Nº 3, Sep 2008

17. Deiner S & Silverstein JH. Postoperative delirium and cognitive dysfunction. British Journal of Anesthesia 103(BJA/PGA Supplement): i41- i46 (2009)

18. Di Carlo A, Penna AM, Pantoni L, Basile AM, Bonacchi M, Pracucci G. Clinically relevant cognitive impairment after cardiac surgery: a 6-month follow-up study. J Neurol Sci. 2001;188(1-2):85-93

19. Dijkstra JB, Jolles J: Postoperative cognitive dysfunction versus complaints: a discrepancy in long-term findings. Neuropsychol Rev 2002; 12: 1-14

20. Dijkstra JB, Jolles J. Cognition after major surgery in the elderly: test performance and complaints. British Journal of Anaesthesia 82 (6): 867-74 (1999)

21. Djaiani GN, Phillips-Bute B, Blumenthal JA, Newman MF, Chronic Exposure to nicotine does not prevent neurocognitive decline after cardiac surgery. Journal of Cardiothoracic and Vascular Anesthesia, 2003;17(3):341-345

22. Edlund A, Lundstrom M, Brannstrom B, Bucht G, Gustafson Y. Delirium Before and After Operation for Femoral Neck Fracture. J. Am Geriatr Soc, 2001; 49:1335-1340

23. Fong HK, Sands LP, and Leung JM. The Role of Postoperative Analgesia in Deliriun and Cognitive Decline in Elderly Patients: A Systematic Review. Anesth Analg 2006; 102:1255-66

24. Funder KS, Steinmetz J, Rasmussen LS. Cognitive dysfunction after cardiovascular surgery. Minerva Anestesiol, 2009; 75:329-32

25. Gao L, Taha R, Gauvin D, Othmen LB, Wang Y, Blaise G. Postoperative cognitive dysfunction after cardiac surgery. Chest. 2005; 128 (5):3664-70

26. Greene NH, Attix DK, Weldon BC, Smith PJ, McDonagh DL, Monk TG. Measures of executive function and depression identify patients at risk for postoperative delirium. Anesthesiology, 2009;110(4)

27. Grigore AM, Grocott HP, Mathew JP, Phillips-Bute B, Stanley TO, Butler A, Landolfo KP, Reves JG, Blumenthal JA, Newman MF. The Rewarming Rate and Increased Peak Temperature Alter Neurocognitive Outcome After Cardiac Surgery. Anesth Analg 2002;94:4-10

28. Grigore AM, Mathew JP, Grocott HP, Reves JG, Blumenthal JA, White WD, Smith PK, Jones RH, Kirchner JL, Mark DB, Newman MF. Prospective randomized trial of nomothermic versus hypothermic cardiopulmonary bypass on cognitive function after coronary artery bypass graft surgery. Anesthesiology, 2001;95(5)

29. Grocott HP, Homi HM, Puskas F. Cognitive dysfunction after cardiac surgery: revisiting etiology. Semin Cardiothorac Vasc Anesth. 2005; 9(2):123-9

30. Grocott HP, Yoshitani K. Neuroprotection during cardiac surgery. J Anesth, 2007;21(3):367-77

31. Hanning CD. Postoperative cognitive dysfunction. Br J Anaesth 2005; 95:82-7

32. Heyer EJ, Gold MI, Kirby EW, Zurica J, Mitchell E, Halazun HJ, Teverbaugh L, Sciacca RR, Solomon RA, Quest DO, Maldonado TS, Riles TS and Connolly Jr ES. A study of Cognitive Dysfunction in Patients Having Carotid Endarterectomy Performed with Regional Anesthesia.Anesth Analg.2008 August; 107 (2): 636-642.

33. Hogue CW Jr, Palin CA, Arrowsmith JE. Cardiopulmonary bypass management and neurologic outcomes: an evidence-based appraisal of current practices. Anesth Analg. 2006;103(1):21-37

34. Hudetz JA, Ptterson KM, Byrne AJ, Iqbal Z, Gandhi SD, Warltier DC, Pagel PS. A history of Alcohol Dependence Increases the Incidence and Severity of Postoperative Cognitive Dysfunction in Cardiac Surgical Patients. Int. J. Environ. Res. Public Health 2009, 6, 2725-2739

35. Hudetz JA, Iqbal Z, Gandhi SD, Patterson KM, Byrne AJ, Hudetz AG, Pagel PS, Warltier DC. Ketamine attenuates post-operative cognitive dysfunction after cardiac surgery. Acta Anaesthesiol Scand. 2009 Aug; 53(7):864-72

36. Ille R, Lahousen T, Schweiger S, Hofmann P, Kapfhammer HP. Influence of patient-related and surgery-related risk factors on cognitive performance, emocional state, and convalescence after cardiac surgery. Cardiovasc Revasc Med. 2007;8(3):166-9

37. Iohom G, Szarvas S, Laney V, O'Brien J, Buckley E, Butler M and Shorten G. Anesth Analg 2004; 99:1245-52

38. Iselin-Chaves IA, Willems SJ, Jermann FC, Foster A, Adam SR, Linden MV. Investigation of implicit memory during isoflurane anesthesia for elective surgery using the process dissociation procedure. Anesthesiology, 2005;103(5)

39. Ishida K, Gohara T, Kawata R, Ohtake K, Morimota Y, Sakabe T. Are serum S100B proteins and neuron-specific enolase predictors of cerebral damage in cardiovascular surgery? J Cardiothorac Vasc Anesth 2003; 17:4-9

40. Inzitari M, Pozzi C, Ferrucci L, Chiarantini D, Rinaldi LA, Baccii M, Pini R, Masotti G, Marchionni N, Di Bari M: Subtle neurological abnormalities as risk

factors for cognitive and functional decline, cerebrovascular events, and mortality in older community-dwelling adults. Arch Intern Med 2008, 168: 1270-6

41. Jensen BO, Hughes P, Rasmussen LS, Pedersen PU, Steinbrüchel DA. Cognitive Outcomes in Elderly High-Risk Patients After Off-Pump Versus Conventional Coronary Artery Bypass Grafting- A Randomized Trial. Circulation, 2006; 113: 2790-2795

42. Johnson T, Monk TG, Rasmussen LS- ISPOCD 2 Investigators. Postoperative cognitive dysfunction in middle-aged patients. Anesthesiology 2002;96:1351-7

43. Kadoi Y, Goto F. Factors associated with postoperative cognitive dysfunction in patients undergoing cardiac surgery. Surg Today. 2006; 36 (12): 1053-7

44. Kain ZN, Caldwell-Andrews AA, Maranets I, McClain B, Gaal D, Mayes LC, Feng R, Zhang H. Preoperative Anxiety and Emergence Delirium and Postoperative Maladaptive Behaviors. Anesth. Analg 2004; 99:1648-54

45. Kleindienst A, Bullock MR: A critical analysis of the roles of the neurotrophic protein S100B in acute brain injury. J Neurotrauma 2006; 23: 1185-200

46. Kudoh A, Takase H, Takahira Y and Takazawa T. Postoperative Confusion Increases in Elderly Long-Term Benzodiazepine Users. Anesth Analg 2004;99:1674-8

47. Kuzumi E, Vuylsteke A, Guo X, Menon DK, Serum S100 protein as a marker of cerebral damage during cardiac surgery. Br J Anaesth. 2000;85(6):936-42

48. Koster S, Oosterveld FGJ, Hensens AbG, Wijma A, Palen J. Delirium After Cardiac Surgery and Predictive Validity of a Risk Checklist.Ann Thorac Surg, 2008; 86: 1883-7

49. Lelis RGB, Auler Jr JOC. Lesão neurológica em cirurgia cardíaca: aspectos fisiopatológicos. Ver Bras Anestesiol, 2004; 54(4): 607- 17

50. Linnea EV, Sands LP, Wang Y, Mullen EA Leung JM: Postoperative delirium: the importance of pain and pain management. Anesth Analg 2006; 102:1267-73

51. Linstedt U, Meyer O, Kropp P, Berkau A, Tapp E, Zenz M. Serum concentration of S 100B protein in assessment of cognitive dysfunction after general anaesthesia in different types of surgery. Acta Anaesthesiol Scand 2002; 46: 384-9

52. Mandal PK, Schifilliti D, Mafrica F and Fodale V. Inhaled Anesthesia and Cognitive Performance.Drugs of Today, 2009, 45 (1): 47-54

53. Mahanna EP, Blumenthal JA, White WD, Croughwell ND, Clancy CP, Smith LR, Newman MF. Defining Neuropsychological Dysfunction After Coronary Artery Bypass Grafting. The Society of Thoracic Surgeons Published by Science

54. Martin JFV, Melo ROV, Sousa LP. Postoperative cognitive dysfunction after cardiac surgery. Rev Bras Cir Cardiovasc 2008; 23 (2): 245-255

55. Martin JFV, Melo ROV, Sousa LP. Disfunção cognitiva após cirurgia cardíaca. Rev Bras Cir Cardiovasc 2008; 23(2): 245-255

56. Mathew JP, Mackensen B, Philips-Bute B, Grocott HP, Glower DD, Laskowitz DT, Blumenthal JA, Newman MF. Randomized, double-blinded, placebo controlled study of neuroprotection with lidocaine in cardiac surgery. Stroke. 2009; 40:880-887

57. Mathew JP, Shernan SK, White WD, Fitch JCK, Chen JC, Bell L, Newman MF. Preliminary report of the effects of complement suppression with pexelizumab on neurocognitive decline after coronary artery bypass graft surgery. Stroke. 2004; 35:2335-2339

58. Mathew JP, Grocott HP, Phillips-Bute B, Stafford-Smith M, Laskowitz DT, Rossignol D, Blumenthal JA, Newman MF. Lower Endotoxin Immunity Predicts Increased Cognitive Dysfunction in Elderly Patients After Cardiac Surgery. Stroke 2003;34(2):508-13

59. Mathew JP, Grocott HP, McCurdy II JR, Ti LK, Davis RD, Laskowitz DT, Podgoreanu MV, Swaminathan M, Lynch J, Stanfford-Smith M, White WD, Newman MF. Preoperative Statin Therapy Does Not Reduce Cognitive Dysfunction After Cardiopulmonary Bypass. Journal of Cardiothoracic and Vascular Anesthesia, Vol 19, nº 3, 2005: pp 294-299

60. Mathew JP, Podgoreanu MV, Grocott HP, White WD, Morris RW, Stanfford-Smith M, Mackensen GB, Rinder CS, Blumenthal JA, Schwinn DA, Newman MF. Genetic Variants in P-Selectin and C-Reactive Protein Influence Susceptibility to Cognitive Decline After Cardiac Surgery. Journal of the American College of Cardiology, vol. 49, nº 19, 2007

61. Mathew JP, Mackensen GB, Phillips-Bute B, Stafford-Smith M, Podgoreanu MV, Grocott HP, Hill SE, Smith PK, Blumenthal JA, Reves JG, Newman MF; Neurologic Outcome Research Group (NORG) of the Duke Heart Center. Effects of Extreme Hemodilution during Cardiac Surgery on Cognitive Function in the Elderly. Anesthesiology, 2007;107(4):577-84

62. McDonagh DL, Mathew JP, White WD, Philips-Bute B, Laskowitz DT, Podgoreanu MV, Newman MF; for the Neurologic outcome research grouptt. cognitive function after major noncardiac surgery, apolipoprotein E4 genotype, and biomarkers of brain injury. Anesthesiology 2010; 112:852-9

63. Monk TG, Saini V, Weldon BC, Sigl JC. Anesthetic Management and One-Year Mortality After Noncardiac Surgery. Anesth Analg 2005;100:4-10

64. Monk TG, Weldon BC, Garvan CW, Dede DE, van der Aa MT, Heilman KM, Gravestein JS: Predictors of cognitive dysfunction after major noncardiac surgery. Anesthesiology 2008; 108: 18-30

65. Newfield P. Postoperative cognitive dysfunction. Medicine Reports LTD, 2009, 1: 14

66. Newman MF, Grocott HP, Mathew JP, White WD, Landolfo K, Reves JG, Laskowitz DT, Mark DB, Blumenthal JA. Report of the Substudy assessing the Impact Of Neurocognitive Function on Quality of Life 5 Years After Cardiac Surgery. Stroke is available at http://www.strokeaha.org

67. Newman MF, Kramer D, Croughwell ND, Sanderson I, Blumenthal JA, White WD, Smith LR, Towner EA, Reves JG. Differential Age Effects of Mean Arterial Pressure and Rewarming on Cognitive Dysfunction After Cardiac Surgery. Anesth Analg 1995;81:236-42

68. Newman NF, Kirchner JL, Phillips-Bute B, Gaver V, Grocott H, Jones RH, Mark DB, Reves JG, The Cardiothoracic Anesthesiology Research Endeavors

Investigators. Longitudinal Assessment of Neurocognitive Function After Coronary-Artery Bypass Surgery. The New England Journal of Medicine 2001; vol.344 No. 6

69. Newman S, Stygall J, Hirani S, Shaefi S, Maze M. Postoperative Cognitive Dysfunction After Noncardiac Surgery. Anesthesiology, V 106, N° 3 Mar 2007

70. Niemi-Murola L, Pöyhiä R, Onkinen K, Rhen B, Mäkelä A and Niemi TT. Patient Satisfaction with Postoperative Pain Management-Effect of Preoperative Factors. Pain Management Nursing, vol 8, n° 3 (September), 2007:pp122-129

71. Nötzold A, Michel K, Khattab AA, Sievers HH, Hüppe M. Dabetes mellitus increases adverse neurocognitive outcome after coronary artery bypass grafting surgery. Thorac Cardiovasc Surg 2006;54(5):307-12

72. Norkiene I, Samalavicius R, Misiuriene I, Paulauskiene K, Budrys V, Ivas kevicius J. Incidence and risk factors for early postoperative cognitive decline after coronary artery by-pass grafting. Medicina (Kaunas) 2010; 46(7): 460-4

73. Phillips-Bute B, Mathew JP, Blumenthal JA, Grocott HP, Laskowitz DT, Jones RH, Mark DB, Newman MF. Association of Neurocognitive Function and Quality of Life 1 Year After Coronary Artery Bypass Graft (CABG) Surgery. Psychosomatic Medicine 68:369-375 (2006)

74. Praticò C, Quattrone D, Lucanto T, Amato A, Penna O, Roscitano C, Fodale V. Drugs of anaesthesia acting on central cholinergig system may cause post-operative cognitive dysfunction and delirium. Med Hypotheses 2005, 65 (5): 972-82

75. Puskas F, Grocott HP, White WD, Mathew JP, Newman MF, Bar-Yosef S. Intraoperative Hyperglycemia and Cognitive Decline After CABG. The Society of Thoracic Surgeons Published by Elsevier

76. Price CC, Garvan CW, Monk TG. Type and Severity of Cognitive Decline in Older Adults after Noncardiac Surgery. Anesthesiology, V 108, n° 1, Jan 2008

77. Rasmussen LS, Christiansen M, Rasmussen H, Kristensen PA, Moller JT. Do blood concentrations of neuron specific enolase and S100B protein reflect cognitive dysfunction after abdominal surgery? ISPOCD group. Br J Anaesth 2000; 84:242-4

78. Rasmussen LS, Johnson T, Kuipers HM, Kristensen D, Sierm VD, Vila P, Jolles J, Papaioannou A, Abildstrom H, Silverstein JH, Bonal JA, Raeder J, Nielsen IK, Korttila K, Munoz L, Dodds C, Hanning CD, Moller JT; Internacional Study of Postoperative Cognitive Dysfunction 2 Investigators: Does anaesthesia cause postoperative cognitive dysfunction? A randomised study of regional versus general anaesthesia in 438 eldery patients. Acta Anaesthesiol Scand 2003; 47: 260-6

79. Rasmussen LS, Christiansen M, Eliasen K, Sander-Jensen K, Moller JT. Biochemical markers for brain damage after cardiac surgery- time profile and correlation with cognitive dysfunction. Acta Anaesthesiol Scand 2002; 46:547-551

80. Rasmussen LS. Postoperative cognitive dysfunction: Incidence and prevention. Best Pract Res Clin Anaesthesiol 2006, 20(2):315-30

81. Rasmussen LS, O´Brien JT, Silverstein JH, Johnson TW, Siersma VD, Canet J, Jolles J, Hanning CD, Kuipers HM, Abildstrom H, Papaioannou A, Raeder J, Yli-Hankala A, Sneyd JR, Munoz L, Moller JT.Is peri-operative cortisol secretion related to post-operative cognitive dysfunction?. Acta Anaesthesiol Scand 2005; 49: 1225-1231

82. Rymaszewska J, Kiejna A, Hardrys T. Depression and anxiety in coronary artery bypass grafting patients. European Psychiatry 18 (2003) 155-160

83. Rasmussen LS, Larsen K, Houx P, Skovgaard LT, Hanning CD, Moller JT and the ISPOCD Group. The assessment of postoperative cognitive function. Acta Anaesthesiol Scand 2001;45:275-289

84. Reynolds JD, Amory DW, Grocott HP, White WD, Newman MF. Change in Plasma Glutamate Concentration During Cardiac Surgery Is a Poor Predictor of Cognitive Outcome. Journal of Cardiothoracic ond Vascular Anesthesia, vol 16, nº4 (August), 2002: pp 431-436

85. Rohan D, Buggy DJ, Crowley S, Ling FK, Gallagher H, Regan C, Moriarty DC. Increased incidence of postoperative cognitive dysfunction 24 hr after minor surgery in the elderly. Can J Anaesth 2005, 52 (2): 137-42

86. Rudolph JL, Schreiber KA, Culley DJ, McGlinchey RE, Crosby G, Levitsky S, Marcantonio ER. Measurement of post-operative cognitive dysfunction after cardiac surgery: a systematic review. Acta Anaesthesiol Scand. 2010 Apr 15.

87. Stanley TO, Mackensen GB, Grocott HP, White WD, Blumenthal JA, Laskowitz DT, Landolfo KP, Reves JG, Mathew JP, Newman MF. The Impact of Postoperative Atrial Fiblillation on Neurocognitive Outcome After Coronary Artery Bypass Graft Surgery. Anesth Analg 2002;94:290-5

88. SELNES OA, ZEGER SL: Coronary artery bypass grafting baseline cognitive assessment: essencial not optional. Ann Thorac. Coronary Surg 2007;83:374-6

89. Sear, JW. Implication of aging on anaesthetic drugs. Curr Opin Anaesthesiol 2003, 16(4):37-8

90. Swaminathan M, McCreath BJ, Phillips-Bute BG, Newman MF, Mathew JP, Smith PK, Blumenthal JA, Stafford-Smith M. Serum Creatinine Patterns in Coronary Bypass Surgery Patients With and Without Postoperative Cognitive Dysfunction.Anesth Analg 2002;95:1-8

91. Selwood A, Orrell M. Long term cognitive dysfunction in older people after non-cardiac surgery. Br Med J 2004; 328:120-1

92. Silbert BS, Evered LA, Scott DA, Cowie TF: The apolipoprotein E4 allele is not associated with cognitive dysfunction in cardiac surgery. Ann Thorac Surg 2008; 86:841-8

93. Stern Y: What is cognitive reserve? Theory and research application of the reserve concept. J Int Neuropsychol Soc 2002: 8:448-60

94. Steinmetz J, Christensen KB, Lund T, Lohse N, Rasmussen LS; for the International Study of Postoperative Cognitive Dysfunction Group: Long-term consequences of postoperative cognitive dysfunction. Anesthesiology 2009; 110:548-55

95. Smith PJ, Attix DK, Weldon BC, Greene NH, Monk TG. Executive Function and Depression as Independent Risk Factors for Postoperative Delirium. Anesthesiology, V 110, nº4, Apr 2009

96. Tagarakis GI, Tsolaki-Tagaraki F, Tsolaki M, Diegeler A, Tsilimingas NB, Papassotiropoulos A. The role of apolipoprotein E in cognitive decline and delirium after bypass heart operations. Am J Alzheimers Dis Other Demen. 2007; 22(3): 223-8

97. Tardiff BE, Newman MF, Saunders AM, Strittmatter WJ, Blumenthal JA, White WD, Croughwell ND, Davis RDJ, Roses AD, Reves JG. Preliminary Report of a Genetic Basis for Cognitive Decline After Cardiac Operations. Ann Thorac Surg 1997;64:715-20

98. Ti LK, Mathew JP, Mackensen GB, Grocott HP, White WD, Reves JG, Newman MF. Effect Apolipoprotein E Genotype on Cerebral Autoregulation During Cardiopulmonary Bypass. Stroke is available at http://www.strokeaha.org

99. Vaurio LE, Sands LP, Wang Y, Mullen EA, Leung JM. Postoperative Delirium: The Importance of Pain and Pain Management. Anesth Analg 2006;102:1267-73

100. Willians-Russo P, Sharrock, Nigel EM, Mattis, Steven, Liguori, Gregory A, Mancuso, Carol MD, Peterson MG, Hollenberg JM, Ranawat, Chitranjan MD, Salvati, Eduardo MD, Sculco, Thomas MD. Randomized Trial of Hypotensive Epidural Anesthesia in Older Adults. Anesthesiology. 1999;91(4):926

101. Wong C, Bonser RS, Missler U, Weismann M. Serum S-100 Protein in Stroke and Cardiac Surgery Response. Stroke 1998;29;2446-2447

102. Wu CL, Hsu W, Richman JM and Raja SN. Postoperative Cognitive Function as an Outcome of Regional Anesthesia and Analgesia. Regional Anesthesia And Pain Medicine, 2004;29(3):257-268

103. Yi-qing Y, Ai-lun L, Xiang-yang G, Li-huan L and Yu-guang H. Postoperative neuropsychological change and its underlying mechanism in

patients undergoing coronary artery bypass grafting. Chinese Medical Journal 2007; 120 (22): 1951-1957

104. Zhou W, Xu D, Peng X, Zhang Q, Jia J, Crutcher KA; Meta-analysis of APOE4 allele and outcome after traumatic brain injury. J Neurotrauma 2008; 25: 279-90

APÊNDICES

APÊNDICES

Os quadros seguintes apresentam os valores porcentuais de cada atributo em cada momento do estudo.

Quadro I. Dados dos pacientes incluídos no estudo

PAC HC	GENERO	CIRURGIA	EXCLUSÃO	INCLUSÃO	AVAL. PC	3º DIA	7º DIA	21º DIA	90º DIA	180º DIA	Em avaliação	Fases incompletas		GENERO 1	GENERO 2
1	2	1	0	1	1	1	1	1	1	1	0	1	1	0	1
2	2	1	0	1	1	1	1	1	1	1	0	1	1	0	1
3	2	4	0	1	1	1	0	0	0	0	0	1	1	0	1
4	2	1	0	1	1	1	1	0	1	0	0	1	1	0	1
5	2	1	0	1	1	1	1	0	0	0	0	1	1	0	1
6	2	5	0	1	1	0	0				1	0	1	0	1
7	2	1	0	1	1	1	1				1	0	1	0	1
8	2	9	0	1	1	1	1	1	1	1	0	1	1	0	1
9	2	1	0	1	1	1	1	1	1	1	0	1	1	0	1
10	2	10	0	1	1	1	1	1	1	1	0	1	1	0	1
11	2	1	0	1	1	1	1	0	0	0	0	1	1	0	1
12	2	7	0	1	1	1	1	1	1	1	0	1	1	0	1
13	2	9	0	1	1	1	1				1	0	1	0	1
14	2	1	0	1	1	1	1				1	0	1	0	1
15	2	1	0	1	1	1	1				1	0	1	0	1
16	2	1	0	1	1	1	1	0	0	0	0	1	1	0	1
17	2	1	0	1	1	1	1	0	1	1	0	1	1	0	1
18	2	1	0	1	1	1	1	1	0	0	0	1	1	0	1
19	2	1	0	1	1	1	1	0			1	0	1	0	1
20	2	2	0	1	1	1	1	1	1		1	0	1	0	1
21	1	1	0	1	1	1	1	1	1		1	0	1	1	0
22	2	1	0	1	1	1	1	1	1	1	0	1	1	0	1
23	1	1	0	1	1	1	1				1	0	1	1	0
24	1	10	0	1	1	1	1	1			1	0	1	1	0
25	2	1	0	1	1	1	1				1	0	1	0	1
26	2	5	0	1	1	1	1				1	0	1	0	1
27	1	14	0	1	1	1	1				1	0	1	1	0
28	2	10	0	1	1	1	1				1	0	1	0	1
29	2	18	0	1	1	1	1				1	0	1	0	1
30	2	1	0	1	1	1	1				1	0	1	0	1
31	1	3	0	1	1	1	1				1	0	1	1	0
32	2	1	0	1	1	1	1				1	0	1	0	1
33	1	1	0	1	1	1	1				1	0	1	1	0
34	2	1	0	1	1	1	1				1	0	1	0	1
35	2	1	0	1	1	1	1				1	0	1	0	1
36	2	1	0	1	1	1	1				1	0	1	0	1
37	2	1	0	1	1	1					1	0	1	0	1
			0	37	37	36					23	14			
			**0,00**	**50,00**	**50,00**	**48,65**					**62,16**	**37,84**	37	6	31
														**16,22**	**83,78**

Quadro II. Dados Demográficos em gênero e pacientes incluídos

TOTAL	155		
COMPLETO	4,52	%	
MASC	45	29,03	%
FEM	110	70,97	%
INCLUSÃO	23,87	%	
EXCLUSÃO	76,13	%	

Quadro III. Fases da avaliação neuropsicológica em relação aos pacientes incluídos

AINDA EM AVALIAÇÃO	15,48	%
AVAL. PC	41,94	%
3 / 7 DIA	7,10	%
21 DIA	8,39	%
90 DIA	8,39	%
180 DIA	6,45	%

Quadro IV. Porcentagem de tipos de cirurgias realizadas

CIRURGIA		
COLECISTECTOMIA	52,26	%
COLECTOMIA PARCIAL	3,87	%
COLECTOMIA VÍDEO LAPAROSCOPIA	1,29	%
ESOFAGECTOMIA DISTAL	0,65	%
FECHAMENTO DE ENTEROSTOMIA	0,65	%
HERNIOPLASTIA	1,29	%
HERNIOPLASTIA EPIGÁSTRICA	0,65	%
HERNIOPLATIA INGUINAL	11,61	%
JEJUNOSTOMIA	0,65	%
MASTOIDECTOMIA RADICAL	0,65	%
PARATIREOIDECTOMIA	2,58	%
PARATIREOIDECTOMIA PARCIAL	0,65	%
RECONSTRUÇÃO T. INTESTINAL	0,65	%
TIMPANOPLASTIA	1,94	%
TIREOIDECTOMIA PARCIAL	2,58	%
TIREOIDECTOMIA TOTAL	10,32	%
TRATAMENTO CIRURGICO MEGAESOFAGO	3,87	%
TRATAMENTO REFL. GÁSTRICO ESOFÁGICO	3,87	%

Termo de Consentimento

Anexo I

HOSPITAL DAS CLÍNICAS

DA

FACULDADE DE MEDICINA DA UNIVERSIDADE DE SÃO PAULO

TERMO DE CONSENTIMENTO LIVRE E ESCLARECIDO

(Instruções para preenchimento no verso)

I - DADOS DE IDENTIFICAÇÃO DO SUJEITO DA PESQUISA OU RESPONSÁVEL LEGAL

1. NOME DO PACIENTE: ....

DOCUMENTO DE IDENTIDADE Nº: ....SEXO: M Ž F Ž

DATA NASCIMENTO: ....../....../......

ENDEREÇO:... Nº .... APTO: ....

BAIRRO: ....CIDADE ....

CEP:................. TELEFONE: DDD (............) ....

2.RESPONSÁVEL LEGAL ....

NATUREZA (grau de parentesco, tutor, curador etc.) ....

DOCUMENTO DE IDENTIDADE :.................................. SEXO: M F

DATA NASCIMENTO: ....../......./......

ENDEREÇO: .... Nº .... APTO: ....

BAIRRO:... CIDADE: ....

CEP: .... TELEFONE: DDD (............)..

II - DADOS SOBRE A PESQUISA CIENTÍFICA

1. TÍTULO DO PROTOCOLO DE PESQUISA

DISFUNÇÃO COGNITIVA APÓS CIRURGIA SOB ANESTESIA GERAL

PESQUISADOR: Maria José Carvalho Carmona.

CARGO/FUNÇÃO: Professora Associada.... INSCRIÇÃO CONSELHO REGIONAL Nº ...54.142

UNIDADE DO HCFMUSP: .Divisão de Anestesia do Instituto Central

3. AVALIAÇÃO DO RISCO DA PESQUISA:

SEM RISCO ☐ RISCO MÍNIMO X RISCO MÉDIO ☐

RISCO BAIXO ☐ RISCO MAIOR ☐

(probabilidade de que o indivíduo sofra algum dano como consequência imediata ou tardia do estudo)

4.DURAÇÃO DA PESQUISA : .2 anos.

III - REGISTRO DAS EXPLICAÇÕES DO PESQUISADOR AO PACIENTE OU SEU REPRESENTANTE LEGAL SOBRE A PESQUISA:**O senhor (a) está sendo convidado(a) a participar de uma pesquisa, coordenada por um profissional da saúde agora denominado pesquisador. Para poder participar é necessário que o senhor(a) leia este documento com atenção. Ele pode conter palavras que o senhor(a) não entenda. Por favor peça aos responsáveis pelo estudo para explicar qualquer palavra ou procedimentos que o senhor(a) não entenda claramente.**

O propósito deste documento é dar o senhor(a) as informações sobre a pesquisa e, se assinado, dará a sua permissão para participar do estudo. O documento descreve o objetivo, procedimentos, benefícios e eventuais riscos ou desconfortos caso queira participar. O senhor(a) só deve participar do estudo se quiser e pode se recusar a participar ou se retirar deste estudo a qualquer momento.

O objetivo desse estudo é determinar a incidência de alterações da função mental, especialmente da memória após anestesia geral e avaliar os efeitos da dexametasona sobre a

incidência de disfunção cognitiva (alterações da memória) em pacientes submetidos a cirurgia sob anestesia geral. O uso de corticóide antes da cirurgia pode diminuir a dor pós-operatória, além de diminuir a fadiga, a ocorrência de náuseas e vômitos, o período de recuperação e tempo de retorno às atividades diárias. Por outro lado, a dexametasona tem riscos de efeitos colaterais cardiovasculares, ósseos, retenção de líquidos, aumento de gorduras e glicose no sangue, mas como será usada em dose baixa e única não é de esperar a ocorrência de qualquer efeito colateral.

Antes da cirurgia o senhor(a) será sorteado para receber ou não dexametasona e os pacientes que receberam esta medicação serão comparados com os que não receberam esta medicação.

Participarão do estudo pessoas acima dos 60 anos que serão submetidas a cirurgia sob anestesia geral e que não tenham alteração da função mental e que não estejam usando cortisona. O senhor já foi anteriormente contactado por via telefônica e, se o senhor deu seu consentimento verbal para a pesquisa, respondeu algumas perguntas que indicaram que o senhor(a) pode participar desta pesquisa. Se o senhor concordar em continuar participando deste estudo, um psicólogo lhe aplicará alguns testes que o senhor responderá verbalmente no dia anterior à cirurgia, na noite do dia da cirurgia e no 3º, 7º, 21º e 90º dia após a cirurgia. Nestas datas, o senhor será convidado a comparecer ao hospital para a realização dos testes ou os mesmos serão aplicados por via telefônica.

O segundo objetivo desta pesquisa é a investigação da possível associação entre alterações mentais no pós-operatório e a presença de uma tendência hereditária para este fato, avaliada pela presença de proteínas específicas no sangue. Para tal haverá necessidade da coleta de uma quantidade de 6 mL de sangue que será dividido em 2 amostras e cada tubo com sangue será encaminhado a um laboratório para realização de exames específicos.

O senhor(a) não terá aumento de seu risco cirúrgico por participar desta pesquisa. A coleta das duas amostras de 3mL de sangue será feita no momento da punção da veia para administração da dexametasona antes da cirurgia. Esta mesma veia será utilizada durante a cirurgia para que o senhor receba anestésicos e soro. Durante a cirurgia, os controles feitos pelo anestesista incluirão a utilização de um aparelho já bastante utilizado em anestesia chamado monitor de índice bispectral (BIS) e que utiliza 2 eletrodos conectados na testa para determinação da profundidade da anestesia.

É através das pesquisas clínicas que ocorrem os avanços na medicina, e sua participação é de fundamental importância. Esta pesquisa não ira trazer benefícios diretos para o senhor(a), mas ela irá nos ajudar a melhor entender a alteração da função mental após cirurgia.

IV - ESCLARECIMENTOS DADOS PELO PESQUISADOR SOBRE GARANTIAS DO SUJEITO DA PESQUISA :

A sua participação neste estudo é voluntária. O senhor(a) tem a liberdade de recusar participar do estudo, ou se aceitar participar, retirar seu consentimento a qualquer momento. Este fato não implicará na interrupção de seu atendimento e tratamento, os quais estão assegurados.

Pela sua participação no estudo, o senhor não receberá qualquer valor em dinheiro ou terá qualquer custo. Em caso de eventuais danos à saúde decorrentes desta pesquisa, o senhor terá disponibilidade de assistência neste hospital.

As informações relacionadas ao estudo poderão ser inspecionadas pelos médicos que executam a pesquisa e pelas autoridades legais. No entanto, se qualquer informação for divulgada em relatório ou publicação, isto será feito sob forma codificada sem nomes. Esta medida assegura sua confidencialidade. Quando os resultados forem publicados, não aparecerá seu nome, e sim um código. O senhor(a) tem direito de acesso aos seus dados. O senhor(a) pode discutir esta questão mais adiante com seu médico do estudo. Se o senhor(a) ou seus parentes tiver(em) alguma dúvida com relação ao estudo, direitos do paciente, ou no caso de danos relacionados ao estudo, o senhor(a) deve contactar o pesquisador ou sua equipe .Se o senhor(a) tiver dúvidas sobre seus direitos como um paciente de pesquisa, você pode contactar o Comitê de Ética em Pesquisa em Seres Humanos do Hospital (CEP). A CEP trata-se de um grupo de indivíduos com conhecimento científicos e não científicos que realizam a revisão ética inicial e continuada do estudo de pesquisa para mantê-lo seguro e proteger seus direitos.

V. INFORMAÇÕES DE NOMES, ENDEREÇOS E TELEFONES DOS RESPONSÁVEIS PELO ACOMPANHAMENTO DA PESQUISA, PARA CONTATO EM CASO DE INTERCORRÊNCIAS CLÍNICAS E REAÇÕES ADVERSAS.

Dra Maria José Carvalho Carmona

Divisão de Anestesia do ICHC-FMUSP

Av Enéas Carvalho de Aguiar, n 255 – São Paulo

Fone: 11 3069-5012 ou 3069-6335

VI. OBSERVAÇÕES COMPLEMENTARES

VII - CONSENTIMENTO PÓS-ESCLARECIDO

Declaro que, após convenientemente esclarecido pelo pesquisador e ter entendido o que me foi explicado, consinto em participar do presente Protocolo de Pesquisa.

São Paulo, de de .

assinatura do sujeito da pesquisa ou responsável legal assinatura do pesquisador

(carimbo ou nome Legível)

I- Aprovação pela Comissão de Ética

HOSPITAL DAS CLÍNICAS

APROVAÇÃO

A Comissão de Ética para Análise de Projetos de Pesquisa - CAPPesq da Diretoria Clínica do Hospital das Clínicas e da Faculdade de Medicina da Universidade de São Paulo, em sessão de 01/08/2007, **APROVOU** o Protocolo de Pesquisa nº **998/06**, intitulado: **"DISFUNÇÃO COGNITIVA APÓS CIRURGIA SOB ANESTESIA GERAL"** apresentado pelo **DEPARTAMENTO DE CIRURGIA**, inclusive o Termo de Consentimento Livre e Esclarecido.

Cabe ao pesquisador elaborar e apresentar à CAPPesq, os relatórios parciais e final sobre a pesquisa (Resolução do Conselho Nacional de Saúde nº 196, de 10/10/1996, inciso IX.2, letra "c").

Pesquisador (a) Responsável: **Profa. Dra. Maria José Carvalho Carmona**

Pesquisadores Executantes: **Luiz Marcelo Sá Malbouisson, Kátia Osternack Pinto, José Otávio Costa Auler Jr.**

CAPPesq, 08 de Agosto de 2007

Prof.Dr. Eduardo Massad
Presidente da Comissão de Ética para Análise de Projetos de Pesquisa

Comissão de Ética para Análise de Projetos de Pesquisa do HCFMUSP e da FMUSP Diretoria Clínica do Hospital das Clínicas da Faculdade de Medicina da Universidade de São Paulo Rua Ovídio Pires de Campos, 255, 5º andar - CEP 05403 010 - São Paulo – SP Fone: 011 3069 6442 Fax: 011 3069 6492 e-mail: cappesq@hcnet.usp.br / secretariacappesq2@hcnet.usp.br -sol

Diretoria Clínica

Comissão de Ética para Análise de Projetos de Pesquisa - CAPPesq.

PARECER

PROTOCOLO DE PESQUISA Nº: 0998/06	Data da sessão: 31/08/11

TÍTULO DA PESQUISA: Disfunção cognitiva após cirurgia sob anestesia geral.
PESQUISADOR (A) RESPONSÁVEL: Maria José Carvalho Carmona

PESQUISADOR (A) EXECUTANTE: Luiz Marcelo Sá Malbouisson

DEPARTAMENTO: Cirurgia

CONSIDERAÇÕES DO RELATOR APROVADAS PELO PLENÁRIO:
Em carta datada de 09 de maio de 2011, os autores solicitam a mudança da finalidade acadêmica do projeto para Doutorado, com a mudança do pesquisador executante para Dra. Livia Stocco Sanches Valentin. Justificam a inclusão desta avaliação pois a presença de sintomas despressivos pode desencadear disfunção cogntiva pós operatória, capazes de interferir no pronto restabelecimento do paciente após a cirurgia.
Solicitam ainda a inclusão do inventário de depressão de Beck para a avaliação da depressão. Solicitam ainda a alteração das datas de avaliação neuropsicológica: à noite anterior à cirurgia e 3, 7, 21, 90 e 180 dias pós operatórios.
Os autores devem justificar como farão a interpretação dos resultados nos casos já operados e incluidos do estudo. Devem esclarecer se tratará de avaliação de sub-grupo.
O projeto deveria ter sido encerrado em 2008 e os autores devem refazer o cronograma do estudo e reencaminhar a esta Comissão.
O TCLE apresentado não segue a formatação da CAPPesq e deve ser corrigido.

CONCLUSÃO: Devolvido para atendimento às pendencias

HÁ NECESSIDADE DE ENVIAR Á CONEP	SIM ( )<br>INFORME A ÁREA TEMÁTICA:	NÃO (X)

AS RESPOSTAS ÁS PENDÊNCIAS, DEVERÃO SER APRESENTADAS NO PRAZO DE 60 DIAS.

PROF. DR. EUCLIDES AYRES DE CASTILHO
Coordenador
Comissão de Ética para Análise de Projetos de Pesquisa - CAPPesq

Comissão de Ética para Análise de Projetos de Pesquisa do HCFMUSP da Diretoria Clínica do Hospital das Clínicas da Faculdadede Medicina da Universidade de São Paulo
Rua Ovídio Pires de Campos. 225, 5º andar - CEP 05430 010 – São Paulo - SP
Fone: 011 - 30696442 011-30696431: ramais 16,17,18 e 20 fax : 011 - 3069 6492 ramal 26
e-mail : cappesq@hcnet.usp.br

São Paulo, 12 de Dezembro de 2011.

Ilmo. Sr.

Prof. Dr. Euclides Ayres de Castilho

D.D. Presidente da Comissão de Ética para Análise de Projetos de Pesquisa – CAPPesq - HCFMUSP

Ref.: Projeto de Pesquisa nº 998/06 – **"DISFUNÇÃO COGNITIVA APÓS CIRURGIA SOB ANESTESIA GERAL".**

Prezado Professor Euclides,

Em resposta às sugestões e solicitações apresentadas pelo digníssimo parecerista, considero valiosas as questões levantadas e encaminho as devidas correções e/ou esclarecimentos:

1) Foi inserida a pesquisadora Lívia Stocco Sanches Valentin como pesquisador executante no projeto de pesquisa por sua contribuição na coleta de dados e análise da bateria de testes neuropsicológicos como parte de sua pesquisa para obtenção do título de Doutora em Ciências junto ao Programa de Pós-Graduação em Anestesiologia. Foi também inserido no projeto de pesquisa o Inventário de Depressão de Beck (BDI) que consta de 21 questões que exploram sintomas de depressão numa escala de 0 a 4, na qual zero corresponde a ausência de sintomas e quatro corresponde ao nível máximo da sintomatologia. É um instrumento adaptado à população brasileira, mostrando-se particularmente adequado a investigação de um possível histórico pré mórbido para a patologia ou sintomas reativos ao fator estressante do evento cirúrgico.

2) Os quarenta (40) pacientes incluídos no estudo piloto deste projeto de pesquisa seguiram a versão original deste projeto de pesquisa e serão avaliados separadamente. Os demais casos serão submetidos aos testes mencionados nesta nova versão do projeto de pesquisa;

3) Em relação ao cronograma de execução do estudo, informamos que o atraso deve-se à demora para obtenção de financiamento ao estudo junto à FAPESP e dificuldade

no recrutamento de dados. O projeto FAPESP irá encerrar-se em março de 2013 (Processo número 09/54233-0) e prevê-se o encerramento do recrutamento de pacientes para o final de 2012.

4) Quanto ao TCLE, informamos que o mesmo foi revisto e está sendo apresentado com a nova redação e formatação sugerida. (Anexo A)

Agradeço antecipadamente a atenção dispensada e contribuições ao projeto de pesquisa e coloco-me à disposição para eventuais esclarecimentos que se fizerem necessários.

Atenciosamente

Profa. Dra. Maria José Carvalho Carmona
Pesquisador Gerente

RECEBIDO POR:
DATA: 06/01/12
Comissão de Ética para Análise de
Projeto de Pesquisa CAPPesq - HCFMUSP

HOSPITAL DAS CLÍNICAS DA FACULDADE DE MEDICINA DA UNIVERSIDADE DE SÃO PAULO-HCFMUSP

TERMO DE CONSENTIMENTO LIVRE E ESCLARECIDO

DADOS DE IDENTIFICAÇÃO DO SUJEITO DA PESQUISA OU RESPONSÁVEL LEGAL

1. NOME: .:... ....
DOCUMENTO DE IDENTIDADE Nº : .... SEXO : .M □ F □
DATA NASCIMENTO: ..../......../......
ENDEREÇO .... Nº .... APTO: ....
BAIRRO: .... CIDADE ....
CEP:.................................TELEFONE: DDD (............) ....
2. RESPONSÁVEL LEGAL ....
NATUREZA (grau de parentesco, tutor, curador etc.) ....
DOCUMENTO DE IDENTIDADE :....................................SEXO: M □ F □
DATA NASCIMENTO.: ....../......./......
ENDEREÇO: .... Nº .... APTO: ....
BAIRRO: .... CIDADE: ....
CEP: .... TELEFONE: DDD (............)..

DADOS SOBRE A PESQUISA

II - DADOS SOBRE A PESQUISA CIENTÍFICA

1. TÍTULO DO PROTOCOLO DE PESQUISA: **DISFUNÇÃO COGNITIVA APÓS CIRURGIA SOB ANESTESIA GERAL**
2. PESQUISADOR: Maria José Carvalho Carmona.

CARGO/FUNÇÃO: Professora Associada INSCRIÇÃO CONSELHO REGIONAL Nº 54.142

UNIDADE DO HCFMUSP: Divisão de Anestesia do Instituto Central

3. AVALIAÇÃO DO RISCO DA PESQUISA:

SEM RISCO □ RISCO MÍNIMO **X** RISCO MÉDIO □
RISCO BAIXO □ RISCO MAIOR □

(probabilidade de que o indivíduo sofra algum dano como consequência imediata ou tardia do estudo)

4. DURAÇÃO DA PESQUISA : .2 anos.

Rubrica do sujeito de pesquisa ou responsável_______

Rubrica do pesquisador_______

III - REGISTRO DAS EXPLICAÇÕES DO PESQUISADOR AO PACIENTE OU SEU REPRESENTANTE LEGAL SOBRE A PESQUISA:**O senhor (a) está sendo convidado(a) a participar de uma pesquisa, coordenada por um profissional da saúde agora denominado pesquisador. Para poder participar é necessário que o senhor(a) leia este documento com atenção. Ele pode conter palavras que o senhor(a) não entenda. Por favor peça aos responsáveis pelo estudo para explicar qualquer palavra ou procedimentos que o senhor(a) não entenda claramente.**

O propósito deste documento é dar o senhor(a) as informações sobre a pesquisa e, se assinado, dará a sua permissão para participar do estudo. O documento descreve o objetivo, procedimentos, benefícios e eventuais riscos ou desconfortos caso queira participar. O senhor(a) só deve participar do estudo se quiser e pode se recusar a participar ou se retirar deste estudo a qualquer momento.

O objetivo desse estudo é determinar a incidência de alterações da função mental, especialmente da memória após anestesia geral e avaliar os efeitos da dexametasona sobre a incidência de disfunção cognitiva (alterações da memória) em pacientes submetidos a cirurgia sob anestesia geral. O uso de corticóide antes da cirurgia pode diminuir a dor pós-operatória, além de diminuir a fadiga, a ocorrência de náuseas e vômitos, o período de recuperação e tempo de retorno às atividades diárias. Por outro lado, a dexametasona tem riscos de efeitos colaterais cardiovasculares, ósseos, retenção de líquidos, aumento de gorduras e glicose no sangue, mas como será usada em dose baixa e única não é de esperar a ocorrência de qualquer efeito colateral.

Antes da cirurgia o senhor(a) será sorteado para receber ou não dexametasona e os pacientes que receberam esta medicação serão comparados com os que não receberam esta medicação.

Participarão do estudo pessoas acima dos 60 anos que serão submetidas a cirurgia sob anestesia geral e que não tenham alteração da função mental e que não estejam usando cortisona. O senhor já foi anteriormente contatado por via telefônica e, se o senhor deu seu consentimento verbal para a pesquisa, respondeu algumas perguntas que indicaram que o senhor(a) pode participar desta pesquisa. Se o senhor concordar em continuar participando deste estudo, um psicólogo lhe aplicará alguns testes que o senhor responderá verbalmente no dia anterior à cirurgia, na noite do dia da cirurgia e no 3º, 7º, 21º e 90º dia após a cirurgia. Nestas datas, o senhor será convidado a comparecer ao hospital para a realização dos testes ou os mesmos serão aplicados por via telefônica.

O segundo objetivo desta pesquisa é a investigação da possível associação entre alterações mentais no pós-operatório e a presença de uma tendência hereditária para este fato, avaliada pela presença de proteínas específicas no sangue. Para tal haverá necessidade da coleta de uma quantidade de 6 mL de sangue que será dividido em 2 amostras e cada tubo com sangue será encaminhado a um laboratório para realização de exames específicos.

O senhor(a) não terá aumento de seu risco cirúrgico por participar desta pesquisa. A coleta das duas amostras de 3mL de sangue será feita no momento da punção da veia para administração da dexametasona antes da cirurgia. Esta mesma veia será utilizada durante a cirurgia para que o senhor receba anestésicos e soro. Durante a cirurgia, os controles feitos pelo anestesista incluirão a utilização de um aparelho já bastante utilizado em anestesia chamado monitor de índice bispectral (BIS) e que utiliza 2 eletrodos conectados na testa para determinação da profundidade da anestesia.

É através das pesquisas clínicas que ocorrem os avanços na medicina, e sua participação é de fundamental importância. Esta pesquisa não ira trazer benefícios diretos para o senhor(a), mas ela irá nos ajudar a melhor entender a alteração da função mental após cirurgia.

IV - ESCLARECIMENTOS DADOS PELO PESQUISADOR SOBRE GARANTIAS DO SUJEITO DA PESQUISA:

A sua participação neste estudo é voluntária. O senhor(a) tem a liberdade de recusar participar do estudo, ou se aceitar participar, retirar seu consentimento a qualquer momento. Este fato não implicará na interrupção de seu atendimento e tratamento, os quais estão assegurados.

Pela sua participação no estudo, o senhor não receberá qualquer valor em dinheiro ou terá qualquer custo. Em caso de eventuais danos à saúde decorrentes desta pesquisa, o senhor terá disponibilidade de assistência neste hospital.

As informações relacionadas ao estudo poderão ser inspecionadas pelos médicos que executam a pesquisa e pelas autoridades legais. No entanto, se qualquer informação for divulgada em relatório ou publicação, isto será feito sob forma codificada sem nomes. Esta medida assegura sua confidencialidade. Quando os resultados forem publicados, não aparecerá seu nome, e sim um código. O senhor(a) tem direito de acesso aos seus dados. O senhor(a) pode discutir esta questão mais adiante com seu médico do estudo. Se o senhor(a) ou seus parentes tiver(em) alguma dúvida com relação ao estudo, direitos do paciente, ou no caso de danos relacionados ao estudo, o senhor(a) deve contatar o pesquisador ou sua equipe .Se o senhor(a) tiver dúvidas sobre seus direitos como um paciente de pesquisa, você pode contatar o Comitê de Ética em Pesquisa em Seres Humanos do Hospital (CEP). A CEP trata-se de um grupo de indivíduos com conhecimento científicos e não científicos que realizam a revisão ética inicial e continuada do estudo de pesquisa para mantê-lo seguro e proteger seus direitos.

V. INFORMAÇÕES DE NOMES, ENDEREÇOS E TELEFONES DOS RESPONSÁVEIS PELO ACOMPANHAMENTO DA PESQUISA, PARA CONTATO EM CASO DE INTERCORRÊNCIAS CLÍNICAS E REAÇÕES ADVERSAS.

Dra Maria José Carvalho Carmona
Divisão de Anestesia do ICHC-FMUSP
Av Enéas Carvalho de Aguiar, n 255 – São Paulo
Fone: 11 3069-5012 ou 3069-6335

VI. OBSERVAÇÕES COMPLEMENTARES

VII - CONSENTIMENTO PÓS-ESCLARECIDO

Declaro que, após convenientemente esclarecido pelo pesquisador e ter entendido o que me foi explicado, consinto em participar do presente Protocolo de Pesquisa.

São Paulo, de de .

____

assinatura do sujeito da pesquisa ou responsável legal assinatura do pesquisador

(carimbo ou nome Legível)

Rubrica do sujeito de pesquisa ou responsável_______

Rubrica do pesquisador_______

HOSPITAL DAS CLÍNICAS
DA FACULDADE DE MEDICINA
DA UNIVERSIDADE DE SÃO PAULO

Diretoria Clínica

Comissão de Ética para Análise de Projetos de Pesquisa - CAPPesq.

PARECER

PROTOCOLO DE PESQUISA Nº: 0998/06	Data da sessão: 01/02/12

TÍTULO DA PESQUISA: Disfunção cognitiva após cirurgia sob anestesia geral.
PESQUISADOR (A) RESPONSÁVEL: Maria José Carvalho Carmona

PESQUISADOR (A) EXECUTANTE: Luiz Marcelo Sá Malbouisson

DEPARTAMENTO: Cirurgia

CONSIDERAÇÕES DO RELATOR APROVADAS PELO PLENÁRIO:

Em carta datada de 12 de dezembro de 2011, os autores respondem às pendências do parecer de 31 de agosto de 2011.

Justificam o atraso no cronograma de execução do estudo pelo atraso na obtenção do financiamento junto à FAPESP, que foi concedido (processo número 09/54233-0). O financiamento prevê o encerramento da pesquisa em março de 2013 e do recrutamento para o final de 2012.

O TCLE apresentado ainda não segue a formatação da CAPPesq e deve ser corrigido.

CONCLUSÃO: Providenciar formatação adquada do TCLE

HÁ NECESSIDADE DE ENVIAR Á CONEP	SIM ( )<br>INFORME A ÁREA TEMÁTICA:	NÃO (X)

PROF. DR. LUIZ EUGÊNIO GARCEZ LEME
Coordenador
Comissão de Ética para Análise de Projetos de Pesquisa - CAPPesq

PG/MA1/030/2012

São Paulo, 02 de agosto de 2012.

Ilmo. Sr.

Prof. Dr. Luiz Eugênio Garcez Leme

Coordenador da Comissão de Ética para Análise de Projetos de Pesquisa

CAPPESq

Ref.: **Protocolo n° 0998/06 envio de TCLE – titulo: Disfunção cognitiva após cirurgia sob anestesia geral.**

Prezado Professor,

Em resposta ao parecer, datado em 01 de fevereiro de 2012, do referido protocolo de pesquisa, encaminho a esta Comissão o Termo de Consentimento Livre e Esclarecido elaborado de acordo com a formatação existente no site da Comissão de Ética para análise de projetos de pesquisa.

Sem mais, coloco-me a disposição para quaisquer esclarecimentos, atenciosamente.

Profa. Dra. Maria José Carvalho Carmona

Investigador Principal

RECEBIDO POR: [illegible]
DATA: 03 / 08 / 12
Comissão de Ética para Análise de
Projeto de Pesquisa-CAPPesq - HCFMUSP

HC HOSPITAL DAS CLÍNICAS
DA FACULDADE DE MEDICINA
DA UNIVERSIDADE DE SÃO PAULO

Diretoria Clínica

Comissão de Ética para Análise de Projetos de Pesquisa - CAPPesq.

PARECER

PROTOCOLO DE PESQUISA Nº: 0998/06	Data da sessão: 19/09/2012

TÍTULO DA PESQUISA: Disfunção cognitiva após cirurgia sob anestesia geral.

PESQUISADOR (A) RESPONSÁVEL: Maria José Carvalho Carmona

PESQUISADOR (A) EXECUTANTE: Luiz Marcelo Sá Malbouisson

DEPARTAMENTO: Cirurgia

CONSIDERAÇÕES DO RELATOR APROVADAS PELO PLENÁRIO:

Em carta datada de 12 de dezembro de 2011, os autores respondem às pendências do parecer de 31 de agosto de 2011.

Justificam o atraso no cronograma de execução do estudo pelo atraso na obtenção do financiamento junto à FAPESP, que foi concedido (processo número 09/54233-0). O financiamento prevê o encerramento da pesquisa em março de 2013 e do recrutamento para o final de 2012.

O TCLE apresentado segue a formatação da CAPPesq.

CONCLUSÃO: Aprovado.

HÁ NECESSIDADE DE ENVIAR Á CONEP	SIM ( ) INFORME A ÁREA TEMÁTICA:	NÃO (X)

Comissão de Ética para Análise de Projetos de Pesquisa do HCFMUSP da Diretoria Clínica do Hospital das Clínicas da Faculdade de Medicina da Universidade de São Paulo
Rua Ovídio Pires de Campos, 225, 5º andar - CEP 05430 010 – São Paulo - SP
Fone: 011 - 30696442 011-30696431: ramais 16,17,18 e 20 fax : 011 - 3069 6492 ramal 26
e-mail : cappesq@hcnet.usp.br

Hospital das Clínicas da FMUSP
Comissão de Ética para Análise de Projetos de Pesquisa
CAPPesq

Nº Protocolo: 998/06
Título: "Disfunção cognitiva após cirurgia sob anestesia geral"
Pesquisador Responsável: Maria José Carvalho Carmona
Pesquisador Executante: Luiz Marcelo Sá Malbouisson
Departamento: CIRURGIA

A Comissão de Ética para Análise de Projetos de Pesquisa – CAPPesq da Diretoria Clínica do Hospital das Clínicas da Faculdade de Medicina da Universidade de São Paulo, **APROVOU / TOMOU CIÊNCIA** na sessão datada de 19/09/2012, do(s) documento(s) abaixo mencionado(s):

PG/MA1/020/2011:
- **Finalidade acadêmica: Doutorado**
- **Pesquisador Responsável/Orientador: Profa. Dra. Maria José Carvalho Carmona (Lattes: 2636111337875377)**
- **Pesquisador Executante: Dra. Lívia Stocco Sanches Valentin (Lattes: 1665879506366648)**
- **Inclusão do teste: avaliação de depressão (BDI – Inventário de Depressão de Beck)**
- **Alteração das datas de avaliação neuropsicológica: à noite anterior à cirurgia e 3°, 7°, 21°, 90° 180° dias pós-operatórios.**

A CAPPesq em obediência à Resolução CNS 196/96, solicita ao pesquisador (a) s elaboração de relatório parcial e final.

No caso de relatório parcial é necessário informar o tempo previsto para a conclusão do protocolo e breve resumo dos resultados obtidos.

CAPPesq, 19 de Setembro de 2012

PROF. DR. LUIZ EUGÊNIO GARCEZ LEME
Coordenador
Comissão de Ética para Análise de Projetos de Pesquisa - CAPPesq

Rua Dr. Ovídio Pires de Campos, 225 - Prédio da Administração - 5º andar - CEP 05403-010 - São Paulo - SP.
55 11 2661-7585 - 55 11 2661-6442 ramais: 16, 17, 18 | marcia.carvalho@hc.fm.usp.br

HOSPITAL DAS CLÍNICAS DA FACULDADE DE MEDICINA DA UNIVERSIDADE DE SÃO PAULO-HCFMUSP

TERMO DE CONSENTIMENTO LIVRE E ESCLARECIDO

I- DADOS DE IDENTIFICAÇÃODO SUJEITO DA PESQUISA OU RESPONSÁVEL LEGAL

1. NOME: ____
DOCUMENTO DE IDENTIDADE Nº : ____________________ SEXO : .M □ F □
DATA NASCIMENTO: ____/____/____
ENDEREÇO ______________________________ Nº _____ APTO: _____
BAIRRO: ____________________ CIDADE ____________________
CEP:____________ TELEFONE: DDD (_____) ______________
2. RESPONSÁVEL LEGAL ______________________________________
NATUREZA (grau de parentesco, tutor, curador etc.) ______________
DOCUMENTO DE IDENTIDADE :____________________ SEXO: M □ F □
DATA NASCIMENTO.: ____/____/____
ENDEREÇO: ______________________________ Nº _____ APTO: _____
BAIRRO: ____________________ CIDADE:____________________
CEP: ____________ TELEFONE: DDD (_____)______________

DADOS SOBRE A PESQUISA

II - DADOS SOBRE A PESQUISA CIENTÍFICA

1. TÍTULO DO PROTOCOLO DE PESQUISA: **DISFUNÇÃO COGNITIVA APÓS CIRURGIA SOB ANESTESIA GERAL**

2. PESQUISADOR: Maria José Carvalho Carmona.

CARGO/FUNÇÃO: Professora Associada INSCRIÇÃO CONSELHO REGIONAL Nº 54.142

UNIDADE DO HCFMUSP: Divisão de Anestesia do Instituto Central

3. AVALIAÇÃO DO RISCO DA PESQUISA:

SEM RISCO □ RISCO MÍNIMO **X** RISCO MÉDIO □
RISCO BAIXO □ RISCO MAIOR □

(probabilidade de que o indivíduo sofra algum dano como consequência imediata ou tardia do estudo)

4. DURAÇÃO DA PESQUISA : 2 anos.

Rubrica do sujeito de pesquisa ou responsável_______

Rubrica do pesquisador_______

III-REGISTRO DAS EXPLICAÇÕES DO PESQUISADOR AO PACIENTE OU SEU REPRESENTANTE LEGAL SOBRE A PESQUISA:

O senhor(a) está sendo convidado a participar de uma pesquisa, coordenada por um profissional de saúde agora denominado pesquisador. Para poder participar é necessário que o senhor(a) leia este documento com atenção. O propósito deste documento é dar ao senhor(a) as informações sobre a pesquisa e, se assinado, dará a sua permissão para participar do estudo.

O objetivo desse estudo é verificar se houve alterações da função mental, especialmente da memória e avaliar os efeitos da dexametasona sobre a memória do paciente submetido a cirurgia sob anestesia geral. O uso de corticoide (dexametasona) antes da cirurgia pode diminuir a dor pós-operatória, a fadiga, a ocorrência de náuseas, vômitos, encurtar o período de recuperação e o tempo de retorno às atividades diárias.

Antes da cirurgia o senhor(a) será sorteado para receber ou não a dexametasona. Os pacientes que receberam esta medicação serão comparados com os que não receberam esta medicação. Para fazer esta comparação existe a necessidade da coleta de uma quantidade mínima de sangue, aproximadamente 6ml, que será dividido em 2 amostras, e será retirado no momento antes da cirurgia. Cada tubo com sangue será encaminhado a um laboratório para realização de exames específicos. O senhor(a) não terá aumento de seu risco cirúrgico por participar desta pesquisa.

Participarão do estudo pessoas acima dos 60 anos que serão submetidas a cirurgia sob anestesia geral e que não tenham alteração da função mental e que não estejam usando cortisona. O senhor(a) foi convidado por um de nossos pesquisadores a participar da pesquisa, porque as suas características correspondem aos critérios para participar do estudo. As avaliações serão feitas por um psicólogo por um período de 6(seis) meses. Nos 3º, 7º e 90º dias o psicólogo o avaliará por telefone e nos 21º e 180º após a cirurgia o senhor(a) será convidado a realizar a avaliação no HC-FMUSP em horário e dia agendados para sua comodidade.

É através das pesquisas clínicas que ocorrem os avanços na medicina, e sua participação é de fundamental importância para este progresso. Esta pesquisa não ira trazer benefícios diretos para o senhor(a), nem tão pouco a opção de procedimentos alternativos, mas ela irá nos ajudar a melhor entender a alteração da função mental após cirurgia.

As informações relacionadas ao estudo poderão ser inspecionadas pelos médicos que executam a pesquisa e pelas autoridades legais. O(A) senhor(a) pode ter acesso aos seus dados e discutir esta questão futuramente com seu médico. O investigador principal é a Profa. Dra. Maria José Carvalho Carmona, a pesquisadora executante é a psicóloga Lívia Stocco Sanches Valentin, que podem ser encontradas na Divisão de Anestesia do ICHC-FMUSP na Av. Enéas Carvalho de Aguiar, nº 255- São Paulo ou pelos telefones: (11) 2661-5367 / 2661-6335. Se o(a) senhor(a) tiver qualquer dúvida ou consideração sobre a ética da pesquisa, entre em contato com o Comitê de Ética em Pesquisa (CEP)-Rua Ovídio Pires de Campos, 225-5ºandar –tel: 2661-6442, ramais 16, 17, 18- email:cappesq@hcnet.usp.br.

A sua participação neste estudo é voluntária. O senhor(a) tem a liberdade de recusar a participar do estudo, ou se aceitar a participar, tem o direito de retirar seu consentimento a qualquer momento. Este fato não implicará na interrupção de seu atendimento e tratamento, os quais estão assegurados.

Para garantir a confidencialidade dos dados da pesquisa as informações obtidas pelos resultados serão analisadas no total, não sendo divulgados resultados individuais e serão apresentados sob forma de código e sem nomes.

O(A) senhor(a) tem o direito de ser mantido atualizado sobre os resultados parciais e andamento da pesquisa, sempre que possível, quando esta informação for de conhecimento e acesso do pesquisador.

Rubrica do sujeito de pesquisa ou responsável_______

Rubrica do pesquisador_______

O(A) senhor(a) não receberá qualquer valor em dinheiro ou terá qualquer custo em participar desta pesquisa. Em caso de eventuais danos à saúde decorrentes deste estudo, o senhor terá disponibilidade de assistência neste hospital.

O pesquisador assume o compromisso de utilizar os dados e o material coletado de maneira ética e estritamente para esta pesquisa.

IV-CONSENTIMENTO PÓS-ESCLARECIDO

Acredito ter sido suficientemente informado a respeito das informações que li ou que foram lidas para mim, descrevendo o estudo **DISFUNÇÃO COGNITIVA APÓS CIRURGIA SOB ANESTESIA GERAL.**

Eu discuti com Lívia S. S. Valentin (pesquisador executante) sobre minha decisão em participar deste estudo. Ficaram claros para mim quais são os propósitos do estudo, os procedimentos a serem realizados, seus desconfortos e riscos, as garantias de confidencialidade e de esclarecimentos permanentes. Ficou claro também que minha participação é isenta de despesas e que tenho garantia ao acesso ao tratamento hospitalar se e quando necessário. Concordo voluntariamente em participar deste estudo e poderei retirar o meu consentimento a qualquer momento, antes ou durante o mesmo, sem penalidades ou prejuízo ou perda de qualquer benefício que eu possa ter adquirido, ou no meu atendimento neste Serviço.

Assinatura do paciente/representante legal Data ___/___/___

Assinatura da testemunha Data ___/___/___

para casos de pacientes menores de 18 anos, analfabetos, semi-analfabetos ou portadores de deficiência auditiva ou visual.

(Somente para o responsável do projeto)

Declaro que obtive de forma apropriada e voluntária o Consentimento Livre e Esclarecido deste paciente ou representante legal para a participação neste estudo.

Assinatura do responsável pelo estudo Data ___/___/___

HOSPITAL DAS CLÍNICAS
DA FACULDADE DE MEDICINA
DA UNIVERSIDADE DE SÃO PAULO

Diretoria Clínica

Comissão de Ética para Análise de Projetos de Pesquisa - CAPPesq.

PARECER

PROTOCOLO DE PESQUISA Nº: 0998/06	Data da sessão: 01/02/12

TÍTULO DA PESQUISA: Disfunção cognitiva após cirurgia sob anestesia geral.
PESQUISADOR (A) RESPONSÁVEL: Maria José Carvalho Carmona

PESQUISADOR (A) EXECUTANTE: Luiz Marcelo Sá Malbouisson

DEPARTAMENTO: Cirurgia

CONSIDERAÇÕES DO RELATOR APROVADAS PELO PLENÁRIO:

Em carta datada de 12 de dezembro de 2011, os autores respondem às pendências do parecer de 31 de agosto de 2011.

Justificam o atraso no cronograma de execução do estudo pelo atraso na obtenção do financiamento junto à FAPESP, que foi concedido (processo número 09/54233-0). O financiamento prevê o encerramento da pesquisa em março de 2013 e do recrutamento para o final de 2012.

O TCLE apresentado ainda não segue a formatação da CAPPesq e deve ser corrigido.

CONCLUSÃO: Providenciar formatação adquada do TCLE

HÁ NECESSIDADE DE ENVIAR Á CONEP	SIM ( )<br>INFORME A ÁREA TEMÁTICA:	NÃO (X)

PROF. DR. LUIZ EUGÊNIO GARCEZ LEME
Coordenador
Comissão de Ética para Análise de Projetos de Pesquisa - CAPPesq

São Paulo, 20 de Novembro de 2014.

Ilmo. Sr.
Prof. Dr. José Alfredo Mansur
D.D. Presidente de Ética para Análise de Projetos de Pesquisa
HCFMUSP

Ref: Subprojeto do Projeto de Pesquisa nº 998/06**: Disfunção Cognitiva após cirurgia sob anestesia geral.**

Prezado Professor,

Solicito análise do subprojeto **"Avaliação cognitiva e da ocorrência de disfunção cognitiva pós operatória em pacientes cirúrgicos analfabetos ou de baixa escolaridade"**, integrante do Projeto de Pesquisa "**Disfunção Cognitiva após cirurgia sob anestesia geral".** Para este subprojeto solicito a inclusão da aluna de graduação **Jéssica Kazumi Okuma**.

Informo que os dados a serem analisados neste subprojeto referem-se a pacientes recrutados no projeto de pesquisa acima referido, mas não randomizados para o estudo devido ao grau de escolaridade**.** Por terem se caracterizado como um grupo especial dentro da amostra recrutada com resultados específicos em relação à avaliação neuropsicológica, sugeriu-se a análise separada dos dados, com o objetivo específico de avaliação da ocorrência de disfunção cognitiva e da aplicabilidade de métodos de avaliação neuropsicológica nesse grupo de analfabetos ou com baixa escolaridade.

Em anexo, encaminho o subprojeto, a versão final do Projeto de Pesquisa original e o paper que está em fase de publicação. Informo que os dados referentes à avaliação da APOe4 não foram inclusos no paper devido atraso na liberação dos resultados. A tese referente ao estudo original será defendida pela pesquisadora executante do projeto original Lívia Stocco Sanches Valentin.

Agradeço antecipadamente e coloco-me à disposição para esclarecimentos adicionais que se fizerem necessários.

Atenciosamente

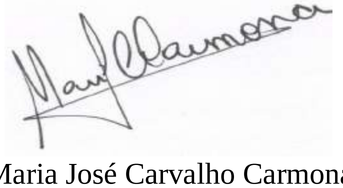

Maria José Carvalho Carmona
Pesquisador Gerente

DISCIPLINA DE ANESTESIOLOGIA
FACULDADE DE MEDICINA
UNIVERSIDADE DE SÃO PAULO

<u>Subprojeto</u>: Avaliação cognitiva pós operatória em pacientes cirúrgicos analfabetos ou de baixa escolaridade

<u>Projeto de Pesquisa</u> (CAPPesq nº 998/06): Disfunção Cognitiva após cirurgia sob anestesia geral.

Pesquisador Gerente: Profa. Dra. Maria José Carvalho Carmona

Outros Pesquisadores envolvidos:

- **Lívia Stocco Sanches Valentin – Aluna de Pós-Graduação**
- **Jéssica Kazumi Okuma – Aluna de Graduação**

São Paulo
2014

Avaliação cognitiva pós operatória em pacientes cirúrgicos analfabetos ou de baixa escolaridade –São Paulo, 2014.Faculdade de Medicina da Universidade de São Paulo.

RESUMO

INTRODUÇÃO: Um número significativo de pacientes submetidos a cirurgias com anestesia geral pode apresentar disfunção cognitiva no período pós-operatório. Esta disfunção pode ser transitória e reversível, mas pode ser de longa duração em alguns casos, especialmente em idosos. A disfunção cognitiva pós-operatória (POCD) envolve alterações de atenção, memória, velocidade do processamento de informações e alguns aspectos da função executiva. Além da idade, a etiologia da POCD é multifatorial e controversa. Pacientes com baixa escolaridade e/ou com baixa reserva cognitiva são descritos como mais vulneráveis à ocorrência de POCD. Este sub-projeto faz parte do projeto de pesquisa aprovado pela Comissão de Ética Institucional (CAPPesq- processo nº 998/06), que teve como objetivo primário avaliar o efeito da dexametasona e da profundidade da anestesia sobre a incidência de POCD em pacientes idosos submetidos a cirurgias não cardíacas eletivas sob anestesia geral. Dentre os pacientes recrutados para este estudo, observou-se alta prevalência de pacientes analfabetos ou de baixa escolaridade. Apesar de terem sido avaliados, os casos não foram analisados por não terem alcançado a pontuação mínima nos testes neuropsicológicos. Considerado como um grupo com características peculiares, optou-se pela avaliação específica dos resultados obtidos neste grupo de pacientes. **OBJETIVOS**: O objetivo deste subprojeto é a avaliação cognitiva pós operatória em pacientes idosos analfabetos ou de baixa escolaridade submetidos a cirurgias eletivas sob anestesia geral.

CASUÍSTICA E MÉTODOS: O Projeto original propôs o recrutamento de 300 pacientes. Dentre os pacientes recrutados para o estudo original, serão avaliados os resultados dos casos que não atingiram a pontuação mínima nos testes neuropsicológicos e que não foram submetidos à randomização proposta no estudo. Trata-se de pacientes com idade superior a 60 anos e submetidos a cirurgias de médio porte sob anestesia geral. Os pacientes foram avaliados por testes psicológicos e neuropsicológicos para aferir a qualidade de vida, sintomas depressivos, o estado mental geral, a capacidade de aprendizagem, atenção e percepção, memória imediata, operacional e de evocação e habilidades executivas. A avaliação foi realizada antes da cirurgia, e nos 3º, 7º, 21º, 90º, 180º dias pós-operatórios. Os resultados dos escores dos testes em cada grupo serão comparados através do teste de Friedmann, e entre os grupos será utilizado o teste de Kruskal Wallis. Porcentagens serão comparadas pelo do teste de Qui-Quadrado. Os dados serão expressos como mediana e intervalo de confiança de 95%. Serão considerados significativos resultados cujos valores p sejam menores que 0,05 nos testes estatísticos.

Palavras Chave: POCD, Idosos, Analfabetismo, Funções Neuropsicológicas

Avaliação cognitiva e da ocorrência de disfunção cognitiva pós operatória em pacientes cirúrgicos analfabetos ou de baixa escolaridade –São Paulo, 2014.Faculdade de Medicina da Universidade de São Paulo.

1) Introdução:

No mundo 774 milhões de pessoas não possuem acesso à comunicação escrita por meio da alfabetização, aproximadamente 85% da população mundial considerada analfabeta está localizada em 35 países. No Brasil o número entre pessoas maiores de 15 anos analfabetas representa cerca de 16 milhões, sendo que metade desta população está concentrada em menos de 10% do municípios[1]. O baixo nível educacional é também comum na população idosa brasileira, estimando-se que cerca de 30% dos idosos brasileiros sejam analfabetos[2].

O baixo nível educacional e o envelhecimento são apontados como dois importantes fatores para o desenvolvimento de déficits cognitivos, entre eles a disfunção cognitiva pós operatória(DCPO)[3-4]. Estudos mostram que a capacidade cognitiva pré cirúrgica e a presença de distúrbios congnitivos prévios tem um potencial valor preditivo de disfunções neurocognitivas que podem surgir no período pós-operatório tardio[5]. Tem-se então sugerido que uma avaliação de funções neuropsicológicas do paciente seja essencial no período pré-operatório para avaliação do risco de desenvolvimento de disfunções cognitivas no pós-operatório[6].

Baixo nível educacional da população idosa brasileira pode afetar a avaliação cognitiva, tanto em relação ao envelhecimento normal como patológico. Pacientes idosos com baixa escolaridade podem ser classificados erroneamente como portadores de danos cerebrais, por apresentar desempenhos inferiores aos que os testes podem aferir. Porém, esses desempenhos remetem a escassez de testes adequados para a medição de analfabetos[7].

Diante do acelerado crescimento da população idosa no contexto brasileiro e os avanços da medicina para o atendimento cirúrgico desta população, verifica-se a

grande importância de disponibilizar instrumentos adequados, tanto para a pesquisa quanto para a avaliação neuropsicológica de idosos, principalmente em funções marcadoras de processos patológicos, buscando o pronto restabelecimento após intervenções cirúrgicas para uma melhor qualidade de vida .

Este sub-projeto faz parte do projeto de pesquisa aprovado pela Comissão de Ética Institucional (CAPPesq- processo nº 998/06), que teve como objetivo primário avaliar o efeito da dexametasona e da profundidade da anestesia sobre a incidência de DCPO em pacientes idosos submetidos a cirurgias não cardíacas eletivas sob anestesia geral. Dentre os pacientes recrutados para este estudo, observou-se alta prevalência de pacientes analfabetos ou de baixa escolaridade. Apesar de terem sido avaliados, os casos não foram utilizados no estudo acima por não terem alcançado a pontuação mínima nos testes neuropsicológicos. Considerado como um grupo particular, sugeriu-se a avaliação específica dos resultados obtidos neste grupo de pacientes, bem como discutir a aplicabilidade dos métodos atualmente disponíveis para avaliação neuropsicológica.

2) Objetivos

1) Analisar a ocorrência de DCPO em pacientes analfabetos ou de baixa escolaridade

2) Analisar e discutir a aplicação dos métodos de avaliação neuropsicológica e cognitiva nesse grupo específico de indivíduos.

4) Casuística e Métodos

Procedimentos

O protocolo do estudo original foi aprovado pela Comissão de Ética Médica do Hospital das Clínicas da Faculdade de Medicina da Universidade de São Paulo (CAPPesq - projeto de pesquisa nº: 998/06) e recebeu auxílio à pesquisa do Fundo de Amparo à Pesquisa do Estado de São Paulo. (Processo FAPESP nº: 09/54233-0) e os resultados encontram-se em fase de publicação. Foram estudados 300 pacientes idosos de ambos os sexos submetidos à anestesia geral para procedimentos cirúrgicos.

Foram aplicados testes de qualidade de vida, depressão e bateria neuropsicológica para avaliar o estado mental geral, aprendizagem, atenção, percepção visuoespacial, memória imediata, operacional e de evocação e habilidades executivas, incluindo velocidade de processamento. Esta bateria define o índice cognitivo da estabilidade (medida longitudinal do status cognitivo em avaliações repetidas que detectam mudanças estatisticamente significantes no funcionamento do sistema nervoso central). A avaliação neuropsicológica foi realiza na noite anterior à cirurgia e nos dias pós-operatórios 3, 7, 21, 90 e 180, sendo que os profissionais responsáveis pelos testes neuropsicológicos não tiveram conhecimentos dos grupos aos quais pertencem os pacientes.

Participantes

Dentre os participantes do estudo original, foram excluídos aqueles que antes da avaliação pré-operatória não atingiram a pontuação mínima no MMEE. Posteriormente, após assinatura do TCLE e antes da randomização, foram excluídos do estudo original aqueles que apresentaram baixo desempenho nos testes subsequentes.

Esses pacientes serão analisados nesse subprojeto quanto à ocorrência de DCPO e quanto aos escores obtidos nos testes neuropsicológicos.

Termo de consentimento

Os pacientes que preencheram os critérios de inclusão e aceitaram participar do estudo original foram submetidos aos testes neuropsicológicos. Após definição da indicação cirúrgica e avaliação dos critérios de inclusão e exclusão, os pacientes foram convidados a participar da pesquisa. Após esclarecimentos em relação aos objetivos gerais do estudo, assinaram o termo de consentimento livre e esclarecido.

Metodologia

Os dados serão analisados utilizando-se o software de análise estatística SPSS versão 10. Os dados demográficos como idade, peso, altura serão comparados através de análise de variância univariada seguida de teste de comparações múltiplas se aplicável. As variáveis colhidas através de testes neuropsicológicos aplicados antes e depois do procedimento cirúrgico são de natureza qualitativa ou ordinal, sendo atribuídos resultados numéricos e os resultados finais dos testes serão expressos em

escores. Os resultados dos escores dos testes serão comparados através do teste de Friedmann. Mann Whitney para comparações da evolução do sujeito. Porcentagens serão comparadas através do teste de Qui-Quadrado. Os dados serão expressos como mediana e intervalo de confiança de 95%. Serão considerados significativos resultados cujos valores p sejam menores que 0,05 nos testes estatísticos.

Instrumentos

Foram utilizados como instrumentos:

1) Informações Sócio-demográficas – Serão investigadas as informações sobre idade, sexo, escolaridade, estado civil, ocupação, e demais informações relevantes para compor o perfil sócio demográfico da amostra, bem como a compatibilidade com os critérios de exclusão e inclusão, conforme detalhado em anexo;

2)Versão Brasileira do Questionário de Qualidade de Vida SF-36 – O SF-36 investiga condições relacionadas à saúde, atividades de vida diária, produtividade, problemas emocionais, relacionamentos, motivação e outras condições que traçam o perfil da qualidade de vida a partir da percepção do próprio sujeito, conforme apresentado em anexo;

3) Mini Exame do Estado Mental- MMEE é um instrumento para breve *screening* que permite avaliar e quantificar o cognitivo do paciente. A pontuação será de acordo com a padronização brasileira com pontos de corte.

4) Teste de Palavras coloridas de Stroop- (Stroop colours Word Test) Serão avaliados, o tempo dispendido e o número de erros em cada cartão. Consiste na apresentação de três lâminas ao sujeito. Na primeira lâmina o sujeito deve verbalizar os nomes das cores impressas com tinta preta. Na segunda, a verbalização é feita da cor que estão preenchidos retângulos, na mesma disposição das palavras da lâmina anterior. A terceira consiste em verbalizar as cores impressas em detrimento da palavra escrita. Avalia atenção seletiva, capacidade inibitória e flexibilidade mental.

5) Trail Making Test (TMT)- Este teste é composto por duas partes. Na parte A, o sujeito deve traçar linhas conectando consecutivamente círculos numerados. Na parte B, o sujeito deve traçar linhas conectando alternadamente círculos com letras e números em uma sequência ordenada. O teste envolve (Parte A) rastreio visual complexo e velocidade motora e, na parte B, processos executivos. Entre os processos executivos, a capacidade inibitória e a alternância cognitiva parecem ser aqueles mais

envolvidos na realização da parte B. Serão avaliados o tempo dispendido e o número de erros cometidos em cada parte.

6) Subteste Digit Symbol do Wechsler de Inteligência- Scale-Revised- Este teste afere a memória de curto prazo, habilidade visuoespacial e atenção. É uma tarefa gráfica realizada em 180 segundos. O indivíduo tem que reproduzir os símbolos exemplificados em uma tarjeta nos espaços abaixo do número correspondente.

7) Telephone Interview for Cognitive Status – TICS – É um teste padronizado para avaliação do funcionamento neuropsicológico que foi desenvolvido para utilizar em situações onde a avaliação de rastreio cognitivo realizada pessoalmente é impraticável ou ineficiente, como, por exemplo, em pesquisas epidemiológicas de grandes populações ou com pacientes que estão incapacitados de comparecerem ao retorno clínico. É muito útil também para avaliar pessoas com incapacidade para ler ou escrever, pois pode ser aplicado pessoalmente através de entrevista, exigindo apenas capacidade de compreensão verbal. Consiste em um roteiro de entrevista com onze itens abordando as habilidades de orientação espacial, temporal, controle mental, memória, informação geral, repetição de sentenças, memória semântica, praxias e antônimos de palavras, conforme detalhado em anexo.

8) Inventário de Depressão de Beck – BDI – Consta de 21 questões que exploram sintomas de depressão numa escala de 0 a 4, na qual zero corresponde a ausência de sintomas e quatro corresponde ao nível máximo da sintomatologia. Um total de 18 pontos tem sido considerado indicativo da presença de sintomatologia depressiva. Foi desenvolvido originalmente por Beck, Ward, Mendelson, Mock e Erbaugh (1961), com estimativa de fidedignidade que variou entre 0,79 e 0,90 a partir de seis amostras psiquiátricas (Beck & Steer, 1993). É um instrumento adaptado à população brasileira, mostrando-se particularmente adequado para uso com pacientes psiquiátricos e tem sido também amplamente usado na clínica e em pesquisa com pacientes não psiquiátricos e na população geral (Cunha, 2001).

Análise estatística

Os dados serão analisados usando os programas estatísticos SAS (SAS Institute Inc., Cary, NC, EUA) e SPSS (SPSS inc., Chicago, IL, EUA), considerando-se significativo $p<0,05$. Os resultados dos testes neuropsicológicos aplicados antes e depois do procedimento cirúrgico são de natureza qualitativa ou ordinal, sendo

atribuídos resultados numéricos e os resultados finais dos testes serão expressos em escores. Os dados serão expressos como mediana e intervalo de confiança de 95%.

5) REFERÊNCIAS BIBLIOGRÁFICAS

1. Unesco. O desafio da alfabetização global. Setor de Educação Divisão para a Coordenação das Prioridades das Nações Unidas na Educação. Organização das Nações Unidas para a Educação, a Ciência e a Cultura. Paris, 2009

2. Carvalho JAM, Garcia RA. O envelhecimento da população brasileira: um enfoque demográfico**.** Cad Saúde Pública. 2003; 19 (3): 109-18.

3. Gao L, Taha R, Gauvin D, Othmen LB, Wang Y, Blaise G. Postoperative cognitive dysfunction after cardiac surgery. Chest. 2005; 128 (5):3664-70

4. Kadoi Y, Goto F. Factors associated with postoperative cognitive dysfunction in patients undergoing cardiac surgery. Surg Today. 2006; 36 (12): 1053-7

5. Teixeira-Sousa V, Costa C, Costa A, Grangeia R, Reis C, Coelho R. Disfunção neurocognitiva após cirurgia valvar. Acta Med Port. 2008 Nov-Dec; 21(6): 610.

6. Selnes OA, Zeger SL: Coronary artery bypass grafting baseline cognitive assessment: essencial not optional. Ann Thorac Surg 2007;83:374-6

7. Ribeiro VM. Analfabetismo e alfabetismo funcional no Brasil. Boletim INAF. São Paulo: Instituto Paulo Montenegro, julho-agosto; 2006.

8. Andrade FHS. (org.) Neuropsicologia hoje. São Paulo: Artes Médicas, 2004.

9. Gazalle FK, Lima MS, Tavares BF, Hallal PC. Depressive symptoms and associated factors in an elderly population in southern Brazil. Rev. Saúde Pública. 2004 June ; 38(3): 365-371.

10. Inzitari M, Pozzi C, Ferrucci L, Chiarantini D, Rinaldi LA, Baccii M, Pini R, Masotti G, Marchionni N, Di Bari M. Subtle neurological abnormalities as risk factors for cognitive and functional decline, cerebrovascular events, and mortality in older community-dwelling adults. Arch Intern Med 2008, 168: 1270-6

11. Di Carlo A, Penna AM, Pantoni L, Basile AM, Bonacchi M, Pracucci G. Clinically relevant cognitive impairment after cardiac surgery: a 6-month follow-up study. J Neurol Sci. 2001;188(1-2):85-93

12. Monk TG, Weldon BC, Garvan CW, Dede DE, van der Aa MT, Heilman KM, Gravestein JS: Predictors of cognitive dysfunction after major noncardiac surgery. Anesthesiology 2008; 108: 18-30

13. Ishida K, Gohara T, Kawata R, Ohtake K, Morimota Y, Sakabe T. Are serum S100B proteins and neuron-specific enolase predictors of cerebral damage in cardiovascular surgery? J Cardiothorac Vasc Anesth 2003; 17:4-9

14. Kleindienst A, Bullock MR: A critical analysis of the roles of the neurotrophic

protein S100B in acute brain injury. J Neurotrauma 2006; 23: 1185-200

15. Boss GL, Soares LF, Oliveira Filho GR. Postoperative Cognitive Dysfunction: Prevalence and Associated Factors Ver Bras Anestesiol, 2005; 55:5 517-524

16. Petersen RC, Doody R, Kurz A, Mohs RC, Morris JC, Rabins PV, Ritchie K, Rossor M, Thal L, Winblad B. Current concepts in mild cognitive impairment. Arch Neurol. 2001 Dec;58(12):1985-92.

17. Elkins JS, O´Meara ES, Longstreth WT Jr, Manolio TA, Newman AB, Bhadelia RA, Jonhston SC. Education and the cognitive decline associated with MRI-defined brain infarct. Neurology 2006; 67:435-40.

18. Diniz, B.S.O. et al. Nível educacional e idade no desempenho. Rev. Psiq. Clín. 34 (1); 13-17, 2007

19. Souza Filho, Marcilio Lira de, Belo, Raquel, & Gouveia, Valdiney Veloso. (2006). Testes psicológicos: análise da produção científica brasileira no período 2000-2004. Psicologia: Ciência e Profissão, 26(3), 478-489

20. Laks, J. et al. Normas do Mini-Exame do Estado Mental para uma amostra de idosos com baixa escolaridade residentes na comunidade no Brasil. Cad. Saúde Pública, Rio de Janeiro, v. 23, n. 2, Feb. 2007 .

DISCIPLINA DE ANESTESIOLOGIA – FMUSP
INSTITUTO CENTRAL DO HCFMUSP

Protocolo de Pesquisa

DISFUNÇÃO COGNITIVA APÓS CIRURGIA SOB ANESTESIA GERAL

Coordenador da Pesquisa:
Maria José Carvalho Carmona

Pesquisador executante:
Lívia Stocco Sanches Valentin

Outros Pesquisadores envolvidos:
Kátia Osternack Pinto
Vinícius Fernando da Luz
Letícia Maria de Araújo de Souza
Ricardo Pietrobon
André Prato Schmidt
Jean Pierre Oses
Karen C. Nielsen
João Ricardo Nickenig Vissoci

São Paulo
2012

RESUMO

Introdução: Disfunção cognitiva pós-operatória (POCD) é um evento adverso multifatorial mais frequente em pessoas com idade superior a 60 anos ou doenças neurológicas e psiquiátricas. Este estudo objetiva avaliar primariamente o efeito da dexametasona sobre a incidência de POCD em idosos após cirurgia não cardíaca sob anestesia geral. Os objetivos secundários do estudo são a investigação da associação entre alterações cognitivas no pós-operatório e a profundidade da anestesia, a relação entre POCD e a presença do alelo ϵ4 da apolipoproteína e a relação entre os marcadores de lesão cerebral enolase específica do neurônio e S100β, e a ocorrência de POCD. **Métodos**: Serão recrutados até 300 pacientes para deste estudo prospectivo, randomizado, envolvendo a administração ou não de 8 mg de dexametasona IV antes da indução anestésica para anestesia geral profunda ou superficial de acordo com o índice bispectral. Os testes neuropsicológicos serão aplicados no pré-operatório e em 3, 7, 21, 90 e 180 dias após a cirurgia e comparados com os dados normativos. Enolase específica do neurônio e S100β serão avaliados antes e 12 horas após a indução da anestesia. A regressão linear com inferência baseada no método de equações de estimação generalizadas (GEE) será aplicado, seguido pelo teste post-hoc de Bonferroni, considerando $P < 0{,}05$ como significativo.

Palavras-chave: Testes neuropsicológicos; Dexametasona; Anestesia geral; Idosos.

1 INTRODUÇÃO

1 INTRODUÇÃO

A ocorrência de disfunção cognitiva no período pós-operatório (POCD) é evento relativamente frequente, principalmente em idosos e naqueles submetidos a cirurgias cardíacas, em que a incidência pode chegar a 83%[1-5]. Na maioria dos casos, a POCD é reversível, podendo ser permanente em menos de 1% dos casos. Embora mais frequente após anestesia geral, procedimentos sob anestesia regional também podem causar disfunção cognitiva transitória no pós-operatório imediato[4,6-9].

Os principais fatores identificados como predisponentes à POCD são a ocorrência de hipoxemia e hipotensão intraoperatória. Outros fatores incluem o uso de opioides, a dor, a depressão e a qualidade de vida no pré e pós-operatório[10-14]. Diversos outros fatores têm sido estudados como determinantes da ocorrência de POCD, como a profundidade anestésica[15] e a possível predisposição genética como a presença do alelo ɛ4 do gene da apolipoproteína[16,17].

A disfunção cognitiva após anestesia geral tem alta incidência imediatamente após o evento cirúrgico e uma diminuição da POCD até 2 anos[18]. A incidência de POCD está relacionada com o tipo de cirurgia, com a idade do paciente estudado, bem como com as doenças e o desempenho cognitivo do sujeito antes do evento cirúrgico[19].

O diagnóstico de POCD é feito por meio da aplicação de testes neuropsicológicos, que idealmente deveriam ser aplicados antes e após o procedimento cirúrgico. Muitos testes que são utilizados para a avaliação cognitiva, como o Mini Exame do Estado Mental (MEEM) não suprem a verdadeira necessidade do avaliador. Estes teste avaliam superficialmente o funcionamento mental e oferecem de forma ampla e generalizada como está o status cognitivo global do paciente no momento da aplicação. O diagnóstico das funções cognitivas deve ser minucioso, detalhando cada função avaliada. Para isto, o avaliador deve usar testes específicos para cada função cognitiva que se pretende investigar. Os testes neuropsicológicos específicos são instrumentos validados, confiáveis e padronizados que ajudam a elucidar e quantificar mudanças de comportamento resultantes de traumas cerebrais ou distúrbios do Sistema Nervoso Central (SNC). Os domínios cognitivos comumente avaliados incluem

linguagem, atenção/concentração, percepção viso-espacial e habilidade construtiva, sistema frontal e funções executivas, aprendizagem verbal/não verbal e memória. Mas nenhum teste neuropsicológico deve ser aplicado isoladamente a fim de diagnosticar qualquer disfunção. Para o diagnóstico de POCD, é necessário conhecer o histórico prévio do paciente, sintomas apresentados, exames médicos realizados e, se possível, ter uma avaliação neuropsicológica pré-operatória para comparação do desempenho do sujeito e para documentar se existiu melhoria ou declínio no funcionamento cognitivo do paciente[20-22].

O uso de testes padronizados com escores esperados para a população estudada é essencial para estabelecer um diagnóstico preciso no pré-operatório. Dados preliminares são importantes para subsequente comparação com dados pós-operatórios.

Não há consenso sobre condutas para diminuição da incidência de POCD. Por outro lado, sabe-se que o uso de corticoides como fármacos coadjuvantes durante a anestesia geral diminui a necessidade de opioides no intraoperatório e a incidência a náuseas, vômitos e dor no período pós-operatório, com aceleração da recuperação e redução do tempo de retorno às atividades diárias no pós-operatório[23-26]. Hipotetizou-se que o uso de dexametasona antes da indução anestésica pode ter efeito sobre a incidência de POCD no pós-operatório.

2 OBJETIVOS

2 OBJETIVOS

2.1 Objetivo primário

O objetivo primário deste estudo é avaliar o efeito da dexametasona sobre a incidência de disfunção cognitiva no pós-operatório de idosos submetidos a cirurgias sob a anestesia geral.

2.2 Objetivos secundários

Os objetivos secundários do estudo são:

- Investigar a associação entre alterações cognitivas no pós-operatório e a profundidade da anestesia;
- Investigar a relação entre POCD e a presença do alelo ϵ4 da apolipoproteína;
- Investigar a relação entre os marcadores de lesão cerebral enolase específica do neurônio e S100β, e a ocorrência de POCD.

3 REVISÃO DA LITERATURA

3 REVISÃO DA LITERATURA

A memória, concentração, linguagem, compreensão e integração social são funções neuropsicológicas que podem apresentar comprometimento após intervenções cirúrgicas, caracterizando a disfunção cognitiva pós-operatória (POCD), fato que pode ocorrer desde dias até semanas após uma cirurgia e, eventualmente, permanecer pelo resto da vida[14,27]. A maioria destas mudanças cognitivas é transitória, com resolução entre seis semanas e seis meses após o procedimento, o que acaba minimizando a atenção médica sobre a importância dos déficits cognitivos transitórios para a qualidade de vida do paciente[28,29]. Mesmo que transitório, este evento adverso perioperatório pode permanecer durante a recuperação do paciente e, quando de longa duração, comprometer o retorno do paciente às suas atividades[29-31].

Disfunção cognitiva pós-operatória (POCD) refere-se a uma deterioração da cognição ocorrida após um evento cirúrgico e anestésico[32,33]. Estudos recentes mostram as correlações e fatores de risco para esta disfunção, embora ainda haja muito a ser elucidado sobre incidência, etiologia, prevenção e tratamento[34,35].

Segundo Monk *et al.*, um número significativo de idosos submetidos a cirurgias com anestesia geral apresentam disfunção cognitiva no período pós-operatório, de maneira transitória e reversível, mas, em alguns casos, as disfunções se tornam crônicas[36,37]. A POCD envolve alterações de atenção, memória e alguns aspectos da função executiva. A função cognitiva tende a melhorar e a se estabilizar após alguns meses[38-41].

A POCD compreende desde transtornos sutis em quaisquer das áreas da cognição, até alterações incapacitantes como delírium e demência[42,43]. A etiologia da POCD é multifatorial e os fatores de risco não estão bem definidos[44,45].

Entretanto, a incidência de mortalidade relacionada à POCD permanece relativamente alta, principalmente no primeiro ano após procedimentos como cirurgias cardíacas[46,47], ortopédicas[48,49] e outros procedimentos de grande porte[50,51].

A incidência de POCD varia entre 5% e 10% na população geral e é, percentualmente, maior na população idosa, podendo chegar a até 26%[30]. Os avanços

tecnológicos e farmacológicos das últimas décadas contribuíram para o aumento da segurança e diminuição da mortalidade cirúrgica[52]. Diversos fatores vêm sendo estudados e relacionados à POCD, entre eles, o estado físico, histórico familiar, uso de drogas e álcool, qualidade de vida, escolaridade e, principalmente, a idade[53].

3.1 Fatores de risco para POCD

Para os fatores de risco associados à POCD, estão os abusos de substâncias tóxicas, álcool, doenças pré-existentes, distúrbios psiquiátricos e neurológicos. Estudos demonstram que o risco de desenvolvimento da POCD aumenta com a idade.

A principal etiologia das disfunções cognitivas pós-operatória permanece em aberto, reforçando a hipótese de um problema multifatorial[54].

Entre os fatores de risco envolvidos, estão os pré-operatórios (idade, escolaridade, doenças prévias), intraoperatórios (número de êmbolos, duração do procedimento, pressão arterial, temperatura) e pós-operatórios (temperatura, recuperação do procedimento)[55,56]. Além destes possíveis fatores de risco, alguns pesquisadores relataram influência genética sobre esse desfecho cirúrgico, merecendo especial destaque a presença do alelo ε4 da apolipoproteína -ε[57-59].

3.2 Fatores de risco pré-operatórios

A idade representa o fator de risco demográfico menos contestado para o declínio cognitivo pré-operatório, embora ainda com causa injustificada. Aterosclerose progressiva associada à doença cerebrovascular oculta e fatores intrinsecamente relacionados ao risco de embolização parecem ser a explicação mais aceitável para a POCD associada ao aumento da idade[6,60,61]. Idosos são predispostos à alteração vascular e da autorregulação do fluxo cerebral, apresentam, também, uma resposta anormal a fármacos e uma redução natural do nível cognitivo, que, associados a uma pequena diminuição cognitiva no pós-operatório, podem acarretar num significante impacto sobre a qualidade de vida[62].

Um estudo americano avaliou o fator idade após a alta hospitalar, em jovens (117 - 36%), pacientes de meia-idade (112-30, 4%) e idosos (138 - 41,4%), quanto à disfunção cognitiva após uma cirurgia, houve diferença entre todos os grupos de idade. Em três meses após a cirurgia, a POCD esteve presente em 16 (5,7%) dos jovens, 19 (5,6%) de meia-idade e 39 (12,7%) idosos. A prevalência de disfunção cognitiva foi significativamente maior em idosos em comparação aos outros grupos de pacientes[63].

Um estudo internacional sobre POCD em idosos (média de idade de 68 anos, com intervalo de 60-81 anos) que sofreram cirurgias não cardíacas demonstrou uma incidência de 26% de POCD na primeira semana após a cirurgia, e após 3 meses do evento cirúrgico esta disfunção permaneceu em 10% dos casos[64].

Outro fator pré-operatório que merece importante destaque na patogênese da POCD é o grau de escolaridade. Não se conhece plenamente o modo como um maior grau de escolaridade implicaria em maior reserva cognitiva, mas uma hipótese que explicaria essa associação está baseada no fato de que a escolaridade aumenta a densidade sináptica no neocórtex, aumentando a comunicação neuronal, e minimizando os sinais de comprometimento cognitivo e funcional[65,66]. História de comorbidades, como Diabete Mellito, hipertensão arterial sistêmica e insuficiência renal crônica, são fatores pré-operatórios que, igualmente à idade e escolaridade, estão relacionados a desfechos neurológicos prejudicados nos pós-operatórios[13,67,68].

3.3 Fatores de risco genéticos para POCD

Embora um grande número de fatores seja capaz de predizer o risco de POCD, os fatores genéticos contribuem cerca de 10% a 40% na ocorrência destes episódios[69]. Entre os possíveis polimorfismos genéticos candidatos a fatores de risco para a POCD, merece destaque especial a presença do alelo ε4 da apolipoproteína E (Apo-ε4). Esse polimorfismo é reconhecido e bem estabelecido como fator de risco para doença de Alzheimer e por desordens neurodegenerativas relacionadas. Na análise sobre a associação da presença de apo-ε4 com POCD, os resultados são controversos e justificam a necessidade de estudo dos demais fatores etiológicos relacionados às disfunções cognitivas[58,70].

A identificação de um suscetível genótipo a desenvolver POCD, bem como um biomarcador de plasma, permite uma intervenção precoce pré-cirúrgica prevenindo maiores agravos no comprometimento das funções cognitivas[71,72].

Estudos indicam o aumento da concentração sanguínea da enolase específica do neurônio (NSE) e proteína S-100β após cirurgias acarretando, mesmo que temporariamente em prejuízos cognitivos identificados em testes neuropsicológicos, assim como em marcadores bioquímicos. NSE parece ser o marcador bioquímico mais útil na predição de disfunção cognitiva após intervenções cirúrgicas. Estudos levantam a hipótese de que APO-ε4 pode ser responsável pela disfunção cognitiva pós-operatória como consequência de um aumento significativo da resposta inflamatória[59,73,74].

A APO-ε é uma constituinte das lipoproteínas VLDL e HDL, e desempenha um papel-chave no transporte e metabolismo de colesterol e triglicérides. Esta proteína, adicionalmente, está relacionada com a proteção neuronal. Três principais variantes da APO-ε são encontradas na população humana, sendo resultantes da mudança de um único aminoácido. Os alelos que codificam essas variantes são denominados APOE -ε2, APOE -ε3 e APOE -ε4.

A participação de 394 pacientes em um estudo para investigar a POCD evidenciou 24% da amostra com o alelo da APO-ε4. Os polimorfismos da APO-ε estavam em equilíbrio de *Hardy-Weinberg*, e com exatidão genotípica de 100% para APO-ε4. Entre os 350 (89%) pacientes que retornaram para a avaliação neuropsicológica em seis semanas, a POCD foi percebida em 56% com o alelo APO-ε4 comparado com 52,6% naqueles sem a APO-ε4. Neste mesmo estudo, não houve associação significativa entre o nível de biomarcadores e APO-ε4. Este estudo realizou uma primeira análise combinada da POCD após cirurgias não cardíacas, o genótipo APO-ε4 e biomarcadores plasmáticos de lesão cerebral, apesar de uma sólida amostra em tamanho, não foi possível encontrar uma associação entre declínio cognitivo e genótipo APO-ε4, acreditando que, da mesma forma, não poderiam encontrar associação entre o pós-operatório e NSE, e os níveis de S100β e o declínio cognitivo[75].

Abildstrom *et al.* estudaram a relação entre APO-ε4 e disfunção cognitiva não cardíaca em 972 pacientes, e não encontraram diferença na incidência de POCD (10,3% vs 9,9%) entre os pacientes com e sem alelo APO-ε4. Da mesma forma, não

houve associação do APO-ε4 com declínio cognitivo pós-operatório no estudo de 350 pacientes submetidos a cirurgias não cardíacas, bem como num estudo de 513 pacientes submetidos a cirurgias de revascularização[76].

Porém, estudos em pacientes submetidos a cirurgias cardíacas e alteração na cognição de sujeitos com o alelo APO-ε4 é mais evidente, com incidência de 20,6%[77]. Na população para cirurgias não cardíacas, os dados sobre a resposta inflamatória e os biomarcadores de plasma são raros.

A avaliação dos biomarcadores plasmáticos de lesão cerebral, NSE e S100β também produzem estudos com resultados pouco significativos. NSE é, geralmente, considerado um marcador de lesão cerebral, enquanto S100β é um marcador de astrocísticos gliais. Rasmussen *et al.* estudaram 65 pacientes idosos submetidos à cirurgia abdominal e não encontraram associação entre os níveis de NSE e S100β, e declínio cognitivo[73]. Um estudo com 120 pacientes submetidos a cirurgias não cardíacas encontrou uma associação de S100β, mas não de NSE com disfunção cognitiva após uma semana das cirurgias[78].

Em estudos sobre biomarcadores e POCD, os níveis dos biomarcadores voltam a se estabilizar, na maioria dos casos, após 18h, os índices de S100β e NSE aumentam ao longo do tempo pós-cirúrgico, sugerindo uma resposta inflamatória própria do quadro pós-cirúrgico[79].

Alguns estudos revelam que o tempo mais adequado para a coleta de sangue para NSE é 36 horas após a cirurgia, em que ocorre a concentração máxima da proteína S100β e NSE, evidenciando a existência de uma correlação negativa entre o aumento precoce em NSE e disfunção cognitiva[71].

Alguns trabalhos, citados por Rasmussen *et al.*, referem-se a um possível papel dos fatores genéticos implicados na patogênese das alterações cognitivas pós-cirúrgicas e a presença do alelo 4 da apolipoproteína-ε. Este alelo, reconhecidamente associado com a demência de Alzheimer, dará ao seu portador uma propensão genética, tornando o paciente mais vulnerável a qualquer tipo de lesão neuronal e consequente disfunção cognitiva, afetando, principalmente, as áreas de concentração, memória e linguagem[80].

3.4 Fatores de risco intraoperatórios

Os fatores de risco intraoperatórios, como a formação de êmbolos, cuja gênese estaria em ateromas da parede da aorta; agregados plaquetários, bolhas de ar oriundas do oxigenador e/ou das câmaras cardíacas, podem ser a causa primária da lesão encefálica ou do agravamento de lesões preexistentes. Estes se subdividem em micro e macroêmbolos, sendo os primeiros mais relevantes para surgimento da POCD[38]. A duração do procedimento cirúrgico também está relacionada à maior obstrução microvascular por êmbolos, o que sugere uma relação entre estes fatores e o desenvolvimento de disfunção cognitiva[81].

O valor da pressão arterial média durante o procedimento cirúrgico também é hipótese de disfunção cognitiva, sendo a hipotensão intraoperatória e, consequentemente, hipoperfusão cerebral uma causa potencial para comprometimento neurológico[82]. Outro fator que pode contribuir para a lesão neurológica é a resposta inflamatória, a hiperglicemia, principalmente nas cirurgias cardíacas, e o efeito da temperatura durante o procedimento cirúrgico[83].

A compreensão dos fatores relacionados ao tratamento cirúrgico dos idosos representa um desafio a uma equipe médica. Estes pacientes apresentam uma diminuição das reservas funcionais de diversos órgãos e sistemas e, como consequência, toleram muito pouco as exigências representadas pelo estresse anestésico cirúrgico. Alguns fatores relacionados são a diminuição da capacidade homeostática autonômica, funções imunológicas prejudicadas e redução da capacidade aeróbica[84,85].

Monk *et al.* evidenciaram, em pesquisa, a alta incidência de POCD (54,3%) em idosos submetidos à cirurgia não cardíaca grave em 6 semanas e 46,1% em 1 ano [51, 86]. Rasmussen *et al.* encontraram uma prevalência de 41,4% e 12,7% de POCD após uma semana e três meses, respectivamente[30].

3.4.1 Fármacos e anestésicos e profundidade da anestesia

Existe a necessidade de mais estudos específicos sobre técnicas anestésicas, e fármacos usados no transoperatório que possam comprovar realmente a influência

destes nas alterações cognitivas de curto, médio e longo prazo. Porém, com o conhecimento dos fatores de risco, é possível predizer aqueles indivíduos que terão uma maior chance de desenvolvimento de POCD e, dessa forma, proporcionar mecanismos protetores, reduzindo sequelas e evitando a instalação de lesões cerebrais irreversíveis.

A terapia neuroprotetora procura minimizar a ativação de vias tóxicas e incrementar os mecanismos endógenos de proteção[87-90]. Alguns medicamentos que aumentam o risco para a disfunção cognitiva são os anticolinérgicos, benzodiazepínicos, sedativos, antidepressivos e antiparkissonianos[91].

Estudos sobre os possíveis danos causados após intervenções cirúrgicas em anestesia geral se fazem necessários, principalmente quando se planeja uma cirurgia e o tipo de anestesia. As alterações da cognição acarretam em perdas de ordem psicoemocionais, sociais e econômicas. A anestesia não é o fator primordial para a POCD, embora poucos, existem estudos em andamento buscando evidências sobre todos os fatores de risco envolvidos em um procedimento cirúrgico[47,92,93].

A incidência da disfunção cognitiva em idosos após cirurgias não cardíacas sob anestesia geral e regional foi investigada em um estudo com 188 pacientes com idade média de 60 anos[7,94,95]. A hipótese de que a incidência de POCD seria menor para as anestesias regionais foi confirmada[87]. A função cognitiva foi avaliada por meio de quatro testes neuropsicológicos realizados no pré e pós-operatórios. Após sete dias da cirurgia, a POCD foi encontrada em 37/188 pacientes (19,7% [14,3-26,1%]) após anestesia geral e, em 22/176 (12,5% [8,0-18,3%]), após anestesia regional. Depois de três meses, a POCD estava presente em 25/175 pacientes (14,3%, % [9,5-20,4]) após anestesia geral *versus* 23/165 (13,9%, [9,0-20,2%]) após anestesia regional. A incidência de POCD após uma semana foi significativamente maior após anestesia geral[88].

Os corticosteroides, de modo geral, e, em particular, os glicocorticoides, constituem um grupo de drogas muito usadas em medicina clínica. Em virtude de seu amplo espectro ativo e por serem os mais potentes anti-inflamatórios existentes, encontram uso praticamente em todas as especialidades[23-26,96-99].

A administração de anti-inflamatórios antes de procedimentos cirúrgicos atenua dores pós-operatórias, fadiga, náuseas e vômitos, acelerando a recuperação do

paciente, oferecendo oportunidade do retorno às atividades diárias em tempo reduzido. A dexametasona é um medicamento pertencente à classe dos corticosteroides, atuando no controle da velocidade de sínteses de proteínas. Por sua ação anti-inflamatória e imunossupressora, pode prevenir ou suprimir processos inflamatórios de várias naturezas e, se administrada no período pré-operatório, fará com que o paciente tenha benefícios em seu período de recuperação, amenizando os sintomas desagradáveis durante o período convalescente[23-26,96-99].

O diagnóstico para a POCD requer uma avaliação neuropsicológica minuciosa antes e após as intervenções cirúrgicas. Esta avaliação possibilita investigar quais fatores de risco desencadeiam este comprometimento e quais funções corticais superiores podem ser afetadas. Embora, como já mencionado, ainda haja muito a ser elucidado sobre incidência, etiologia, prevenção e tratamento, amenizar os danos causados por estes eventos oferece a integridade física, psíquica e emocional do paciente, melhorando sua qualidade de vida.

Estudos sobre drogas anestésicas mostram que anestésicos influenciam a cognição, pelo menos temporariamente. Partindo-se do pressuposto de que o paciente está inconsciente, sem ter a noção do que está acontecendo com ele durante o processo cirúrgico e portanto amnésicos, o cérebro é um órgão alvo para possíveis mudanças neurológicas e muitas destas mudanças são caracterizadas por declínio cognitivo e confusão mental após a anestesia.

Há uma crescente evidência de que por período longo ou até mesmo permanente ocorrem mudanças neurológicas e neuronais decorrentes da administração do anestésico[100].

Um estudo piloto relatou que o monitoramento com o bispectral (BIS) durante o período intra-operatório pode reduzir a deterioração cognitiva após cirurgia não cardíaca grave em pessoas idosas. O estudo indica que uma intervenção pragmática para a manutenção da oxigenação cerebral com monitoramento do BIS para o controle da profundidade anestésica otimizou os resultados dos testes cognitivos pós-operatórios identificando POCD leve, comprometimento cognitivo global e deficiências de atenção em uma semana pós-cirurgia, bem menores no grupo estudo. A intervenção também conferiu benefícios sustentados na redução POCD leve e melhora na atenção, o desempenho da função executiva e para a cognição global em

52 semanas após a cirurgia. No entanto, o poder estatístico utilizado no estudo foi limitado, especialmente para detectar diferenças na POCD grave, mas mostrou que as frequências foram menores no grupo de intervenção e o padrão geral de resultados indicaram benefício claro no grupo de intervenção. Estes resultados são consistentes com a literatura existente, embora poucos estudos investigaram especificamente o efeito do monitoramento dos valores do BIS para reduzir a incidência de POCD. Estudos com animais idosos evidenciam que anestésicos gerais podem ser neurotóxicos para o desenvolvimeto e envelhecimento do cérebro. No entanto, estudos recentes concluem que seria prematuro mudar a prática clínica, visto que o efeito no ser humano ainda não está completamente estudado e que os anestésicos são bem conhecidos como neuroprotetores e podem mitigar a resposta inflamatória do SNC para o trauma operatório[101]. A hipótese seria de que uma faixa ideal de profundidade anestésica pode maximizar a neuroproteção e o limite de neurotoxidade. Neste mesmo estudo piloto, os pacientes que receberam a intervenção (monitoramento com BIS alvo 40-60 ± 5) apresentaram melhores resultados nos testes cognitivos e S100B, um marcador de lesão cerebral. Embora, o estudo reconhece que a força das correlações é modesta, este fornece evidências de que a monitorização BIS foi um componente-chave da intervenção[102-104].

4 CASUÍSTICA E MÉTODOS

4 CASUÍSTICA E MÉTODOS

4.1 Projeto de pesquisa

O protocolo do estudo foi aprovado pela Comissão de Ética Médica do Hospital das Clínicas da Faculdade de Medicina da Universidade de São Paulo (CAPPesq – Projeto de Pesquisa nº: 998/06), registrado na plataforma do www.clinicaltrials.gov (Identifier: NCT01332812) e recebeu auxílio à pesquisa do Fundo de Amparo à Pesquisa do Estado de São Paulo - Processo FAPESP nº 09/54233-0.

4.2 Critérios de seleção dos pacientes

Critérios de inclusão: pacientes acima de 60 anos, submetidos a cirurgias de médio porte sob anestesia geral com duração prevista de até 4 horas e permanência hospitalar prevista de até 72 horas.

Critérios de exclusão: história pregressa de doença cerebral ou demência, outras doenças psiquiátricas que afetem a cognição, falta de domínio da Língua Portuguesa, uso de corticoide ou de opioide no pré-operatório.

4.3 Termo de consentimento livre e esclarecido

Após definição da indicação cirúrgica, e avaliação dos critérios de inclusão e exclusão, os pacientes serão convidados a participar da pesquisa. Após esclarecimentos em relação aos objetivos gerais do estudo, o paciente (ou seu responsável legal) assinou o termo de consentimento livre e esclarecido (TCLE) em duas vias, uma destinada ao prontuário do paciente da pesquisa e a outra entregue ao sujeito de pesquisa (Apêndice B).

4.4 Casuística

O estudo recrutará até 300 pacientes pacientes com programação cirúrgica para procedimento de médio porte sob anestesia geral. Os pacientes serão submetidos ao Miniexame do Estado Mental (MEEM), com exclusão daqueles que não atingirem a pontuação mínima exigida (>18 a 23 pontos) por este teste de rastreio segundo o grau de instrução do sujeito.

Miniexame do estado mental – (MEEM): O MEEM avalia o raciocínio, orientação espaço-temporal, memória e escolaridade. O ponto de corte deste instrumento, para a população brasileira, é considerado para dois tipos de escolaridade – até 4 anos de estudo formal acadêmico e com mais de 4 anos de escolaridade; com ponto de corte de 18 para os menos escolarizados e com mais de 23 pontos para indivíduos com maior nível de escolaridade[105, 106].

Pacientes randomizados para o estudo serão mantidos no estudo e analisados pelo método intenção de tratamento (ITT). Pacientes que não puderem comparecer às avaliações presenciais (3ª e 5ª fases) serão avaliados com o instrumento específico de avaliação por via telefônica, *Telephone Interview Cognitive Status* (TICS).

O recrutamento de pacientes iniciou-se em fevereiro de 2010.

4.5 Métodos

O estudo será realizado nos períodos pré, intra e pós-operatórios. As avaliações neuropsicológicas serão realizadas nos períodos pré e pós-operatório.

Os critérios para a definição de POCD serão aqueles adotados pelo ISPOCD, e amplamente divulgados e considerados em outras pesquisas internacionais. Os critérios definidos para POCD partirão da pontuação do próprio paciente numa avaliação pré-cirúrgica, comparada com seus próprios resultados em avaliações pós-operatórias em seguimento durante as pesquisas. Desta forma, POCD será determinada pela dedução do escore pré-operatório ao escore pós-operatório, dando um valor individual para cada tarefa. A média obtida pelo grupo controle, calculada da mesma forma que para os sujeitos do estudo, será subtraída pela média dos pacientes. Este

escore é, então, dividido pelo desvio padrão para a mudança nos resultados, controlado pelo efeito aprendizado e variações normais ao redor do tempo. Assim, POCD será definida por 2 escores de desvio padrão já estabelecidos pelo z-score de cada teste. Estes critérios são encontrados em tabelas normativas.

4.5.1 Período pré-operatório

A avaliação pré-operatória incluiu um questionário sociodemográfico com informações relevantes para compor o perfil da amostra, do teste MEEM e testes neuropsicológicos. O questionário sociodemográfico será preenchido pelo aplicador, neuropsicólogo envolvido com a pesquisa, com as respostas oferecidas pelo paciente. Os testes neuropsicológicos e o MEEM serão realizados pelo paciente através de respostas orais quando solicitadas e por sua vez anotadas pelo pesquisador na folha de respostas do teste ou pela produção gráfica ou escrita, dependendo do constructo, pelo próprio paciente na folha de resposta do teste.

Informações Sociodemográficas

Serão coletadas as informações sobre idade, gênero, escolaridade, estado civil, ocupação, e demais informações relevantes para compor o perfil sociodemográfico da amostra, bem como a compatibilidade com os critérios de exclusão e inclusão no estudo.

Versão Brasileira do Questionário de Qualidade de Vida SF-36

O SF-36 será utilizado para investigar condições relacionadas à saúde, atividades de vida diária, produtividade, problemas emocionais, relacionamentos, motivação e outras condições que traçam o perfil da qualidade de vida a partir da percepção do próprio sujeito.

Inventário de Depressão de Beck – BDI

O Inventário de Depressão de Beck (BDI) consta de 21 questões que exploram sintomas de depressão numa escala de 0 a 4, na qual zero corresponde à ausência de

sintomas e quatro corresponde ao nível máximo da sintomatologia. Um total de 18 pontos tem sido considerado indicativo da presença de sintomatologia depressiva. O inventário foi desenvolvido, originariamente, por Beck, Ward, Mendelson, Mock e Erbaugh (1961), com uma estimativa de fidedignidade que variou entre 0,79 e 0,90 a partir de seis amostras psiquiátricas.[107, 108] O instrumento utilizado foi adaptado à população brasileira e particularmente adequado para uso com pacientes psiquiátricos, e tem sido, também, amplamente usado na clínica e em pesquisa com pacientes não psiquiátricos e na população geral.

Telephone Interview for Cognitive Status – (TICS)

É um teste padronizado para avaliação do funcionamento neuropsicológico, é utilizado quando a avaliação do cognitivo global é impraticável ou ineficiente na forma presencial, por exemplo, em pesquisas epidemiológicas de grandes populações ou com pacientes que estão incapacitados de comparecerem ao retorno clínico. Também pode ser aplicável em pacientes com incapacidade para ler ou escrever, exigindo apenas capacidade de compreensão verbal. O teste consiste em um roteiro de entrevista com onze itens que avaliam as habilidades de orientação espacial e temporal, controle mental, memória, informação geral, linguagem e cálculos, conforme detalhado no apêndice.

Visual Verbal Learning – (VLT)

Apresenta-se uma lista de 15 palavras para ser memorizada e recordada em três tentativas sucessivas (VLT/A-B-C), com evocação tardia após 15 a 25 minutos (VLT-D). Avalia-se o número de palavras recordadas e o número de erros cometidos para cada apresentação. O VLT avalia as modalidades da memória – imediata, consolidada e de longo prazo.

Symbol Digit Modilities Test – (SDMT)

Este teste afere a memória de curto prazo, habilidade viso-espacial e atenção. Também avalia a capacidade do indivíduo de organizar, planejar e buscar estratégias para a realização da tarefa em menor tempo e com maior quantidade de símbolos feitos em 180 segundos, habilidades da função executiva. O indivíduo tem que reproduzir os

símbolos exemplificados em uma tarjeta nos espaços abaixo do número correspondente.

Trail Making Test - (TMT)

Este teste é composto por duas partes. Na parte A, o sujeito deve traçar linhas conectando, consecutivamente, círculos numerados. Na parte B, o sujeito deve traçar linhas conectando alternadamente círculos com letras e números em uma sequência. O teste envolve, além da atenção seletiva e alternada, o rastreio visual complexo e destreza motora (Parte A) e processos executivos (Parte B). Entre os processos executivos, a capacidade inibitória e a alternância cognitiva parecem ser aquelas mais exigidas na execução da tarefa. São avaliados o tempo despendido e o número de erros cometidos em cada parte.

Stroop Card Word and Color Test - (SCWCT)

Consiste na apresentação de três lâminas de papel ao sujeito. Na primeira lâmina, o sujeito deve verbalizar os nomes das cores impressas com tinta preta. Na segunda, a verbalização é feita da cor que estão preenchidos retângulos, na mesma disposição das palavras da lâmina anterior. A terceira consiste em verbalizar as cores impressas em detrimento da palavra escrita. Avalia atenção seletiva, capacidade inibitória e flexibilidade mental. São avaliados o tempo despendido e o número de erros em cada cartão.

4.5.2 Período intraoperatório

Após admissão na sala de operações, os pacientes serão monitorados com oximetria de pulso, eletrocardiografia contínua, pressão arterial não invasiva e índice bispectral, seguido de punção venosa periférica com cateter de calibre 18G.

O índice bispectral é uma escala numérica que decresce de 100 a 0, na qual o 100 ou próximo dele representa o paciente acordado e o 0 representa um eletroencefalograma com completa supressão cortical, ou sem atividade cerebral. Este monitor realiza análise de dados eletroencefalográficos captados através de eletrodos

instalados na região frontal do paciente. A escala próxima de 100 mostra que o paciente não sofreu a influência de nenhum fármaco hipnótico, à medida que aumenta o nível de sedação a escala é reduzida. Um índice de 70 na escala é considerado como sedação leve e, abaixo de 60, são considerados níveis profundos de sedação. Será utilizado um índice bispectral entre 45 a 55 para manter a anestesia superficial, e entre 35 e 45 para a manutenção da anestesia profunda.

Antes da indução anestésica, serão colhidas duas amostras de sangue para determinação do perfil genotípico da Apoɛ, e dosagem de S100β e NSE. As amostras para determinação do perfil genotípico (APOɛ4) serão encaminhadas para processamento no Laboratório de Biologia Molecular do InCor.

Os pacientes serão distribuídos aleatoriamente em dois grupos e posteriormente redistribuídos em 4 subgrupos, compreendendo um total de quatro grupos. Antes da indução da anestesia os pacientes serão randomizados para receber ou não 8 mg de dexametasona e avaliados sob análise intenção de tratar (ITT). De acordo com o índice Bispectral (BIS), os pacientes serão alocados em um subgrupo que recebeu anestesia superficial (BIS entre valores 46-55) ou em um subgrupo que recebeu anestesia profunda (BIS entre valores 35-45).

O primeiro grupo de pacientes recebeu a dose de dexametasona e anestesia profunda; o segundo grupo também recebeu a dose de dexametasona e anestesia superficial. Os terceiro e quarto grupos não receberam a dose de dexametasona; sendo que para o terceiro grupo a profundidade anestésica será monitorada pelo BIS entre os valores 46 a 55 considerados valores para a anestesia profunda e o quarto grupo recebeu a anestesia superficial, controlada pelo BIS entre os valores 35 a 45. A randomização dos grupos será realizada em blocos de 100 casos, utilizando-se o site da Internet randomizer.com e os resultados inseridos em envelope opaco selado, a ser aberto na sala cirúrgica, imediatamente antes da indução anestésica.

A anestesia geral será induzida com Propofol (2-3 mg/kg), fentanil (2-3 µg/kg) e cisatracúrio (0,10 mg/kg). A manutenção anestésica subsequente será realizada com propofol em infusão alvo-controlada conforme valor do índice bispectral determinado pelo grupo ao qual o paciente pertencia, remifentanil (0,10 a 1,0 µg/kg/min) e doses suplementares de cisatracúrio a critério do anestesiologista. A ventilação será controlada mecanicamente para manter a pressão de dióxido de carbono próximo a 35

mmHg, utilizando-se FiO_2 de 60%. Solução de ringer simples será administrada durante a cirurgia para reposição volêmica.

Ao término do procedimento anestésico-cirúrgico, os pacientes serão encaminhados à Unidade de Recuperação Pós-anestésica e, posteriormente, ao leito de origem. A analgesia pós-operatória será realizada com Dipirona na dose de 30mg/kg ao final do procedimento cirúrgico e com Tramadol na dose de 50-100mg no período pós-operatório, se necessário.

Os neuropsicólogos e técnicos de laboratório envolvidos no estudo não tiveram conhecimento do grupo ao qual cada paciente pertencia antes da finalização da análise dos dados do estudo.

4.5.3 Período pós-operatório

A coleta de amostra sanguínea para dosagem de S100β e NSE será repetida após 12 horas do final da cirurgia. As amostras para dosagem de S100β e NSE serão centrifugadas, e o soro armazenado a -70ºC para posterior análise no Laboratório de Neurociências da UFRGS.

Devido à condição social da maioria dos pacientes incluída na pesquisa (pacientes do Hospital das Clínicas da Faculdade de Medicina da USP), considerou-se, desde o início do estudo, que dificuldades de comunicação com os sujeitos (principalmente alterações de números de telefones) poderiam dificultar a coleta dos dados, especialmente no 3º e 6º mês pós-operatório.

4.5.3.1 Análise dos biomarcadores

Os níveis séricos de proteína S100β e NSE serão obtidos em 3 ml de amostras de sangue venoso coletado dos pacientes em dois momentos:

- 1ª amostra: No dia anterior à cirurgia;
- 2ª amostra: 12 horas após a cirurgia.

Para a determinação destes momentos de coleta, serão utilizadas como referência publicações que investigaram a meia-vida destes marcadores de lesão neuronal.[95]

Os níveis séricos de proteína S100β e NSE serão obtidos a partir de amostras de sangue venoso que serão colhidas antes da indução anestésica e 12 horas após a cirurgia. As amostras serão colocadas em tubos secos e centrifugados; o soro será removido e armazenado a -80 ºC até o momento da análise. A proteína S100β será medida por um ensaio imunológico absorvente (ELISA) kit ligado a enzima S100β disponível comercialmente (DiaSorin, Itália) em um ensaio monoclonal quantitativo de microplacas em que o último anticorpo adicionado ao sistema de reação é marcado com peroxidase. Após a adição de um substrato da peroxidase, a reação produz um produto de cor final lida num espectrofotómetro. Níveis S100β serão expressos em microgramas por mililitro. A enolase específica do neurônio (NSE) será medida utilizando um ensaio de electroquimioluminescência. Consiste num ensaio duplo-sanduíche que utiliza um anticorpo anti-NSE, marcado com ruténio, o qual é a molécula luminescente. A quantificação às reações será realizada com Elecsys-2010 (Roche Diagnostics Corporation, EUA). Os níveis de NSE serão expressos em microgramas por mililitro[109-113].

APOε4 será colhida em uma amostra de sangue preoperativamente e serão colocadas em tubos secos, e o plasma centrifugado será removido e armazenado a -70 °C até o momento da análise. Todos os pesquisadores, inclusive os que colaboraram com as análises laboratoriais, permaneceram cegados durante aa genotipagem. O DNA será preparado para determinar a frequência do alelo da APOε4.

4.5.3.2 Análise dos testes neuropsicológicos

As avaliações neuropsicológicas pós-operatórias serão repetidas no 3º, 7º, 21º, 90º e 180º dias pós-operatórios, sendo:

- 3º P.O. – A avaliação cognitiva será realizada por via telefônica pelo teste – TICS;

- 7º P.O. – A avaliação por via telefônica se repetiu pela aplicação do instrumento TICS, com o objetivo de verificar sutil melhora cognitiva na fase de recuperação pós-operatória;
- 21º P.O. – Esta fase avaliativa será realizada, preferencialmente, de forma presencial com agendamento prévio. Os testes serão aplicados em condição adequada proporcionando *set* terapêutico em sala ambulatorial do Hospital das Clínicas da Faculdade de Medicina da Universidade de São Paulo. Exceto o teste MEEM, que será utilizado como *screening* para a inclusão do sujeito na amostra, serão aplicados, incluindo o TICS, todos os testes neuropsicológicos, inventários de depressão e qualidade de vida já aplicados na fase pré-operatória. Em todas as fases presenciais, os testes para aferição da memória tinham as listas de palavras diversificadas com mudanças em suas categorias léxico-semânticas;
- 90º P.O. – Será realizado contato telefônico para a aplicação do instrumento TICS. Nesta fase, conseguiu-se observar, qualitativamente, o *status* cognitivo do sujeito avaliado;
- 180º P.O. – Repetiu-se a aplicação da bateria neuropsicológica e testes psicológicos, encerrando a participação do paciente no protocolo de pesquisa.

4.5.4 Normatização e metodologia para análise dos resultados

A aplicação de avaliações neuropsicológicas deve ser considerada como base para a definição e diagnóstico da POCD. A bateria neuropsicológica utilizada neste estudo, que afere as funções cognitivas superiores de memória, executiva, atenção e linguagem, seguiu o protocolo sugerido pelo Estudo Internacional de Disfunção Cognitiva Pós-Operatória (*International Study of Postoperative Cognitive Disfunction – ISPOCD*)[64] com a finalidade de detectar possíveis e sutis disfunções neuropsicológicas em pacientes idosos após cirurgia com anestesia geral.[63,64,114]

Os pacientes que atingiram a pontuação mínima no MEEM serão inclusos no estudo e avaliados por uma bateria ampliada de testes neuropsicológicos (BTN) para avaliação das funções cognitivas de memória, atenção, raciocínio, linguagem e

executiva, além de mensurar os aspectos emocionais relacionados à depressão e qualidade de vida. Todos os testes neuropsicológicos serão administrados verbalmente ou graficamente, cronometrados e corrigidos individualmente e comparados com resultados em escalas de acordo com a idade, o gênero e a escolaridade de uma amostra padronizada em população geral, tendo como referência as escalas de neuropsicologia do compêndio de testes Neuropsicológicos de Strauss E, Sherman EMS e Spreen O.[102] Em relação ao tempo para a execução da tarefa, é esperado que o indivíduo não exceda um tempo maior que três minutos.

Memória: A memória será avaliada imediatamente após a evocação da linguagem e depois de vinte minutos.

Função Executiva: A avaliação da função executiva para a busca de estratégias, planejamento e organização do conteúdo armazenado, assim como a linguagem, ocorreu por observação clínica do discurso do paciente, eloquência, fluência e curso do pensamento, e pela performance do sujeito durante a realização dos testes.

Atenção: O tônus e manutenção da atenção serão avaliados durante a aplicação da BTN e por instrumentos padrão.

POCD será estabelecida por dois critérios: Dois escores de desvio padrão já definidos pelo z-score de cada teste e/ou a partir dos resultados prévios de testes realizados pelo próprio sujeito antes da cirurgia, quando testado.[103] Os escores esperados são encontrados em tabelas normativas para grande parte dos testes utilizados pelo ISPOCD e definidos a partir da escolaridade ou idade.

Serão observados pacientes submetidos à cirurgia com anestesia geral superficial ou profunda. Esses pacientes serão randomizados em 4 grupos nomeados:

GRUPO- ANESTESIA PROFUNDA: este grupo não receberá nenhum fármaco adicional à técnica anestésica preconizada; recebendo o tratamento convencional à necessidade cirúrgica. A profundidade anestésica para este grupo será

controlada pelo médico anestesiologista pelo índice bispectral (BIS) entre 35 a 45 para manter a anestesia profunda.

GRUPO- ANESTESIA SUPERFICIAL: este grupo também não receberá nenhum fármaco adicional à técnica anestésica preconizada; recebendo apenas o tratamento convencional à necessidade cirúrgica. A profundidade anestésica para este grupo será controlada pelo médico anestesiologista pelo índice bispectral (BIS) entre 45 a 55 para manter a anestesia superficial.

GRUPO- ANESTESIA PROFUNDA + DEXAMETASONA: este grupo receberá 8 mg de dexametasona por via venosa antes do início da indução anestésica e a técnica anestésica preconizada será mantida. A profundidade anestésica para este grupo será controlada pelo médico anestesiologista pelo índice bispectral (BIS) entre 35 a 45 para manter a anestesia profunda.

GRUPO- ANESTESIA SUPERFICIAL + DEXAMETASONA: este grupo também receberá 8 mg de dexametasona por via venosa antes do início da indução anestésica e a técnica anestésica preconizada será mantida tal qual os demais grupos. A profundidade anestésica para este grupo será controlada pelo médico anestesiologista pelo índice bispectral (BIS) entre 45 a 55 para manter a anestesia profunda.

Os pacientes serão submetidos a uma bateria de testes neuropsicológicos ao longo dos 6 meses após a cirurgia, sendo um dos testes, telefônico, aplicado no pré-operatório, 3º dia, 7º dia, 21º dia, 90º dia e 180º dia pós-operatório, e os demais testes realizados presencialmente no pré-operatório, 21 dias e 180 dias após a cirurgia. Serão analisados os resultados dos testes ao longo dos 6 meses após a cirurgia e a diferença destes resultados entre os grupos.

4.5.5 Cálculo amostral e análise estatística

O cálculo do tamanho da amostra foi revisto, com previsão de recrutamento dos 300 pacientes propostos no projeto inicial. Com base em uma incidência de disfunção cognitiva pós-operatória de 40% para pacientes idosos e tendo uma redução de 30%

na POCD (Cognição Geral) para o grupo que recebeu a dose de dexametasona considerada clinicamente relevante, com confiança de 95% e poder estatístico desejado de 80%, estima-se que 30 pacientes em cada grupo sejam necessários para compor a amostra deste estudo.

Os resultados dos testes neuropsicológicos serão descritos de acordo com os grupos e horários de avaliação, pela média e desvio padrão, e comparadas entre os grupos usando Equação de Estimação Generalizada (EEG).[96]

Os resultados dos testes neuropsicológicos serão comparados com os resultados de tabelas normativas pareados por idade, gênero e educação formal, usando como parâmetro o *Z-score*. As abordagens de máxima verossimilhança serão utilizadas no caso de falta de dados, que não ultrapassou 12% do total de dados coletados, por uma análise de modelo misto linear (*Mixed Model*) para a imputação dos dados faltantes, aumentando a acurácia estatística. A análise dos resultados de cada teste neuropsicológico será comparativa, de acordo com a idade, nível de educação e gênero por tabelas padronizadas de cada instrumento.

Para os modelos que apresentaram significância estatística, a análise será seguida de comparações múltiplas de Bonferroni para saber entre quais grupos ou momentos ocorrem as diferenças nas escalas.[97]

Para comparação dos biomarcadores entre os grupos e momentos de avaliação, serão utilizadas EEG com matriz de correlações autorregressiva de ordem 1 entre os momentos, seguidas de comparações múltiplas de Bonferroni para saber entre quais grupos ou momentos ocorrem as diferenças nos biomarcadores.[97,98]

Serão calculadas as variações dos biomarcadores (pré e pós-anestesia) e dos testes neuropsicológicos em 21 dias e 180 dias (21 dias; pré e 180 dias e pré, respectivamente) e serão calculadas as correlações de Pearson entre as variações dos biomarcadores e as variações nos testes neuropsicológicos em separado, para os grupos e no total de pacientes, para avaliar a existência de correlação entre as variações dos biomarcadores com a variação dos testes neuropsicológicos.

A alteração de cognição nos testes neuropsicológicos serão descritas segundo momentos de aplicação e presença do APOε4 com uso de frequências absolutas e relativas e comparados entre presença do APOε4 e momentos com uso de equações de estimação generalizadas com matriz de correlações componente simétrica entre os

momentos, com distribuição marginal Binomial e função de ligação logito. Para os modelos que apresentaram significância estatística a análise será seguida de comparações múltiplas de Bonferroni para saber entre quais alelos ou momentos ocorrem as diferenças na probabilidade de disfunção.[98] Todos os testes serão realizados no nível de significância de 5%. Os cálculos estatísticos serão realizados utilizando SPSS12 e GraphPad Prism versão 6.00 para Mac (GraphPad Software, La Jolla, Califórnia EUA www.graphpad.com).

8 REFERÊNCIAS

8 REFERÊNCIAS

1 Rasmussen LS, Johnson T, Kuipers HM, Kristensen D, Sierm VD, Vila P. Does Anaesthesia cause postoperative cognitive dysfunction? A randomised study of regional versus general anaesthesia in 438 elderly patients. *Acta Anaesthesiol Scand.* 2003;47(3):260-6.

2 Ancelin ML, de Roquefeuil G, Ledésert B, Bonnel F, Cheminal JC, Ritchie K. Exposure to anaesthetic agents, cognitive functioning and depressive symptomatology in the elderly. *Br J Psychiatry.* 2001;178:360-6.

3 Bekker AY, Weeks EJ. Cognitive function after anaesthesia in the elderly. *Best Pract Res Clin Anaesthesiol.* 2003;17(2):259-72

4 Wu CL, Hsu W, Richman JM, Raja SN. Postoperative cognitive function as an outcome of regional anesthesia and analgesia. *Reg Anesth Pain Med.* 2004;29(3):257-68.

5 Cohendy R, Brougere A, Cuvillon P. Anaesthesia in the older patient. *Curr Opin Clin Nutr Metab Care.* 2005;8(1):17-21.

6 Halaszynski T. Pain management in the elderly and cognitively imparired patient: the role of regional anesthesia and analgesia. *Curr Opin Anaesthesiol.* 2009;22(5):594-9.

7 Mason SE, Noel-Storr A, Ritchie CW. The impact of general and regional anesthesia on the incidence of pos-operative cognitive dysfunction and pos-operative delirium: a systematic review with meta-analysis. *J Alzheimers Dis.* 2010;22(Suppl 3):67-79.

8 Lelis RGB, Krieger JE, Pereira AC, Schmidt AP, Carmona MJ, Oliveira SA, Auler JOC Jr. Apolipoprotein E4 genotype increases the risk of postoperative cognitive dysfunction in patients undergoing coronary artery bypass graft surgery. *J Cardiovasc Surg.* 2006;47(4):451-6.

9 McDonagh DL, Mathew JP, White WD, Phillips-Bute B, Laskowitz DT, Podgoreanu MV, Newman MF. Cognitive function after major noncardiac surgery, apolipoprotein e4 genotype, and biomarrkers of brain injury. *Anesthesiology.* 2010;112(4):852-9.

10 Andrew MJ, Baker RA, Kneebone AC, Knight JL. Mood state as a predicitor of neuropsychological deficts following cardiac surgery. *J Psychosom Res.* 2000;48(6):537-46.

11 Kain ZN, Caldwell-Andrews AA, Maranets I, McClain B, Gaal D, Mayes LC, Feng R, Zhang H. Preoperative anxiety and emergence delirium and postoperative maladaptive behaviors. *Anesth Analg.* 2004;99(6):1648-54.

12 Zipfel S, Schneider A,Wild B, Lowe B, Junger J, Haass M, Sack F, Bergmann G, Herzog W. *Effect of* depressive symptoms on survival after in heart transplant. *Psychosom Med.* 2002;64(5):740-7.

13 Rasmussen LS, O'Brien JT, Silverstein JH, Johnson TW, Siersma VD, Canet J, Jolles J, Hanning CD, Kuipers HM, Abildstrom H, Papaioannou A, Raeder J, Yli-Hankala A, Sneyd JR, Munoz L, Moller JT; ISPOCD2 Investigators. Is peri-operative cortisol secretion related to post- operative cognitive dysfuntion? *Acta Anaesthesiol Scand.* 2005;49(9):1225-31.

14 Benoit AG, Campbell BI, Tanner JR, Staley JD, Wallbridge HR, Biehi DR, Bradley BD, Louridas G, Gusman RP, Rebecca A. Risk factors and prevalence of perioperative cognitive dysfunction in abdominal aneurysm patients. *J Vasc Surg.* 2005;42(5):884-90.

15 Tung A, Herrera S, Fornal CA, Jacobs BL. The effect of prolonged anesthesia with isoflurane, propofol, dexmedetomidine, or ketamine on neural cell proliferation in the adult rat. *Anesth Analg.* 2008;106(6):1772-7.

16. Risacher SL, Kim S, Shen L, Nho K, Foroud T, Green RC, Petersen RC, Jack CR Jr, Aisen PS, Koeppe RA, Jagust WJ, Shaw LM, Trojanowski JQ, Weiner MW, Saykin AJ; Alzheimer's Disease Neuroimaging Initiative (ADNI).The role of apolipoprotein E (APOE) genotype in early mild cognitive impairment (E-MCI). *Front Aging Neurosci.* 2013;5:11.

17. Lopez MF, Krastins B, Ning M. The role of apolipoprotein E in neurodegeneration and cardiovascular disease. *Expert Rev Proteomics.* 2014;11(3):371-81.

18. Abildstrom H, Rasmussen LS, Rentowl P, Hanning CD, Rasmussen H, Kristensen PA, Moller JT. Cognitive dysfunction 1-2 years after non-cardiac surgery in the elderly. ISPOCD group. International Study of Post-Operative Cognitive Dysfunction. *Acta Anaesthesiol Scand.* 2000;44(10):1246-5.

19. Moller JT, Cluitmans P, Rasmussen LS, Houx P, Rasmussen H, Canet J, Rabbitt P, Jolles J, Larsen K, Hanning CD, Langeron O, Johnson T, Lauven PM, Kristensen PA, Biedler A, van Beem H, Fraidakis O, Silverstein JH, Beneken JE, Gravenstein JS. Long-term postoperative cognitive dysfunction in the elderly ISPOCD1 study. ISPOCD investigators. International Study of Post-Operative Cognitive Dysfunction. *Lancet.* 1998;351(9106):857-61.

20. Rasmussen LS, Larsen K, Houx P, Skovgaard LT, Hanning CD, Moller JT, ISPOCD group. The International Study of Postoperative Cognitive Dysfunction. The assessment of postoperative cognitive function. *Acta Anaesthesiol Scand.* 2001;45(3):275-89.

21. Lewis MS, Maruff P, Silbert BS, Evered LA, Scott DA. Detection of postoperative cognitive decline after coronary artery bypass graft surgery is affected by the number of neuropsychological tests in the assessment battery. *Annals Thorac Surg.* 2006;81(6):2097-104.

22. Laalou FZ, Jochum D, Pain L. [Postoperative cognitive dysfunction (POCD): strategy of prevention, assessment and management]. *Ann Fr Anesth Reanim.* 2011 Oct;30(10):e49-53.

23 Murphy GS, Szokol JW, Greenberg SB, Avram MJ, Vender JS, Nisman M, Vaughn J. Preoperative dexamethasone enchances quality of recovery after laparoscopic cholecystectomy. *Anesthesiology*. 2011;114(4):882-90.

24 De Oliveira GS, Almeida MD, Benzon HT, McCarthy RJ. Perioperative single dose systemic dexamethasone for postoperative pain. *Anesthesiology*. 2011;115(3):457-9.

25 Rimaitis K, Svitojute A, Macas A. The influence of dexamethasone and Ketolganon postoperative nausea and vomiting and estimation of risk factors in women undergoing gynecologic laparoscopic surgeries. *Medicina (Kaunas)*. 2010;46(4):261-7.

26 Fukami Y, Terasaki M, Okamoto Y, Sakaguchi K, Murata T, Ohkubo M, Nishimae K. Efficacy of preoperative dexamethasone in patients with laparoscopic cholecystectomy: a prospective randomized double-blind study. *J Hepatobiliary Pancreat Surg.* 2009;16(3):367-71.

27 Evered LA, Silbert BS, Scott DA, Maruff P, Ames D, Choong PF. Preexisting cognitive impairment and mild cognitive impairment in subjects presenting for total hip joint replacement. *Anesthesiolgy.* 2011;6(114):1297-304.

28 Avidan MS, Searleman AC, Storandt M, Barnett K, Vannucci A, Saager L, Xiong C, Grant EA, Kaiser D, Morris JC, Evers AS. Long-term cognitive decline in older subjects was not attributable to non-cardiac surgery or major illness. *Anesthesiology*. 2009;111(5):964-70.

29 Silverstein JH, Allore AH, Deiner S, Sano M, Rasmussen L. Is postoperative cognitive decline clinically relevant? *Anesthesiology*. 2010;112(5):1280-97.

30 Rasmussen L. Postoperative cognitive dysfunction: incidence and prevention. *Best Pract Res Clin Anaesthesiol.* 2006;20(2):315-30.

31 Avidan MS, Xiong C, Evers A. Postoperative cognitive decline: the unsubstantiated phenotype. *Anesthesiology.* 2010;113(5):1246-48.

32 Rasmussen L. Defining postoperative cognitive dysfuction. *Eur J Anaesthesiol.* 1998;15(6):761-4.

33 Rasmussen LS, Larsen K, Houx P, Skovgaard LT, Hanning CD, Moller JT. The assessment of postoperative cognitive function. *Acta Anaesthesiol Scand.* 2001;45(3):275-89.

34 Rohan D, Buggy DJ, Crowley S, Ling FK, Gallagher H, Regan C, Moriarty DC. Increased incidence of postoperative cognitive dysfunction 24 hr after minor surgery in the elderly. *Can J Anesth.* 2004;52(2):137-42.

35 McAlpine JN, Hodgson EJ, Abramowitz S, Richman SM, Su Y, Kelly MG, Luther M, Baker L, Zelterman D, Rutherford TJ, Schwartz PE. The incidence and risk factors associated with postoperative delirium in geriatric patients undergoing for suspected gynecologic malignancies. *Gynecol Oncol.* 2008;109(2):296-302.

36 Monk TG, Weldon BC, Garvan CW, Dede DE, van der Aa MT, Heilman KM, Gravenstein JS. Predictors of cognitive after major noncardiac surgery. *Anesthesiology*. 2008;108(1):18-30.

37 Monk TG, Saini V, Weldon C, Sigl J. Anesthetic management and one-year mortality after noncardiac surgery. *Anesth Analg.* 2005;100(1):4-10.

38 Vicario A, Martinez CD, Baretto D, Casale AD, Nicolosi L. Hipertension and cognitive decline: impact on executive function. *J Clin Hypertens (Greenwich)*. 2005;7(10):598-604.

39 Münte S, Schmidt M, Meyer M, Nager W, Lüllwitz E, Münte TF, Piepenbrock S. Implicit memory for words played during isoflurane or propofol-based anesthesia. *Anesthesiology.* 2002;96(3):588-94.

40 Iselin-Chaves IA, Willems SJ, Jermann FC, Forster A, Adan SR, Van der Linden M. Investigation of implicit memory during isoflurane anesthesia for elective surgery using the process dissociation procedure. *Anesthesiology.* 2005;103(5):925-33.

41 Paulo DLV, Yassuda MS. The relation between memory complaints in the elderly and education, cognitive performance, depression andanxiety. *Rev Psiq Clin.* 2010;37(1):23-6.

42 Bilotta F, Doronzio A, Stazi E, Titi L, Zeppa IO, Cianchi A, Rosa G, Paoloni FP, Bergese S, Asouhidou I, Ioannou P, Abramowicz AE, Spinelli A, Delphin E, Ayrian E, Zelman V, Lumb P. Early postopertive ognitive dysfunction and postoperative delirium after anaesthesia with various hypnotics: study protocol for a randomised controlled trial- the PINOCCHIO trial. *Trials*. 2011;12:170.

43 Fong HK, Sands LP, Leung JM. The role of postoperative analgesia in delirium and cognitive decline in elderly patients: a systematic review. *Anesth Analg.* 2006;102(4):1255-66.

44 Ille R, Lahousen T, Schweiger S, Hofmann P, Kapfhammer HP. Influence of patient-related risk factors on cognitive performance, emocional state, and convalescence after cardiac surgery. *Cardiovasc Revasc Med.* 2007;8(3):166-9.

45 Smith PJ, Attix DK, Weldon CB, Greene NH, Monk TG. Executive function and depression as independent risk factors for postoperative delirium. *Anesthesiology.* 2009;110(4):781-7.

46 Gao L, Taha R, Gauvin D, Othmen LB, Wang Y, Blaise G. Postoperative cognitive dysfunction after cardiac surgery. *Chest.* 2005;128(5):3664-70.

47 Lewis MC, Nevo I, Paniagua MA, Ben-Ari A, Pretto E, Eisdorfer S, Davidson E, Matot I, Eisdorfer C. Uncomplicated general anesthesia in elderly results in cognitive decline: does cognitive decline predict morbity and mortality? *Med Hypotheses.* 2007;68(3):484-92.

48 Cottrell J. We care, therefore we are: anesthesia-related morbility and mortality. Anesthesiology. 2008;109(3):377-88.

49 Shaw PJ, Bates D, Cartlidge NE, French JM, Heaviside D, Julian DG, Shaw DA. Nerologic and neuropsychological morbility fllowing major surgery: comparison of coronary bypass and peripheral vascular surgery. *Stroke.* 1987;18(4):700-7.

50 Dijkstra JB, Houx PJ, Jolles J. Cognition after major surgery in the elderly: test performance and complaints. *Br J Anaesthesia.* 1999;82(6):867-74.

51 Monk TG, Weldon BC, Dede DE, van der Aa MT, Heiman KM, Gravenstein JS. Predictors of cognitive dysfunction after major noncardiac surgery. *Anesthesiology.* 2008;108(1):18-30.

52 Laalou FZ, Jochum D, Pain L. [Postoperative cognitive dysfunction (POCD): strategy of prevention, assessment and management]. *Ann Fr Anesth Reanim.* 2011;30(10):e49-53.

53 Tardiff BE, Newman MF, Saunders AM, Strittmatter WJ, Blumenthal JA, White WD, Croughwell ND, Davis D Jr, Roses AD, Reves JG. Preliminary report of a genetic basis for cognitive decline after cardiac operations. *Ann Thorac Surg.* 1997;64(3):715-20.

54 Arozullah AM, Khuri SF, Henderson WG, Daley J. Development and validation of a multifactorial risk index for predicting postoperative pneumonia after major noncardiac surgery. *Ann Intern Med.* 2001;135(10):847-57.

55 Millar K, Asbury AJ, Murray GD. Pre-existing cognitive impairment as a factor influencing outcome after cardiac surgery. *Br J Anaesthesia.* 2001;86(1):63-7.

56 Nötzold A, Michel K, Khattab AA. Diabetes mellitus increases adverse neurocognitive outcome after coronary artery bypass grafting surgery. *Thorac Cardiovasc Surg.* 2006;54(5):307-12.

57 Ballard CG, Morris CM, Rao H, O'Brien JT, Barber R, Stephens S. APOE e4 and cognitive decline in older stroke patients with early cognitive impairment. *Neurology*. 2004;63(8):1399-402.

58 Wolk DA, Dickerson BC. Apolipoprotein E (APOE) genotype has dissociable effects on memory and attentional-executive network function in Alzheimer's disease. *Proc Natl Acad Sci USA.* 2010;107(22):10256-61.

59 Kofke WA, Konitzer P, Meng QC, Guo J, Cheung A. The effect of apolipoprotein E genotype on neuron specific enolase and S-100B levels after cardiac surgery. *Anesth Analg.* 2004;99(5):1323-5.

60 Bettelli G. Anaesthesia for the elderly outpatient: preoperative Assessment and evaluation, anaesthetic technique and postoperative pain management. *Anesthesiology*. 2010;23(6):726-31.

61 Jonker C, Schmand B, Lindeboom J, Havekes LM, Launer LJ. Association between apolipoprotein E e4 and the rate of cognitive decline in community-dwelling elderly individuals with and without dementia. *Arch Neurol.* 1998;55:1065-9.

62 Xavier FMF, Ferraz MPT, Bertollucci P, Potares D, Moriguchi EH. The prevalence of major depression and its impact in the quality of life, sleep patterns and cognitive function in a octonagerian population. *Rev Bras Psiquiatr.* 2001;23(2):62-70.

63 Funder KS, Steinmetz J. Pot-operative cognitive dysfunction- Lessons from the ISPOCD studies. *Trends Anaesth Crit Care*. 2012;2(3):94-7.

64 Moller JT, Cluitmans P, Rasmussen LS, Houx P, Rasmussen H, Canet J, Rabbitt P, Jolles J, Scient KLC, Hanning CD, Langeron MD, Johnson T, Lauven PM, Kristensen PA, Biedler A, Been H, Fraidakis O, Silverstein JH, Beneken JEW, Gravenstein JS. Long-term postoperative cognitive dysfunction in the elderly: ISPOCD1 study. *Lancet*. 1998;351(9106):857-61.

65 Lee S, Kawachi I, Berkman LF, Grodstein F. Education, other socioeconomic indicators, and cognitive function. *Am J Epidemiol*. 2003;157(8):712-20.

66 Asilioglu K, Celik SS. The effect of preoperative education on anxiety of open cardiac surgery patients. *Patient Educ Couns*. 2004;53(1):65-70.

67 Grodstein F, Chen J, Wilson RS, Manson JE. Type 2 Diabetes and Cognitive Function in Community-Dwelling Elderly Women. *Diabetes Care.* 2001;24(6):1060-5.

68 Alencar RC, Cobas RA, Gomes MB. Assessment of cognitive status in patients with type 2 diabetes through the mini-mental sttus examination: a cross-sectional study. *Diabetol Metab Syndr.* 2010;2(10):2-6.

69 Mathew JP, Podgoreanu MV, Grocott HP, White WD, Morris RW, Stafford-Smith M, Mackensen GB, Rinder CS, Blumenthal JA, Schwinn DA, Newman MF. Genetic variants in P-selectin and C-reactive protein influence susceptibility to cognitive decline after cardiac surgery. *J Am Coll Cardiol.* 2007;49(19):1934-42.

70 Papon MA, Whittington RA, El_Khoury NB, Planel E. Alzheimer's disease and anesthesia. *Front Neurosci*. 2011;4(272):1-7.

71 Kleindienst A, Ross Bullock M. A critical analysis of the roles of the neurotropic protein S100B in acute brain injury. *J Neurotrauma.* 2006;23(8):1185-200.

72 Connolly ES Jr, Winfree CJ, Rampersad A, Ruchey S, Willian JM, Mocco J, Solomon RA, Todd G, Quest DO, Stern Y, Heyer EJ. Serum S100B protein levels are correlated with subclinical neurocognitive declines after carotic endarterectomy. *Neurosurgery.* 2001;49(5):1076-83.

73 Rasmussen LS, Christiansen M, Rasmussen H, Kristensen PA, Moller JT. Do boold concentrations of neurone specific enolase and s-100B protein reflect cognitive dysfunction after abdominal surgery? *Br J Anaesth.* 2000;84(2):242-4.

74 Tiainen M, Roine RO, Ville P, Takkunen O. Serum neuron-specific enolase and S-100B protein in cardiac arrest patients treated with hypothermia. *Stroke.* 2003;34(12):2881-6.

75 Ishida K, Gohara T, Kawata R, Ohtake K, Morimota Y, Sakabe T. Are serum S100B proteins and neuron-specific enolase predictors of cerebral damage in cardiovascular surgery? *J Cardiothorac Vasc Anesth.* 2003;17(1):4-9.

76 Abildstrom H, Christiansen M, Siersma VD, Rasmussen LS. Apolipoprotein E genotype and cognitive dysfunction after noncardiac surgery. *Anesthesiology.* 2004;101(4):855-61.

77 Abildstrom H, Rasmussen LS, Hanning CD, Rasmussen H, Kristensen PA, Moller JT. Cognitive dysfunction 1-2 years after non-cardiac surgery in the elderly. *Acta Anaesthesiol Scand.* 2000;44(10):1246-51.

78 Steinmetz J, Christensen KB, Lund T, Lohse N, Rasmunssen LS. Long-term consequences of postoperative cognitive dysfunction. *Anesthesiology.* 2009;110(3):548-55.

79 Baranov D, Bickler PE, Crosby GJ, Culley DJ, Eckenhoff MF, Eckenhoff RG, Hogan KJ, Jevtovic-Todorovic V, Palotás A, Perouansky M, Planel E, Siverstein JH, Wei H, Whittington RA, Xie Z, Zuo Z. Consensus statement: First International Workshop on Anesthesics and Alzeimer's Disease. *Anesth Analg.* 2009;108(5):1627-30.

80 Schlindwein-Zanini R. Dementia in the elderly: neuropsychological aspects. *Rev Neurocienc.* 2010;18(2):220-6.

81 Brown JP, Sollers JJ, Thayer JF, Zonderman AB, Waldstein SR. Blood pressure reactivity and cognitive function in the Baltimore Longitudinal Study of Aging. *Health Psychol.* 2009;28(5):641-6.

82 Pelinka LE, Kroepfl A, Schmidhammer R, Krenn M, Buchinger W, Redl H, Raabe A. Glial fibrillary acidic protein in serum after traumatic brain injury and multiple trauma. *J Trauma*. 2004;57(5):1006-12.

83 Yocum GT, Gaudet JG, Teverbaugh LA, Quest DO, McCormick PC, Connolly ES Jr, Heyer EJ. Neurocognitive performance in hypertensive patients after spine surgery. *Anesthesiology.* 2009;110(2):254-61.

84 Grigore AM, Grocott HP, Mathew JP, Phillips-Bute B, Stanley TO, Butler A, Landolfo KP, Reves JG, Blumenthal JA, Newman MF. The rewarming rate and increased peak temperature alter neurocognitive outcome after cardiac surgery. *Anesth Analg.* 2002;94(1):4-10.

85 Tournay-Jetté E, Dupuis G, Bherer L, Deschamps A, Cartier R, Denault A. The relationship between cerebral oxygen saturation changes and postoperative cognitive dysfunction in elderly patients after coronary artery bypass graft surgery. *J Cardiothorac Vasc Anesth.* 2011 Feb;25(1):95-104.

86 Johnson T, Monk T, Rasmunssen LS. Postoperative cognitive dysfunction in middle-aged patients. *Anesthesiology.* 2002;96(6):1351-7.

87 Kadoi Y. Pharmacological neuroprotection during cardiac surgery. *Asian Cardiovasc Thorac Ann.* 2007;15(2):167-77.

88 Mathew JP, Mackensen GB, Phillips-Bute B, Grocott HP, Glower DD, Laskowitz DT, Blumenthal JA, Newman MF. Randomized, double-blinded, placebo controlled study of neuroprotection with lidocaine in cardiac surgery. *Stroke.* 2009;40(3):880-7.

89 Haljan G, Maitland A, Buchan A, Arora RC, King M, Haigh J, Culleton B, Faris P, Zygun D. The erythropoietin neuroprotective effect: assessment in CABG surgery (TENPEAKS): a randomized, double- blind, placebo controlled, proof-of-concept clinical trial. *Stroke.* 2009;40(8):2769-75.

90 Grocott HP, Yoshitani K. Neuroprotection during cardiac surgery. *J Anesth.* 2007;21(3):367-77.

91 Kudoh A, Takase H, Takashira Y, Takazawa T. Postoperative confusion increases in elderly long-term benzodiazepine users. *Anesth Analg.* 2004;99(6):1674-8.

92 Leung JM, Sands LP. Long-term cognitive decline. Is there a link to surgery and anesthesia? *Anesthesiology.* 2009;111(5):931-2.

93 Mandal PK, Schifilliti D, Mafrica F, Fodale V. Inhaled anesthesia and cognitive performance. *Drugs Today*. 2009;45(1):47-54.

94 Nielsen KC, Tucker MS, Marcy S, Steele S. Outcomes after regional anesthesia. *Int Anesthesiol Clin*. 2005;43(3):91-110.

95 Heyer EJ, Gold MI, Kirby EW, Zurica J, Mitchell E, Halazun HJ, Teverbaugh L, Sciacca R, Solomon RA, Quest DO, Maldonado TS, Riles TS, Connolly ES Jr. A study of cognitive dysfunction in patients having carotid endarterectomy performance with regional anesthesia. *Anesth Analg.* 2008;107(2):636-42.

96 De Oliveira GS AM, Benzon HT, McCarthy RJ. Perioperative single dose systemic dexamethasone for postoperative pain. *Pain Med.* 2011;115(3):575-88.

97 Kardash KJ BF, Tessler MJ, Velly AM. Single-dose dexamethasone reduces dynamic pain after total hip arthroplasty. *Anesth Analg.* 2008;106(4):1253-7.

98 Wallenborn J GG, Bulst D, Behrends K, Wallenborn H, Rohrbach A, Krause U, Kuhnast T, Wiegel M, Olthoff. Prevention of postoperative nause and vomiting by metoclopramide combined with dexamethasone: randomised double blind multicentre trial. *BMJ.* 2006;333(7563):324.

99 Feo CV, Sortini D, Ragazzi R, De Palma M, Liboni A. Randomized clinical trial of the effect of preoperative dexamethasone on nausea and vomiting after laparoscopic cholecystectomy. *Br J Surg*. 2006;93(3):295-9.

100 Saniova B, Drobny M, Sulaj M. Delirium and postoperative cognitive dysfunction after general anesthesia. *Med Sci Monit*. 2009;15(5):CS81-7.

101 Perouansky M, Hemmings HC, Jr. Neurotoxicity of general anesthetics: cause for concern? *Anesthesiology.* 2009;111(6):1365-71.

102 Gaba V. Correlation of the depth of anesthesia with POCD (postoperative cognitive dysfunction). *Anesth Analg.* 2007;104(5):1298; author reply -9.

103 Royse C. Is depth of anesthesia, as assessed by the bispectral index, related to postoperative cognitive dysfunction and recovery? *Anesth Analg.* 2007;104(5):1297; author reply -8.

104 Ballard C, Jones E, Gauge N, Aarsland D, Nilsen OB, Saxby BK, Lowery D, Corbett A, Wesnes K, Katsaiti E, Arden J, Amoako D, Prophet N, Purushothaman B, Green D. Optimised anaesthesia to reduce post operative cognitive decline (POCD) in older patients undergoing elective surgery, a randomised controlled trial. *PloS One*. 2012;7(6):e37410.

105 Wajman JR, Oliveira FF, Schultz RR, Marin SM, Bertolucci PH. Educational bias in the assessment of severe dementia: Brazilian cutoffs for severe Mini-Mental State Examination. *Arq Neuropsiquiatr.* 2014;72(4):273-7.

106 Van Heugten CM, Walton L, Hentschel U. Can we forget the Mini-Mental State Examination? A systematic review of the validity of cognitive screening instruments within one month after stroke. *Clin Rehabilit.* 2014.

107 Beck AT, Ward CH, Mendelson M, Mock J, Erbaugh J. An inventory for measuring depression. *Arch Gen Psychiatry*. 1961;4:561-71.

108 Beck AT, Steer RA. *Beck depression Inventory. manual.* San Antonio: Psychology Corporation. 1993.

109 Chaves ML, Camozzato AL, Ferreira ED, Piazenski I, Kochhann R, Dall'Igna O, Mazzini GS, Souza DO, Portela LV. Serum levels of S100B and NSE proteins in Alzheimer's disease patients. *J Neuroinflammation.* 2010;7:6.

110 Schaf DV, Tort AB, Fricke D, Schestatsky P, Portela LV, Souza DO, Rieder CR. S100B and NSE serum levels in patients with Parkinson's disease. *Parkinsonism Relat Disord.* 2005;11(1):39-43.

111 Calderon LM, Guyette FX, Doshi AA, Callaway CW, Rittenberger JC, Post Cardiac Arrest S. Combining NSE and S100B with clinical examination findings to predict survival after resuscitation from cardiac arrest. *Resuscitation.* 2014;85(8):1025-9.

112 Donato R, Cannon BR, Sorci G, Riuzzi F, Hsu K, Weber DJ, Geczy CL. Functions of S100 proteins. *Cur Mol Med.* 2013;13(1):24-57.

113 Schroeter ML, Steiner J, Schonknecht P, Mueller K. Further evidence for a role of S100B in mood disorders: a human gene expression mega-analysis. Journal of psychiatric research. 2014;53:84-6.

114 Lowe C, Rabbitt P. Test/re-test reliability of the CANTAB and ISPOCD neuropsychological batteries: theorical and practical issues. *Neuropsychologia.* 1998;36(9):915-23.

APÊNDICE

Apêndice E - Testes Neuropsicológicos (modelos)

ETIQUETA DE IDENTIFICAÇÃO

CONCLUSÃO PSICOLÓGICA:

INCLUÍDO: ( ) SIM ( ) NÃO

NOME:_______________ **R.G.:**_______________

Sexo:____ **Idade(anos):**________ **Peso(kg):** _____ **Altura(cm):**_____ **IMC:**_______________

Endereço_______________

Cidade: _______________ **Estado:**_______ **Fone (__)**_______________

Cel (__)_____________ **Outros fones/contatos**_______________ _______________

Estado civil

( ) solteiro (a)
( ) casado(a)
( ) divorciado(a)
( ) viúvo(a)
( ) união estável

Escolaridade

( ) Ensino fundamental incompleto
( ) Ensino fundamental completo
( ) Ensino médio completo
( ) Ensino médio completo
( ) Superior incompleto
( ) Superios completo
( ) Pós-graduação

Anos de estudo: ____________

Profissão:_______________

Emprego:

( ) empregado(a)
( ) desempregado(a)
( ) aposentado(a)
( ) trabalhos eventuais, sem vinculo empregatício
( ) profissional liberal

Renda familiar: R$: _______________

( ) Não quiz informar
( ) 0
( ) 0-1 salário mínimo
( ) 1-5 salários mínimos
( ) 6-10 salários mínimos
( ) 10-15 salários mínimos
( ) 15-20 salários mínimos
( ) mais que 20 salários mínimos

Número de pessoas que vivem com esta renda: ____

MINI-MENTAL

(FOLSTEIN, FOLSTEIN & MCHUGH, 1975)

Paciente: ____

Data de avaliação: __________ Avaliador: ______________________

Orientação

1) Dia da Semana (1 ponto) ( )
2) Dia do Mês (1 ponto) ( )
3) Mês (1 ponto) ( )
4) Ano (1 ponto) ( )
5) Hora aproximada (1 ponto) ( )
6) Local específico (andar ou setor) (1 ponto) ( )
7) Instituição (residência, hospital, clínica) (1 ponto) ( )
8) Bairro ou rua próxima (1 ponto) ( )
9) Cidade (1 ponto) ( )
10) Estado (1 ponto) ( )

Memória Imediata

Fale três palavras não relacionadas. Posteriormente pergunte ao paciente pelas 3 palavras. Dê 1 ponto para cada resposta correta. ( )

Depois repita as palavras e certifique-se de que o paciente as aprendeu, pois mais adiante você irá perguntá-las novamente.

Atenção e Cálculo

(100-7) sucessivos, 5 vezes sucessivamente (93,86,79,72,65)
(1 ponto para cada cálculo correto) ( )

Evocação

Pergunte pelas três palavras ditas anteriormente
(1 ponto por palavra) ( )

Linguagem

1) Nomear um relógio e uma caneta (2 pontos) ( )
2) Repetir "nem aqui, nem ali, nem lá" (1 ponto) ( )
3) Comando: "pegue este papel com a mão direita, dobre ao meio e coloque no chão (3 pontos) ( )
4) Ler e obedecer: "feche os olhos" (1 ponto) ( )
5) Escrever uma frase (1 ponto) ( )
6) Copiar um desenho (1 ponto) ( )

Escore: (/ 30)

Paciente: ________________________________ *Idade: __________
Data de Avaliação: ______________

ESCREVA UMA FRASE

____

COPIE O DESENHO

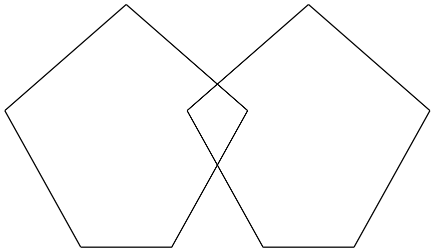

* CONTINUAÇÃO MINI-MENTAL

ISPOCD 2

Test Score Form **testversion 1** — Portugese

Date of first session (yymmdd) |__|__|/|__|__|/|__|__| *Subject ID* |__| |__|__|__|

Mini-Mental State Examination (MMSE) Total score: |__|__| Status: __

Verbal Learning Test (VLT)

	trial 1	trial 2	trial 3	delayed trial		
placa	☐	☐	☐	☐	placa	Tr.1 #corr. |__|__
leão	☐	☐	☐	☐	leão	
cama	☐	☐	☐	☐	cama	Tr.1 errors |__
viola	☐	☐	☐	☐	viola	
anel	☐	☐	☐	☐	anel	Tr.2 #corr. |__|__
relva	☐	☐	☐	☐	relva	
lápis	☐	☐	☐	☐	lápis	Tr.2 errors |__
água	☐	☐	☐	☐	água	
escadas	☐	☐	☐	☐	escadas	Tr.3 #corr. |__|__
mala	☐	☐	☐	☐	mala	
boca	☐	☐	☐	☐	boca	Tr.3 errors |__
avião	☐	☐	☐	☐	avião	
uva	☐	☐	☐	☐	uva	Del.Rc.#corr. |__|__
montanha	☐	☐	☐	☐	montanha	
cão	☐	☐	☐	☐	cão	Del.Rec. errors |__
error	________	________	________	________	error	
error	________	________	________	________	error	time end tr. 3 __ __ : __ __ Status |__

Stroop Colour Word Test (SCW), Part I time |__|m|__|__|s|__|__|

azul	azul	amarelo	verde	vermelho	azul	amarelo	amarelo	verde	amarelo	
vermelho	vermelho	verde	vermelho	verde	vermelho	azul	azul	verde	vermelho	errors: |__|__
amarelo	verde	amarelo	amarelo	verde	vermelho	vermelho	verde	amarelo	vermelho	
azul	amarelo	azul	azul	amarelo	verde	azul	azul	verde	vermelho	

Part II time: |__|m|__|__|s|__|__|

verde	vermelho	amarelo	amarelo	vermelho	azul	verde	azul	azul	amarelo	
amarelo	vermelho	azul	amarelo	azul	verde	vermelho	azul	verde	vermelho	errors: |__|__
azul	amarelo	verde	azul	vermelho	amarelo	verde	vermelho	verde	amarelo	
amarelo	verde	amarelo	verde	vermelho	azul	azul	vermelho	verde	vermelho	

Part III time: |__|m|__|__|s|__|__|

amarelo	vermelho	azul	amarelo	vermelho	azul	amarelo	verde	azul	verde	
verde	azul	amarelo	verde	amarelo	azul	vermelho	amarelo	verde	vermelho	errors: |__|__
vermelho	amarelo	amarelo	vermelho	vermelho	azul	verde	amarelo	azul	verde	
azul	verde	vermelho	vermelho	verde	azul	amarelo	vermelho	azul	verde	Status: |__

Status values: 1= Complete and reliable [illegible]

ISPOCD 2

Test Score Form **testversion 2** — Portugese

Date of first session (YY/MM/DD) |__|__|/|__|__|/|__|__| Subject ID |__| |__|__|__|

Mini-Mental State Examination (MMSE) Total score: |__|__| Status: |__|

Verbal Learning Test (VLT)

	trial 1	trial 2	trial 3	delayed trial		
banana	☐	☐	☐	☐	banane	Tr.1 #corr. |__|__|
ponte	☐	☐	☐	☐	ponte	
gato	☐	☐	☐	☐	gato	Tr.1 errors |__|
faca	☐	☐	☐	☐	faca	
tigre	☐	☐	☐	☐	tigre	Tr.2 #corr. |__|__|
louceiro	☐	☐	☐	☐	louceiro	
vassoura	☐	☐	☐	☐	vassoura	Tr.2 errors |__|
sapato	☐	☐	☐	☐	sapato	
flôr	☐	☐	☐	☐	flôr	Tr.3 #corr. |__|__|
carta	☐	☐	☐	☐	carta	
vinho	☐	☐	☐	☐	vinho	Tr.3 errors |__|
parede	☐	☐	☐	☐	parede	
telefone	☐	☐	☐	☐	telefone	Del.Rc.#corr. |__|__|
mão	☐	☐	☐	☐	mão	
carro	☐	☐	☐	☐	carro	Del.Rec. errors |__|
error	______	______	______	______	error	
error	______	______	______	______	error	time end tr. 3 _ _ : _ _ Status |__|

Stroop Colour Word Test (SCW), Part I time |__|h|__|__|.|__|__|

azul	azul	amarelo	verde	vermelho	azul	amarelo	amarelo	verde	amarelo	
vermelho	vermelho	verde	vermelho	verde	vermelho	azul	azul	verde	vermelho	errors: |__|__|
amarelo	verde	amarelo	amarelo	verde	vermelho	vermelho	verde	amarelo	vermelho	
azul	amarelo	azul	azul	amarelo	verde	azul	azul	verde	vermelho	

Part II time: |__|h|__|__|.|__|__|

verde	vermelho	amarelo	amarelo	vermelho	azul	verde	azul	azul	amarelo	
amarelo	vermelho	azul	amarelo	azul	verde	vermelho	azul	verde	vermelho	errors: |__|__|
azul	amarelo	verde	azul	vermelho	amarelo	verde	vermelho	verde	amarelo	
amarelo	verde	amarelo	verde	vermelho	azul	azul	vermelho	verde	vermelho	

Part III time: |__|h|__|__|.|__|__|

amarelo	vermelho	azul	amarelo	vermelho	azul	amarelo	verde	azul	verde	
verde	azul	amarelo	verde	amarelo	azul	vermelho	amarelo	verde	vermelho	errors: |__|__|
vermelho	amarelo	amarelo	vermelho	vermelho	azul	verde	amarelo	azul	verde	
azul	verde	vermelho	vermelho	verde	azul	amarelo	vermelho	azul	verde	Status: |__|

Status values: 1= Complete and reliable 2= techn. problems 3= refusal/unself. motivation patient 4=fysical or cogn. limit. 5= patient deviated from instr. 6= test not adm.

ISPOCD 2

Test Score Form **testversion 3** -- Portugese

Date of first session (yy/mm/dd) |__|__|/|__|__|/|__|__| Subject ID |__|__|__|__|__|

Mini-Mental State Examination (MMSE)   Total score: |__|__| Status: __

Verbal Learning Test (VLT)

	trial 1	trial 2	trial 3	delayed trial		
rádio	❑	❑	❑	❑	rádio	Tr.1 #corr. __ __
barba	❑	❑	❑	❑	barba	
combóio	❑	❑	❑	❑	combóio	Tr.1 errors __
limão	❑	❑	❑	❑	limão	
sol	❑	❑	❑	❑	sol	Tr.2 #corr. __ __
cavalo	❑	❑	❑	❑	cavalo	
colher	❑	❑	❑	❑	colher	Tr.2 errors __
elefante	❑	❑	❑	❑	elefante	
lâmpada	❑	❑	❑	❑	lâmpada	Tr.3 #corr. __ __
chave	❑	❑	❑	❑	chave	
chapéu	❑	❑	❑	❑	chapéu	Tr.3 errors __
cacto	❑	❑	❑	❑	cacto	
livro	❑	❑	❑	❑	livro	Del.Rc #corr. __ __
leite	❑	❑	❑	❑	leite	
casa	❑	❑	❑	❑	casa	Del.Rec. errors __
[illegible]	________	________	________	________	[illegible]	
[illegible]	________	________	________	________	[illegible]	time end tr. 3 __ __ : __ __ Status __

Stroop, Part I   time |__|:|__|__|.|__|__|

azul	azul	amarelo	verde	vermelho	azul	amarelo	amarelo	verde	amarelo	
vermelho	vermelho	verde	vermelho	verde	vermelho	azul	azul	verde	vermelho	errors __ __
amarelo	verde	amarelo	amarelo	verde	vermelho	vermelho	verde	amarelo	vermelho	
azul	amarelo	azul	azul	amarelo	verde	azul	azul	verde	vermelho	

Part II   time: |__|:|__|__|.|__|__|

verde	vermelho	amarelo	amarelo	vermelho	azul	verde	azul	azul	amarelo	
amarelo	vermelho	azul	amarelo	azul	verde	vermelho	azul	verde	vermelho	errors __ __
azul	amarelo	verde	azul	vermelho	amarelo	verde	vermelho	verde	amarelo	
amarelo	verde	amarelo	verde	vermelho	azul	azul	vermelho	verde	vermelho	

Part III   time: |__|:|__|__|.|__|__|

amarelo	vermelho	azul	amarelo	vermelho	azul	amarelo	verde	azul	verde	
verde	azul	amarelo	verde	amarelo	azul	vermelho	amarelo	verde	vermelho	errors: __ __
vermelho	amarelo	amarelo	vermelho	vermelho	azul	verde	amarelo	azul	verde	
azul	verde	vermelho	vermelho	verde	azul	amarelo	vermelho	azul	verde	Status: __

Nome: ____Avaliação Escrita

Idade:_______ Escolaridade:__________ Data: ______/______/______ Lateralidade:______

(	⊤	⊢	┌	⊣	>	+	)	⊥
1	2	3	4	5	6	7	8	9

(	⊣	⊤	(	⊢	>	⊤	┌	(	>	⊤	(	>	(	⊤

┌	>	(	⊤	⊣	>	⊢	┌	(	⊤	>	⊥	┌	⊢	)

┌	⊣	+	)	(	⊢	+	┌	)	⊣	⊤	⊥	⊢	┌	+

⊤	┌	⊣	(	>	┌	(	⊣	>	+	⊥	)	⊢	>	┌

⊥	⊣	)	⊢	>	+	┌	⊣	⊤	⊢	+	⊥	⊤	)	(

>	⊥	+	⊤	⊢	>	┌	⊥	(	+	⊤	⊣	>	)	┌

⊤	)	+	⊥	⊢	+	)	⊣	(	⊥	⊤	(	┌	⊢	>

⊣	⊤	(	>	┌	⊤	(	>	⊥	+	⊢	⊣	┌	)	⊥

Total ________

Entrevista Telefônica para Avaliação do Estado Cognitivo - versão modificada (TICS-M)

Pontuar '1' para cada resposta correta e '0' para incorreta

Orientação

1. (i) Que dia da semana é hoje?	*Dia*	☐
(ii) Qual a data de hoje?	*Dia*	☐
	Mês	☐
	Ano	☐
(iii) Em qual estação nós estamos?	*Estação*	☐
2. Qual é sua idade?	*Idade*	☐
3. Qual é o seu número de telefone?	*número*	☐

Registro / Memória imediata

4. Eu vou ler uma lista de 10 palavras. Por favor, ouça com atenção e tente gravar estas palavras na sua memória. Quando eu terminar, você tem que me dizer todas as palavras que você lembrar, não importa a ordem. Você está pronto (a)?

Agora, me diga todas as palavras que você conseguir lembrar

Cabana	☐
Cachimbo	☐
Elefante	☐
Peito	☐
Seda	☐
Teatro	☐
Relógio	☐
Chicote	☐
Travesseiro	☐
Gigante	☐

Atenção / Cálculo

5. Por favor, quanto é 100 menos 7?	*93*	
Agora continue diminuindo 7 do número que sobrar	*86*	
até que eu peça para você parar.	*79*	
	72	
	65	
6. Por favor, conte de 1 a 20, só que de trás para frente	*Sem erros*	

Memória de compreensão, semântica e episódica recente

7. O que as pessoas geralmente utilizam para cortar papel?	*Tesoura*	
8. Qual é a planta verde cheia de espinhos encontrada no deserto?	*Cactus, macambira, mandacaru, palma ou similar*	
9. Quem é o atual presidente do Brasil ?	*Nome completo*	
10. Quem é o atual governador de São Paulo? *(adaptar pergunta conforme Estado)*	*Nome completo*	
11. Qual é o oposto de Leste?	*Oeste*	

Linguagem / Repetição

12. Por favor, repita isso: Igreja Presbiteriana	*Exatamente correto*	

Evocação tardia

13. Por favor, repita a lista de 10 palavras	*Cabana*		
que eu li anteriormente	*Cachimbo*		
	Elefante		
	Peito		
	Seda		
	Teatro		
	Relógio		
	Chicote		
	Travesseiro		
	Gigante		
			Máximo de 39 pontos

**SF-36 PESQUISA EM SAÚDE**	**SCORE :_______**

Nome: ....Idade:..............................
Sexo:............................... Grau de Instrução:................................ ....
RG...................... ....; CPF:....................................... -..............
Endereço:............................ ....- nº.........
Estado:.............................. ....; Cidade:.......................... .CEP:....................
Telefone para contato:................................ ....Falar com:.........................
Celular para contato:..F alar com:.......................
Data da cirurgia:....................................Ci rurgia:.......................... ....

Instruções: Esta pesquisa questiona você sobre sua saúde. Estas informações nos manterão informados de como você se sente e quão bem você é capaz de fazer suas atividades de vida diária. Responda cada questão marcando a resposta como indicado.

NOTA: O inventário foi preenchido pelo avaliador a partir das respostas do paciente.

1. Em geral, você diria que sua saúde é :
. Excelente 1
. Muito boa 2
. Boa 3
. Ruim 4
. Muito Ruim 5

2. **Comparada há um ano**, como você classificaria sua saúde em geral, **agora**?
. Muito melhor agora do que um ano atrás 1
. Um pouco melhor agora do que um ano atrás 2
. Quase a mesma de um ano atrás 3
. Um pouco pior agora do que um ano atrás 4
. Muito pior agora do que um ano atrás 5

3. Os seguintes itens são sobre atividades que você poderia fazer atualmente durante um dia comum. **Devido a sua saúde,** você tem dificuldade para fazer essas atividades? Neste caso, quanto? (circule um número em cada linha)

3. Os seguintes itens são sobre atividades que você poderia fazer atualmente durante um dia comum. **Devido a sua saúde,** você tem dificuldade para fazer essas atividades? Neste caso, quanto? (circule um número em cada linha)

Atividades	Sim. Dificulta muito	Sim. Dificulta pouco	Não. Não dificulta modo algum
a. **Atividades vigorosas**, que exigem muito esforço, tais como correr, levantar objetos pesados, participar em esportes árduos	1	2	3
b. **Atividades moderadas,** tais como mover uma mesa , passar aspirador de pó, jogar bola, varrer a casa	1	2	3
c. Levantar ou carregar mantimentos	1	2	3
d. Subir **vários** lances de escada	1	2	3
e. Subir **um lance** de escada	1	2	3
f. Curvar-se , ajoelhar-se ou dobrar-se	1	2	3
g. Andar **mais de 1 quilômetro**	1	2	3
h. Andar **vários quarteirões**	1	2	3
i. Andar **um** quarteirão	1	2	3
j. Tomar banho ou vestir-se	1	2	3

4. Durante as **últimas 4 semanas**, você teve algum dos seguintes problemas com o seu trabalho ou com alguma atividade diária regular, **como consequência de sua saúde física?** (circule uma em cada linha)

	Sim	Não
a. Você diminuiu **a quantidade de tempo** que se dedicava ao seu trabalho ou a outras atividades?	1	2
b. Realizou **menos tarefas do** que você gostaria ?	1	2
c. Esteve **limitado n**o seu tipo trabalho ou em outras atividades?	1	2
d. Teve **dificuldade** de fazer seu trabalho ou outras atividades (p. ex: necessitou de um esforço extra) ?	1	2

5. Durante as últimas 4 semanas, você teve algum dos seguintes problemas com o seu trabalho ou outra atividade regular diária, como consequência de algum problema emocional (como sentir-se deprimido ou ansioso)? (circule uma em cada linha)

	Sim	Não
a. Você diminuiu **a quantidade de tempo** que se dedicava ao seu trabalho ou a outras atividades?	1	2
b. Realizou **menos tarefas** do que você gostaria ?	1	2
c. Não trabalhou ou não fez qualquer das atividades com tanto **cuidado** como geralmente faz ?	1	2

6. Durante as **últimas 4 semanas**, de que maneira sua saúde física ou problemas emocionais interferiram nas suas atividades sociais normais, em relação a família, vizinhos , amigos ou em grupo? (circule uma)

. De forma nenhuma 1
. Ligeiramente 2
. Moderadamente 3
. Bastante 4
. Extremamente 5

7. Quanta dor **no corpo** você teve durante as **últimas 4 semanas?**

. Nenhuma 1
. Muito leve 2
. Leve 3
. Moderada 4
. Grave.. .5
. Muito grave.. 6

8. Durante as **últimas 4 semanas**, quanto a dor interferiu com o seu trabalho normal (incluindo tanto o trabalho, fora de casa e dentro de casa)?

. De maneira alguma.. 1
. Um pouco 2
. Moderadamente... 3
. Bastante... 4
. Extremamente.. 5

9. Estas questões são sobre como você se sente e como tudo tem acontecido com você durante as **últimas 4 semanas**. Para cada questão, por favor dê uma resposta que mais se aproxime da maneira como você se sente. Em relação **as últimas 4 semanas.** (circule um número para cada linha)

	Todo tempo	A maior parte do tempo	Uma boa parte do tempo	Alguma parte do tempo	Uma pequena parte do tempo	Nunca
a. Quanto tempo você tem se sentido cheio de vigor, cheio de vontade, cheio de força?	1	2	3	4	5	6
b. Quanto tempo você tem se sentido uma pessoa muito nervosa?	1	2	3	4	5	6
c. Quanto tempo você tem se sentido tão deprimido que nada pode animá-lo?	1	2	3	4	5	6
d. Quanto tempo você tem se sentido calmo ou tranquilo?	1	2	3	4	5	6
e. Quanto tempo você tem se sentido com muita energia?	1	2	3	4	5	6
f. Quanto tempo você tem se sentido desanimado e abatido?	1	2	3	4	5	6
g. Quanto tempo você tem se sentido esgotado?	1	2	3	4	5	6
h. Quanto tempo você tem se sentido uma pessoa feliz?	1	2	3	4	5	6
i. Quanto tempo você tem se sentido cansado?	1	2	3	4	5	6

10. Durante as últimas **4 semanas**, quanto do seu tempo a sua **saúde física ou problemas emocionais** interferiram com as suas atividades sociais (como visitar amigos, parentes, etc.)?

. Todo o tempo.. 1
. A maior parte do tempo 2
. Alguma parte do tempo..3
. Um pequena parte do tempo............ 4
. Nenhuma parte do tempo 5

11. O quanto **verdadeiro** ou **falso** é **cada** uma das afirmações para você?

	Definitivamente verdadeiro	A maioria das vezes verdadeiro	Não sei	A maioria das vezes falsa	Definitivamente falsa
a. Eu costumo adoecer um pouco mais facilmente que as outras pessoas	1	2	3	4	5
b. Eu sou tão saudável quanto qualquer pessoa que eu conheço	1	2	3	4	5
c. Eu acho que a minha saúde vai piorar	1	2	3	4	5
d. Minha saúde é excelente	1	2	3	4	5

BDI

Data: ____________

Nome: ______________________ Estado Civil: __________ Idade: ________ Sexo: ________

Ocupação: ______________________ Escolaridade: ______________________

Este questionário consiste em 21 grupos de afirmações. Depois de ler cuidadosamente cada grupo, faça um círculo em torno do número (0, 1, 2 ou 3) próximo à afirmação, em cada grupo, que descreve **melhor** a maneira que você tem se sentido na **última semana, incluindo hoje**. Se várias afirmações num grupo parecerem se aplicar igualmente bem, faça um círculo em cada uma. **Tome o cuidado de ler todas as afirmações, em cada grupo, antes de fazer a sua escolha.**

1
0 Não me sinto triste.
1 Eu me sinto triste.
2 Estou sempre triste e não consigo sair disto.
3 Estou tão triste ou infeliz que não consigo suportar.

2
0 Não estou especialmente desanimado quanto ao futuro.
1 Eu me sinto desanimado quanto ao futuro.
2 Acho que nada tenho a esperar.
3 Acho o futuro sem esperança e tenho a impressão de que as coisas não podem melhorar.

3
0 Não me sinto um fracasso.
1 Acho que fracassei mais do que uma pessoa comum.
2 Quando olho para trás, na minha vida, tudo o que posso ver é um monte de fracassos.
3 Acho que, como pessoa, sou um completo fracasso.

4
0 Tenho tanto prazer em tudo como antes.
1 Não sinto mais prazer nas coisas como antes.
2 Não encontro um prazer real em mais nada.
3 Estou insatisfeito ou aborrecido com tudo.

5
0 Não me sinto especialmente culpado.
1 Eu me sinto culpado grande parte do tempo.
2 Eu me sinto culpado na maior parte do tempo.
3 Eu me sinto sempre culpado.

6
0 Não acho que esteja sendo punido.
1 Acho que posso ser punido.
2 Creio que vou ser punido.
3 Acho que estou sendo punido.

7
0 Não me sinto decepcionado comigo mesmo.
1 Estou decepcionado comigo mesmo.
2 Estou enojado de mim.
3 Eu me odeio.

8
0 Não me sinto de qualquer modo pior que os outros.
1 Sou crítico em relação a mim por minhas fraquezas ou erros.
2 Eu me culpo sempre por minhas falhas.
3 Eu me culpo por tudo de mal que acontece.

9
0 Não tenho quaisquer idéias de me matar.
1 Tenho idéias de me matar, mas não as executaria.
2 Gostaria de me matar.
3 Eu me mataria se tivesse oportunidade.

10
0 Não choro mais que o habitual.
1 Choro mais agora do que costumava.
2 Agora, choro o tempo todo.
3 Costumava ser capaz de chorar, mas agora não consigo, mesmo que o queira.

11
0 Não sou mais irritado agora do que já fui.
1 Fico aborrecido ou irritado mais facilmente do que costumava.
2 Agora, eu me sinto irritado o tempo todo.
3 Não me irrito mais com coisas que costumavam me irritar.

12
0 Não perdi o interesse pelas outras pessoas.
1 Estou menos interessado pelas outras pessoas do que costumava estar.
2 Perdi a maior parte do meu interesse pelas outras pessoas.
3 Perdi todo o interesse pelas outras pessoas.

13
0 Tomo decisões tão bem quanto antes.
1 Adio as tomadas de decisões mais do que costumava.
2 Tenho mais dificuldades de tomar decisões do que antes.
3 Absolutamente não consigo mais tomar decisões.

________ Subtotal da Página 1

CONTINUAÇÃO NO VERSO

"Traduzido e adaptado por permissão de The Psychological Corporation, U.S.A. Direitos reservados ®1991, a Aaron T. Beck.
Tradução para a língua portuguesa. Direitos reservados ®1993 a Aaron T. Beck. Todos os direitos reservados."

Tradução e adaptação brasileira, 2001, Casa do Psicólogo® Livraria e Editora Ltda.
BDI é um logotipo da Psychological Corporation.

14 0 Não acho que de qualquer modo pareço pior do que antes.
1 Estou preocupado em estar parecendo velho ou sem atrativo.
2 Acho que há mudanças permanentes na minha aparência, que me fazem parecer sem atrativo.
3 Acredito que pareço feio.

15 0 Posso trabalhar tão bem quanto antes.
1 É preciso algum esforço extra para fazer alguma coisa.
2 Tenho que me esforçar muito para fazer alguma coisa.
3 Não consigo mais fazer qualquer trabalho.

16 0 Consigo dormir tão bem como o habitual.
1 Não durmo tão bem como costumava.
2 Acordo 1 a 2 horas mais cedo do que habitualmente e acho difícil voltar a dormir.
3 Acordo várias horas mais cedo do que costumava e não consigo voltar a dormir.

17 0 Não fico mais cansado do que o habitual.
1 Fico cansado mais facilmente do que costumava.
2 Fico cansado em fazer qualquer coisa.
3 Estou cansado demais para fazer qualquer coisa.

18 0 O meu apetite não está pior do que o habitual.
1 Meu apetite não é tão bom como costumava ser.
2 Meu apetite é muito pior agora.
3 Absolutamente não tenho mais apetite.

19 0 Não tenho perdido muito peso se é que perdi algum recentemente.
1 Perdi mais do que 2 quilos e meio.
2 Perdi mais do que 5 quilos.
3 Perdi mais do que 7 quilos.

Estou tentando perder peso de propósito, comendo menos: Sim______Não______

20 0 Não estou mais preocupado com a minha saúde do que o habitual.
1 Estou preocupado com problemas físicos, tais como dores, indisposição do estômago ou constipação.
2 Estou muito preocupado com problemas físicos e é difícil pensar em outra coisa.
3 Estou tão preocupado com meus problemas físicos que não consigo pensar em qualquer outra coisa.

21 0 Não notei qualquer mudança recente no meu interesse por sexo.
1 Estou menos interessado por sexo do que costumava.
2 Estou muito menos interessado por sexo agora.
3 Perdi completamente o interesse por sexo.

__________ Subtotal da Página 2

__________ Subtotal da Página 1

__________ Escore Total.

TRAIL MAKING

PART A

AMOSTRA

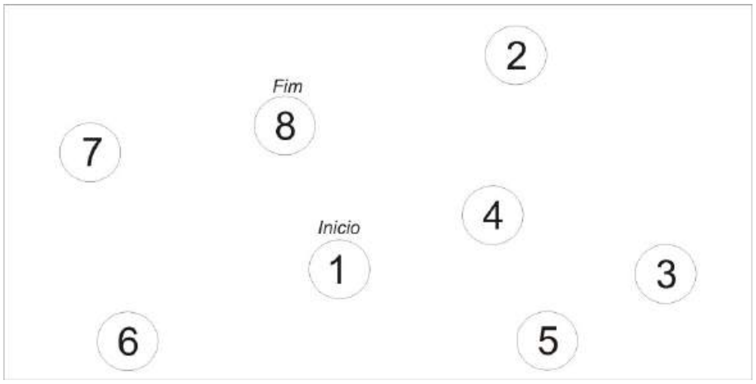

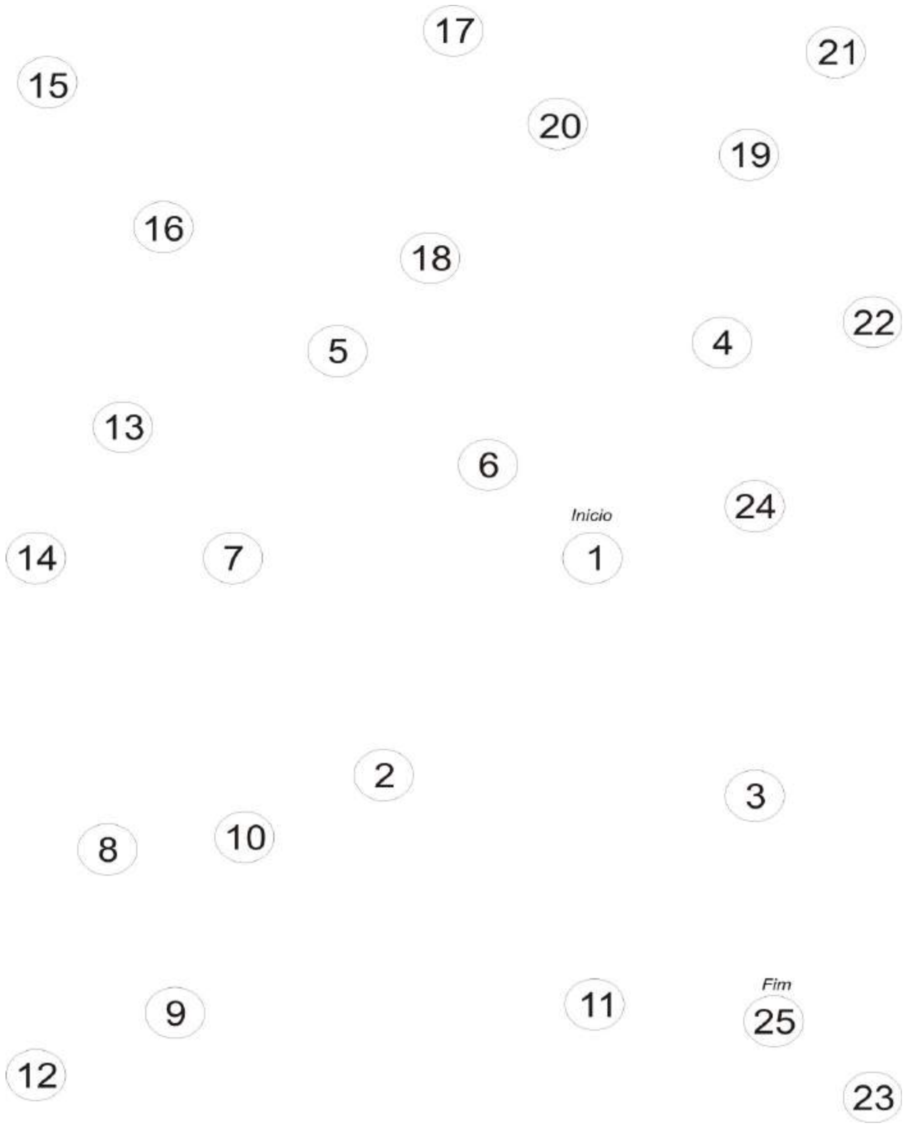
17
21
15
20
19
16
18
22
5
4
13
6
Inicio
24
14
7
1
2
3
10
8
Fim
11
25
9
12
23

TRAIL MAKING

PART B

AMOSTRA

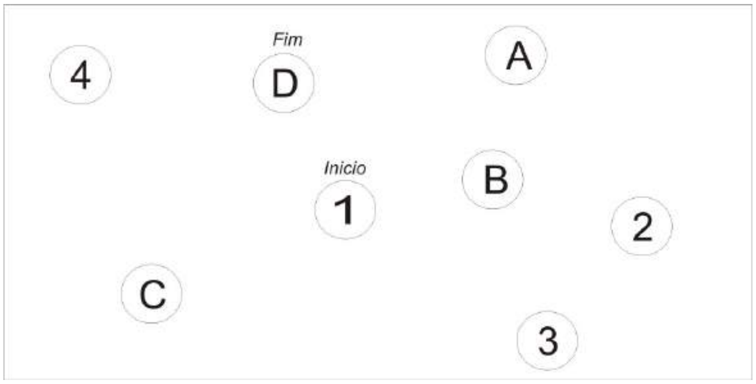

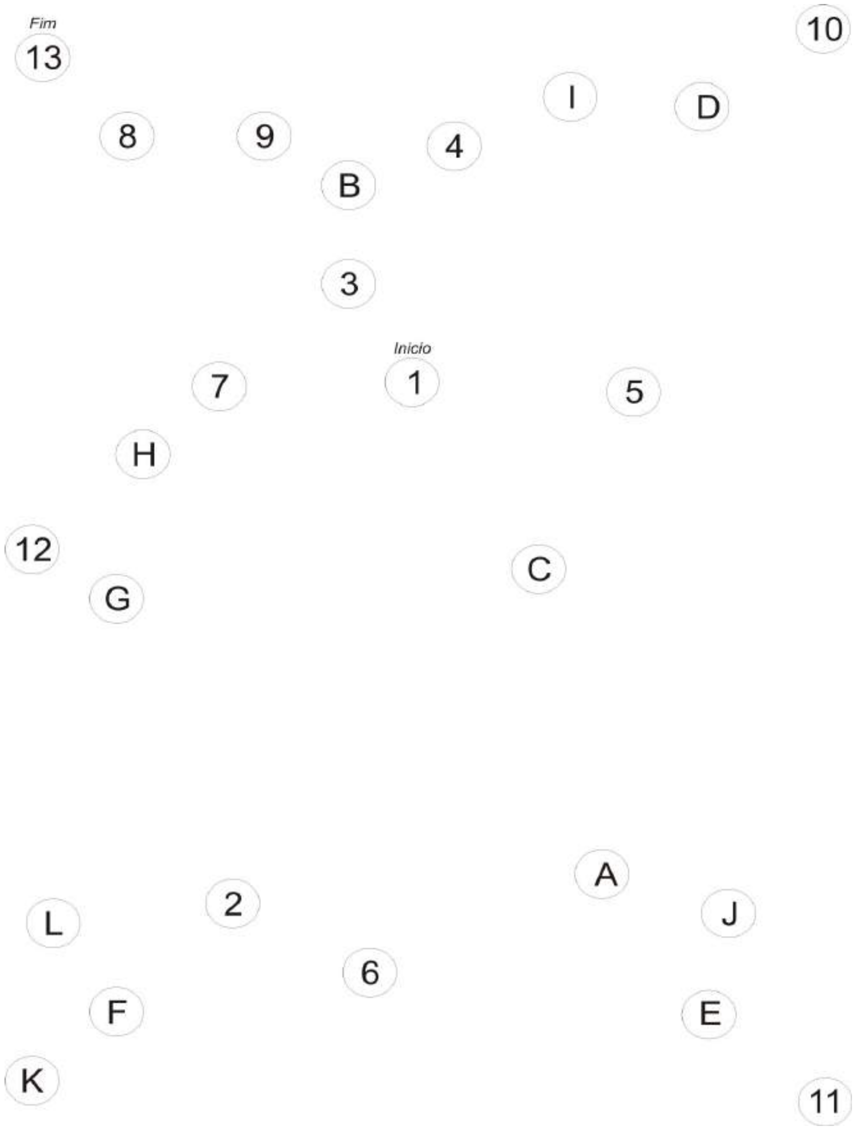
Fim
13
10
I
D
8
9
4
B
3
Inicio
1
7
5
H
12
C
G
A
2
J
L
6
F
E
K
11

Lâmina 1

Azul	Azul	Amarelo	Verde	Vermelho	Azul	Amarelo	Amarelo	Verde	Amarelo
Vermelho	Vermelho	Verde	Vermelho	Verde	Vermelho	Azul	Azul	Verde	Vermelho
Amarelo	Verde	Amarelo	Amarelo	Verde	Vermelho	Vermelho	Verde	Amarelo	Vermelho
Azeul	Amarelo	Azeul	Azul	Amarelo	Verde	Azul	Azul	Verde	Vermelho

Lâmina 2

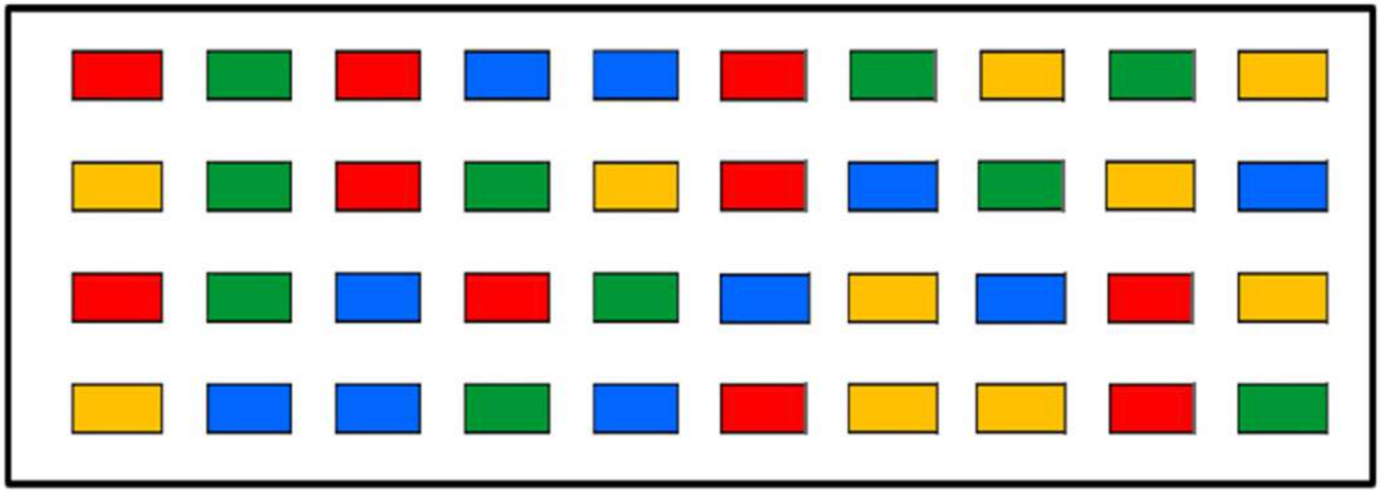

Lâmina 3

Vermelho	Verde	Amarelo	Verde	Amarelo	Vermelho	Vermelho	Azul	Amarelo	Vermelho
Vermelho	Amarelo	Azul	Amarelo	Vermelho	Verde	Verde	Azul	Amarelo	Azul
Azul	Verde	Azul	Azul	Verde	Verde	Azul	Vermelho	Amarelo	Amarelo
Verde	Amarelo	Verde	Verde	Azul	Verde	Vermelho	Amarelo	Vermelho	Vermelho

Effects of dexamethasone on the incidence of postoperative cognitive dysfunction

Authors' names (academic degrees), and affiliations and Contact information:

Livia Stocco Sanches Valentin
Title: Neuropsy; Ph.D
E-mail: lssv@usp.br
Affiliation: Discipline of Anaesthesiology, LIM 8 – Laboratory of Anaesthesiology, Faculdade de Medicina da Universidade de Sao Paulo, Brazil
Role: This author helped conduct the study, analyse the data, and write the manuscript.
Conflicts: Livia Stocco Sanches Valentin reported no conflicts of interest.
Attestation: Livia Stocco Sanches Valentin has seen the original study data, reviewed the analysis of the data, approved the final manuscript, and is the author responsible for archiving the study files.

Ricardo S. Pietrobon
Title: MD; Ph.D
E-mail: rpietro@duke.edu
Affiliation: Researcher on Research Group, Duke University Health System, Durham, North Carolina, USA, and Department of Surgery, Duke University Health System, Durham, NC, USA
Role: This author helped design the study, the statistical analysis and processing of data and drafting of the manuscript.
Conflicts: Ricardo Pietrobon reported no conflicts of interest.
Attestation: Ricardo Pietrobon has seen and reviewed the statistical analysis, helped process the data and draft the manuscript and approved the final manuscript.

Andre P. Schmidt
Title: MD, Ph.D
E-mail: aschmidt@ufrgs.br
Affiliation: Department of Biochemistry, ICBS, Federal University of Rio Grande do Sul, Porto Alegre (UFRGS) - RS – Brazil and Department of Anaesthesia and Perioperative Medicine, Hospital de Porto Alegre (HCPA), Porto Alegre, RS, Brazil
Role: This author helped in the analysis data of the Biomarkers and processing of data and drafting of the manuscript.
Conflicts: Andre P. Schmidt reported no conflicts of interest.
Attestation: André P Schmidt has seen and reviewed the data analysis of the Biomarkers, helped process the data and drafts the manuscript and approved the final manuscript.

Jean P. Oses

Title: MSc, Ph.D
E-mail: jean.pierre.oses@gmail.com
Affiliation: Program of Post-Graduation in Health and Behaviour, Centre of Sciences of the Life and Health, Catholic University of Pelotas, Pelotas, RS, Brazil.
Role: This author helped in the analysis data of the Biomarkers and processing of data and drafting of the manuscript.
Conflicts: Jean P. Oses reported no conflicts of interest.
Attestation: Jean P. Oses has seen and reviewed the data analysis of the Biomarkers, helped process the data and drafts the manuscript and approved the final manuscript.

Luis V. Portela
Title: MSc, Ph.D
E-mail: roskaportela@gmail.com
Affiliation: Department of Biochemistry, ICBS, Federal University of Rio Grande do Sul (UFRGS), Porto Alegre, RS, Brazil.
Role: This author helped in the analysis data of the Biomarkers and processing of data and drafting of the manuscript.
Conflicts: Luis V. Portela reported no conflicts of interest.
Attestation: Luis V. Portela has seen and reviewed the data analysis of the Biomarkers, helped process the data and drafts the manuscript and approved the final manuscript.

Diogo O. Souza
Title: MD, Ph.D
E-mail: diogo@ufrgs.br
Affiliation: Department of Biochemistry, ICBS, Federal University of Rio Grande do Sul (UFRGS), Porto Alegre, RS, Brazil.
Role: This author helped in the analysis data of the Biomarkers and processing of data and drafting of the manuscript.
Conflicts: Diogo O. Souza reported no conflicts of interest.
Attestation: Diogo O. Souza has seen and reviewed the data analysis of the Biomarkers, helped process the data and drafts the manuscript and approved the final manuscript.

Karen C. Nielsen
Title: MD
E-mail: niels006@mc.duke.edu
Affiliation: Department of Anaesthesiology, Duke University Medical Centre, Durham, North Carolina, USA.
Role: This author helped in the design and preparation of the study.
Conflicts: Karen C. Nielsen reported no conflicts of interest.
Attestation: Karen C. Nielsen has seen and reviewed the statistical analysis and helped process the data and draft the manuscript, and approved the final manuscript.

João Ricardo Nickenig Vissoci
Title: Psy; MS
E-mail: joaovissoci@gmail.com

Affiliation: Researcher on Research Group, Duke University Health System, Durham, North Carolina, USA, and Faculdade Inga, Medicine Department Maringa, Parana, Brazil
Role: This author helped in the statistical analysis and processing of data and drafting of the manuscript.
Conflicts: Joao Ricardo Nickenig Vissoci reported no conflicts of interest.
Attestation: Joao Ricardo Nickenig Vissoci has seen and reviewed the statistical analysis and helped process the data and draft the manuscript, and approved the final manuscript.

Vinícius Fernando da Luz
Title: MD
E-mail: viniciusfernando@yahoo.com.br
Affiliation: Discipline of Anaesthesiology, LIM 8 – Laboratory of Anaesthesiology, Faculdade de Medicina da Universidade de Sao Paulo, Brazil
Role: This author helped in recruitment for the study and the data analysis.
Conflicts: Vinícius Fernando da Luz reported no conflicts of interest.
Attestation: Vinícius Fernando da Luz has seen the original study data, reviewed the data analysis, approved the final manuscript, and is the author responsible for archiving the study files.

Leticia Maria de Araujo de Souza Trintoni

Title: Psy
E-mail: leticiatrintoni@gmail.com

Affiliation: Discipline of Anaesthesiology, LIM 8 – Laboratory of Anaesthesiology, Faculdade de Medicina da Universidade de Sao Paulo, Brazil
Role: This author helped in recruitment and assessment neuropsychological for the study and the data analysis from cognitive tests.
Conflicts: Leticia de Araujo de Souza Trintoni reported no conflicts of interest.
Attestation: Leticia de Araujo de Souza Trentoni has seen the neuropsychological data from cognitive tests, reviewed the data analysis, approved the final manuscript, and is the author responsible for archiving the study files.

Katia Osternack Pinto
Title: Neuropsy; Ph.D
E-mail: katiadip@uol.com.br
Affiliation: Currently the author is not affiliated with any academic institution
Role: This author helped in the preparation of the study, from the initial design phase to the construction of the specific battery of neuropsychological tests.
Conflicts: Katia Osternack Pinto reported no conflicts of interest.
Attestation: Katia Osternack Pinto was involved in the preparation of the study, from the initial design phase to the construction of the specific battery of neuropsychological tests, and approved the final manuscript.

Maria Jose Carvalho Carmona
Title: MD; Ph.D
Affiliation: Departments of Anaesthesia, LIM 8 – Laboratory of Anaesthesiology, Faculdade de Medicina da Universidade de Sao Paulo, Brazil
E-mail: maria.carmona@incor.usp.br
Role: This author helped design the study, analyse the data, and write the manuscript.
Conflicts: Maria José Carvalho Carmona reported no conflicts of interest.
Attestation: Maria Jose Carvalho Carmona has seen the original study data, reviewed the analysis of the data, approved the final manuscript, and is the author responsible for guiding the fellows of the postgraduate program and archiving the study files.

Corresponding Author:

Livia Stocco Sanches Valentin

Discipline of Anaesthesiology, LIM 08/Anestesiologia – Faculdade de Medicina da Universidade de São Paulo,

Avenida Dr. Eneas de Carvalho Aguiar, 155, 8º andar, Prédio dos Ambulatórios -Bloco 3- Divisão de Anestesia

Bairro Cerqueira Cesar

CEP 05403-001 Sao Paulo, SP, Brazil

Phone: 55-1192640350

FAX: 55-112661-7947

E-mail: lssv@usp.br

Effects of dexamethasone on the incidence of postoperative cognitive dysfunction

INTRODUCTION: Postoperative cognitive dysfunction (POCD) is frequently reported following general anesthesia, especially in elderly patients and after cardiac surgery. The International Study of Postoperative Cognitive Dysfunction (ISPOCD 1 and 2) demonstrated that this adverse event also affects up to 26% of geriatric patients one week after non-cardiac surgery under general anesthesia, and 10% of them 3 months after surgery[1, 2]. In elderly patients POCD increased the risk of comorbidities in the first year after surgery. However, there is no consensus about the factors related to the induction or prevention of cognitive dysfunction in surgical patients. Besides age, postoperative cognitive function may be compromised by hypoxia, hypotension, anesthetics, surgical procedures and external factors such as quality of life[3]. The depth of anesthesia is associated with neuropsychological changes, from mental confusion to memory loss, decreasing the level of consciousness, flexibility and the ability to reason and are defined as a transitory change in state of consciousness, also known as delirium.

Surgery can result in a complex system response, which includes neuroinflammation and may directly affect patient outcome[4]. Data support the concept that inflammation and brain injury are possible pathogenic mechanism for POCD. Neuron specific enolase (NSE) is primarily located in the cytoplasm of neurons and involved in increasing neuronal chloride levels during onset of neuronal activity[5, 6]. Additionally, in the early stages of brain injury, glial cells are activated and S100β is released into blood circulation[7].

Corticosteroids such as dexamethasone are often used as adjuvant drugs in anesthesia to mitigate the side effects of anesthesia and surgery such as pain, vomiting and fatigue, all of which are physical symptoms that compromise cognitive functioning[8, 9]. The use of dexamethasone before surgery or its association with the deep of anesthesia were not investigated as possible protective factors against cognitive impairment in non-cardiac surgery.

On the assumption that POCD might be also secondary to an inflammatory process and depends on the depth of anesthesia, we hypothesized that the use of anti-inflammatory drugs could reduce the incidence of this adverse event.

This study aimed to evaluate the effect of dexamethasone and the depth of anesthesia on the incidence of postoperative cognitive dysfunction in elderly patients undergoing non-cardiac surgery under general anesthesia.

MATERIALS AND METHODS

Study design and subject enrolment

The study was designed as a randomized, double blind and prospective clinical trial. After approval by the Institutional Ethical Committee and receiving written informed consent, 140 patients (aged 60 years or older) who were candidates for elective non-cardiac surgery in a university hospital between January 2011 and February 2012 were enrolled in the study and the neuropsychological assessment was completed in March 2013. Inclusion criteria were as follows: candidates for non-cardiac surgery under general anesthesia that was not expected to exceed 6 hours, no history of brain disease, dementia or other psychiatric disorders that affect cognition, literate and no continuous use corticosteroids, antidepressants or opioids preoperatively.

The mental state was preliminarily assessed using the Mini Mental State Examination (MMSE), which is validated for the Brazilian population as a brief screening instrument designed to measure and quantify the global cognitive state, assessing reasoning, spatial-temporal orientation, memory and schooling. The test uses for the Brazilian people a cut-off of 18 points for subjects who have completed up to 4 years of formal academic study and 23 points for individuals with higher levels of education. Patients were excluded if they had an MMSE score below 18 or 23 in accordance with educational level[10, 11].

Preoperative evaluation and neuropsychological assessment

Demographic data were analyzed, including education, marital status, occupation, and current medication. Signs and symptoms of depression were assessed preoperatively using the Beck Depression Inventory (BDI), which consists of 21 questions that explore depressive symptoms on a scale of 0 to 4, where zero represents no symptoms and four is the maximum symptomatology. A total of 14 points was considered indicative of the presence of moderate depressive symptoms[12]. The quality of life was investigated using the Short Form Health Survey (SF-36) to investigate health-related conditions, activities of daily living, productivity, emotional problems, relationships, and motivation, and one question about the perception of the patients itself[13].

The Telephone Interview for Cognitive Status (TICS) was used as a standardized test to assess neuropsychological functioning when assessing cognitive skills. This test was used when screening personally was impractical or when patients were unable to attend the clinic. This test consists of a structured interview with 11 items that assess the skills of spatial and temporal orientation, mental control, memory, general information, language and calculations[14].

The specific neuropsychological battery test, based on the International Studies of POCD 1 and 2 translated and adapted for the specific studied population, was performed one day before surgery (baseline) and on the 3rd, 7th, 21st, 90th and 180th postoperative day. The cognitive functions assessed were memory, attention and executive function in aspects such as strategy, elaboration of thought and inhibiting the control of actions. Tests used for the evaluation of these skills are redundant, especially for the evaluation of attention.

Memory was evaluated using the Rey-Auditory Verbal Learning Test (RAVLT), which consists of a list of 15 words to be memorized and recalled on three successive attempts, with delayed recall after 15 to 25 minutes, evaluating the number of words recalled and the number of errors made for each presentation[15].

The Symbol Digit Modified Test (SDMT) was applied for the evaluation of short-term memory, visual search skill and attention. This is a graphical task where the individual has to fill in symbols exemplified in the spaces below the corresponding number within 180 seconds. The result is measured as the number of symbols drawn and the number of errors[15].

Attention was also evaluated using the Trail Making Test (TMT) for the assessment of selective and alternating attention. In part A of the test, the subject must draw lines connecting consecutively numbered circles. In part B, the subject must draw lines connecting circles alternately with letters and numbers in a sequence. The result is measured as time and trail errors[15].

Criteria for POCD diagnosis

TICS values were used to evaluate the evolution of general cognition postoperatively and were applied in every phase’s pre and postoperatively. For the diagnosis of POCD,

a composite cognitive index was established defined by the occurrence of cognitive impairment in TICS and at least 1 of 8 possible deficits of the others neuropsychologist tests. Changes in neuropsychological tests have been compared with the results of tables of normative subjects matched for age, gender and formal education[16].

Assessment of serum levels of S100 β and NSE

Serum levels of S100β protein and NSE were obtained from venous blood samples collected before anesthesia induction and 12 hours after surgery. Samples were placed in dry tubes and centrifuged; serum was removed and stored to -80 ºC until the analysis and S100β protein was measured by a commercially available S100β enzyme-linked immune absorbent assay, (ELISA) kit (Diasorin, Italy). A quantitative monoclonal 2-site ELISA micro plate assay in which the last antibody added to the reaction system is labeled with peroxidase. After the addition of a peroxidase substrate, the reaction produces a final color product read in a spectrophotometer. S100β levels were expressed as micrograms per milliliter. NSE was measured using an electrochemiluminescent assay. It consists in a double-sandwich assay that uses an anti-NSE antibody, labeled with ruthenium, which is the luminescent molecule. Reactions and quantification was performed with Elecsys-2010 (Roche Diagnostics Corporation, USA). NSE levels were expressed as micrograms per milliliter[17].

Randomization and anesthesia technique

Immediately before anesthesia induction patients were randomized to receive or not 8 mg of dexamethasone and evaluated under intention to treat (ITT) analysis. According to the bispectral index (BIS), they were allocated into a superficial anesthesia subgroup (BIS between 46–55) or deep anesthesia subgroup (BIS between 35–45). The randomization was performed using the website

http://www.randomizer.org/form.htm, recorded on paper and stored in sealed opaque envelopes to be opened in the operating room. Anesthesiologists were aware of the allocated arm, but patients, neuropsychologists, laboratory technicians and outcome assessors involved in the study were kept blinded to the group to which each patient belonged. The study groups were identified only after the evaluation of all patients and statistical analysis. All the study was blinded and randomized, from intervention with dexamethasone to technical and anesthetic depth.

In the operating room, standard monitoring included continuous registration of electrocardiogram at DII, pulse oximetry, non-invasive blood pressure, tidal CO_2 and bispectral index. General anesthesia was induced with propofol (2–3 μg/kg), fentanyl (0.05 μg/kg) and cisatracurium (0.10 mg/kg), Maintenance of anesthesia was performed with propofol target-controlled infusion, according to Schneider's pharmacokinetic model remifentanil (0.10 to 1.0 μg/kg/ min) or fentanyl (intermittent doses of 2–3 μg/kg) according to anesthesiologist judgment, plus cisatracurium in supplemental doses. Hypnosis level was approached according to BIS, using superficial anesthesia (BIS between 46 and 55) or deep anesthesia (BIS between 35 and 45); both levels are acceptable for general anesthesia and are currently used by anesthesiologists. Glycaemia was controlled perioperatively; after anesthesia induction all patients had a first glycaemic check and insulin was given when blood glucose concentrations exceeded 180 mg/dL. After extubation, patients were transferred to the post-anesthesia care unit. The control of postoperative pain was conducted following institutional protocols.

A quality analysis of the results of the assessment cognitive and other data in this randomized clinical trial was conducted using the CONSORT criteria (www.consort-

statement.org), as well as for greatest impact according to consolidated Standards of Reporting Trials criteria.

Statistical analyses

Based on an incidence of postoperative cognitive dysfunction of 40% for elderly patients and the group receiving dexamethasone decreased 20% in the POCD considered clinically relevant at 21st, a maximum error of 5%, and a confidence interval and desired statistical power of 80%, we estimated that 138 patients were required. Results of neuropsychological tests were described according to the groups and times of applying by the mean and standard deviation and compared between the groups and the times by using Generalized Estimation Equation (GEE). Changes in neuropsychological tests have been compared with the results of tables of normative subjects matched for age, gender and formal education, using Z-score analysis[18]. The maximum likelihood approaches were used in the case of missing data, which did not exceed 12% of the total data collected, in general linear mixed modelling, imputation increases statistical accuracy[19, 20]. Neuropsychological tests were described according groups and application times with mean and standard deviation of use and compared between groups and moments with the use of generalized estimating equations with matrix of correlations autoregressive order 1 between times, with marginal distribution Normal and identity link function. For models that showed statistical significance analysis was followed by Bonferroni multiple comparisons to find out, which groups or moments occur, differences in scales. For comparison of the biomarkers between groups and time points were used generalized estimating equations with matrix of correlations autoregressive order 1 between times, with marginal gamma distribution and identity link function, followed by Bonferroni multiple comparisons to learn which

groups or moments occur differences in biomarkers. Measures changes in biomarkers were created (post-anesthesia - pre) and neuropsychological tests in 21 days and 180 days (21 days - before and 180 days - pre respectively) and Pearson's correlations were calculated between the changes in biomarkers and changes in neuropsychological, separate tests for the groups and the total number of patients to evaluate the correlation between the changes in biomarkers with the variation of neuropsychological tests. Described genotypes and alleles second groups with use of absolute and relative frequencies and checked for association with the use of likelihood ratio tests. The variation in neuropsychological tests described in the second allele using summary measured and compared variations on the tests between alleles using ANOVA followed by Bonferroni multiple comparisons when necessary. The tests were done at 5% significance level. All tests were carried out at a significance level of 5%. Statistical calculations were performed using SPSS12 and GraphPad Prism version 6.00 for Mac (GraphPad Software, La Jolla California USA www.graphpad.com).

RESULTS

Three hundred and six patients were recruited for elective non-cardiac surgery under general anesthesia but 83 patients were excluded because they are not in the inclusion criteria or declined to participate in the study or for other reasons not justified by the patient. Two hundred and twenty-three patients were assessed with the MMSE cognitive screening test and 27 patients were excluded after failing to normative score (≤ 18 or ≤ 23 points) according education levels. One hundred and ninety-six patients were assessed using neuropsychological battery tests adapted from ISPOCD and 140 patients were ultimately randomized and evaluated under intention to treat (ITT) analysis up to 180th postoperative day (Fig. 1). There was no statistical difference

between groups concerning baseline and demographic data (Table 1). The intraoperative data are shown in Table 2, revealing a statistical difference in blood glucose levels during the fourth hour of surgery, with increased glucose levels in the groups receiving dexamethasone ($p=0.02$). No reports of intraoperative recall were related. The mean bispectral index values were comparable between groups at baseline but differed according to anesthesia procedure (Table 2). The results for global cognitive function evaluated by TICS (Table 3) showed that the highest incidence of POCD on the 3rd postoperative day was observed in the deep anesthesia group (68.2%, $p<0.0001$). After 6 months the same group again showed the highest incidence of POCD, although this was lower than on the 3rd postoperative day (25.3%, $p<0.0001$). The group receiving superficial anesthesia and dexamethasone at anesthesia induction showed the lowest incidence of POCD during the study. On the 21st, 90th and 180th days this group showed no changes in global cognitive function according to TICS. The mean results of the TICS for this group were above the cut-off point (27 ± 3) for the test (32.5 ± 1.4; 33 ± 3.7; 32.5 ± 1.6 respectively).

In terms of memory function (immediate and delayed) assessed via RAVLT, there was a significant difference during postoperative phases between groups for immediate memory ($p=0.0002$) and delayed recall ($p=0.001$). In terms of immediate memory, the highest incidence of dysfunction was observed on the 180th day in the deep anesthesia group (74.6%) and then the superficial anesthesia group (53.4%). For delayed recall the highest incidence was 42.5% on the 21st day in the deep anesthesia group. The superficial anesthesia plus dexamethasone group showed no dysfunction for delayed recall. For the Selection Attention Function assessed by the TMT in part A, dysfunction was detected in 77.5% ($p<0.0001$) of patients in the deep anesthesia group

on the 180th day. The same group showed impairment in 42.5% of cases for the ability to toggle attention, assessed by TMT part B, on the 180th day (p=0.001). For executive function, visual and motor skills, assessed by the SDMT, the highest incidence of POCD was presented by the deep anesthesia plus dexamethasone group on the 21st day (24.3% of patients), followed by the deep anesthesia group in twenty-two and a half per cent. The groups receiving dexamethasone showed no executive dysfunction on the 180th postoperative day (Table 3).

Based on the SF-36 (Table 3), all groups showed good general health at the end of the study (p = 0.09). On the 180th day, the superficial anesthesia group had a lower score on the mental health subscale (66.5±14.6) and 12% of subjects in this group had below average results (50±10). All groups showed absence of depressive symptoms in the preoperative assessment. At the end of the study group who received superficial anesthesia and the dose of dexamethasone showed a lower mean for depressive symptoms (5.8±3.4) assessed by the BDI. At the end of the study the groups that had received dexamethasone showed no depressive symptoms (Table 3).

S100β serum levels were significantly increased 12 hours after surgery in patients submitted to either deep or superficial anesthesia ($p<0.05$). However, this effect was prevented by the administration of dexamethasone. Additionally, postoperative NSE serum levels were significantly reduced in patients receiving dexamethasone regardless anesthetic depth ($p<0.05$) (Table 4).

DISCUSSION

In our study the patients were underwent cognitive testing with multiple tests including the MMSE, the TICS, the RAVLT and Symbol Digit Modified Test. Many of these tests overlap in the cognitive domains evaluated yet the little assessment of other areas

including long-term memory or visuo-spatial testing. This is a not big limitation but a concern regarding to data analysis, thus to avoid duplication of data that may result in overestimation of impairment on cognitive functions, for each test were evaluated only the main functions that the test is designed to evaluate. For example, in TMT-A we only evaluated the selective attention. We also had another concern that was the approach to analysis of the psychometric testing results. On this case the data were evaluated based on normative data and between study groups the risk for overestimation of cognitive end-points were decreased. The patients were classified as having POCD if their test results were lower than the normative data. The frequency of POCD based on this analysis was then tabulated for each treatment group and compared the data between treatment groups. Our study found that patients receiving a single dose of dexamethasone at induction of anesthesia presented a lower incidence of postoperative cognitive dysfunction. When this corticosteroid was associated with superficial anesthesia rather than deep hypnosis their cognitive functions established more rapidly. A better quality of life was also observed in the postoperative period in the dexamethasone groups, as well as a lower incidence of depressive symptoms.

In anesthesia practice, patients with lower postsurgical pain return to daily activities in less time than those who have postoperative injuries, such as pain, nausea and vomiting. Dexamethasone is nowadays commonly used in the perioperative setting by anesthetists, both for its antiemetic properties and its ability to reduce airway swelling and fatigue. Our findings corroborate some of the observations made in other studies on corticosteroids such as the decrease in the postoperative recovery time due to the wellbeing caused by the administration of dexamethasone before, during or after cardiac or general surgery. Although no specific studies have evaluated the possible

effects of dexamethasone on the incidence of POCD in patients undergoing noncardiac surgery, the inhibitory effect of dexamethasone on inflammation could also prevent some undesirable neuropsychological adverse events such as cognitive dysfunction, delirium, and even stroke[8].

In the early stages of brain injury, glial cells are activated, and S100β is released into blood circulation[7]. On the other hand, NSE is primarily located in the cytoplasm of neurons and involved in increasing neuronal chloride levels during onset of neuronal activity[6].

In the present study, S100β serum levels were significantly elevated after surgery regardless anesthetic depth and might represent substantial glial cell damage or physiological responses of astrocytes trying to keep neuronal function and survival. Notably, the administration of dexamethasone prevented the postoperative increase in S100β serum levels, an effect that might indicate some degree of neuroprotection. Additionally, serum levels of NSE were similar between patients submitted to deep or superficial anesthesia and the pre-anesthetic administration of dexamethasone was associated to a significant decrease in serum levels of NSE after surgery. This finding is somewhat difficult to explain, but previous data indicated that a decrease in NSE levels might be related to energy metabolism dysfunctions rather than neuronal degeneration[21]. Altogether, these results indicate some differences regarding time profile between both biochemical markers following noncardiac procedures. Considering our present findings and that S100β protein is highly brain specific, it seems to be more useful as a biochemical marker of brain damage and POCD than NSE.

Although controversy remains, several authors were able to demonstrate a relationship between the degree of cognitive dysfunction and S100β protein[4, 22], which is consistent with our present findings.

The hyperglycemic response associated with both surgical stress and corticosteroids could be a contraindication for the routine use of dexamethasone in surgical patients, especially in diabetic patients. However, in a recent study evaluating the hyperglycemic response to major noncardiac surgery and the added effect of steroid administration in patients with and without diabetes, the authors used the same 8 mg dose of dexamethasone used herein and concluded that denying steroid prophylaxis for postoperative nausea and vomiting for fear of a hyperglycemic response should be reconsidered given the limited effect of steroids on intraoperative blood glucose concentrations[23].

Our study found significant differences between groups, showing that superficial anesthesia reduces the occurrence of POCD or allows more rapid restoration of cognitive function in the postoperative period. Some other studies have shown that superficial anesthesia may benefit the patient and prevent postoperative cognitive dysfunction[24, 25].

Although our study demonstrated the benefit of dexamethasone associated with superficial anesthesia in terms of prevention of cognitive dysfunction, other factors should be investigated. Cognitive reserve, even in adulthood, is an enabling resource for a more independent and high quality life, with elderly age and comorbidities being risk factors for POCD.

Besides the existence of cognitive decline with age, other demographic, clinical, environmental and genetic factors can influence performance on neuropsychological

tests in surgical patients. Some clinical studies have shown that a decline in memory function is more evident in adulthood, especially in the elderly. Cognitive decline is inevitable during the aging process, but this impairment may also be associated with surgery and anesthesia. Although longitudinal research on cognitive decline in the elderly population has shown that differences detected in the performance of daily tasks are associated with age, there are also differences between generations and the degree of interference and social work activities. Moreover, multiple co-morbidities are common in the elderly and may interfere with cognitive performance during the course of neuropsychological assessment[26]. People with a higher educational level tend to be healthier than people with a lower educational level and there are different pathways by which education leads to better health. First, the level of education is highly correlated with other social determinants of health, such as the level of income, employment security, and working conditions. The highest incidence of POCD is generally found in the most elderly patients, those with the lowest educational levels, and those with a history of cognitive decline before anesthesia. It is suggested that other factors compromise cognitive function, beyond the depth of anesthesia. For instance, postoperative analgesia is associated with an increase in quality of life and stability of mood. The mood state can also change mainly when pain and stress factors are involved, such as surgery and postoperative recovery phase.

LIMITATIONS OF THE STUDY

One limitation of the current study was the loss of follow-up for many patients, mainly caused by the socioeconomic status of the sample. Due to their low economic status, some patients were not always able to attend the assessments face to face in the hospital, and many patients were living outside the city, the state or even the country.

To attend appointments, most of these patients needed public transportation, the expenses of which exceeded their family budgets. The lack of communication resources such as home phones or cellular phones also meant that the TICS were not applied in some cases. On the other hand, many neuropsychological tests were not designed to investigate cognitive dysfunction in surgical patients, and many do not detect subtle cognitive deterioration. It has already been shown that people who suffer from adverse social conditions also experience high levels of psychological and physiological stress. It is also important to note the low mean educational level of this study sample.

CONCLUSION

In conclusion, dexamethasone when administered jointly with superficial anesthesia can help to preserve most cognitive functions in non-cardiac surgery. Additional studies could help to define the neuroprotective properties of dexamethasone in the prevention of postoperative cognitive dysfunction in patients undergoing noncardiac surgery.

Short Title: EFFECTS OF DEXAMETHASONE ON POCD

Funding: São Paulo Research Foundation (FAPESP) grant number 09/54233-0 supported this study.

REFERENCES

1. Abildstrom H, Rasmussen LS, Rentowl P, Hanning CD, Rasmussen H, Kristensen PA, et al. Cognitive dysfunction 1-2 years after non-cardiac surgery in the elderly. ISPOCD group. International Study of Post-Operative Cognitive Dysfunction. Acta anaesthesiologica Scandinavica. 2000 Nov;44(10):1246-51.
2. Rasmussen LS, investigators I. Post-operative cognitive dysfunction in the elderly. Acta anaesthesiologica Scandinavica. 2005 Nov;49(10):1573.
3. Jungwirth B, Zieglgansberger W, Kochs E, Rammes G. Anesthesia and postoperative cognitive dysfunction (POCD). Mini reviews in medicinal chemistry. 2009 Dec;9(14):1568-79. 4. Peng L, Xu L, Ouyang W. Role of peripheral inflammatory markers in postoperative cognitive dysfunction (POCD): a meta-analysis. PloS one. 2013;8(11):e79624.
5. Jones EL, Gauge N, Nilsen OB, Lowery D, Wesnes K, Katsaiti E, et al. Analysis of Neuron-Specific Enolase and S100B as Biomarkers of Cognitive Decline Following Surgery in Older People. Dementia and geriatric cognitive disorders. 2012;34(5-6):307-11.
6. Kaiser E, Kuzmits R, Pregant P, Burghuber O, Worofka W. Clinical biochemistry of neuron specific enolase. Clinica chimica acta; international journal of clinical chemistry. 1989 Jul 31;183(1):13-31.
7. Donato R, Cannon BR, Sorci G, Riuzzi F, Hsu K, Weber DJ, et al. Functions of S100 proteins. Current molecular medicine. 2013 Jan;13(1):24-57. PubMed PMID: 22834835.
8. Jakobsson J. Preoperative single-dose intravenous dexamethasone during ambulatory surgery: update around the benefit versus risk. Current opinion in anaesthesiology. 2010 Dec;23(6):682-6.
9. Gomez-Hernandez J, Orozco-Alatorre AL, Dominguez-Contreras M, Oceguera-Villanueva A, Gomez-Romo S, Alvarez Villasenor AS, et al. Preoperative dexamethasone reduces postoperative pain, nausea and vomiting following mastectomy for breast cancer. BMC cancer. 2010;10:692.
10. ALMEIDA PO. MINI EXAME DO ESTADO MENTAL E O DIAGNÓSTICO DE DEMÊNCIA NO BRASIL. Arq Neuropsiquiatr. 1998;56(3-B):605-12.
11. Horton AM, Jr., Alana S. Validation of the Mini-Mental State Examination. The International journal of neuroscience. 1990 Aug;53(2-4):209-12.
12. Gorenstein C, Andrade L. Validation of a Portuguese version of the Beck Depression Inventory and the State-Trait Anxiety Inventory in Brazilian subjects. Brazilian journal of medical and biological research = Revista brasileira de pesquisas medicas e biologicas / Sociedade Brasileira de Biofisica [et al]. 1996 Apr;29(4):453-7.
13. Cruz LN, Fleck MP, Oliveira MR, Camey SA, Hoffmann JF, Bagattini AM, et al. Health-related quality of life in Brazil: normative data for the SF-36 in a general population sample in the south of the country. Ciencia & saude coletiva. 2013 Jul;18(7):1911-21.
14. Lopez OL, Kuller LH. Telephone interview for cognitive status. Neuroepidemiology. 2010;34(1):63-4.

15. Strauss E SE SO. A Compendium of Neuropsychological Tests: Administration, Norms, and Commentary. Oxford University Press. Third Edition;1((1)):1-1225.
16. Moller JT, Cluitmans P, Rasmussen LS, Houx P, Rasmussen H, Canet J, et al. Long-term postoperative cognitive dysfunction in the elderly ISPOCD1 study. ISPOCD investigators. International Study of Post-Operative Cognitive Dysfunction. Lancet. 1998 Mar 21;351(9106):857-61.
17. Böhmer AE OJ, Schmidt AP, Perón CS, Krebs CL, Oppitz PP, D'Avila TT, Souza DO, Portela LV, Stefani MA. Neuron-specific enolase, S100B, and glial fibrillary acidic protein levels as outcome predictors in patients with severe traumatic brain injury. Neurosurgery. 2011;68((6)):1624-30.
18. Canet J, Raeder J, Rasmussen LS, Enlund M, Kuipers HM, Hanning CD, et al. Cognitive dysfunction after minor surgery in the elderly. Acta anaesthesiologica Scandinavica. 2003 Nov;47(10):1204-10.
19. MYERS RW. HANDLING MISSING DATA IN CLINICAL TRIALS: AN OVERVIEW. Drug Information Journal. 2000;34:525-33.
20. Johnson TR, Wiest MM. Generalized linear models with coarsened covariates: a practical Bayesian approach. Psychological methods. 2014 Jun;19(2):281-99.
21. Wiener CD, Jansen K, Ghisleni G, Kaster MP, Souza LD, Lara DR, et al. Reduced serum levels of neuron specific enolase (NSE) in drug-naive subjects with major depression and bipolar disorder. Neurochemical research. 2013 Jul;38(7):1394-8.
22. Heyer EJ, Connolly ES. Serum concentration of S-100 protein in assessment of cognitive dysfunction after general anesthesia in different types of surgery. Acta anaesthesiologica Scandinavica. 2003 Aug;47(7):911-2; author reply 2-3.
23. Abdelmalak B, Maheshwari A, Mascha E, Srivastava S, Marks T, Tang WW, et al. Design and Organization of the Dexamethasone, Light Anesthesia and Tight Glucose Control (DeLiT) Trial: a factorial trial evaluating the effects of corticosteroids, glucose control, and depth-of-anesthesia on perioperative inflammation and morbidity from major non-cardiac surgery. BMC anesthesiology. 2010;10:11.
24. Chen X, Zhao M, White PF, Li S, Tang J, Wender RH, et al. The recovery of cognitive function after general anesthesia in elderly patients: a comparison of desflurane and sevoflurane. Anesthesia and analgesia. 2001 Dec;93(6):1489-94, table of contents.
25. Gaba V. Correlation of the depth of anesthesia with POCD (postoperative cognitive dysfunction). Anesthesia and analgesia. 2007 May;104(5):1298; author reply -9.
26. Ardila A BPH, Braga, L.W, Castro-Caldas A, Judd T,, Kosmidis M.H ME, Nitrini R, Ostrosky-Solis F, Rosselli M. Illiteracy: The Neuropsychology of Cognition Without Reading. Archives of Clinical Neuropsychology. 2010;25 (689–712).
