# Supplementary material for: Effects of Single Low Dose of Dexamethasone before Noncardiac and Nonneurologic Surgery and General Anesthesia on Postoperative Cognitive Dysfunction—A Phase III Double Blind, Randomized Clinical Trial
Source: PLoS One. 2016 May 6;11(5):e0152308. doi: 10.1371/journal.pone.0152308 (PMC4859565; doi:10.1371/journal.pone.0152308)
Supplement: S3 File — (DOC) [file pone.0152308.s003.doc]

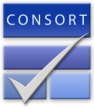
Lista de informações CONSORT 2010 para incluir no relatório de um estudo randomizado

| Seção/Tópico | Item No | Itens da Lista | Relatado na pg No |
| --- | --- | --- | --- |
| Título e Resumo | | | |
|  | 1a | Identificar no título como um estudo clínico randomizado | 8 |
|  | 1b | Resumo estruturado de um desenho de estudo, métodos, resultados e conclusões para orientação específica, consulte CONSORT para resumos | 8 |
| Introdução | | | |
| Fundamentação e objetivos | 2a | Fundamentação científica e explicação do raciocínio | 8-10 |
|  | 2b | Objetivos específicos ou hipóteses | 9 |
| Métodos | | | |
| Desenho do estudo | 3a | Descrição do estudo clínico (como paralelo, factorial) incluindo a taxa de alocação | 8-10 |
|  | 3b | Alterações importantes nos métodos após ter iniciado o estudo clínico (como critérios de eIegibilidade), com as razões |  |
| Participantes | 4a | Critérios de elegibilidade para participantes | 10 |
|  | 4b | Informações e locais de onde foram coletados os dados | 10 |
| Intervenções | 5 | As intervenções de cada grupo com detalhes suficientes que permitam a replicação, incluindo como e quando eles foram realmente administrados | 10-14 |
| Desfechos | 6a | Medidas completamente pré-especificadas definidas de desfechos primários e secundários, incluindo como e quando elas foram avaliadas | 10-14 |
|  | 6b | Quaisquer alterações nos desfechos após o estudo clínico ter sido iniciado, com as razões |  |
| Tamanho da amostra | 7a | Como foi determinado o tamanho da amostra | 14 |
|  | 7b | Quando aplicável, deve haver uma explicação de qualquer análise de interim e diretrizes de encerramento |  |
| Randomização: |  |  |  |
| Seqüência geração | 8a | Método utilizado para geração de seqüência randomizada de alocação | 13 |
|  | 8b | Tipos de randomização, detalhes de qualquer restrição (tais como randomização por blocos e tamanho do bloco) | 13 |
| Alocação mecanismo de ocultação | 9 | Mecanismo utilizado para implementer a seqüência de alocação randomizada (como recipients numerados sequencialmente), descrevendo os passos seguidos para a ocultação da seqüência até as intervenções serem atribuídas | 13 |
| Implementação | 10 | Quem gerou a seqüência de alocação randomizada, quem inscreveu os participantes e quem atribuiu as intervenções aos participantes | 13 |
| Cegamento | 11a | Se realizado, quem foi cegado após as intervenções serem atribuídas (ex. Participantes, cuidadores, assessores de resultado) e como | 13 |
|  | 11b | Se relevante, descrever a semelhança das intervenções |  |
| Métodos estatísticos | 12a | Métodos estatísticos utilizados para comparar os grupos para desfechos primários e secundários | 14 |
|  | 12b | Métodos para análises adicionais, como análises de subgrupo e análises ajustadas | 14 |
| Resultados | | | |
| Fluxo de participantes ( é fortemente recomendado a utilização de um diagrama) | 13a | Para cada grupo, o número de participantes que foram randomicamente atribuídos, que receberam o tratamento pretendido e que foram analisados para o desfecho primário | 15 |
|  | 13b | Para cada grupo, perdas e exclusões após a randomização, junto com as razões | 15 |
| Recrutamento | 14a | Definição das datas de recrutamento e períodos de acompanhamento | 10 |
|  | 14b | Dizer os motivos de o estudo ter sido finalizado ou interrompido |  |
| Dados de Base | 15 | Tabela apresentando os dados de base demográficos e características clínicas de cada grupo | 23 |
| Números analisados | 16 | Para cada grupo, número de participantes (denominador) incluídos em cada análise e se a análise foi realizada pela atribuição original dos grupos |  |
| Desfechos e estimativa | 17a | Para cada desfecho primário e secundário, resultados de cada grupo e o tamanho efetivo estimado e sua precisão (como intervalo de confiança de 95%) | 14 |
|  | 17b | Para desfechos binários, é recomendada a apresentação de ambos os tamanhos de efeito, absolutos e relativos |  |
| Análises auxiliares | 18 | Resultados de quaisquer análises realizadas, incluindo análises de subgrupos e análises ajustadas, distinguindo-se as pré-especificadas das exploratórias | 14 |
| Danos | 19 | Todos os importantes danos ou efeitos indesejados em cada grupo (observar a orientação específica CONSORT para danos) |  |
| Discussão | | | |
| Limitações | 20 | Limitações do estudo clínico, abordando as fontes dos potenciais viéses, imprecisão, e, se relevante, relevância das análises |  |
| Generalização | 21 | Generalização (validade externa, aplicabilidade) dos achados do estudo clínico |  |
| Interpretação | 22 | Interpretação consistente dos resultados, balanço dos benefícios e danos, considerando outras evidências relevantes | 15 |
| Outras informações | | |  |
| Registro | 23 | Número de inscrição e nome do estudo clínico registrado | 10 |
| Protocolo | 24 | Onde o protocolo completo do estudo clínico pode ser acessado, se disponível | 10 |
| Fomento | 25 | Fontes de financiamento e outros apoios (como abastecimento de drogas), papel dos financiadores | 10 |

* Recomendamos fortemente a leitura desta norma em conjunto com o CONSORT 2010. Explicação e Elaboração de esclarecimentos importantes de todos os itens. Se relevante, também recomendamos a leitura das extensões do CONSORT para estudos cluster randomizados, estudos de não-inferioridade e de equivalência, tratamentos não-farmacológicos, intervenções de ervas e estudos pragmáticos. Extensões adicionais estão por vir: para aquelas e até dados de referências relevantes a esta lista de informações, ver [www.consort-statement.org](http://www.consort-statement.org/).
